# Synthetic Heparan Sulfate-like Oligosaccharides as Tools to Decipher Growth Factor Recognition

**DOI:** 10.1021/acs.jmedchem.6c00760

**Published:** 2026-08-20

**Authors:** Joseph Wakpal, Kartikey Singh, Michael Hotor, Livia Philip, Dylan Yost-Slinker, Junzhe Wang, Hawau Abdulsalam, Hien M. Nguyen

**Affiliations:** Department of Chemistry, 2954 Wayne State University, Detroit, Michigan 48202, United States

## Abstract

Heparan sulfate proteoglycans (HSPGs) facilitate interactions between growth factors and their receptors, regulating signaling pathways involved in angiogenesis and cell proliferation. However, the structural heterogeneity and synthetic inaccessibility of native heparan sulfate (HS) have limited systematic exploration of structure–activity relationships (SAR) and the discovery of HS-based inhibitors. Here, we developed 46 aminoglycoside-derived non-native HS mimetics spanning diverse structural and functional group patterns. SAR analysis identified *N*-acetylation and 6-*O*-sulfation as key determinants of HS-FGF2 and HS–VEGF_165_ interaction modulation. Compound **8**, synthesized in two steps, competitively disrupted heparin binding to FGF2 (IC_50_ = 0.23 μM), FGFR1 (IC_50_ = 0.17 μM), and VEGF_165_ (IC_50_ = 0.66 μM), with >140-fold FGF2/FGF4 selectivity and substantially greater potency than native HS pentasaccharide fondaparinux. In NIH3T3 fibroblasts, compound **8** selectively reduced FGF2-induced proliferation in a dose-dependent manner without measurable cytotoxicity, highlighting aminoglycoside-derived HS mimetics as synthetically accessible platforms for modulating growth factor signaling.

## Graphical abstract

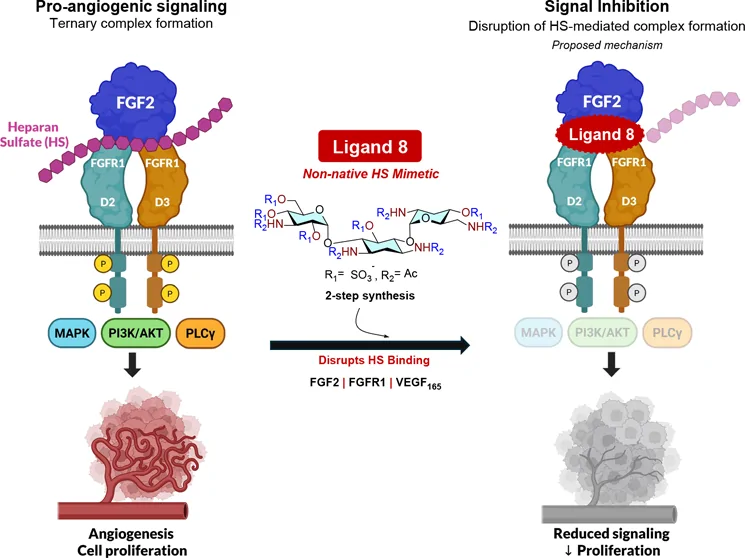

## 1 Introduction

Glycosaminoglycan (GAG)-binding proteins, especially growth factor cytokines like fibroblast growth factor (FGF) and vascular endothelial growth factor (VEGF), are extensively studied for their roles in tumorigenesis, angiogenesis, and proliferative signaling.^1–5^ The link between FGF and VEGF is well recognized, as fibroblast growth factor II (FGF2) is known to promote VEGF-A expression and endothelial revascularization of endothelial cells (Figure 1),^6,7^ contributing to VEGF-independent compensatory angiogenic signaling (Figure 1).^8^

**Figure 1:**
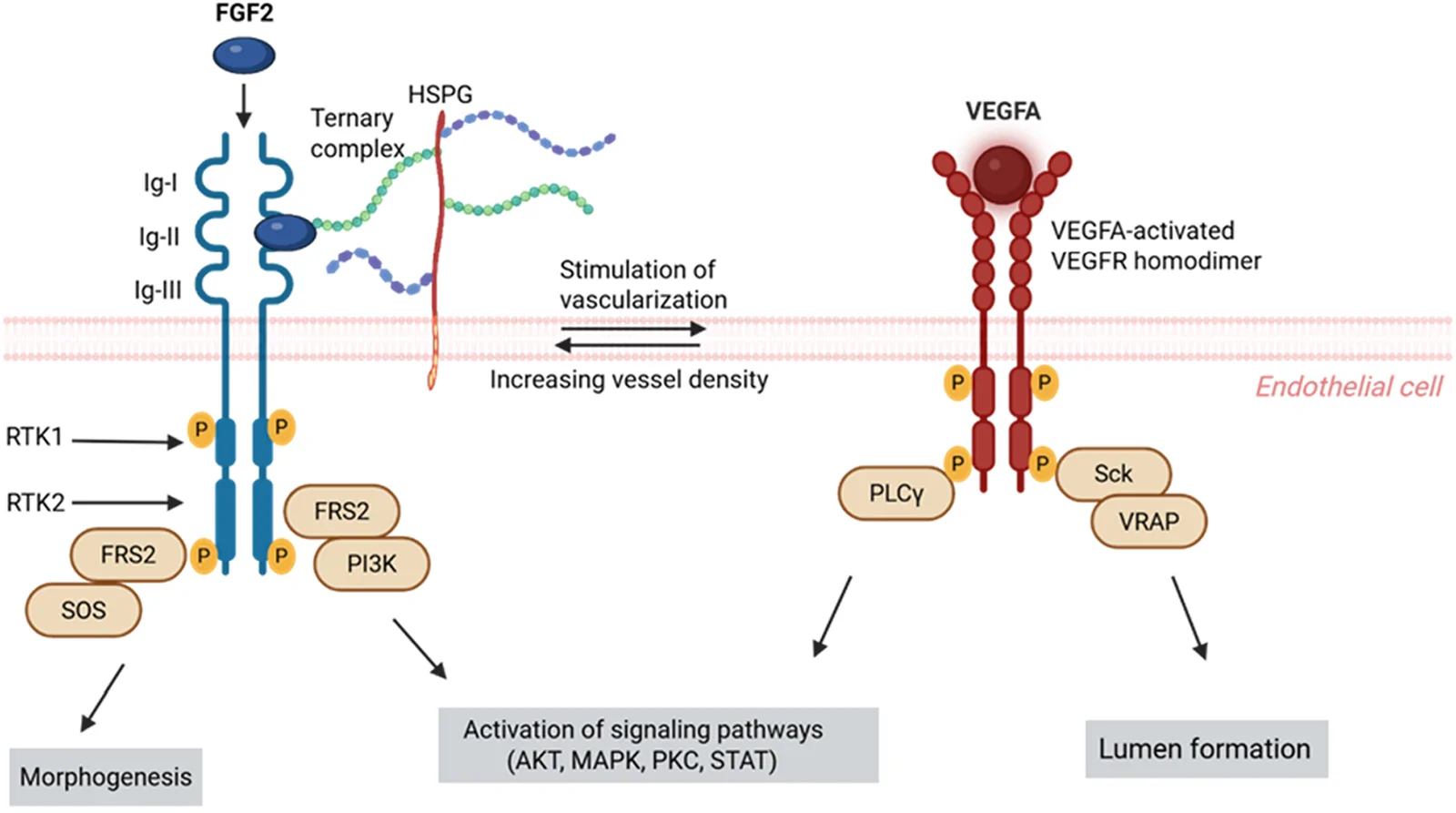
Role of FGF and VEGF in angiogenesis. Activation of VEGF-A by proangiogenic and angiogenic factors stimulates downstream signaling events that promote angiogenesis. FGF overexpression stimulates vasculature growth and promotes VEGF-independent compensatory angiogenic signaling. The binding of HSPG to FGF2 induces FGFR dimerization. This is accompanied by transphosphorylation of receptor subunits (RTK1 and RTK2) and, consequently, initiation of intracellular signaling events. Similar sequences of events are induced by VEGFA activity.

Fibroblast growth factors (FGFs) are cell signaling proteins involved in a wide variety of biological processes.^9^ FGFs bind to FGF receptors (FGFRs) and transmembrane tyrosine kinase receptors.^10–13^ This interaction activates intracellular signaling pathways such as MAPK, PI3K/AKT, and PLCγ, that regulate proliferation, differentiation, and migration (Figures 1 and 2A).^11,14,15^ There are 23 fibroblast growth factors (FGFs), 22 of which are present in humans, classified into seven subfamilies based on sequence and structural homology.^16^ Among them, FGF2, acting through its receptor FGFR1, plays a pivotal role in development, tissue repair, cancer progression, and angiogenesis.^17^ FGF2, a small 18 kDa protein made up of 147 amino acids,^18,19^ contains a heparin-binding domain (HBD), which is rich in basic amino acids, including lysine and arginine residues (Figure 2A–C).^20,21^ This domain enables FGF2 to bind to heparin or heparan sulfate proteoglycans (HSPGs), consequently stabilizing the protein, protecting it from degradation, and facilitating the formation of the FGF2-FGFR-HSPG ternary complex (Figure 2A). This complex is crucial in triggering receptor dimerization and subsequent signaling pathways that regulate cell proliferation, migration, and survival (Figure 1).^22,23^ Dysregulation of this pathway can lead to enhanced tumor growth and metastasis.^10,15,23^ Several inhibitors targeting the FGF2 signaling pathway have been developed to block the kinase domain of FGFR.^24,25^ However, their use is limited due to resistance and side effects.^26,27^ Small molecule inhibitors such as Sm27 and Rosmarinic acid target the extracellular FGFR1-D2-FGF2 interface, but they exhibit low activity and efficacy.^28^ Additionally, the shallow binding pocket of FGF2 (Figure 2B and C)^9,10,15^ poses challenges in designing effective small-molecule inhibitors.

**Figure 2:**
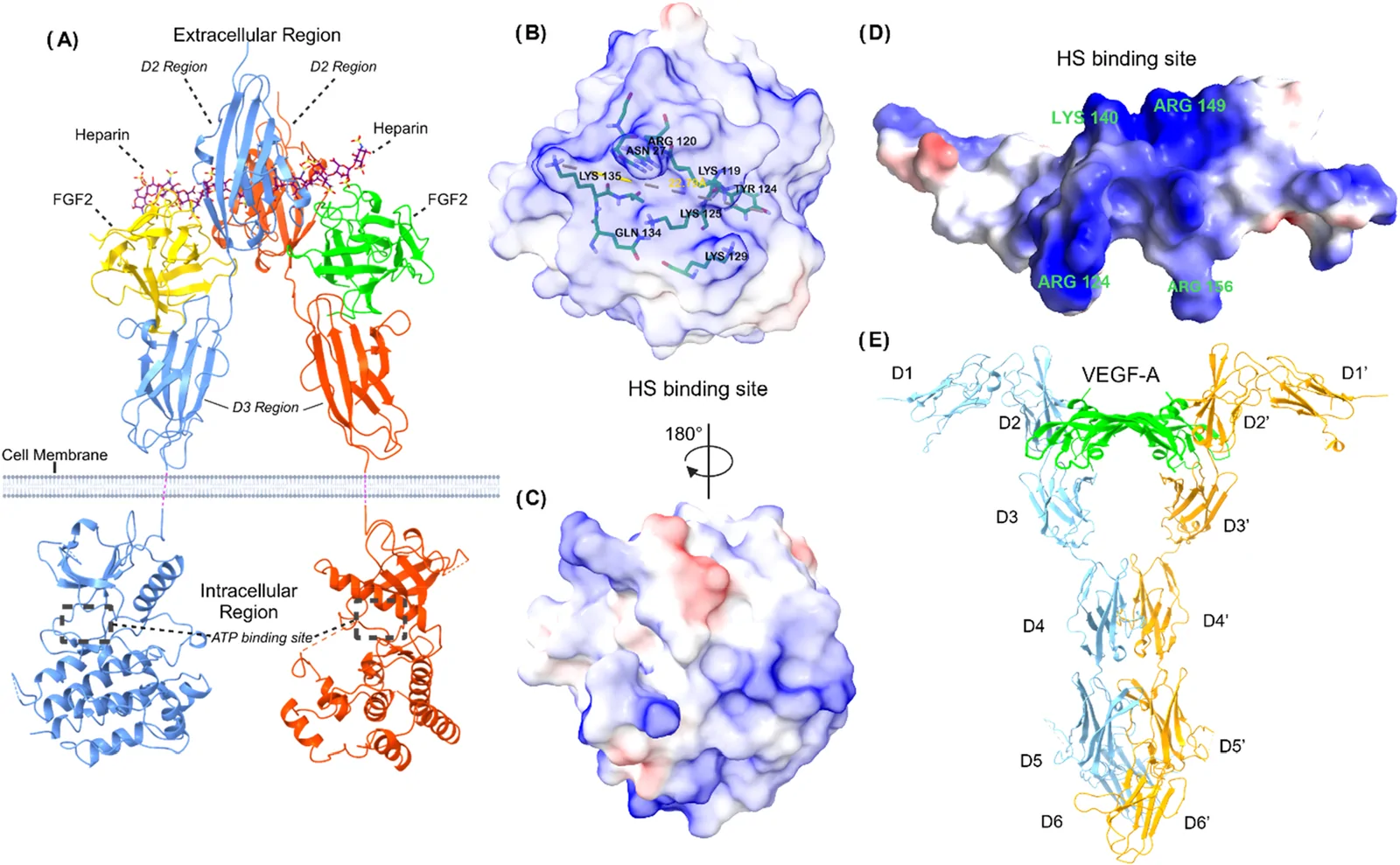
(A) The ternary complex of FGF2-FGFR1-heparin is shown. The extracellular domain contains FGF2-FGFR1-heparin ternary complex, while the intracellular cytoplasmic domain contains the tyrosine kinase core linking the extracellular region via a transmembrane domain. D2 and D3 regions of FGFR1 are color-coded differently and labeled. (B) The electrostatic potential surface of the FGF2 model highlights the electropositive regions of the protein (PDB code: 4OEF for FGF2). The surfaces are color-coded to indicate electrostatic potential, with positive potential in blue and negative potential in red. The figures are rotated by 180° to show the electropositive residues on the opposite side. The electrostatic potential surface of FGF2, displaying the primary heparan sulfate (HS) binding site. (C) Rear view of FGF2, illustrating the second electropositive surface that interacts with the D2 and D3 domains of FGFR1. (D) The heparin-binding domain of VEGF_165_ (PDB code: 2VGH) monomer showing key basic residues in the active site. (E) Structure of the extracellular domain of VEGFR1 in complex with VEGF-A. Six of the seven organized immunoglobulin domains of VEGFR1 are shown with VEGF-A bound to the D2 domain (between D2 and D3 domains). Each monomer ribbon of VEGFR1 is depicted in a different color code, with D1–D6 and D1’–D6’ domains shown in cyan and gold, respectively, while the VEGF-A homodimer is illustrated in green.

VEGF is a 40 kDa glycosylated homodimer from the cystine knot growth factor superfamily, which is categorized into four families based on sequence homology.^29–31^ It acts as an endothelial cell-specific mitogen involved in pathological vascularization and angiogenesis.^32,33^ There are seven VEGF isoforms, with VEGF-A being the most abundant in humans, comprising multiple independent copies (121, 145, 148, 162, 165, 183, 189, and 206) that exhibit distinct biochemical properties and functional components.^6^ VEGF-A-165 (VEGF_165_) is the most dominant isoform with high affinity binding to heparin/HS.^34–36^ Structurally, the VEGF monomer features a central antiparallel β-sheet structure linked by two disulfide bonds, forming the biologically active homodimer (Figure 2D).^37^ Binding of VEGF_165_ to HS stabilizes the growth factor and promotes its interactions with VEGF receptors, enhancing receptor dimerization and activation.^37–40^ VEGF receptors (VEGFR-1, VEGFR-2, and VEGFR-3) subsequently activate intracellular signaling cascades such as Ras/MAPK, PI3K/AKT, and PLCγ.^38,39,41^ Among these interactions, the VEGF_165_–VEGFR2 complex is the primary mediator of endothelial cell migration, proliferation, vascular permeability, and angiogenesis.^42^

As such, our study focused on identifying effective HS mimetics that can simultaneously inhibit FGF2 and VEGF_165_ by blocking their interaction with HSPGs. HSPGs are ubiquitous macromolecules found on the cell surface and within the extracellular matrix (ECM) of various tissues.^43–45^ The basic structure of HSPGs features a protein core with several linear *O*-linked heparan sulfate (HS) chains. These chains are highly complex, consisting of uronic acid and glucosamine residues with various degrees of *O*- and *N*-sulfation, as well as *N*-acetylation on the sugar residues (Figure 3A).^46–48^ These modifications contribute to structural diversity, making HS chains heterogeneous. To gain a comprehensive understanding of the interaction between HS and FGF2/VEGF_165_, it is essential to investigate the structural variability and sulfation patterns of HS glycans, as well as their ability to bind these proteins. However, the complex structure of HS makes elucidating the structure–activity relationship (SAR) studies challenging. The synthesis of HS oligosaccharides often uses natural sequences as a framework. This process often involves 25–40 steps using orthogonally protected monosaccharides, which are not always readily available. These manipulation steps are essential for achieving specific sulfation patterns, which are crucial for studying how HS oligosaccharides interact with growth factors and chemokines.^49–61^ Despite these challenges, a library of HS tetrasaccharides has been synthesized, featuring all potential sulfation patterns in 22–30 steps, enabling the identification of sulfate sequences with high affinity for FGF2 (Figure 3B).^59,62^ In addition, HS disaccharides,^63,64^ HS-like oligosaccharides,^65,66^ a HS tetrasaccharide conjugate,^67^ and homo-oligosaccharides^68^ have been reported to modulate heparan sulfate-growth factor interactions.

**Figure 3:**
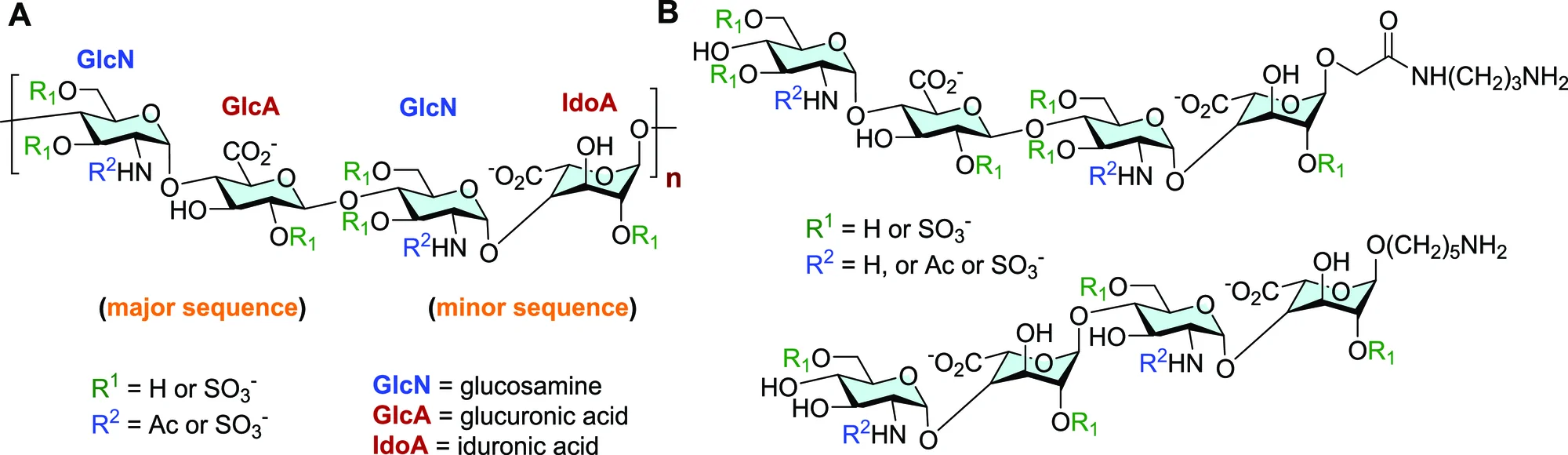
Structure of (A) polymeric heparan sulfate; (B) synthetic heparan sulfate tetrasaccharides.^59,62^

In this study, we present aminoglycosides as an attractive scaffold for the design of non-native HS oligosaccharides owing to their unique structural features, commercial availability, and cost-effectiveness (Figure 4). Unlike native glycosaminoglycans, aminoglycosides do not possess a canonical HS backbone and are therefore viewed as non-native HS-like scaffolds that functionally mimic heparan sulfate through electrostatic and structural complementarity rather than as structural analogs of HS oligosaccharides. Their dense array of hydroxyl and amino groups provides opportunities for chemoselective and regioselective derivatization, enabling systematic installation of *N*-acyl, *N*- and *O*-sulfate, and hydrophobic substituents to investigate growth factor recognition. Three aminoglycoside scaffolds were chosen for investigation (see Figure 4): the small-sized tobramycin and the medium-sized apramycin, both featuring relatively linear pseudo-oligosaccharide structures that resemble heparan sulfate (HS) chains. The larger aminoglycoside, paromomycin, has a branched pseudotetrasaccharide framework, which is hypothesized to provide improved spatial complementarity with the binding pockets of heparin-binding proteins. Using these scaffolds, 46 non-native HS-like oligosaccharides, bearing diverse *N*- and *O*-sulfation patterns, *N*-acyl substituents, and hydrophobic modifications, were investigated to target the cationic heparin-binding domains of FGF2 and VEGF_165_. Notably, the most potent compound bearing the *N*-acetyl-*O*-sulfate groups demonstrated greater affinity for FGF2 and VEGF_165_ in competition assays than the native HS oligosaccharides evaluated in this study.

**Figure 4:**
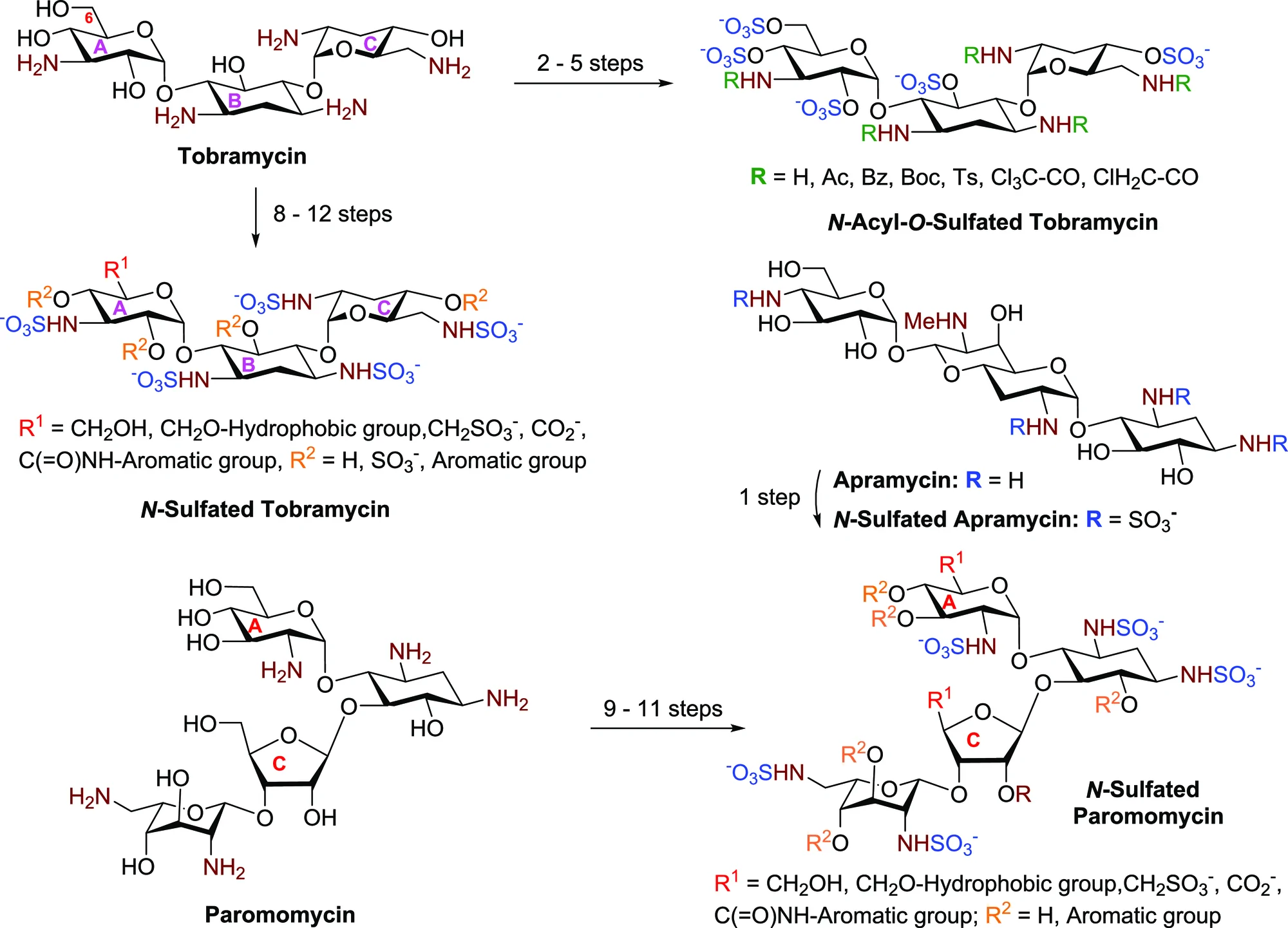
Investigation of non-native HS oligosaccharides from commercially available aminoglycosides such as tobramycin, apramycin, and paromomycin, which consist of a pseudotrisaccharide and pseudo- tetrasaccharide backbone with well-defined *N*-acyl, *N*-and *O*-sulfation patterns and various hydrophobic groups for growth factor recognition.

## 2 Results and Discussion

### 2.1 Synthesis of HS-like Oligosaccharides from Aminoglycosides

In our investigation, the compounds were classified into distinct groups based on specific molecular markers, including sulfation patterns, hydrophobic scaffolds, and chain length (pseudotrisaccharide versus pseudotetrasaccharide). The sulfation patterns correspond to either *O*- or *N*-sulfation on the A–C rings of the pseudotrisaccharide and tetrasaccharide structures (Figure 5). The key feature of these non-native HS oligosaccharides is that Groups A, C, D, and F are *N*-sulfated, whereas Group B bears *N*-acyl substituents. *N*-sulfate content has been extensively studied for HS and protein binding.^69–71^ *N*-acetyl content is a minor component of natural HS/heparin and has been less extensively investigated with respect to its role in protein binding.^70,71^ In addition, the hydroxyl groups of these groups are capped with various functional groups: sulfate (Group B), hydrophobic aryl and cyclic aliphatic rings (Group C), hydrophobic aromatic naphthalene groups (Groups D–F), oleanolic acid (Group E), and biaryl and 1-naphthyl groups (Group F).

**Figure 5:**
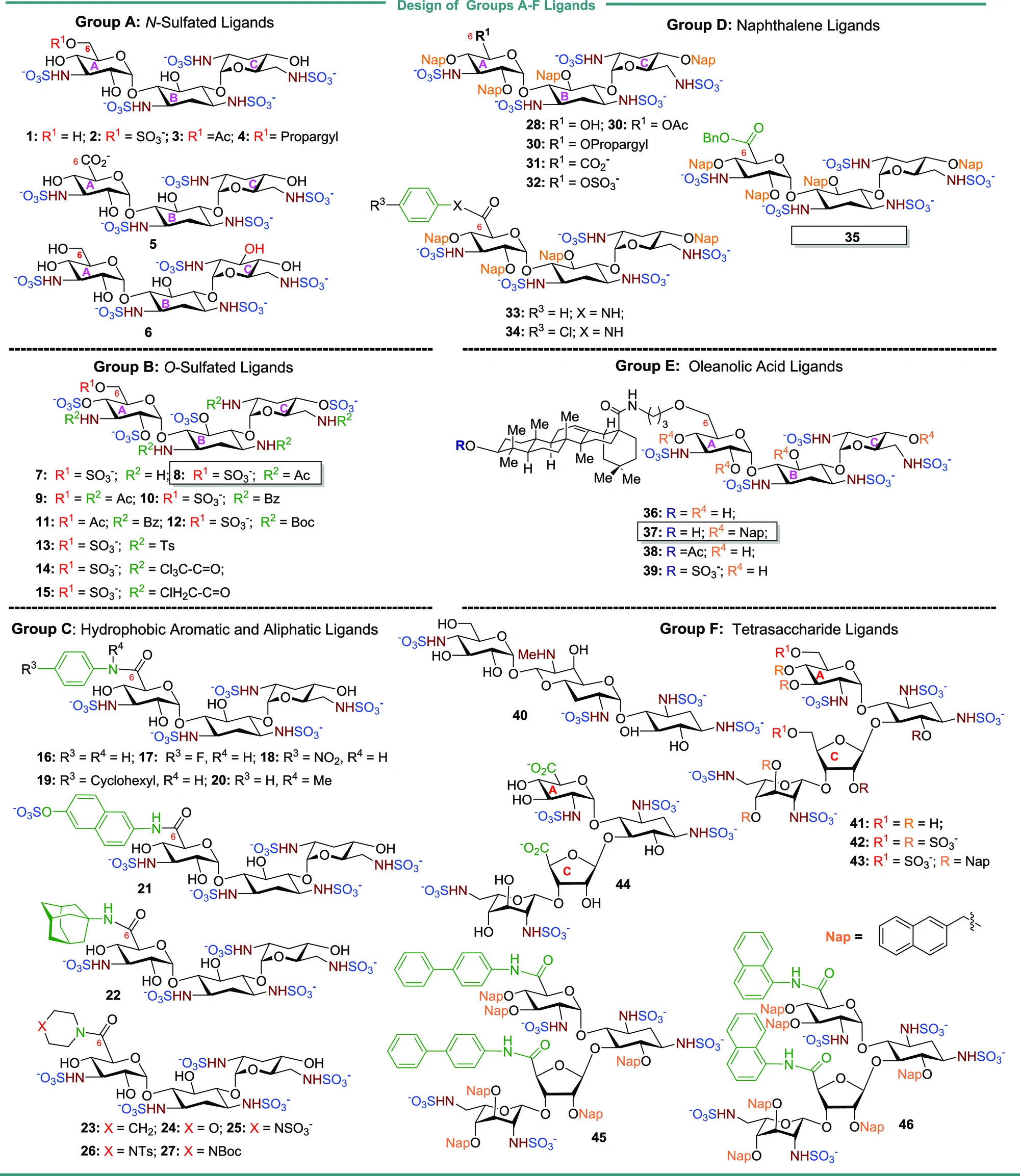
Representative aminoglycoside-based HS-like oligosaccharides derived from tobramycin, paromomycin, and apramycin. The ligands have been grouped based on specific molecular markers. Group A–E are tobramycin derivatives, whereas Group F is both linear and branched tetrasaccharides derived from apramycin and paromomycin, respectively.

We previously studied the impact of modified *N*-sulfated aminoglycosides on the activity of the enzyme heparanase.^72,73^ This study shifts our focus to the novel *N*-acyl compounds and their potential interactions with FGF2, comparing these interactions to those of their *N*-sulfated counterparts. As a result, we focus only on developing strategies for the synthesis of new compounds in group B. The synthesis of *O*-sulfated ligands **7**–**15** in Group B (Figure 5) is shown in Scheme 1. Compound **7** was synthesized in five steps, starting from the azide intermediate 47 of tobramycin, which underwent sulfation followed by hydrogenolysis. When working with azides, it is essential to take adequate precautions, as organic azides can be potentially explosive. To synthesize glycan **8**, tobramycin was first converted into the azide intermediate. The hydroxyl groups at the C6 position and the secondary position were then protected with 2-naphthylmethyl (Nap) groups, resulting in a Nap-capped intermediate. The azide groups on this intermediate were subsequently reduced to yield a free amine intermediate. The resulting crude product containing the free amine was then acetylated, followed by the global deprotection of the Nap groups. This final step exposed the free hydroxyl groups and prepared them for a subsequent sulfation reaction (see Scheme S1). While we can prepare **8** in six steps using the original route, we have discovered an alternative method that requires only two steps. As shown in Scheme 1, we first added acetic anhydride to a solution of tobramycin in methanol to form an *N*-acetylation intermediate. This was followed by *O*-sulfation, resulting in the formation of **8**. The synthesis of **9** was accomplished in five steps. First, 6-*O*-trityl pentaazido-tobramycin was subjected to 2-naphthylmethylation using naphthyl bromide (NapBr) to protect the secondary hydroxyl groups, followed by trityl deprotection and 6-OH acetylation to afford intermediate **48** ^72^ (Scheme S1). Following this sequence, the azide **48** was reduced, and the resulting amine product was treated with acetic anhydride to form the *N*-acetylated intermediate. Finally, the naphthyl group was deprotected, followed by *O*-sulfation to afford **9**. Compound **10** was synthesized by replacing acetic anhydride with benzoic anhydride using a similar route to that for **8**, while compound **11** followed a similar approach to that for **9**. To make **12**, di-*tert*-butyl dicarbonate (Boc anhydride) was added to a solution of tobramycin and sodium bicarbonate in methanol, followed by *O*-sulfation. The addition of *p*-toluenesulfonic anhydride to tobramycin, followed by *O*-sulfation, produced compound **13**. Trichloroacetic anhydride was used to convert tobramycin into **14**. The addition of chloroacetic anhydride to a tobramycin solution in methanol, followed by *O*-sulfation, produced glycan **15** (Scheme 1).

**Scheme 1:**
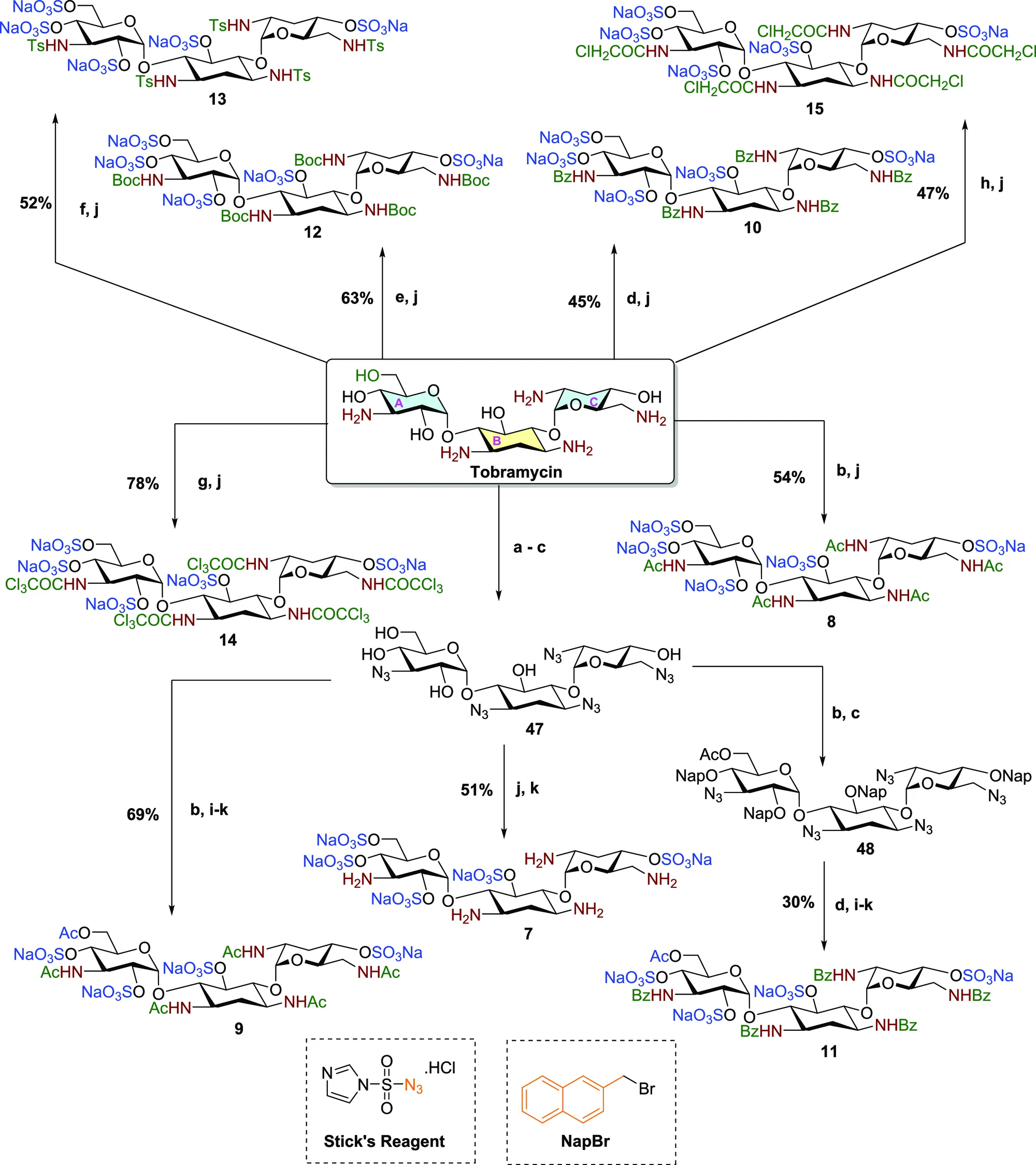
Synthesis of *O*-Sulfated Tobramycin-Based HS Mimetics^a^ ^,^ ^b^

a Reagents and Conditions: (a) Stick’s reagent, CuSO_4_.5H_2_O, K_2_CO_3_, H_2_O, 0 °C–rt, 12 h, 70%; (b) (Ac)_2_O, MeOH, 0 °C–rt, 12 h; (c) NapBr, NaH, TBAI, DMF, 0 °C–rt, 12 h, 79%; (d) benzoic anhydride, MeOH, 0 °C–rt, 12 h; (e) Boc anhydride, NaHCO_3_, H_2_O, THF, 0 °C–rt, 12 h; (f) *p*-Toluenesulfonic anhydride, MeOH, Et_3_N, 0 °C–rt, 12 h; (g) trichloroacetic anhydride, MeOH, 0 °C–rt, 12 h; (h) Chloroacetic anhydride, MeOH, 0 °C–rt, 12 h; (i) propane-1,3-dithiol, DIPEA, DCM/MeOH (2:3) 50 °C, 12 h; (j) SO_3_.Me_3_N, Et_3_N, pyridine, microwave, 100 °C 15 min; (k) 20% Pd/C, H_2_, MeOH/H_2_O (2:1), rt,12 h, 48–55%.
b Na^+^ was Used as a Counterion.

### 2.2 Evaluation of the Binding Affinity of HS-like Oligosaccharides to FGF2

The shallow binding pocket of FGF2 (see Figures S48–S50) enables extensive surface interactions with FGFR1 and heparin, which are crucial for forming the active signaling complex. Dimerization of FGFR1-FGF2 occurs only after heparin/HS binds to FGF2.^18,23^ We evaluated the ability of 46 HS-like oligosaccharides to disrupt the FGF2-heparin binding interaction by performing a biolayer interferometry (BLI) competition assay (Figure 6). Biotinylated heparin was immobilized on a streptavidin biosensor, and the binding of FGF2 to the heparin was monitored. Sulfated ligands were introduced to compete for FGF2 binding, and the effectiveness of each ligand was evaluated by measuring the reduction in heparin binding and calculating half-maximal inhibitory concentrations (IC_50_). It should be noted that this competition format reports disruption of heparin binding to FGF2 and does not directly measure ligand-FGF2 binding affinity or confirm direct occupation of the native HS-binding site on FGF2. A compound could achieve apparent competition through several mechanisms, including direct binding to the heparin-binding domain of FGF2, allosteric effects that reduce FGF2 affinity for heparin, or sequestration of FGF2 in solution away from the immobilized heparin surface.^74^ Therefore, the IC_50_ values reported here reflect the ability of each compound to disrupt the FGF2-heparin interaction and should not be interpreted as direct binding affinities.

**Figure 6:**
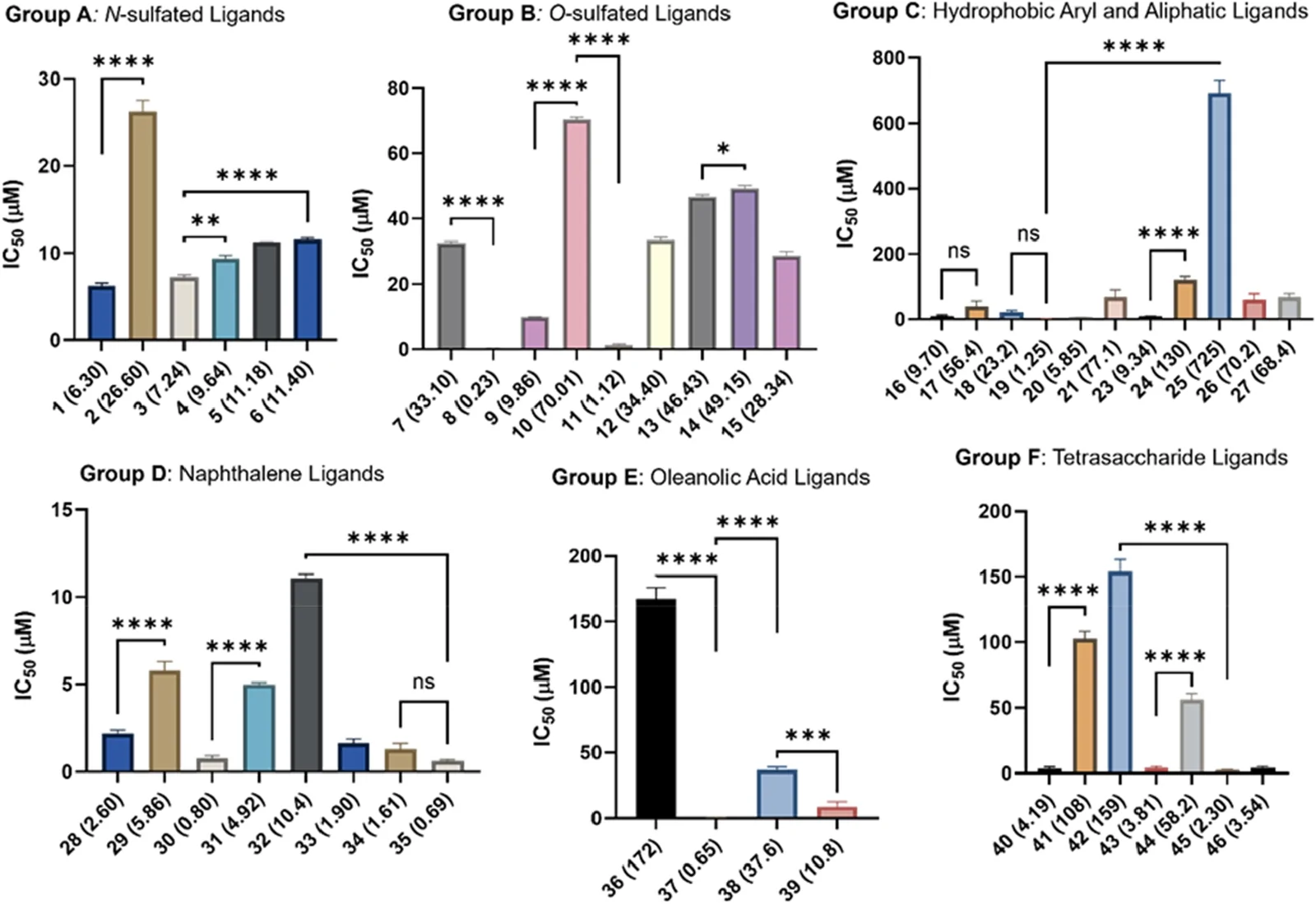
Activity of tobramycin-based HS mimetics against FGF2 using BLI competition assay. Groups A–D show the different classes of ligands tested against the growth factor FGF2. Groups E and F show the activity of the tobramycin-oleanolic acid–based and paromomycin-based ligands, respectively, against the growth factor FGF2. IC_50_ values, shown in parentheses on the *X*-axis (in μM), were calculated using GraphPad Prism, where a sigmoidal curve was fitted to a plot of the percent inhibition against the concentration of the inhibitor. Data are shown as mean ± SEM (*n* = 3). *p < 0.05, **p < 0.01, ***p < 0.001 and ****p < 0.0001.

We first investigated the ability of *N*-sulfated compounds from Groups A and C–F to interact with FGF2 (Figure 6). Our findings indicate that among the Group A compounds, ligand **1**, which contains only *N*-sulfation, has the highest binding affinity to FGF2 with an IC_50_ value of 6.30 μM (Figure 6). Interestingly, incorporating hydrophobic and aromatic groups into the hydroxyl groups significantly improves binding affinity, as demonstrated by compounds **35** (Group D, IC_50_ = 0.69 μM) and **37** (Group E, IC_50_ = 0.65 μM). The most significant finding was observed in *N*-acetyl Group B, where compound **8** exhibited the highest potency (IC_50_ = 0.23 μM), which was 3-fold higher than that of compounds **35** and **37**. Compound **7**, lacking *N*-acetyl groups, showed a 16-fold decrease in activity, highlighting the critical role of *N*-acetyl groups in the saccharide backbone in enhancing ligand-binding affinity for FGF2 (Figure 6). The *N*-trichloroacetyl (**14**) and *N*-chloroacetyl (**15**) derivatives in Group B (Figure 6) both showed moderate activity (IC_50_ > 20 μM). The impact of bulkier groups at the *N*-position, including *N*-Bz (**10** and **11**), *N*-Boc (**12**), and *N*-Tosyl (**13**), was assessed. None of these compounds exhibited greater activity than compound **8**. Overall, among the 46 compounds screened, Group B compound **8** (IC_50_ = 0.23 μM), Group D compound **35** (IC_50_ = 0.69 μM), and Group E ligand **37** (IC_50_ = 0.65 μM) exhibited the highest binding affinity for FGF2. Collectively, these results indicate that optimal FGF2 recognition requires a balance of sulfation pattern, charge density, and steric accessibility, with *N*-acetyl substituents providing a more favorable binding profile than *N*-sulfation and bulky hydrophobic modifications.^75,76^

Next, we performed computational studies to analyze the SAR of these compounds (see Figures S48–S57). The analysis revealed that the shallow binding pocket of FGF2 imposes steric constraints that limit the accommodation of larger ligands. This is evident across Groups C, D, E, and F. Groups C and D share a similar sulfation pattern, but Group D’s hydroxyl groups are functionalized with naphthalene groups on the pseudotrisaccharide scaffold, enabling π–π stacking, hydrophobic interactions, and π-cationic interactions with FGF2 residues, while Group C contains a C6-aromatic amide and participates in more hydrogen bonding interactions due to free hydroxyl groups in the saccharide backbone (see Figure S54). Overall, Group C compounds exhibited low to moderate micromolar efficacy, while Group D ligands retained measurable FGF2 binding despite substantial hydrophobic modification, demonstrating that the scaffold tolerates naphthalene substitution. However, these modifications do not confer greater affinity than the most potent ligand identified in this study, suggesting that additional hydrophobic interactions alone are insufficient to overcome the steric and electrostatic requirements governing recognition at the FGF2 HS binding site. Computational studies suggest that bulky ligands from Groups D–F preferentially localize adjacent to the primary HS binding pocket, rather than within it, consistent with steric exclusion from the shallow binding site and the reduced activity observed experimentally (see Figure S55). Direct experimental confirmation of these predicted binding locations, such as through mutagenesis or X-ray crystallography, has not been performed in the current study and will be the focus of future investigations. BLI data analysis further revealed the low complexation stability and transient binding of Group D ligands to FGF2 (see Figure S9). Group F ligands showed binding in the low micromolar range, with no significant difference in activity observed between the linear compound **40** and branched compounds **41–46** (Figure 6), further supporting the notion that steric bulk is detrimental to FGF2 binding. On the other hand, Group A ligands adopted compact binding orientations within the shallow FGF2 heparin-binding interface, where binding was primarily mediated through hydrogen-bonding and electrostatic interactions with residues lining the positively charged surface (Figures S51 and S53). Although these ligands were accommodated within the binding site, they occupied a relatively smaller portion of the binding interface, which may contribute to their lower experimental activity.

Compared to Groups A and C–F, *N-*acetylation and *O-*sulfation in Group B improved the complementarity of the ligands with the FGF2 heparin-binding interface. These structural modifications enabled the ligands to adopt more extended binding conformations while remaining fully accommodated within the shallow binding groove. Among the Group B analogs, compound **8** exhibited the most extensive binding across the heparin-binding interface, establishing predicted hydrogen-bonding and electrostatic interactions with residues including Lys21, Ser143, Met142, Gln56, Glu96, Arg60, and Val63 (see Figure S52). The arrangement of the *N-*acetyl and sulfate substituents allowed compound **8** to engage multiple regions of the binding interface without exceeding the steric limitations of the shallow FGF2 pocket. These docking results are consistent with the experimental findings, in which compound **8** displayed the highest inhibitory activity against FGF2.

### 2.3 Evaluation of the Binding Affinity of HS-like Oligosaccharides to FGFR1 and Direct Binding to FGF2 and FGFR1

Based on its computational and experimental results, compound **8** was selected for further evaluation against FGF2 and FGFR1. We aimed to target the heparin/HS-binding pocket in FGFR1’s extracellular domain, which facilitates receptor dimerization with FGF2 and heparin/HS (Figure 2A). Using an FGFR1 variant (Pro154 to Leu358) that excludes the kinase domain, we found that compound **8** exhibited strong binding affinity (IC_50_ = 0.17 μM), compared with compound **37** (IC_50_ = 14.1 μM) (Figure 7A). This finding is consistent with the results obtained for FGF2 (Figure 6). To directly evaluate protein binding, we performed BLI experiments in which biotinylated FGF2 or FGFR1 was immobilized on streptavidin tips and exposed to increasing concentrations of compound **8**. This format is mechanistically orthogonal to the heparin competition assay and enables direct measurement of ligand-protein interaction without competition-based inference. Compound **8** bound both proteins with nanomolar affinities: FGF2 (*K*_d_ = 0.35 μM) and FGFR1 (*K*_d_ = 0.85 μM) (Figure 7B), confirming direct target engagement. The higher affinity observed for FGF2 suggests preferential interaction with the growth factor, while binding to FGFR1 supports its ability to engage the receptor component of the signaling axis. Together, these results support the hypothesis that compound **8** modulates FGF2/FGFR1 signaling through direct interactions with both proteins.

**Figure 7:**
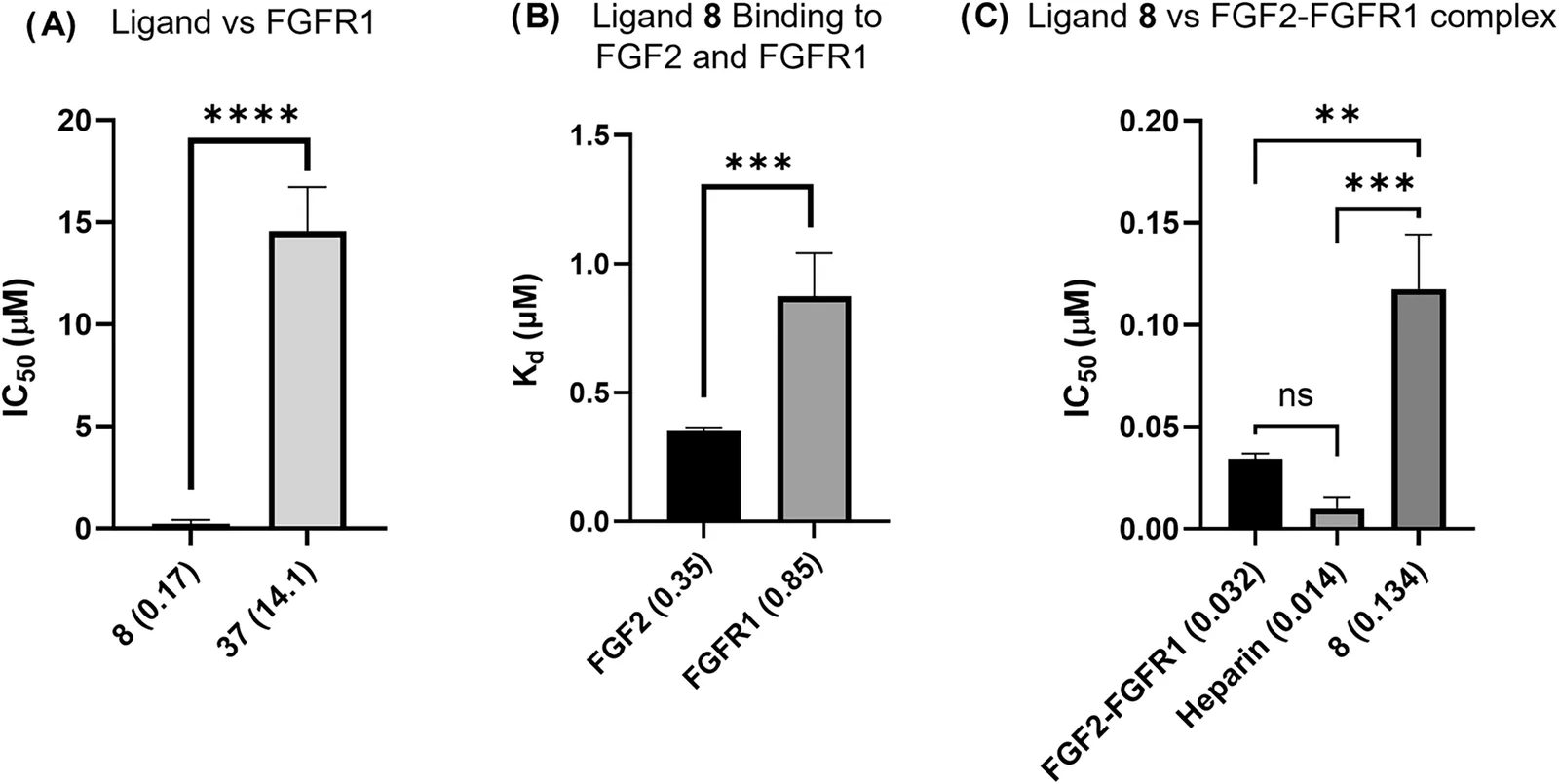
(A) Binding activity of selected HS mimetics against FGFR1. (B) Direct binding (*K*_d_) values of compound **8** to FGF2 and FGFR1. (C) Activity of heparin and compound **8** against the dimeric FGF2-FGFR1 complex and the FGF2-FGFR1 complex alone was used as a control. IC_50_ values are displayed in parentheses on the *X*-axis. IC_50_ values, shown in parentheses on the *X*-axis (in μM), were calculated using GraphPad Prism, where a sigmoidal curve was fitted to a plot of the percent inhibition against the concentration of the ligand. Data are shown as mean ± SEM (*n* = 3). *p < 0.05, **p < 0.01, ***p < 0.001 and ****p < 0.0001.

We performed additional BLI assays to assess the ability of **8** to disrupt FGF2-FGFR1 complex formation (Figure 7C). Compound **8** or heparin was premixed with FGFR1, and biotinylated FGF2 was immobilized on streptavidin biosensor tips to monitor the interaction. We first measured the binding affinity of FGF2 to FGFR1 (IC_50_ = 0.032 μM, Figure 7C). Compound **8** appeared to significantly interfere with FGFR1-FGF2 complex formation, reducing the observed binding affinity by a factor of 4 (IC_50_ = 0.134 μM, Figure 7C). In contrast, heparin enhanced the binding affinity between FGF2 and FGFR1 by a factor of 2 (IC_50_ = 0.014 μM), consistent with its known role in promoting receptor dimerization. These results suggest that **8** may compete with heparin for binding to FGFR1 and FGF2; however, it does not appear to replicate heparin’s ability to promote FGF2-FGFR1 dimerization.

FGF2 signaling is mediated through the assembly of a higher-order complex comprising FGF2, FGFR, and HS/heparin, which facilitates receptor dimerization and subsequent autophosphorylation.^18,20,23^ Multiple structural models of the FGF2–FGFR–HS signaling complex have been reported and are generally consistent with available biological and biochemical data.^18,20,23^ Accordingly, the computational studies presented here were performed using isolated FGF2 and FGFR1 as a simplified model system to evaluate relative ligand-binding trends and potential interaction modes. Although the isolated FGF2 and FGFR1 models do not represent the full FGF2–FGFR–HS assembly, they provide structural insights into the activity of our compounds.

Next, an MD simulation of compound **8** was conducted to assess its stability for interactions with FGF2. The MD simulation of **8** bound to FGF2 was extended to 250 ns, demonstrating a stable binding mode throughout the simulation (see Figure S68). Since these simulations were performed on the nanosecond time scale, the reported poses should be interpreted as representative binding models rather than fully converged free-energy minima.

Our simulations suggest a potential hierarchy in binding site engagement that may correlate with the observed SAR. Compound **8** is predicted to form an intramolecular hydrogen bond between the *N*-acetyl group and the two sugar rings and appears to predominantly associate with the D2 domain near the D2-D3 linker region of FGFR1, with possible interactions involving key HS-binding residues Lys163, Lys172, Lys175, and Lys177 (Figure 8E). During the simulation, a conformational change in the D2-D3 linker region appeared to allow compound **8** to additionally interact with His286 in the D3 domain while maintaining associations with the D2 domain. In contrast, compounds **1**, **16**, and **37** were predicted to associate predominantly with the D3 domain (see Figures S58–S62), with little engagement of the D2 domain. Since the D2 domain has been reported to play an important role in heparin and FGF2 binding, the predicted engagement of both the D2 and D3 domains by **8** may offer a plausible explanation for its superior activity in the BLI competition assay relative to ligands predicted to associate exclusively with D3 (see Figure S61). However, these computationally predicted interactions require experimental confirmation through mutagenesis or direct structural studies before any mechanistic conclusions can be drawn.

**Figure 8:**
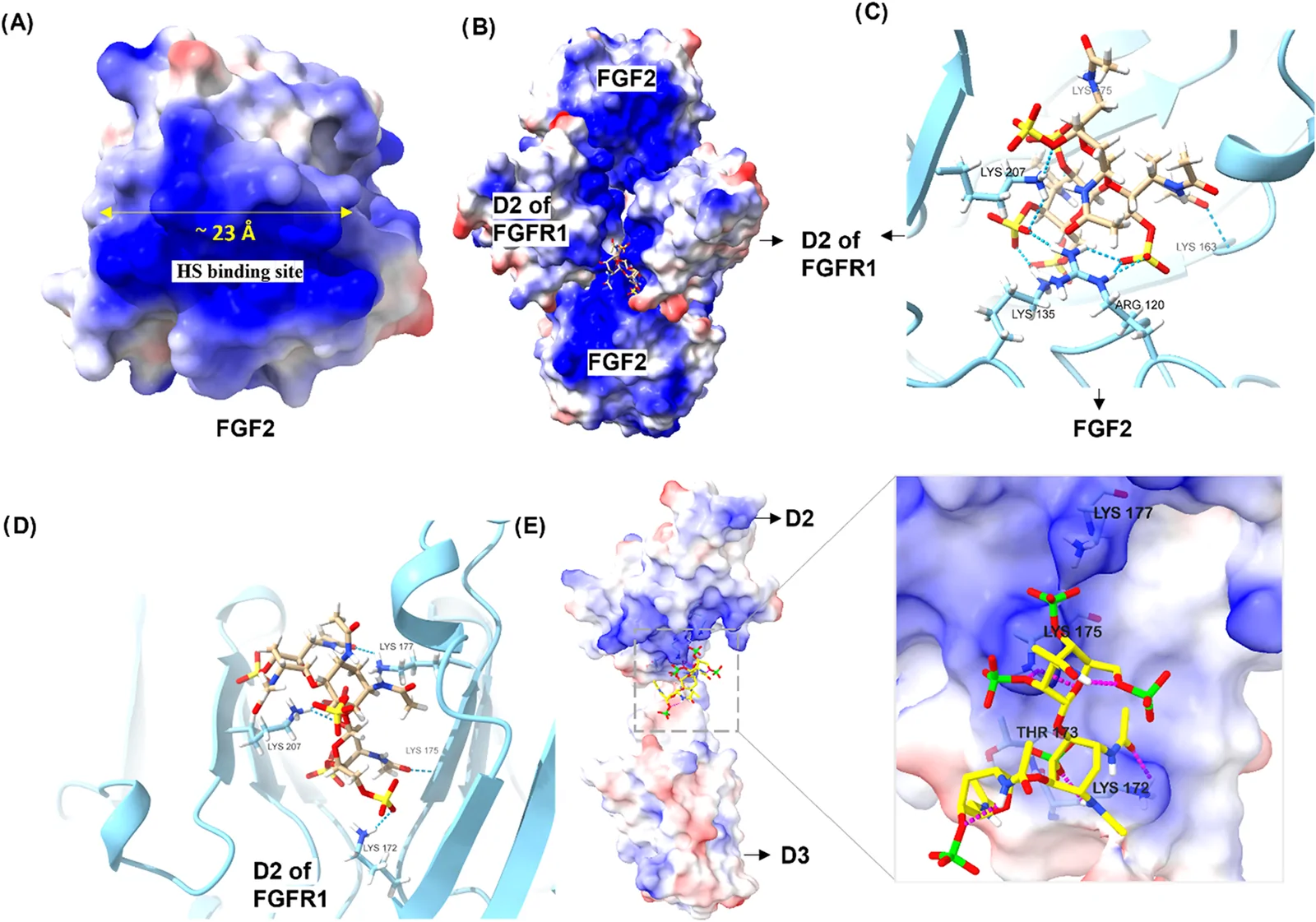
(A) The electrostatic potential of the FGF2 structure. (B) Compound **8** bound to FGF2 and the extracellular D2 region of FGFR1. (C) The binding interaction of **8** and D2 of FGFR1 in complex with FGF2. (D) The binding interaction of compound **8** and the D2 region of FGFR1. (E) Interactions between **8** for a representative snapshot at the end of the MD simulation and key residues in the binding pocket of FGFR1. Zoomed-in depiction of the interactions between **8** and FGFR1 at the end of the MD simulation, and key residues in the binding pocket are shown to the right.

The effect of FGF2 on the predicted binding pose of compound **8** provides additional structural insight. In the absence of FGF2, **8** is predicted to occupy a deeper position in the heparin/HS pocket of FGFR1, with potential interactions involving residues Lys172, Lys175, and Lys177 of one D2 chain and Lys207 of the opposite D2 chain (Figure 8D,E). Throughout the 250 ns simulation, **8** appears highly stable within the binding domain of FGF2, exhibiting minimal fluctuations (see Figure S68). In the presence of FGF2, **8** appears to partially withdraw from the inner binding pocket, maintaining a predicted interaction with Lys207 while shifting outward to potentially interact with Lys135 and Arg120 in the HS binding domain of FGF2, with a possible new hydrogen bond with Lys163 of FGFR1 stabilizing this outward position (Figure 8C). These observations suggest that **8** may adopt different binding poses depending on the presence or absence of FGF2, though direct experimental confirmation of these poses has not been obtained in the current study.

While these biophysical studies demonstrate that **8** interferes with the FGF2-FGFR1 interaction at the molecular level, we next sought to determine whether this disruption translates into functional inhibition of FGF2-induced signaling in cells. To assess whether **8** can modulate FGF2-induced signaling, NIH3T3 fibroblast cells were treated with exogenous FGF2 in the presence or absence of **8**. We first assessed the cytotoxicity of compound **8** in NIH3T3 cells. The results showed no cytotoxic effects at concentrations up to 40 μM (Figure 9A). This finding suggests that compound **8** has a favorable *in vitro* safety profile in fibroblasts, enabling further evaluation of its functional effects. Next, NIH3T3 cells were treated with varying concentrations of FGF2 (Figure 9B). Treatment with FGF2 resulted in a dose-dependent increase in cell viability, reaching approximately 18% at 20 ng/mL and 39% at 100 ng/mL. Co-treatment of cells with FGF2 (20 ng/mL) and compound **8** (0.5–20 μM) resulted in a concentration-dependent reduction in FGF2-induced proliferation, with 20 μM of compound **8** nearly restoring viability to baseline levels (Figure 9C). These results indicate that **8** effectively inhibits FGF2-mediated cellular responses *in vitro*, consistent with its proposed mechanism as a heparan sulfate mimetic that interferes with FGFR activation. This shift in cellular behavior aligns with our molecular dynamics data, supporting a mechanism in which **8** disrupts signaling complex assembly by engaging the D2–D3 linker region of FGFR1. While FGF family members share a common heparin-binding scaffold, the potency of compound **8** and its specific interaction with the FGFR1 linker suggest a degree of selectivity, which will be further evaluated across other FGF isoforms in future studies. Although the observed cellular effects are consistent with the mechanism proposed by the MD simulation studies, pathway-level validation of D2-D3 linker disruption, such as receptor phosphorylation or downstream signaling readouts (e.g., pFGFR1, pERK), was not pursued in the current study and remains an important direction for future work.

**Figure 9:**
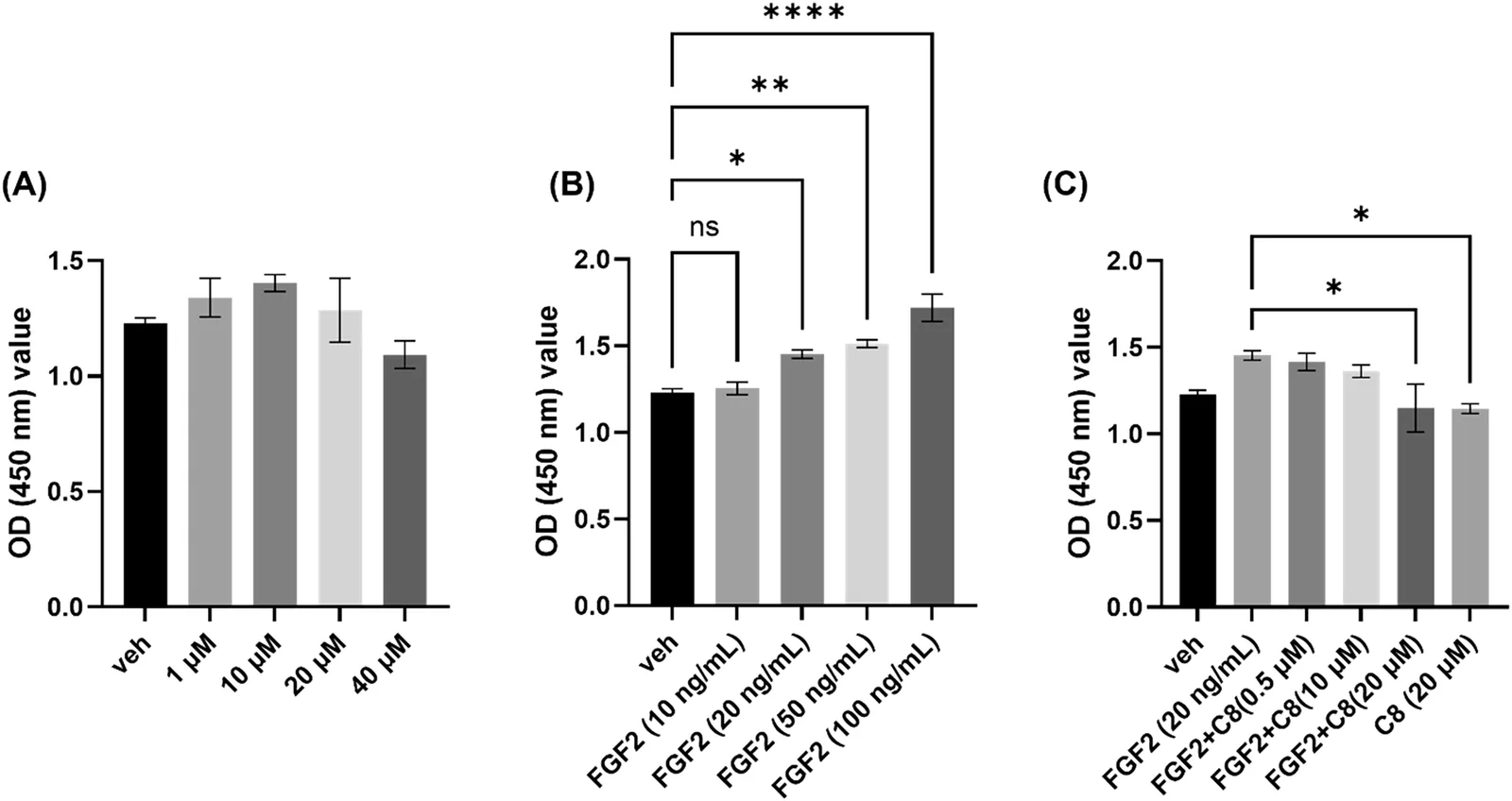
Effect of compound **8** on NIH3T3 cell metabolic activity measured by CCK-8 assay. (A) NIH3T3 cells were treated with compound **8** (1–40 μM) for 24 h, and OD450 values were measured to evaluate potential cytotoxicity. (B) FGF2 treatment increased OD450 signals in NIH3T3 cells in a dose-dependent manner. (C) Co-treatment with compound **8** (**C8**) attenuated the FGF2-induced increase in OD450 signal. Data are presented as mean ± SEM from three independent experiments (*n* = 3). Statistical significance was analyzed using one-way ANOVA with Tukey’s post hoc test. *p < 0.05, **p < 0.01, ***p < 0.001 and ****p < 0.0001.

### 2.4 Evaluation and Comparison of the Binding Affinity of the Non-Native HS-like Oligosaccharides and the Native HS Oligosaccharides to FGF2, VEGF_165_, and FGF4

Previous studies have identified a heparan sulfate tetrasaccharide as the minimal structural unit necessary for effective interactions with growth factors.^59,62^ In light of this finding, we synthesized the native HS tetrasaccharide **49**,^62^ which mimics the highly sulfated region of heparin, and examined its binding affinity to FGF2, alongside the readily available heparin pentasaccharide fondaparinux **50** (Figure 10A). It is important to note that compounds **49** and **50** are structurally and functionally distinct. Tetrasaccharide **49** represents the highly sulfated repeat region of heparin,^59^ while fondaparinux **50** is a specialized pentasaccharide sequence optimized for anticoagulant activity through its selective interaction with antithrombin,^77^ containing distinct saccharide residues including glucuronic acid (GlcA) and 3,6-*O*-sulfate-*N-*sulfate glucosamine (6,3S-GlcNS) that are not representative of the general heparin-binding sequences recognized by FGF2 or VEGF_165_. As such, fondaparinux was not included as a direct structural comparator for FGF2 or VEGF_165_ binding, but rather to explore whether chain length and sulfation pattern influence activity in this assay context, given that compound **8** is a pseudo-trisaccharide, compound **49** is a tetrasaccharide, and fondaparinux **50** is a pentasaccharide. These comparisons should therefore be interpreted with this important structural and functional context in mind. Our analysis also included the most potent non-native HS-like trisaccharides **8**, **35**, and **37** in relation to FGF2. To our excitement, the non-native ligands **8**, **35**, and **37** demonstrated significantly higher affinity for FGF2 than the native HS oligosaccharides **49** and **50**, which showed minimal activity against FGF2 (IC_50_ > 400 μM, Figure 10A). This result underscores the potential of non-native HS oligosaccharide derivatives in enhancing growth factor interactions.

**Figure 10:**
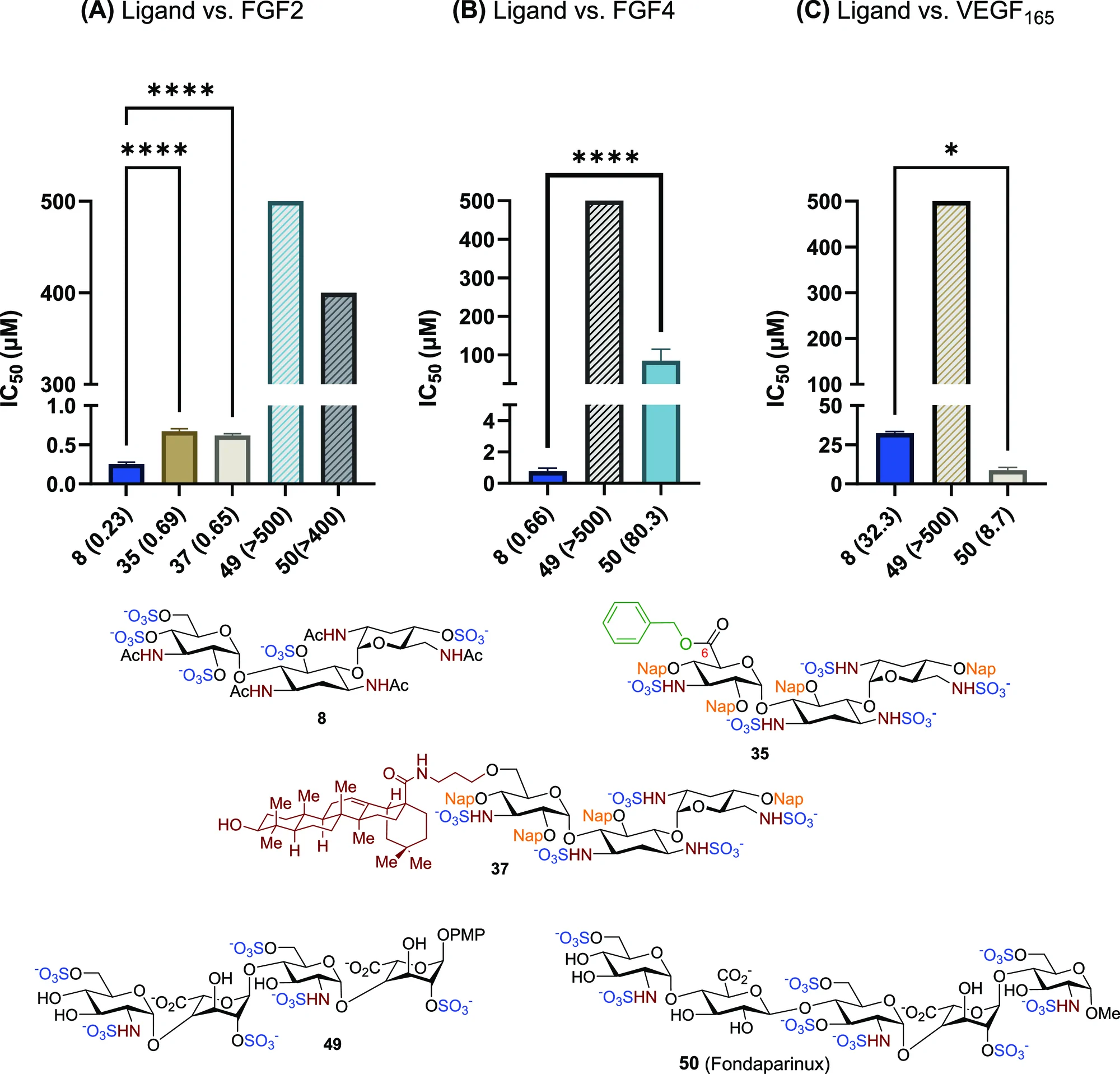
Binding activity of the most potent non-native HS-like oligosaccharide as well as the native HS tetrasaccharide **49**, and pentasaccharide fondaparinux **50** against FGF2, FGF4, and VEGF_165_ using BLI competition assay. (A) The binding activity of the non-native HS mimetics **8**, **35**, and **37**, as well as **49** and **50**, against FGF2. (B) The binding activity of **8**, **49**, and **50** against FGF4. *p < 0.05, one-way ANOVA with Tukey’s post hoc test. (C) The binding activity of **8**, **49**, and **50** against VEGF_165_. IC_50_ values, shown in parentheses on the *X*-axis (in μM), were calculated using GraphPad Prism, where a sigmoidal curve was fitted to a plot of the percent inhibition against the concentration of the inhibitor. Data are shown as mean ± SEM (*n* = 3). *p < 0.05, **p < 0.01, ***p < 0.001 and ****p < 0.0001.

Next, we compared the binding affinity of compounds **8**, **49**, and **50** for FGF4 using a competitive BLI assay (Figure 10B). FGF4 is a 22-kDa signaling protein in the FGF family that plays a key role in embryonic development, cell proliferation, and differentiation.^78^ Similar to FGF2, it interacts with heparin/HS and activates FGF receptors (FGFRs), particularly FGFR2. The results revealed that compound **8** exhibits over 140-fold selectivity for FGF2 (IC_50_ = 0.23 μM, Figure 10A) compared to FGF4 (IC_50_ = 32.3 μM, Figure 10B). Interestingly, while the native HS tetrasaccharide **49** displayed minimal activity against both FGF2 and FGF4 (IC_50_ > 400 μM), the HS pentasaccharide fondaparinux **50** demonstrated pronounced selectivity for FGF4 (IC_50_ = 8.7 μM, Figure 10B).

The varying binding stability of the non-native compound **8** and the native HS oligosaccharides **50** toward FGF2 and FGF4 highlights their specificity for different targets. Hence, we investigated **8**, **49**, and **50** for binding to VEGF_165_ (Figure 10C). VEGF_165_, the dominant isoform of VEGF-A in humans, has distinct biochemical properties and functional components. The VEGF monomer structure (see Figure S69) features a central antiparallel four-stranded β-sheet bearing a cystine knot (two disulfide bonds) at one end. The two symmetrical disulfide bonds covalently link the monomers via Cys51 and Cys60 to form the dimer, which contains positively charged heparin-binding domains^37^ enabling it to bind heparin/HS with high affinity.^34–36^ The results show that compound **8** has a strong binding affinity for VEGF_165_ (IC_50_ = 0.66 μM; Figure 10C) and exhibits 3-fold lower selectivity than FGF2. The non-native HS ligand **49** exhibited no activity against VEGF_165_ (IC_50_ > 500 μM), while **50** showed some binding affinity for VEGF_165_ (IC_50_ = 80.3 μM, Figure 10C).

To explain the high binding affinity of the non-native HS oligosaccharide **8** compared with other compounds, docking studies were conducted. The results revealed that compound **8** binds to the heparin-binding domain (HBD) of VEGF_165_ (PDB code: 2VGH, the A monomer) through key interactions involving three *N*-acetyl groups. Specifically, two *N*-acetyl groups from ring B form hydrogen bonds with Arg13 and Glu12, while another *N*-acetyl from sugar ring A engages in a divalent hydrogen bond with Arg49. Additionally, two *O*-sulfate groups of ring A interact with Arg14 and Arg13, and the saccharide backbone participates in hydrophobic interactions (Figure 11). These interactions enhance the binding stability of compound **8**, thereby increasing its activity.

**Figure 11:**
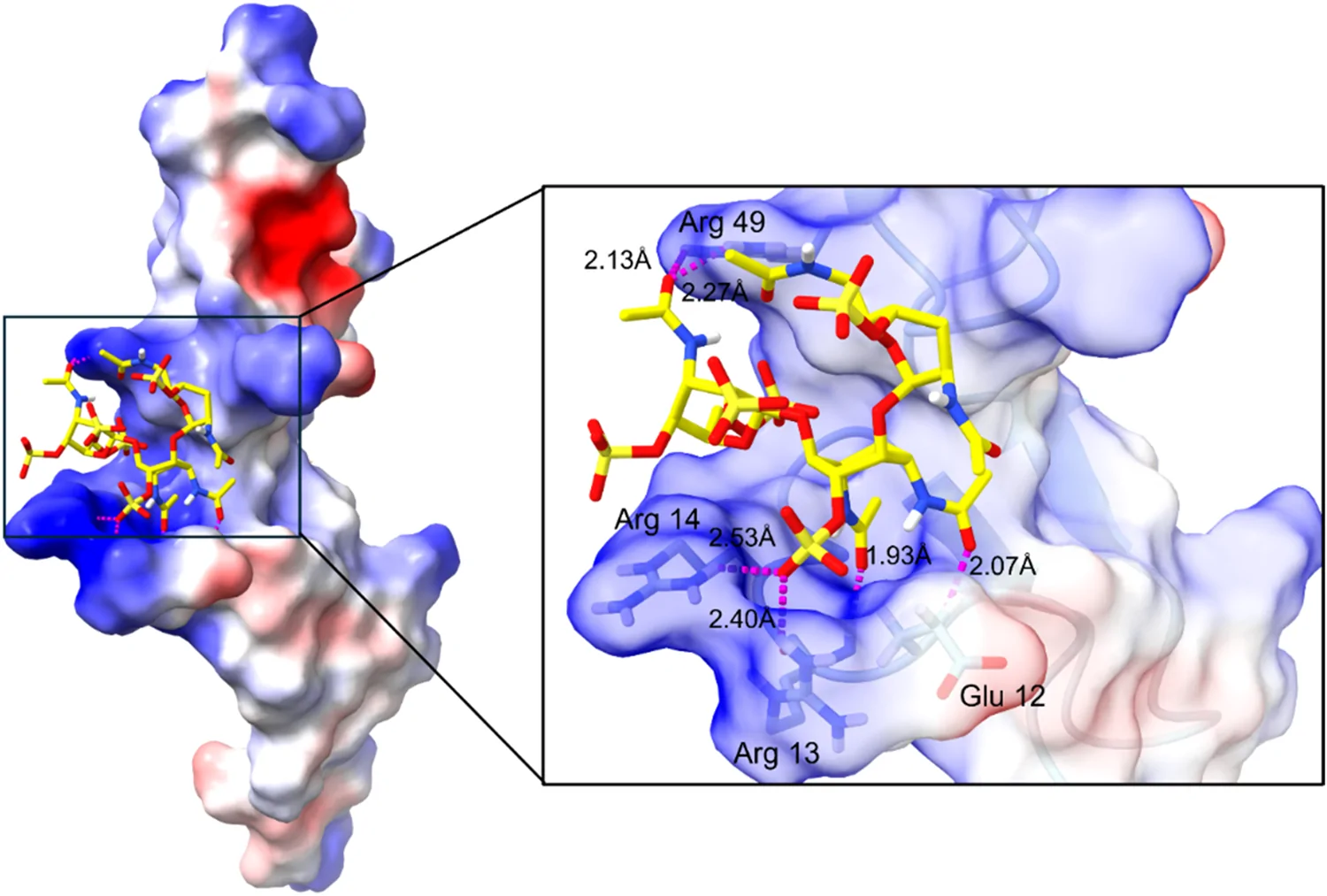
Compound **8** bound to the HS binding domain of VEGF_165_ monomer.

Finally, it should be noted that direct comparisons between non-native HS-like oligosaccharides and native HS oligosaccharides must be interpreted with caution, as differences in molecular size, charge presentation, conformational flexibility, and assay context can all influence apparent competition behavior.^69,79^ Furthermore, it is important to clarify what our non-native compounds mimic. We propose that compound **8** primarily acts via electrostatic competition, leveraging its *O*-sulfate and *N*-acetyl groups to mimic the features of heparan sulfate and thereby competing with native HS for binding to the heparin-binding domains of FGF2 and VEGF_165_, rather than replicating the specific sequence-recognition features of native HS oligosaccharides.

## 3 Conclusion

In this study, we investigate a library of 46 compounds, prepared in 2–12 steps from readily available aminoglycosides, as non-native HS oligosaccharides to elucidate their interactions with growth factors. Using integrated computational and experimental approaches, we systematically interrogated the SAR of this scaffold class across FGF2, FGFR1, and VEGF_165_ proteins. SAR analysis revealed *N*-acetylation and 6-*O*-sulfation as critical determinants for binding activity, while increased steric bulk generally reduces affinity, consistent with the shallow and electropositive binding surface of FGF2. In contrast, VEGF_165_ features a well-defined pocket with two heparin-binding domains, enabling simultaneous interactions with HS chains. Within this framework, compound **8** emerged as the most potent and broadly active compound across all assay formats. It disrupted heparin-binding to FGF2, FGFR1, and VEGF_165_, exhibited nanomolar binding to both FGF2 (*K*_d_ = 0.35 μM) and FGFR1 (*K*_d_ = 0.85 μM), and showed >140-fold selectivity for FGF2 over FGF4, outperforming native HS tetrasaccharide and pentasaccharide in competition assays. These results highlight the importance of the charge distribution of *O*-sulfation and *N*-acetylation in modulating growth factor recognition and demonstrate that non-native aminoglycoside scaffolds can exceed the performance of native HS sequences.

In NIH3T3 fibroblasts, compound **8** reduced FGF2-induced cell proliferation in a dose-dependent manner without measurable cytotoxicity, providing preliminary cellular validation consistent with its biophysical activity profile. Collectively, these findings establish aminoglycoside-derived non-native HS mimetics as a synthetically accessible platform for disrupting heparan sulfate-mediated growth factor interactions, with compound **8** serving as a well-characterized lead scaffold. Future studies will focus on pathway-level inhibition, including receptor phosphorylation and downstream signaling readouts, as well as functional evaluation in angiogenesis-relevant endothelial models and broader profiling across the FGF family to define selectivity boundaries and translational potential of this scaffold class.

## 4 Experimental Section

### 4.1 Chemistry

#### 4.1.1 Reagents and Methods

All reactions were performed with an oven-dried flask fitted with a rubber septum under inert argon conditions unless otherwise stated. All organic solvents used were either anhydrous as purchased from the vendor (Sigma-Aldrich, Alfa Aesar, TCI, and Combi-Blocks) or dried with a solvent purification system. Organic solvents from aqueous workup were concentrated with a Buchi rotary evaporator at 35 °Cor less and 25 Torr. Reaction progress was monitored with analytical thin-layer chromatography (TLC) plates (TLC silica gel 60 F_254_) with 230–400 mesh silica gel. Products and intermediates were visualized with UV light and stained with 5% H_2_SO_4_ for sugar compounds and ceric ammonium molybdate stain for nonsugar. Column chromatography was performed using 40–63 μm silica gel (SiliaFlash F60 from Silicycle) or Sephadex LH-20 from Cytiva. Heparin-biotin (18 kDa, 1 biotin per HP polymer) was purchased from Creative PEGWorks. Streptavidin biosensors were purchased from Sartorius. HBS-EP+ buffer (10 mM HEPES, pH 7.4, 150 mM NaCl, 3.0 mM EDTA, and 0.005% (v/v) surfactant tween20) was purchased from Cytiva. Details of buffer and enzyme concentrations, as well as additional information, can be found in the protocols. Fondaparinux was purchased from Millipore Sigma (Catalog No. SML1240–5MG).

#### 4.1.2 Instrumentation

Newly synthesized compounds were analyzed by NMR spectroscopy and high-resolution mass spectrometry. ^1^H NMR spectra were recorded on either Agilent 400 or 600 MHz spectrometers, while ^13^C NMR spectra were recorded with Agilent 151 MHz or Bruker 126 MHz spectrometers. The NMR chemical shifts are relative to the solvent residual peak and are expressed in parts per million (δ scale). Deuterated solvents used include: (CDCl_3_: δ 7.27 ppm, δ 77.16 ppm; D_2_O: δ 4.79 ppm; and CD_3_OD (d-4): δ 3.31 ppm, δ 49.00 ppm). Automatic phasing and polynomial baseline correction features of the MestReNova software were used to process the acquired NMR data. Data presented as follows: chemical shift (s: singlet, d: doublet, t: triplet, q: quartet, m: multiplet), integration, and coupling constant in hertz (Hz). Low-resolution mass spectrometry was recorded using negative mode ESI-MS with the Shimadzu LCMS-2020 instrument. High-resolution mass spectrometry was recorded with Thermo Orbitrap Exploris 120 in the Lumigen Instrument Center Mass Spectrometry and Analytical Instrument Core at Wayne State University. Compounds **1–46** were characterized by NMR and HRMS.

#### 4.1.3 General Synthetic Procedures

The synthesis of aminoglycoside-based HS mimetics (Groups A, C, D, E, and F) followed the same procedure as previously reported.^72,73^ The synthesized compounds **7–15** (Group B) were obtained through a straightforward two- to five-step process, followed by a single purification step, as detailed below. For general procedures, including amidation, azide reduction, sulfation, and ion exchange, please refer to the methods outlined below and see Scheme S1 for the structures of the intermediates and products. NMR and mass spectrometry analyses confirmed the purity of all biologically tested compounds (>95%).

A. ***N-*** **Acylation**: To a stirred solution of tobramycin sulfate salt (100 mg) in anhydrous MeOH (3 mL) at 0 °C was added the appropriate anhydride or acyl chloride (5 equiv per NH_2_) and triethylamine (5 equiv per NH_2_). The reaction mixture was allowed to warm to room temperature and stirred for 24–48 h. The progress of the reaction was monitored by ESI mass spectrometry.
B. **Sulfation**: The resulting amine substrates from previous step were subjected to a sulfation reaction by dissolving them in anhydrous pyridine (3 mL). Sulfur trioxide trimethylamine complex (SO_3_.Et_3_N) (5 equiv per amine or hydroxy group), and Et_3_N (5 equiv per amine or hydroxy group) were then added. Sulfation reactions were performed with a microwave synthesizer (Manufacturer: Biotage, model: Initiator^+^, Serial No: 013596–27J) at 100 °C and 1 atm for 15 min. The reaction progress was monitored by ESI mass spectrometry (negative ion mode), and the reaction was purified using a Sephadex LH-20 column (Fisher Scientific).
C. **Ion exchange**: To a solution of the sulfated substrate in MeOH (3 mL) was added Amberlite IR120 Na+ resin (10x weight of substrate). The mixture was stirred at room temperature for 12 h, filtered, and concentrated *in vacuo* to afford the sodium salt product, which was purified using a Sephadex LH-20 column.
D. **Hydrogenolysis**: To a stirring solution of 2-naphthylmethyl-protected substrate **48** (See Scheme S1) in 1:1 MeOH/H_2_O (3 mL) was added 10% Pd/C (5 equiv per Nap group) and excess hydrogen gas (balloon) for 12 h. The reaction progress was monitored by ESI mass spectrometry. The reaction mixture was filtered with Celite (Sigma-Aldrich) and concentrated *in vacuo* to give the desired crude product as a white powder.

#### 4.1.4 Synthetic Procedures for Ligands

##### 4.1.4.1 2″,4′,4″,5,6″-Penta-*O*-sulfo-tobramycin (**7**)

Sulfation and global reduction of **47** (See Scheme S1) to produce **7** were achieved according to the outlined sulfation and hydrogenolysis procedures, respectively. Isolated yield: 51%. ^1^H NMR (600 MHz, D_2_O) δ 5.65 (d, *J =* 4.0 Hz, 1H), 5.63 (d, *J =* 3.1 Hz, 1H), 4.70 (dd, *J =* 11.0, 3.9 Hz, 2H), 4.56–4.47 (m, 5H), 4.44 (dd, *J =* 8.6, 4.2 Hz, 1H), 4.26–4.19 (m, 3H), 4.14 (t, *J =* 9.5 Hz, 1H), 4.06 (t, *J =* 10.5 Hz, 1H), 3.91–3.87 (m, 1H), 3.56–3.38 (m, 6H), 2.81 (dd, *J =* 56.8, 8.3 Hz, 1H), 2.56–2.50 (m, 1H), 2.44–2.35 (m, 2H), 1.75 (d, *J =* 12.5 Hz, 1H). ^13^C NMR (151 MHz, D_2_O) δ 95.01, 94.38, 79.43, 75.75, 75.10, 74.65, 74.27, 71.58, 70.07, 68.07, 66.29, 61.28, 58.95, 58.91, 56.04, 50.56, 30.69, 28.78. HRMS: *m*/*z* calc. for C_18_H_32_N_5_Na_5_O_24_S_5_ [M-5Na+3H]^2–^: 432.5137; found: 432.5142.

##### 4.1.4.2 1,2′,3,3″,6′-Penta-*N*-acetyl-2’’,4′,4’’,5,6’’-Penta-*O*-sulfo-tobramycin **(8)**

Tobramycin sulfate salt (100 mg) was dissolved in MeOH (3 mL), and the resulting solution was added to acetic anhydride (5 equiv per NH_2_ group), followed by triethylamine (5 equiv per NH_2_). The crude product was then dissolved in anhydrous pyridine (3.4 mL), after which sulfur trioxide–trimethylamine complex (SO_3_.Et_3_N) (5 equiv per OH group) and triethylamine (5 equiv per OH group) were added. Sulfation reactions were performed with a microwave synthesizer (Manufacturer: Biotage, model: Initiator^+^, Serial #: 013596–27J) at 100 °C and 1 atm for 15–30 min according to the general sulfation procedure above. The reaction progress was monitored by ESI mass spectrometry (negative mode) and loaded onto a Sephadex LH-20 column for purification. Isolated yield: 54%. ^1^H NMR (500 MHz, CD_3_OD) δ 5.46 (d, *J* = 3.4 Hz, 1H), 5.17 (d, *J* = 3.3 Hz, 1H), 4.67 (dd, *J* = 11.7, 2.1 Hz, 1H), 4.42–4.35 (m, 2H), 4.31 (t, *J* = 10.3 Hz, 1H), 4.22–4.14 (m, 2H), 4.08–3.97 (m, 3H), 3.89 (ddd, *J* = 12.2, 10.5, 4.3 Hz, 1H), 3.84–3.75 (m, 3H), 3.58 (dd, *J* = 10.6, 8.9 Hz, 1H), 3.48 (dd, *J* = 10.2, 9.1 Hz, 1H), 3.27–3.19 (m, 1H), 2.32 (dt, *J* = 11.7, 4.6 Hz, 1H), 2.15 (dt, *J* = 10.9, 3.3 Hz, 1H), 2.03 (s, 6H), 2.02 (s, 3H), 2.01 (s, 3H), 1.97 (s, 3H), 1.90 (q, *J* = 12.1 Hz, 1H), 1.39 (q, *J* = 12.6 Hz, 1H). ^13^C NMR (101 MHz, CD_3_OD) δ 174.35, 174.05, 173.71, 173.15, 98.91, 98.79, 85.91, 82.12, 77.68, 76.38, 75.69, 73.45, 71.80, 70.68, 68.94, 51.67, 50.04, 49.78, 49.64, 49.50, 49.43, 49.21, 49.00, 48.79, 48.58, 48.36, 41.38, 34.65, 31.76, 23.39, 23.21, 22.99, 22.85. HRMS: *m*/*z* calc. for C_28_H_42_N_5_Na_5_O_29_S_5_ [M-5Na+2H]^2–^: 537.4827; found: 537.5402.

##### 4.1.4.3 1,2′,3,3″,6′-Penta-*N*-acetyl-2″,4′,4″,5-tetra-*O*-sulfo-6″-*O*-acetyl-tobramycin **(9)**

Compound **9** was synthesized from compound **48** using the general hydrogenolysis procedure, followed by N-acetylation by using acetic anhydride and triethylamine (5 equiv per NH_2_) in methanol. Crude product was then subjected to the general sulfation procedure and subsequent ion exchange to replace the triethylamine complex with sodium ions. Isolated yield: 69%. ^1^H NMR (600 MHz, D_2_O) δ 5.52 (d, *J* = 3.3 Hz, 1H), 5.28 (d, *J* = 3.4 Hz, 1H), 4.56 (t, *J* = 7.4 Hz, 1H), 4.45–4.38 (m, 3H), 4.36 (dd, *J* = 6.9, 2.9 Hz, 2H), 4.34–4.28 (m, 2H), 4.18 (tdd, *J* = 11.8, 8.8, 5.3 Hz, 2H), 4.10 (dt, *J* = 12.7, 4.0 Hz, 1H), 4.05 (dd, *J* = 9.2, 7.3 Hz, 1H), 3.88–3.79 (m, 2H), 3.64 (dd, J = 14.4, 3.3 Hz, 1H), 3.39 (dd, *J* = 14.4, 6.1 Hz, 1H), 2.34 (dt, *J* = 11.8, 4.7 Hz, 1H), 2.15 (s, 3H), 2.15–2.11 (m, 1H), 2.06 (s, 3H), 2.04 (s, 6H), 2.02 (d, *J* = 4.8 Hz, 6H), 1.90 (q, *J* = 12.0 Hz, 1H), 1.59 (dt, *J* = 13.4, 11.7 Hz, 1H). ^13^C NMR (151 MHz, D_2_O) δ 174.32, 174.12, 174.01, 173.57, 173.51, 95.84, 94.25, 79.94, 75.88, 72.14, 68.58, 68.46, 62.93, 48.52, 47.72, 47.14, 39.69, 31.80, 29.54, 22.43, 22.38, 22.22, 22.05, 21.88, 20.21. HRMS: *m*/*z* calc. for C_30_H_45_N_5_Na_5_O_27_S_4_ [M-4Na+3H]^1–^:1038.1418; found: 1038.1426.

##### 4.1.4.4 1,2′,3,3″,6′-Penta-*N*-benzyl-2″,4′,4″,5,6″-penta-*O*-sulfo-tobramycin (**10**)

Benzoic anhydride (0.5 mL, 2.06 mmol) was added to a solution of tobramycin sulfate (100 mg, 0.214 mmol) in methanol (4.3 mL) at 0 °C. triethylamine (0.3 mL, 2.14 mmol) was added, and the reaction mixture was stirred at room temperature for 12 h. The pH of the reaction mixture was monitored, and additional triethylamine was added as necessary to maintain slightly basic conditions. After 12 h, the reaction progress was assessed ESI mass spectrometry (negative mode). Upon completion, the mixture was concentrated *in vacuo*. The resulting crude product was then subjected to sulfation and subsequent sodium ion exchange as previously described. Isolated yield: 45%. ^1^H NMR (600 MHz, D_2_O) δ 7.53–7.49 (m, 1H), 7.43–7.39 (m, 1H), 7.36 (dt, *J =* 8.4, 1.5 Hz, 4H), 7.29 (td, *J =* 7.2, 1.5 Hz, 4H), 7.22 (dd, *J =* 8.3, 1.3 Hz, 2H), 7.19–7.14 (m, 2H), 7.13–7.01 (m, 7H), 6.93 (ddt, *J =* 10.3, 6.7, 3.9 Hz, 5H), 5.02 (d, *J =* 2.5 Hz, 2H), 4.09 (d, *J =* 55.0 Hz, 1H), 3.97–3.89 (m, 5H), 3.87 (ddd, *J =* 10.5, 6.9, 3.7 Hz, 1H), 3.85–3.80 (m, 1H), 3.80–3.76 (m, 1H), 3.59–3.51 (m, 2H), 3.45–3.39 (m, 2H), 3.39–3.33 (m, 1H), 2.88 (dd, *J =* 14.3, 4.3 Hz, 1H), 2.77 (dd, *J =* 14.3, 4.4 Hz, 1H), 2.01 (dt, *J =* 11.9, 4.6 Hz, 1H), 1.81 (dt, *J =* 13.1, 4.4 Hz, 1H), 1.67 (q, *J =* 11.9 Hz, 1H), 1.24 (q, *J =* 12.7 Hz, 1H). ^13^C NMR (126 MHz, D_2_O) δ 164.7, 164.4, 164.2, 164.1, 164.0, 137.8, 132.7, 127.7, 127.1, 127.0, 126.9, 126.7, 125.9, 125.8, 125.4, 125.3, 122.7, 122.3, 122.3, 122.3, 122.1, 122.0, 121.0, 120.8, 120.8, 120.7, 118.9, 90.1, 89.9, 75.6, 74.0, 69.9, 66.3, 63.2, 62.4, 60.4, 42.8, 42.2, 41.7, 33.5, 26.0, 23.4. HRMS: *m*/*z* calc. for C_53_H_52_N_5_Na_5_O_29_S_5_ [M-4Na]^2–^: 691.6283; found: 691.6281.

##### 4.1.4.5 1,2′,3,3″,6′-Penta-*N*-benzyl-6″-*O*-acetyl-2″,4′,4″,5-tetra-*O*-sulfo-tobramycin (**11**)

Compound **11** was synthesized from compound **48** (Scheme S1) using the general hydrogenolysis procedure, followed by N-acetylation by using benzoic anhydride and triethylamine (5 equiv per NH_2_) in methanol (1 mL). Crude product was then subjected to the general sulfation procedure and subsequent ion exchange to replace the triethylamine complex with sodium ions. Isolated yield: 30%. ^1^H NMR (500 MHz, D_2_O) δ 7.85 (d, *J =* 7.7 Hz, 4H), 7.78 (t, *J =* 7.9 Hz, 4H), 7.74–7.65 (m, 4H), 7.56 (dq, *J =* 23.2, 7.9 Hz, 8H), 7.43 (d, *J =* 7.2 Hz, 5H), 5.53 (t, *J =* 4.0 Hz, 2H), 4.68–4.49 (m, 3H), 4.48–4.23 (m, 7H), 4.08–3.99 (m, 2H), 3.91 (dd, *J =* 13.2, 9.6 Hz, 3H), 3.41–3.22 (m, 2H), 2.55–2.45 (m, 1H), 2.36 (d, *J =* 13.3 Hz, 1H), 2.16 (q, *J =* 11.9 Hz, 1H), 1.96 (d, *J =* 1.0 Hz, 3H), 1.76 (q, *J =* 12.6 Hz, 1H). ^13^C NMR (126 MHz, D_2_O): δ 173.9, 173.7, 173.5, 173.4, 172.5, 172.4, 172.3, 169.4, 137.4, 136.8, 136.7, 136.1, 134.8, 134.8, 134.8, 134.7, 134.6, 134.6, 134.4, 133.7, 132.5, 130.0, 129.7, 129.2, 129.2, 129.2, 129.2, 129.1, 129.1, 129.0, 128.9, 128.8, 128.8, 128.7, 128.0, 127.8, 127.8, 127.7, 127.7, 127.7, 127.3, 127.3, 127.3, 127.2, 127.2, 127.1, 127.1, 127.1, 98.8, 96.6, 87.1, 81.8, 78.2, 76.5, 75.5, 75.4, 73.9, 71.8, 71.6, 70.6, 69.2, 64.6, 55.9, 51.7, 47.9, 45.6, 43.8, 41.5, 40.2, 34.6, 31.7, 30.2, 25.0, 24.1, 23.5, 23.2, 23.0, 22.9, 22.8, 20.5, 14.5, 13.3, 11.5, 9.3. HRMS: *m*/*z* calc. for C_53_H_52_N_5_Na_5_O_29_S_5_ [M-5Na+3H]^2–^: 663.6166; found: 663.6117.

##### 4.1.4.6 1,2′,3,3″,6′-Penta-*N*-tertbutyloxycarbonyl-2″,4′,4″,5,6″-penta-*O*-sulfo-tobramycin (**12**)

To a solution of tobramycin (0.025 g, 0.053 mmol) in H_2_O (1 mL) at 0 °C was added sodium bicarbonate (NaHCO_3_), (0.036 g, 0.428 mmol). After the NaHCO_3_ had completely dissolved, THF (0.7 mL) was added, followed by di-*tert*-butyl dicarbonate (0.093 g, 0.428 mmol). The resulting mixture was stirred at room temperature for 12 h. ESI mass spectrometry (negative mode) monitored the reaction progress, and the mixture was concentrated *in vacuo* after completion. The resulting crude product was then subjected to sulfation and subsequent sodium ion exchange as previously described. Isolated yield: 63%. ^1^H NMR (600 MHz, D_2_O) δ 4.99 (d, *J =* 16.2 Hz, 2H), 3.87 (d, *J =* 9.9 Hz, 1H), 3.68 (d, *J =* 11.7 Hz, 1H), 3.61 (t, *J =* 11.2 Hz, 2H), 3.52 (d, *J =* 8.6 Hz, 2H), 3.45 (s, 1H), 3.36 (dq, *J =* 31.9, 10.5 Hz, 6H), 3.21 (s, 2H), 2.90 (s, 1H), 2.77 (s, 1H), 1.99 (s, 2H), 1.79 (s, 2H), 1.55 (q, *J =* 12.0 Hz, 1H), 1.35 (d, *J =* 11.3 Hz, 45H), 1.23 (dd, *J =* 13.9, 8.3 Hz, 1H). ^13^C NMR (126 MHz, D_2_O): δ 158.2, 157.5, 157.5, 157.4, 97.4, 95.6, 81.8, 81.6, 81.3, 81.0, 76.0, 74.5, 72.6, 69.5, 69.0, 67.2, 51.6, 49.1, 48.6, 40.9, 30.3, 27.8, 27.8, 27.8, 27.8, 8.4. HRMS: *m*/*z* calc. for C_43_H_72_N_5_Na_5_O_34_S_5_ [M-4Na]^2–^: 693.2260; found: 693.2285.

##### 4.1.4.7 1,2′,3,3″,6′-Penta-N-tosyl-2″,4′,4″,5,6″-penta-*O*-sulfo-tobramycin (**13**)

*p*-Toluenesulfonic anhydride (0.5 mL, 2.14 mmol) was added to a solution of tobramycin sulfate (100 mg, 0.214 mmol) in methanol (4.3 mL) at 0 °C. Triethylamine (0.3 mL, 2.14 mmol) was added, and the reaction mixture was stirred at room temperature for 12 h. The pH of the reaction mixture was monitored, and additional triethylamine was added as necessary to maintain slightly basic conditions. After 12 h, the progress of the reaction was assessed by ESI mass spectrometry (negative ion mode). Upon completion, the mixture was concentrated *in vacuo*. The resulting crude product was then sulfated and subsequently sodium-exchanged as previously described. Isolated yield: 52%. ^1^H NMR (500 MHz, D_2_O) δ 7.86 (d, *J =* 8.1 Hz, 2H), 7.77 (dd, *J =* 18.5, 8.1 Hz, 4H), 7.65–7.57 (m, 8H), 7.39 (dd, *J =* 26.4, 8.1 Hz, 4H), 7.27 (d, *J =* 8.2 Hz, 2H), 5.06 (d, *J =* 3.8 Hz, 1H), 4.90 (d, *J =* 3.3 Hz, 1H), 4.46 (dd, *J =* 11.1, 2.2 Hz, 1H), 4.19–4.10 (m, 2H), 4.09–4.02 (m, 2H), 3.88 (td, *J =* 7.8, 2.9 Hz, 1H), 3.74 (dd, *J =* 10.3, 3.7 Hz, 1H), 3.59 (t, *J =* 10.1 Hz, 1H), 3.38–3.20 (m, 5H), 3.08 (dd, *J =* 14.6, 7.3 Hz, 1H), 2.62–2.57 (m, 1H), 2.55 (s, 3H), 2.52 (s, 3H), 2.46–2.41 (m, 1H), 2.39 (d, *J =* 4.7 Hz, 6H), 2.37 (d, *J =* 8.9 Hz, 1H), 2.30 (s, 3H), 2.06 (dt, *J =* 12.6, 4.5 Hz, 1H), 1.85 (q, *J =* 10.7 Hz, 1H), 1.77–1.73 (m, 1H), 1.36–1.22 (m, 2H). ^13^C NMR (126 MHz, D_2_O): δ 144.9, 144.7, 144.4, 144.3, 144.2, 137.1, 136.8, 136.8, 136.0, 134.5, 130.3, 129.9, 129.7, 129.6, 127.2, 127.1, 126.9, 126.7, 100.6, 94.6, 85.4, 74.1, 73.1, 71.9, 70.8, 70.6, 69.9, 67.2, 57.4, 52.7, 52.0, 51.1, 42.9, 31.6, 30.8, 21.1, 21.1, 20.9, 20.9, 20.8.

##### 4.1.4.8 1,2′,3,3″,6′-Penta-*N*-trichloroacetyl-2″,4′,4″,5,6″-penta-*O*-sulfo-tobramycin (**14**)

Trichloroacetic anhydride (0.4 mL, 2.14 mmol) was added to a solution of tobramycin sulfate (100 mg, 0.214 mmol) in methanol (4.3 mL) at 0 °C. Triethylamine (0.3 mL, 2.14 mmol) was added, and the reaction mixture was stirred at room temperature for 12 h. The pH of the reaction mixture was monitored, and additional triethylamine was added as necessary to maintain slightly basic conditions. After 12 h, the reaction progress was assessed by ESI mass spectrometry (negative mode). Upon completion, the mixture was concentrated *in vacuo*. The resulting crude product was then subjected to sulfation and subsequent sodium ion exchange as previously described. Isolated yield: 78%. ^1^H NMR (500 MHz, D_2_O) δ 5.62 (d, *J =* 3.4 Hz, 1H), 5.50 (d, *J =* 2.7 Hz, 1H), 4.60 (dd, *J =* 10.8, 3.5 Hz, 1H), 4.56–4.45 (m, 5H), 4.40–4.31 (m, 2H), 4.28–4.23 (m, 1H), 4.15 (ddd, *J =* 12.3, 10.2, 4.2 Hz, 1H), 4.06–3.94 (m, 3H), 3.88 (dd, *J =* 10.3, 9.0 Hz, 1H), 3.80–3.69 (m, 2H), 2.48 (dt, *J =* 13.7, 4.3 Hz, 1H), 2.37 (dt, *J =* 13.1, 4.2 Hz, 1H), 2.25 (dt, *J =* 13.7, 7.9 Hz, 1H), 1.72 (q, *J =* 12.5 Hz, 1H). ^13^C NMR (126 MHz, D_2_O): δ 164.4, 164.4, 164.1, 164.1, 163.8, 129.3, 125.4, 95.4, 94.9, 91.7, 91.7, 91.6, 91.5, 91.4, 79.4, 78.5, 75.4, 74.0, 73.6, 72.0, 69.3, 67.0, 52.2, 51.1, 50.6, 48.5, 40.4, 30.2, 28.4. HRMS: *m*/*z* calc. for C_28_H_27_Cl_15_N_5_Na_5_O_29_S_5_ [M-5Na+3H]^2–^: 767.2627; found: 767.2615.

##### 4.1.4.9 1,2′,3,3″,6′-Penta-*N*-chloroacetyl-2″,4′,4″,5,6″-penta-*O*-sulfo-tobramycin (**15**)

A solution of tobramycin sulfate in MeOH (2 mL) was added to chloroacetic anhydride (5 equiv per NH_2_ group), followed by triethylamine (5 equiv per NH_2_). The crude product was dissolved in anhydrous pyridine (3.4 mL). Sulfur trioxide trimethylamine complex (SO_3_.Et_3_N) (5 equiv per OH) and triethylamine (5 equiv per OH) were then added. Sulfation reactions were performed with a microwave synthesizer (Manufacturer: Biotage, model: Initiator^+^, Serial #: 013596–27J) at 100 °C and 1 atm for 15–30 min according to the general sulfation procedure above (see Scheme S2). The reaction progress was monitored by ESI mass spectrometry (negative mode) and loaded onto a Sephadex LH-20 column for purification. Isolated yield: 47%. ^1^H NMR (600 MHz, D_2_O) δ 5.24 (d, *J =* 3.5 Hz, 1H), 5.03 (d, *J =* 3.8 Hz, 1H), 4.09–4.06 (m, 6H), 4.02 (s, 3H), 3.99 (q, *J =* 4.9 Hz, 4H), 3.94 (q, *J =* 9.6 Hz, 6H), 3.72–3.63 (m, 4H), 3.59 (td, *J =* 9.7, 4.1 Hz, 3H), 3.54–3.44 (m, 6H), 3.44–3.39 (m, 2H), 1.91 (ddt, *J =* 17.8, 13.2, 4.4 Hz, 2H), 1.59 (dq, *J =* 18.9, 12.5 Hz, 2H). ^13^C NMR (126 MHz, D_2_O): δ 174.8, 174.5, 174.3, 173.7, 173.6, 96.4, 95.5, 82.5, 78.5, 76.3, 72.1, 69.3, 68.5, 67.1, 48.7, 48.1, 47.3, 39.7, 32.5, 29.7, 22.4, 22.4, 22.4, 22.1, 22.0. HRMS: *m*/*z* calc. for C_28_H_27_Cl_15_N_5_Na_5_O_29_S_5_ [M-5Na]^2–^: 622.9402; found: 622.9400.

##### 4.1.4.10 Tetrasaccharide (**49**)

Compound **49** was synthesized by following the literature procedure^62^ with *p*-methoxyphenol linker. ^1^H NMR (600 MHz, D_2_O) δ 7.04 (d, *J =* 9.1 Hz, 2H), 6.88 (d, *J =* 9.2 Hz, 2H), 5.61 (d, *J =* 2.8 Hz, 1H), 5.28 (d, *J =* 3.6 Hz, 1H), 5.23 (d, *J =* 3.7 Hz, 1H), 5.14 (d, *J =* 2.4 Hz, 1H), 4.96–4.90 (m, 1H), 4.39 (dd, *J =* 5.0, 2.7 Hz, 1H), 4.30–4.05 (m, 8H), 4.03 (t, *J =* 3.2 Hz, 1H), 3.98–3.93 (m, 1H), 3.71 (d, *J =* 3.1 Hz, 1H), 3.70 (s, 3H), 3.61–3.55 (m, 2H), 3.49–3.39 (m, 2H), 3.21–3.10 (m, 2H). ^13^C NMR (101 MHz, D_2_O) δ 171.65, 171.31, 154.73, 150.18, 119.52, 115.00, 101.73, 99.04, 97.07, 96.59, 77.44, 75.92, 75.76, 75.44, 74.30, 70.81, 70.51, 69.53, 69.21, 68.04, 67.38, 66.79, 57.47, 55.73, 52.98.

### 4.2 Statistical Analysis

#### 4.2.1 Response

All experiments were conducted at least three times independently unless otherwise stated. Statistical analyses were performed using GraphPad Prism (version 10.2.3) while the Octet RED96 system (fortéBio) was used for the BLI assay. IC_50_ was obtained using the GraphPad Prism software. Data are presented as mean ± SEM from three independent experiments. Statistical significance was defined as *p < 0.05, **p < 0.01, ***p < 0.001 and ****p < 0.0001, with *p < 0.05 considered statistically significant unless otherwise specified.

### 4.3 Biolayer Interferometry (BLI) Assay

Recombinant human FGF2 (Catalog No. 232-FA-025/CF), recombinant human VEGF_165_ (Catalog No. 293-VE), and recombinant human FGF4 (Catalog No. 235-F4–025/CF) were obtained from R&D Systems. Biotinylated FGF2 (Catalog No. FGC-H81E3) and the extracellular domain of FGFR1 (Catalog No. NM-023110) were purchased from ACROBiosystems. For the direct binding assays, recombinant FGF2 and FGFR1 were biotinylated using the Thermo Scientific EZ-Link Sulfo-NHS-LC-Biotinylation Kit (Catalog No. 21955) according to the manufacturer’s instructions. Biotinylated heparin was obtained from Creative PEGWorks (Catalog No. HP-207). Ligands (aminoglycoside-based heparan sulfate mimetics) were synthesized in-house and prepared at varying concentrations. Streptavidin biosensors were purchased from Sartorius (Catalog No. 18–5019). The 1× HBS-EP assay buffer (10 mM HEPES, pH 7.4, 150 mM NaCl, 3.0 mM EDTA, and 0.005% Tween-20) was prepared by diluting a 10× HBS-EP+ stock solution obtained from Cytiva (Catalog No. BR100829). The assay was conducted using the Octet Red Instrument (Octet RED96) from FortéBio. Streptavidin biosensors were preincubated in 1X HBS-EP assay buffer for at least 15 min before use. The detailed assay procedures are described below. The BLI durations for each step are outlined in the Supporting Information.

### 4.4 FGF2 Binding Assay (BLI Kinetic Binding Assay)

For each competition assay, a 70 nM FGF2 (16.5 kDa) working solution (35 nM final concentration) was used. A 1 mg/mL stock solution of biotinylated heparin (18 kDa) was diluted to a final concentration of 5 μg/mL in the assay buffer. Ligands were prepared in a series of dilutions, ranging from 100 μM to 0.3 μM. The binding kinetics were analyzed using the Octet RED96 software to determine the competition efficiency by comparing the binding responses of FGF2 in the presence and absence of the ligands. The percentage activity of each concentration, indicating the competitive binding affinity, was then calculated. This percentage response was used to determine the half-maximal inhibitory concentration (IC_50_) values for each ligand using GraphPad Prism software. The assay was repeated three times to ensure the results were reliable.

### 4.5 FGFR1 Binding Assay (BLI Kinetic Binding Assay)

The FGFR1 competitive binding assay for the aminoglycoside HS mimetics and biotinylated heparin followed the same procedure as the FGF2 binding assay. The final working concentration of FGFR1 (74 kDa) was set at 10 μg/mL, as established by Opalinski et al.^80^ All other assay parameters were kept constant.

### 4.6 FGFR1-FGF2 Binding Assay (BLI Kinetic Binding Assay)

The following procedure was used to assess the interference of compound **8** with FGFR1-FGF2 binding. The final working concentration of FGFR1 was 10 μg/mL, and biotinylated human FGF2 (20.1 kDa) was 31.1 nM. This activation or inhibition potential was compared to that of heparin. Briefly, six dilution series of compound **8** or heparin (ranging from 2500 to 0.078 nM, 100 μM) were prepared and premixed with FGFR1 (10 μg/mL, 100 μL). Biotinylated FGF2 (31.1 nM) was immobilized on the streptavidin biosensor, achieving a response unit of about 3.5 nm. The K_D_ of each ligand was obtained using the resulting R-equilibrium, as reported by Selim et al.^81^

### 4.7 VEGF_165_ Binding Assay (BLI Kinetic Binding Assay)

The following procedure was used to assess the interference of compounds with recombinant human VEGF_165_ (19.2 kDa). An initial concentration of 50 nM and a final working concentration of 25 nM was used. The optimum binding concentration of biotinylated heparin (27 kDa) was set at 1 μg/mL. Briefly, six dilution series of compound **8** (2000 to 6 nM), **35** and **37** (100 to 3 μM) or heparin (ranging from 100 to 0.078 nM) were prepared and premixed with VEGF_165_ (50 nM, 100 μL). Biotinylated heparin (1 μg/mL) was immobilized on the streptavidin biosensor. The percentage response was used to determine the half-maximal inhibitory concentration (IC_50_) values for each ligand using GraphPad Prism.

### 4.8 FGF4 Binding Assay (BLI Kinetic Binding Assay)

The following procedure was used to assess the interference of compounds with recombinant human FGF4 (17 kDa). An initial protein concentration of 70 nM and a final working concentration of 35 nM was used. The optimum binding concentration of biotinylated heparin (18 kDa) was set at 5 μg/mL. Briefly, six dilution series of ligands **8** (250 to 7.81 μM), **49** (500 to 15.6 μM) and **50** (100 to 3.1 μM) were prepared and premixed with VEGF_165_ (70 nM, 100 μL). Biotinylated heparin (5 μg/mL) was immobilized on the streptavidin biosensor. The percentage response was used to determine the half-maximal inhibitory concentration (IC_50_) values for each ligand using GraphPad Prism.

### 4.9 FGF2 and FGFR1 Direct Binding Assays

The following procedure was used to assess the direct binding of compounds to human FGF2 and FGFR1 using biolayer interferometry. Biotinylated FGF2 or FGFR1 (10 nM) was immobilized on streptavidin biosensors. A seven-point dilution series of each ligand was prepared over a concentration range of 5 to 0.078 μM. Following baseline equilibration, compound solutions were associated with the immobilized protein, then dissociated in assay buffer. Binding responses were recorded as wavelength shifts and analyzed using GraphPad Prism. Equilibrium binding responses were plotted as a function of ligand concentration and fit to a one-site binding model to determine the apparent dissociation constant (*K*_d_) values for each ligand.

## 5 Computational Docking and Molecular Dynamics (MD) Simulation

Molecular docking and molecular dynamics (MD) simulations were performed using YASARA Structure version 22.9.24, and job runs or calculations were performed using the Wayne State High Performance Computing (HPC, grid.wayne.edu). The docking simulations were carried out using the AutoDock Vina algorithm embedded in YASARA, with subsequent refinement of docking scores performed under explicit-solvent conditions using MD simulations. The PDB files of the ligands were generated using ChemDraw 3D, GaussView, or Avogadro, and then optimized in YASARA. The PDBs of the proteins FGF2 (4OEF), FGFR1 (1FQ9), and VEGF165 (2VGH) were optimized by removing the crystallographic water molecules and ions and adjusting protonation states. For FGFR1 (PDB code: 1FQ9), both FGF2 proteins, heparin polysaccharide, and one FGFR1 unit were removed, leaving a single FGFR1 unit for docking and MD simulation. Energy minimization was then carried out on the protein and ligands using the AMBER14 force field in YASARA before subjecting to docking. Each ligand was individually docked into the protein or enzyme within a defined simulation cell (dimensions 80 × 61 × 76 Å) using AutoDock Vina with default settings. A total of 25 docking runs were performed for each ligand-enzyme complex, applying the AMBER14 force field to the protein and the GLYCAM06 and GAFF/Am1BCC force fields to the ligands. The active site residues were kept flexible during docking. The most populated docking clusters were selected for molecular dynamics (MD) simulations. Molecular dynamics (MD) simulations were performed on the most populous protein or receptor–ligand complexes. The docked complexes were imported into YASARA, where the simulation cell was filled with TIP3P water molecules. A periodic boundary was applied using the AMBER14 force field. The simulations run for 100 ns under physiological conditions (pH 7.4), with sodium and chloride ions added as counterions to maintain neutrality. All docked and molecular dynamics electrostatic structures were generated using ChimeraX.

## Supplementary Material







## Notes

Molecular formula strings (CSV)
PDB models of docked structures (ZIP)
Molecular docking, molecular dynamics, synthetic schemes, biological assays, NMR, HRMS, and HPLC spectra data (PDF)

## Abbreviations Used

Biphenyl: 4-biarylmethyl
BLI: biolayer interferometry
(COCl)_2_: oxalyl chloride
DCM: dichloromethane
DMAP: 4-dimethylaminopyridine
DMF: N,N-dimethylformamide
ECM: extracellular matrix
Et_3_N: trimethylamine
EtOAc: ethyl acetate
FGF2: fibroblast growth factor 2
FGF4: fibroblast growth factor 4
FGFR1: fibroblast growth factor receptor 1
GAG: glycosaminoglycan
HPLC: high-performance liquid chromatography
HS: heparan sulfate
HSPG: heparan sulfate proteoglycans
kDa: kilodalton
MeOH: methanol
NaH: sodium hydride
Nap: 2-naphthylmethyl
NapBr: naphthyl bromide
PhI­(Ac)_2_: diacetoxy iodobenzene
RMSD: root-mean-square deviation
SAR: structure–activity relationship
SO_3_Me_3_N: sulfur trioxide trimethylamine
TBAI: tetrabutylammonium iodide
TEMPO: 2,2,6,6-tetramethyl-1-piperidinyloxy
THF: tetrahydrofuran
VEGF_165_: vascular endothelial growth factor 165
