# Supplementary material for: Synthetic Heparan Sulfate-like Oligosaccharides as Tools to Decipher Growth Factor Recognition
Source: J Med Chem. 2026 Aug 20;69(17):20294–312. doi: 10.1021/acs.jmedchem.6c00760 (PMC13576445; doi:10.1021/acs.jmedchem.6c00760)
Supplement: Supplementary file 1 [file jm6c00760_si_003.pdf]

Supporting Information

Synthetic Heparan Sulfate-Like Oligosaccharides as Tools to Decipher Growth Factor Recognition

*Joseph Wakpal, Kartikey Singh, Michael Hotor, Livia Philip, Dylan Yost-Slinker, Junzhe Wang, Hawau Abdulsalam, Hien M. Nguyen**

Department of Chemistry, Wayne State University, Detroit, Michigan 48202, United States

*Email: hmnguyen@wayne.edu

Table of Contents **Pages**

1. General Synthesis of *N*-sulfated tobramycin-based HS mimetics..S2
2. Lead HS mimetics **8**, **35**, and **37** from tobramycin…………………………………………………...S3
3. Experimental methods of biological studies ………………………………………………..............S3
4. Biolayer Interferometry (BLI) Assay for FGF2, FGFR1, $VEGF_{165}$, and FGF4 ………….....................S5
5. NIH3T3 Cell Culture and Functional Proliferation Assays …………………....................................S26
6. Complete IC_{50} values and structures of all compounds……………………………………………S29
7. Computational studies……………………………………………………………………............S35
8. NMR Spectra of all new Compounds…………………………………………… ………………..S62
9. References……………………………………………………………………………………….S75

Caution:

(1) The azide formation step should be handled with caution, as organic azides are potentially explosive.
(2) Organic azides can and will decompose with the introduction of external energy (heat, light, or pressure).
(3) Organic azides synthesized should be stored at -20 °C in the dark
(4) Organic azide waste should be placed in a separate container designated only for azide waste.
(5) Make sure that organic azide waste should not come in contact with acid.

Scheme S1. Synthesis of *N*-acylated tobramycin-based HS mimetics

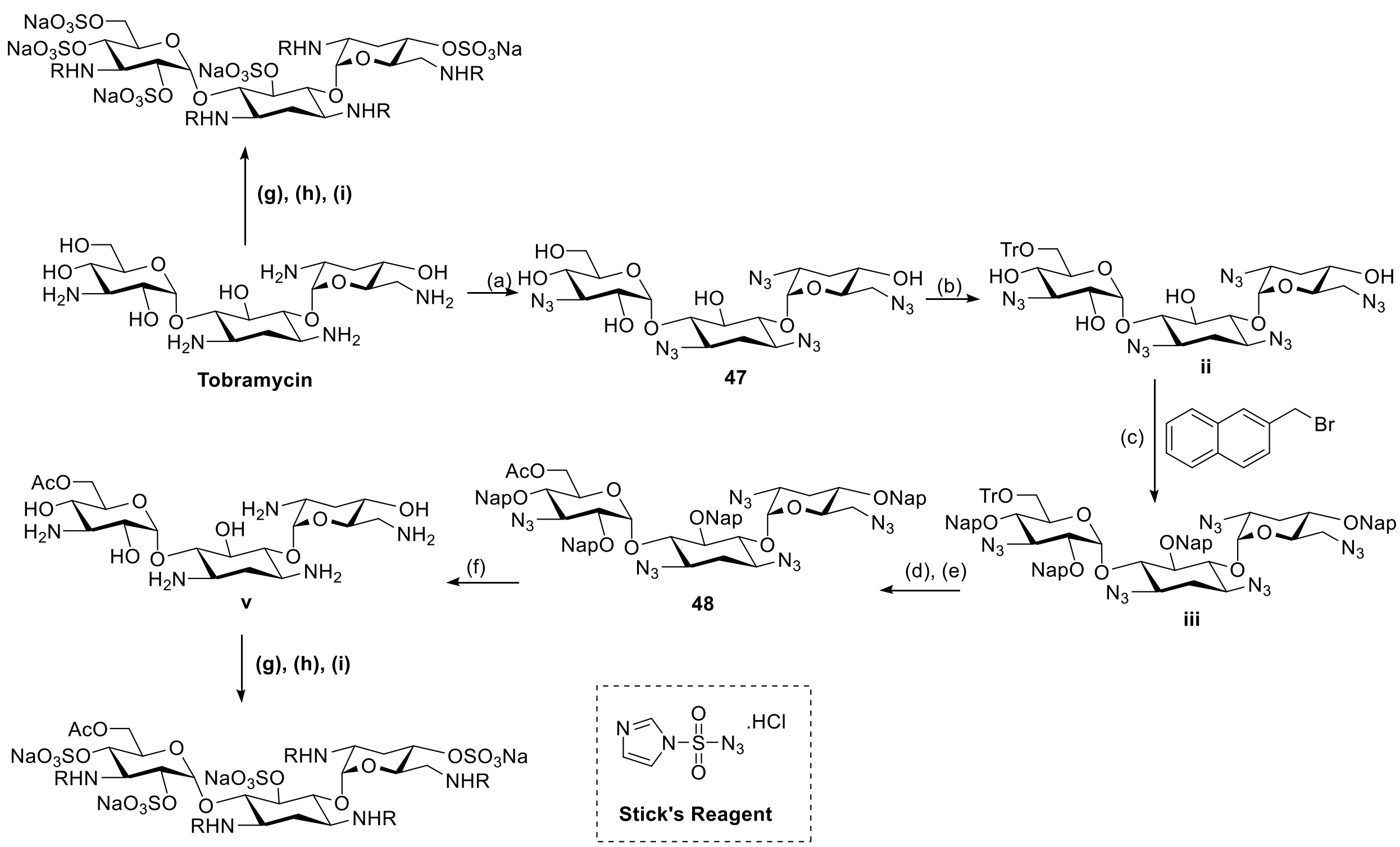

Reagents and Conditions: (a) **Stick's reagent**, $CuSO_4 \cdot 5H_2O$, K_2CO_3, H_2O, 0 °C - rt, 12 h, 65%; (b) TrCl, DMAP, Pyridine, 50 °C - rt, 12 h, 90%; (c) NapBr, NaH, TBAI, DMF, 0 °C - rt, 12 h, 79%, (d) TFA/DCM (2:3), 0 °C - rt, 2 h, 60%; (e) Acetic anhydride (3 equiv.), pyridine (5 equiv.), anhydrous DCM, 0ºC-rt 2 h. (f) 20% Pd/C, H_2, MeOH/H_2O (2:1), rt,12 h, 48 - 55%. (g) Acyl anhydride or tosyl chloride (5 equiv. per NH_2), TEA (5 equiv. per NH_2) MeOH, 0ºC-rt 24-48 h, (h) $SO_3 \cdot Me_3N$, Et_3N, pyridine, microwave, 100 °C 15 min; (i) IR120 Na^+ resin, MeOH, rt, 12 h, 45 - 72%;

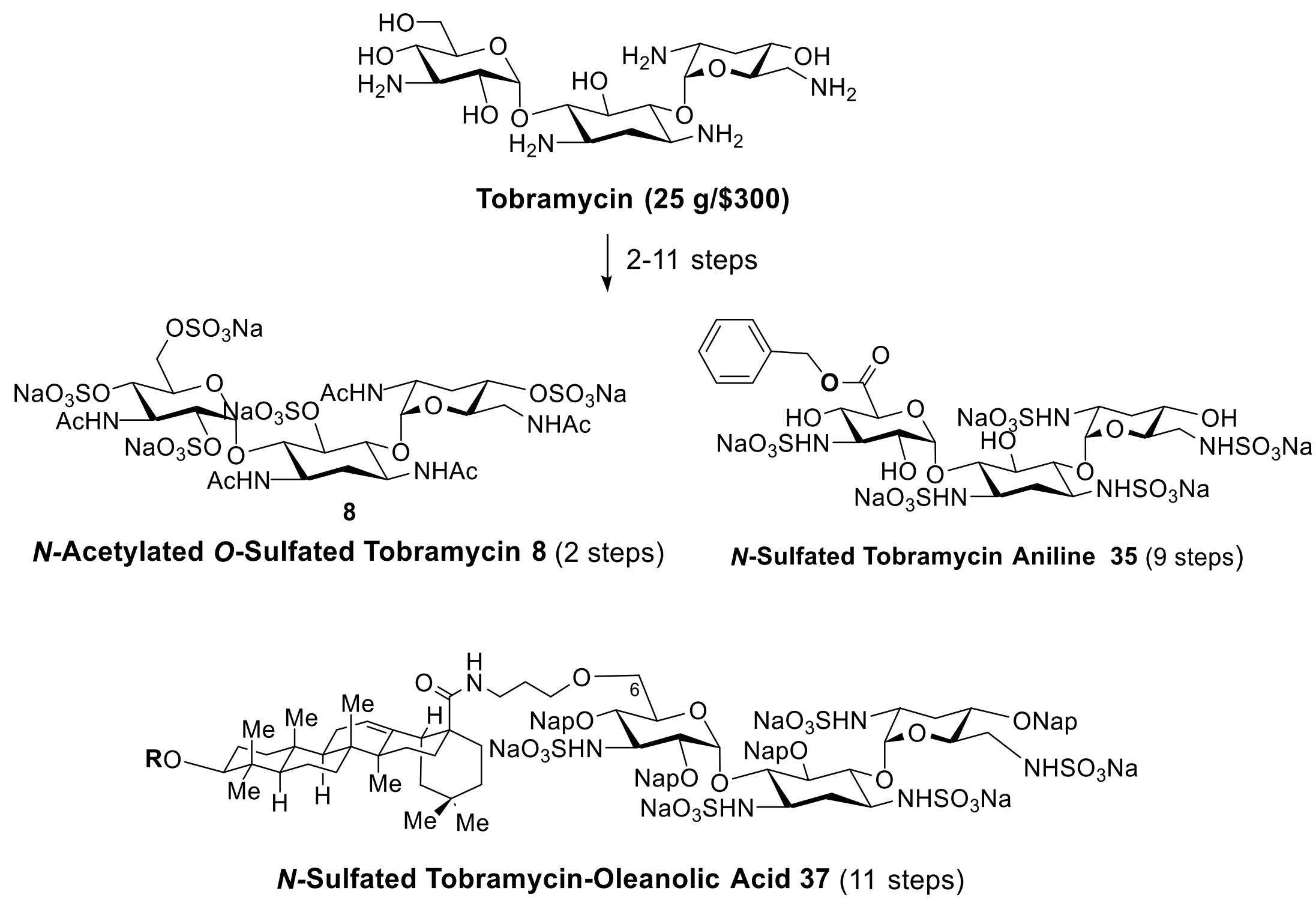

Figure S1. Heparan sulfate-like oligosaccharide mimetics from tobramycin[1]

Biological Studies: Binding Assay (BLI Kinetic Binding Assay): A total of 48 compounds were evaluated by biolayer interferometry (BLI) for determining the binding affinity (K_d) and inhibitory activity (IC_{50}).[2] All measurements were performed in triplicate (n = 3) under identical assay conditions. Kinetic and/or steady-state analyses were conducted using Octet Red Instrument (Octet RED96) at 25 °C, and nonlinear regression analyses for IC_{50} determination were performed using GraphPad Prism (GraphPad Software, Invoice No. 43320046). Representative sensorgrams and fitted curves are provided herein for selected compounds. The reported K_d and IC_{50} values for all compounds were derived from independently repeated experiments using the same fitting models and parameters.

FGF2 Binding Assay (BLI Kinetic Binding Assay)

<u>Assay Procedures</u>

Assay Step Number	Step Data Name	Sensor Column	Sample Column	Step Type	Assay Time
1	Baseline	1	**1**	Baseline	120
2	Loading	1	**2**	Loading	300

3	Baseline	1	**1**	Baseline	120
4	Association	1	**3**	Association	300
5	Dissociation	1	**1**	Dissociation	600

Plate Temp = 25 °C

$$\% \, activity = \frac{Response}{Response \ at \ 0 \ nM} \times 100\%$$

FGFR1 Binding Assay (BLI Kinetic Binding Assay)

<u>Assay Procedures</u>

Assay Step Number	Step Data Name	Sensor Column	Sample Column	Step Type	Assay Time
1	Baseline	1	**1**	Baseline	120
2	Loading	1	**2**	Loading	1000
3	Baseline	1	**1**	Baseline	120
4	Association	1	**3**	Association	500
5	Dissociation	1	**1**	Dissociation	1000

- Plate Temp = 25 °C

$VEGF_{165}$ Binding Assay (BLI Kinetic Binding Assay)

<u>Assay Procedures</u>

Assay Step Number	Step Data Name	Sensor Column	Sample Column	Step Type	Assay Time
1	Baseline	1	**1**	Baseline	120
2	Loading	1	**2**	Loading	300
3	Baseline	1	**1**	Baseline	120
4	Association	1	**3**	Association	300
5	Dissociation	1	**1**	Dissociation	600

- Plate Temp = 25 °C

FGF4 Binding Assay (BLI Kinetic Binding Assay)

<u>**Assay Procedures**</u>

Assay Step Number	Step Data Name	Sensor Column	Sample Column	Step Type	Assay Time
1	Baseline	1	**1**	Baseline	120
2	Loading	1	**2**	Loading	300
3	Baseline	1	**1**	Baseline	120
4	Association	1	**3**	Association	300
5	Dissociation	1	**1**	Dissociation	600

- Plate Temp = 25 °C

Biolayer interferometry assay (BLI) data of ligands in complex with FGF2 and FGFR1

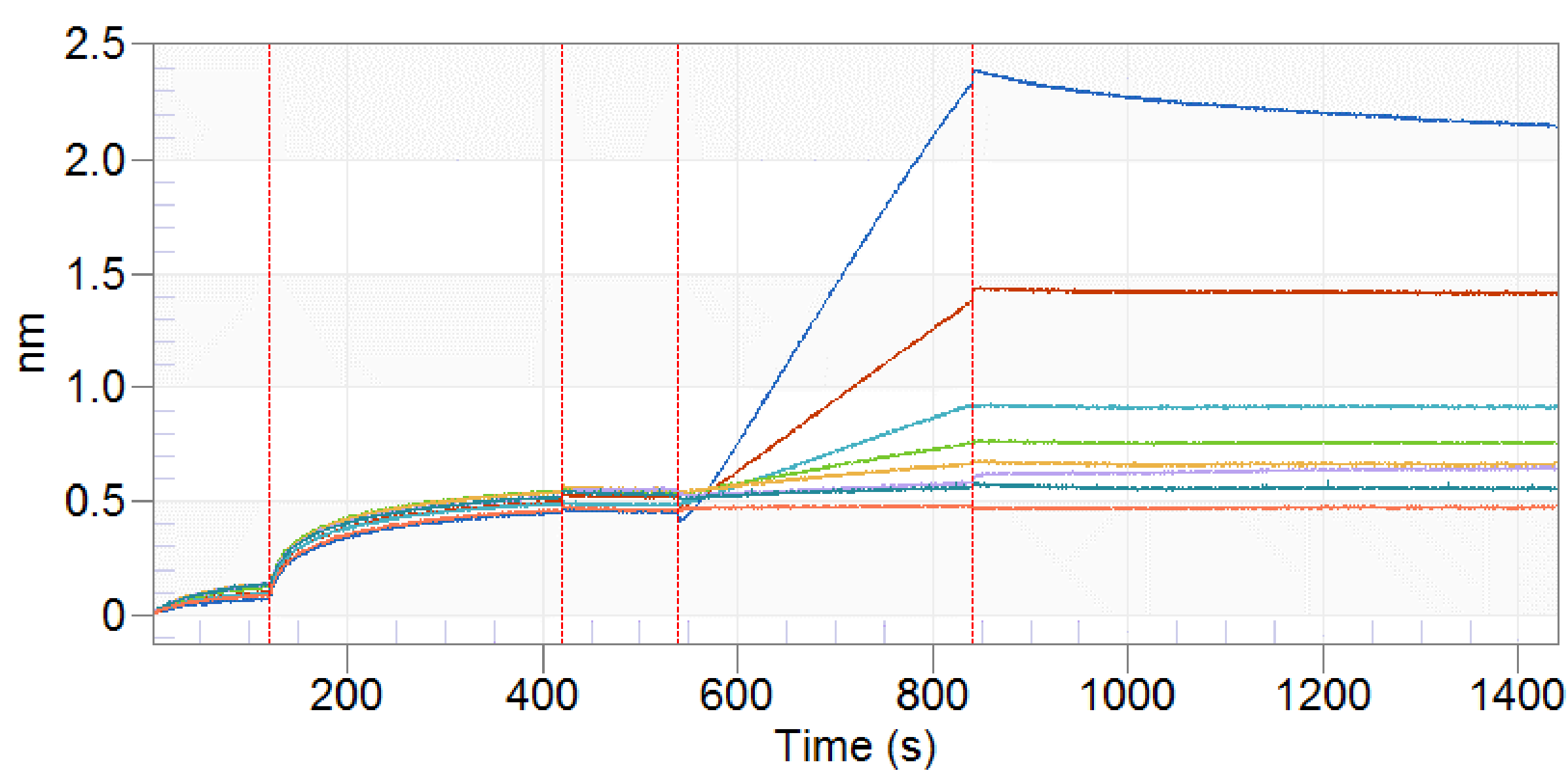

Figure S2. BLI sensorgram (raw data) of the interaction between biotinylated heparin (5 µg/mL) and a two-fold serial dilution of FGF2 (0.75-48 nM).

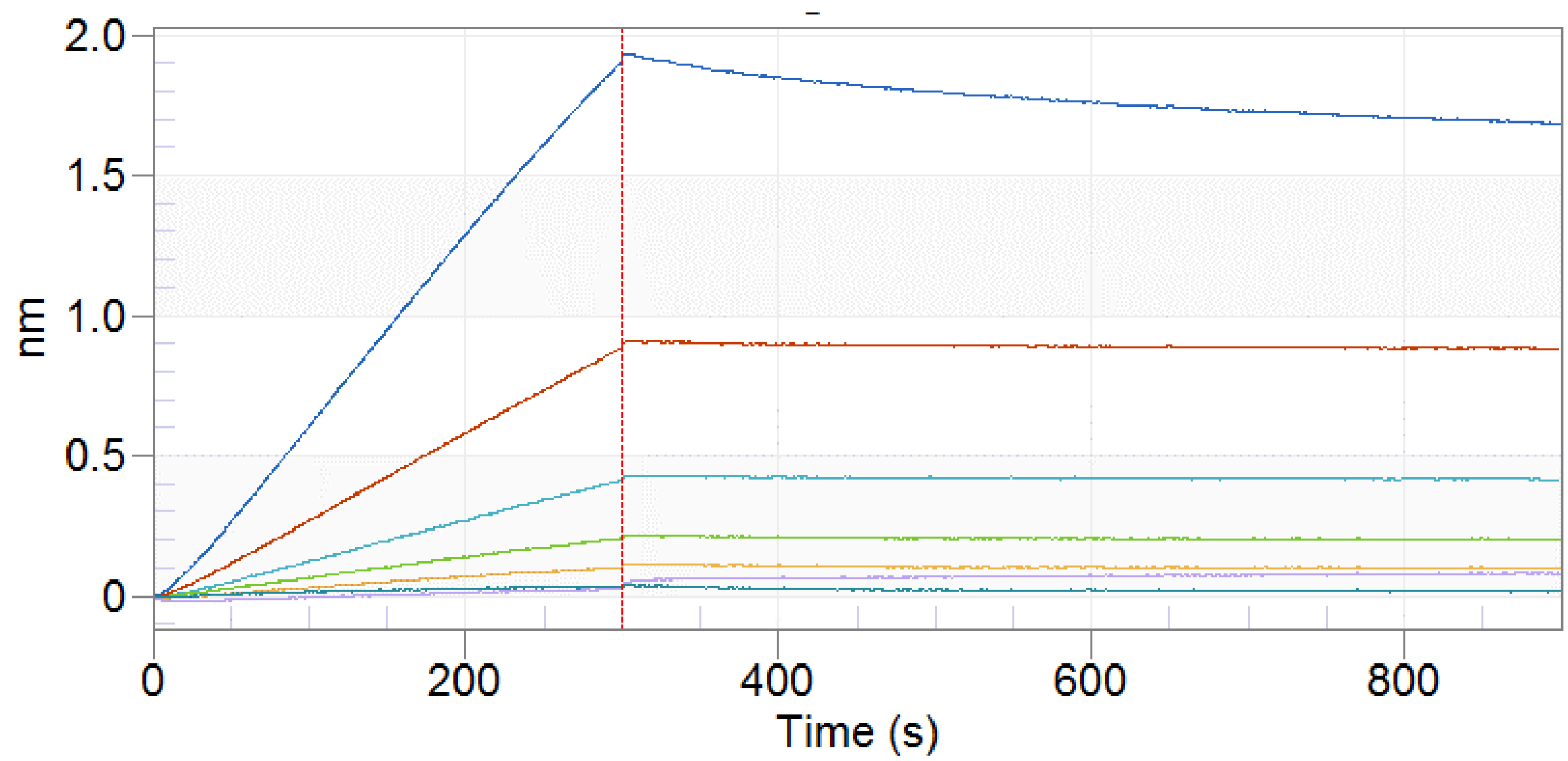

Figure S3. BLI sensorgram (processed data) of the interaction between biotinylated heparin (5 µg/mL) and a two-fold serial dilution of FGF2 (0.75-48 nM).

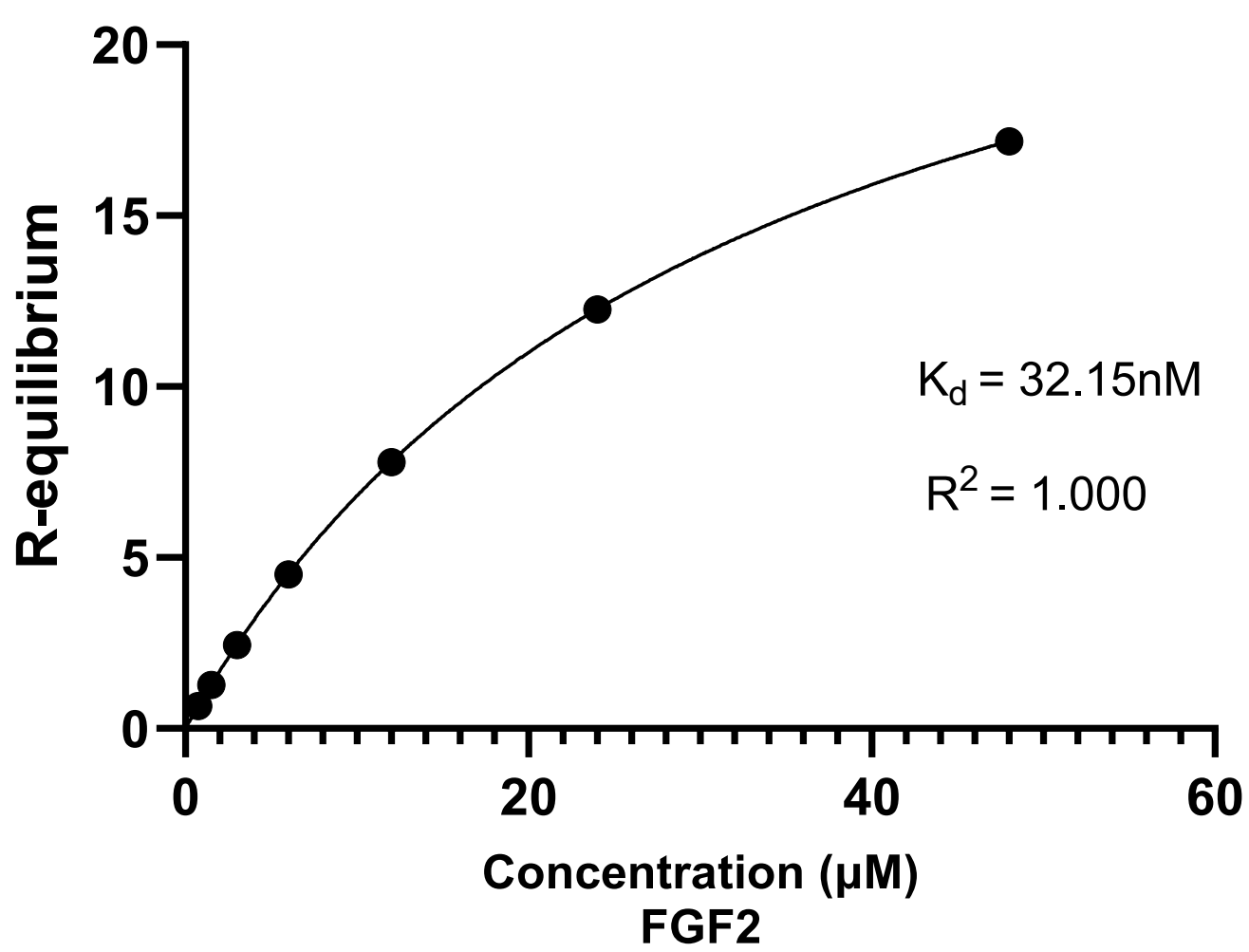

Figure S4. Binding affinity (K_d) of biotinylated heparin with FGF2

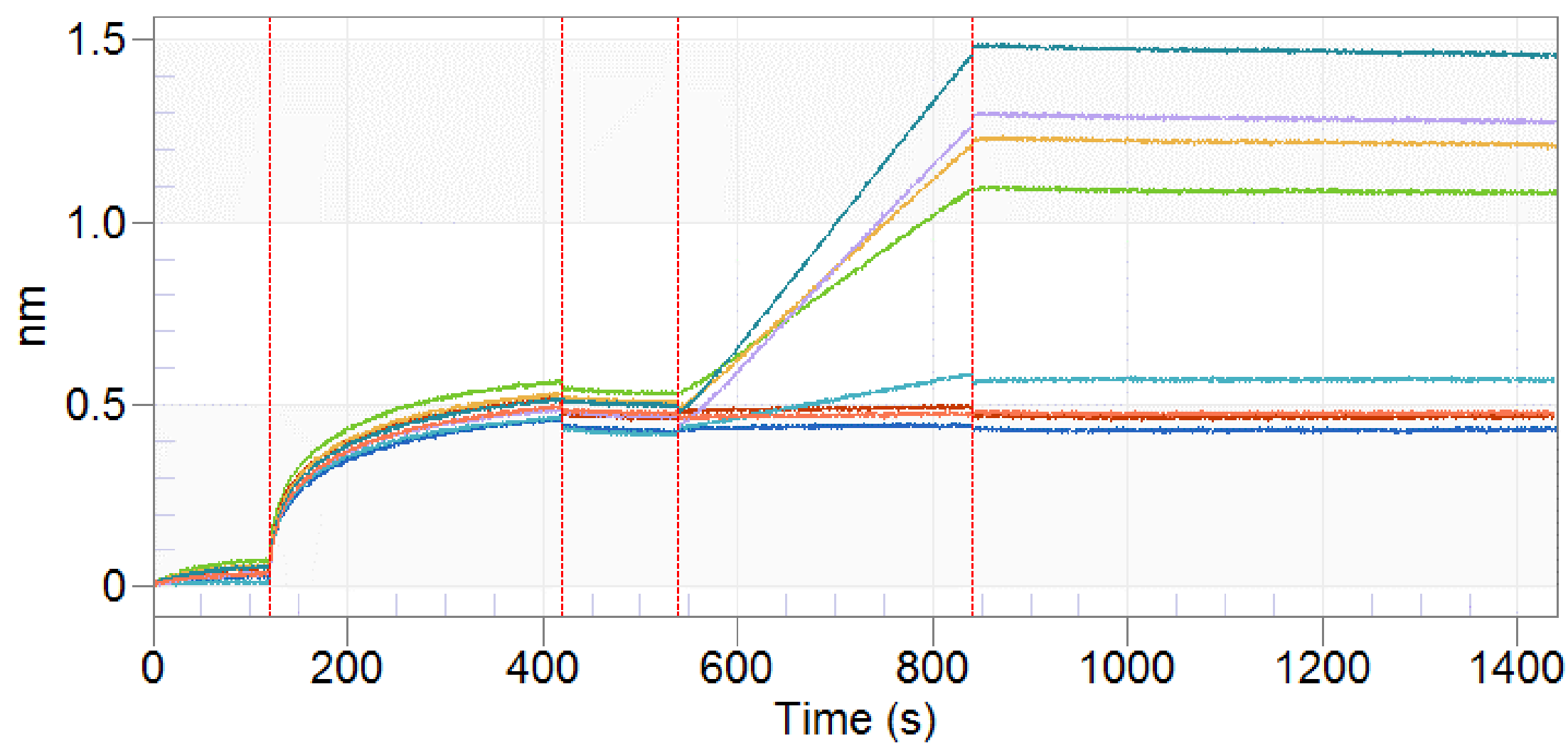

Figure S5. BLI sensorgram (raw data) of the competitive interaction between biotinylated heparin (5 μg/mL) and FGF2 (35 nM) with a three-fold serial dilution of compound **8** (21-5000 nM).

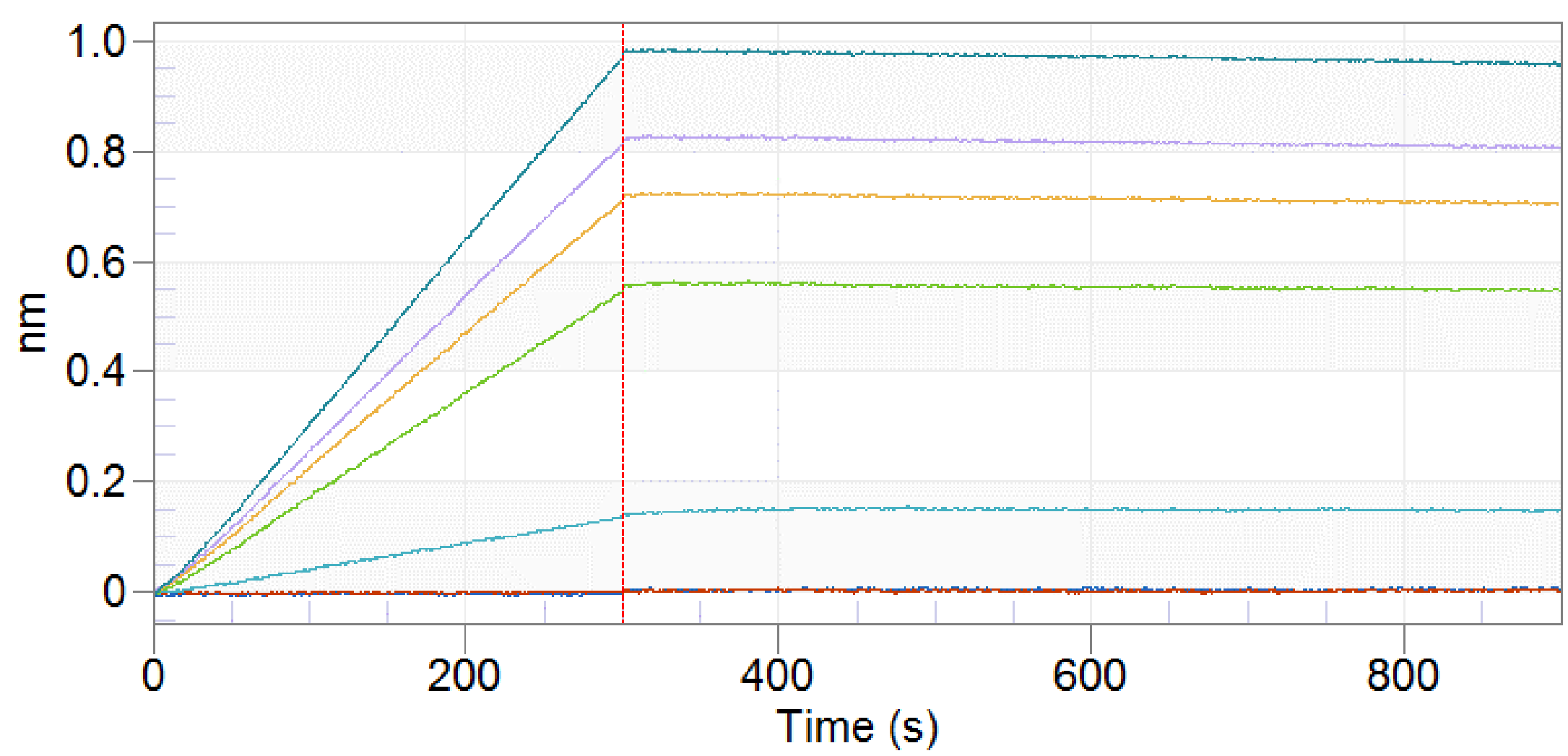

Figure S6. BLI sensorgram (processed data) of the competitive interaction between biotinylated heparin (5 μg/mL) and FGF2 (35 nM) with a three-fold serial dilution of compound **8** (21-5000 nM).

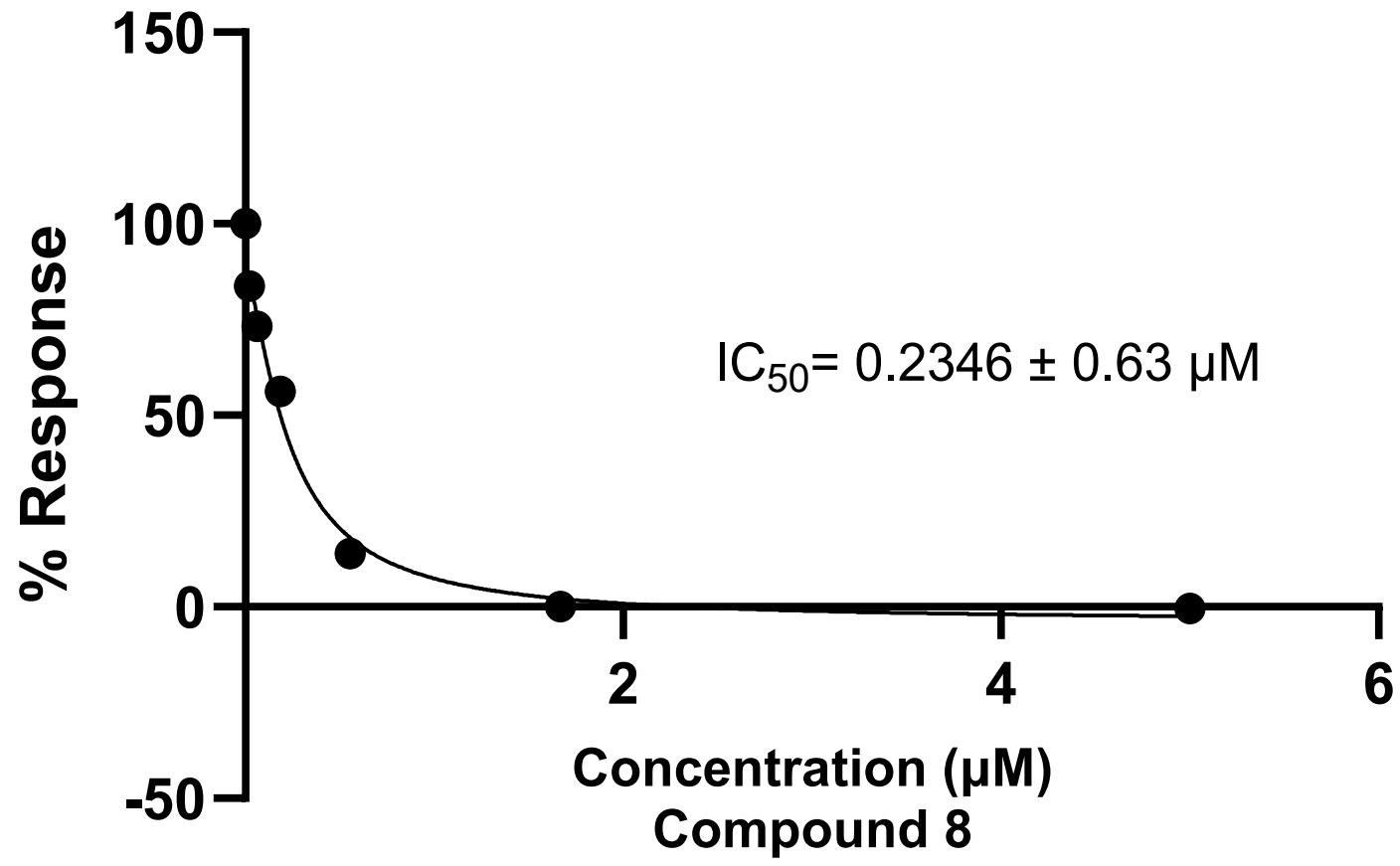

Figure S7. Inhibition activity (IC_{50}) of compound **8** against FGF2 for binding with biotinylated heparin.

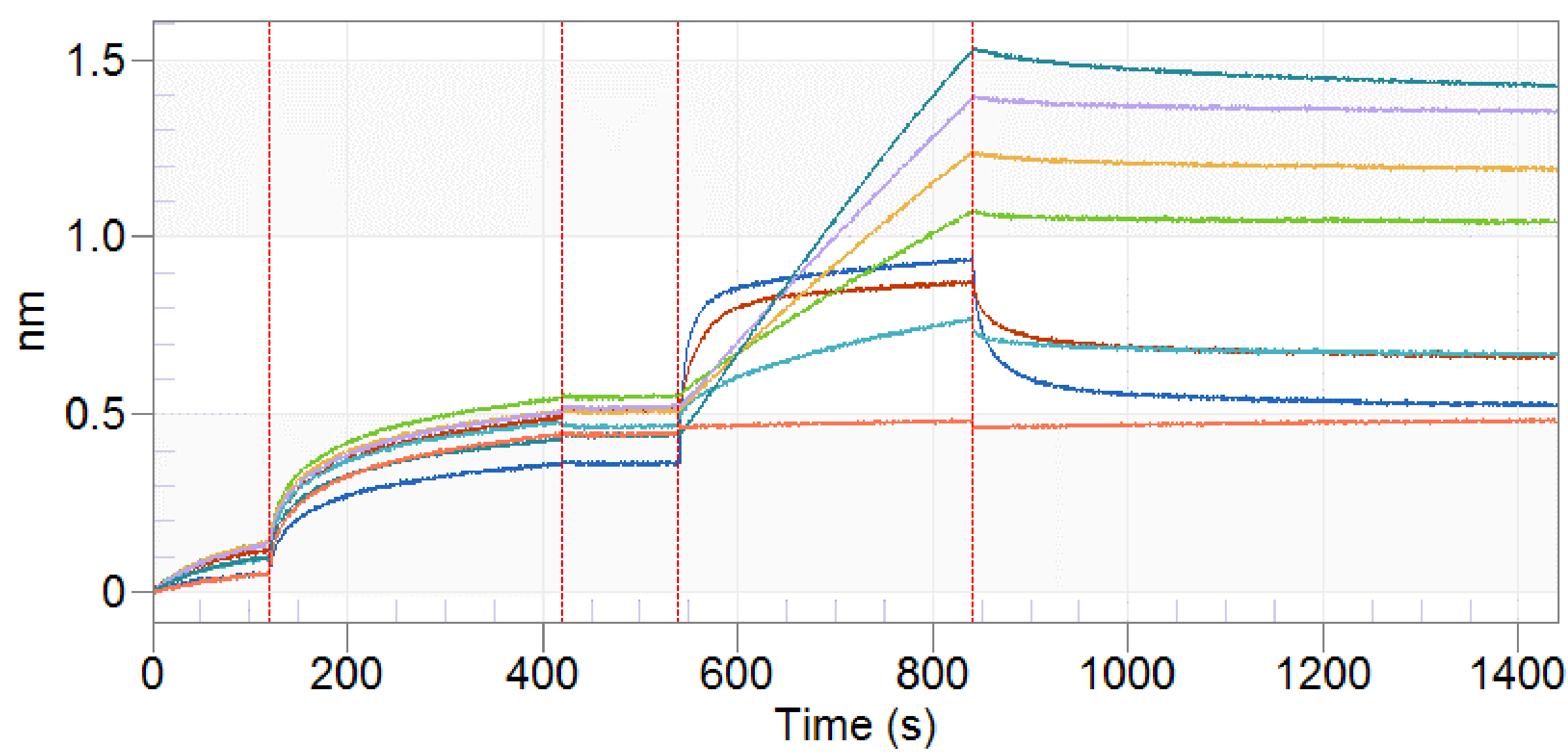

Figure S8. BLI sensorgram (raw data) of the competitive interaction between biotinylated heparin (5 µg/mL) and FGF2 (35 nM) with a three-fold serial dilution of compound **30** (0.21-50 µM).

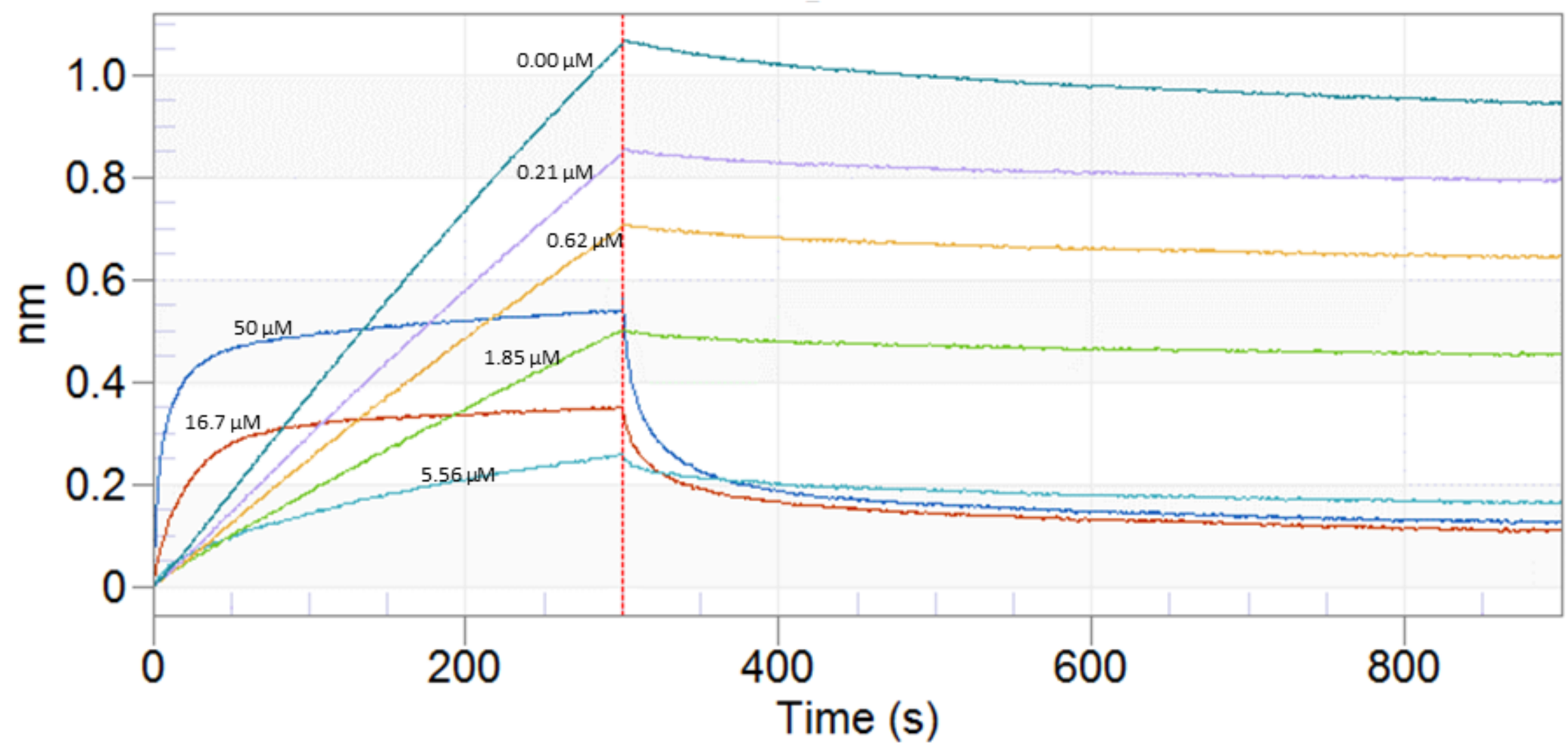

Figure S9. BLI sensorgram (processed data) of the competitive interaction between biotinylated heparin (5 µg/mL) and FGF2 (35 nM) with a three-fold serial dilution of compound **30** (0.21-50 µM).

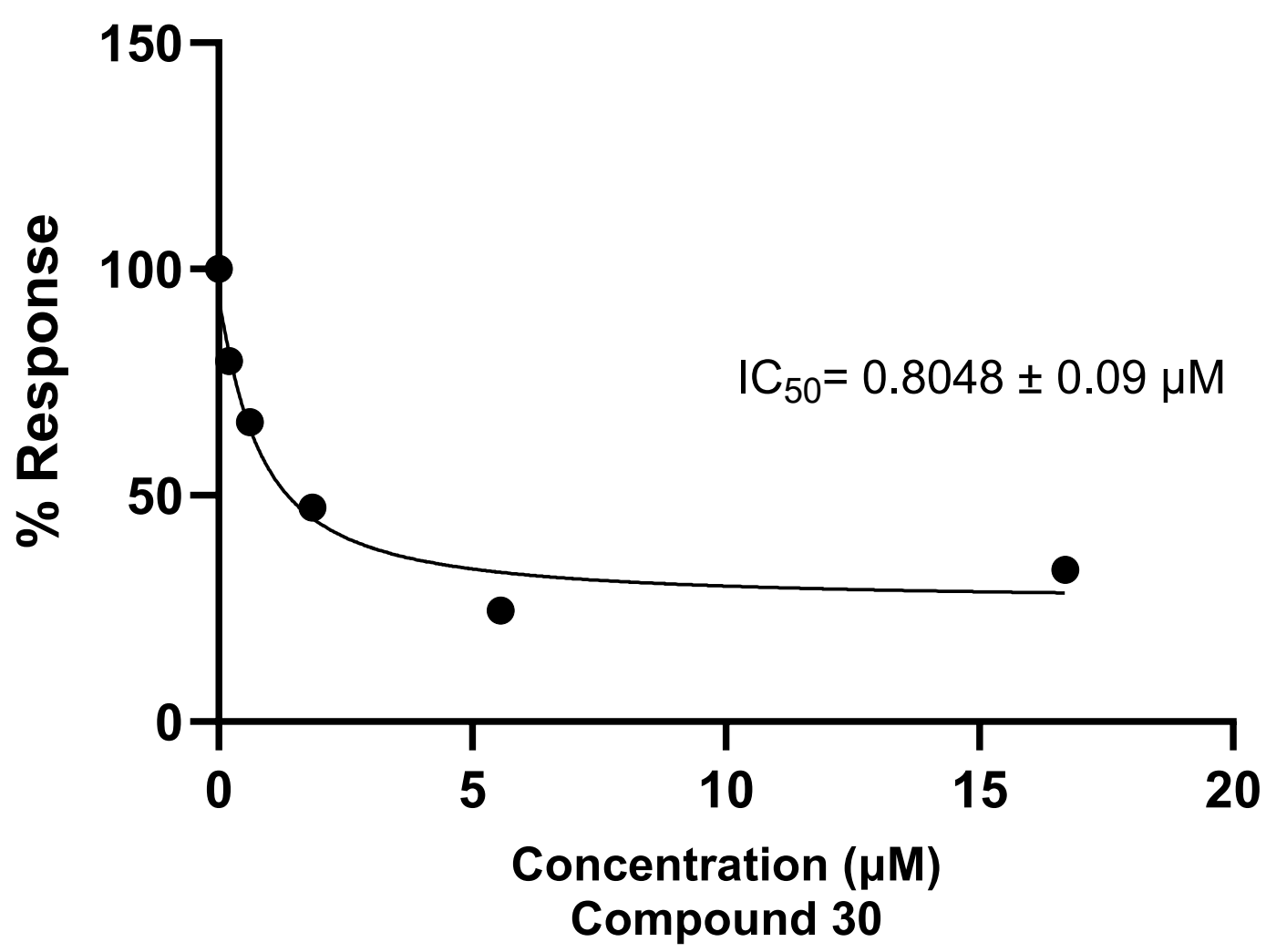

Figure S10. Inhibition activity (IC_{50}) of compound **30** against FGF2 for binding with biotinylated heparin.

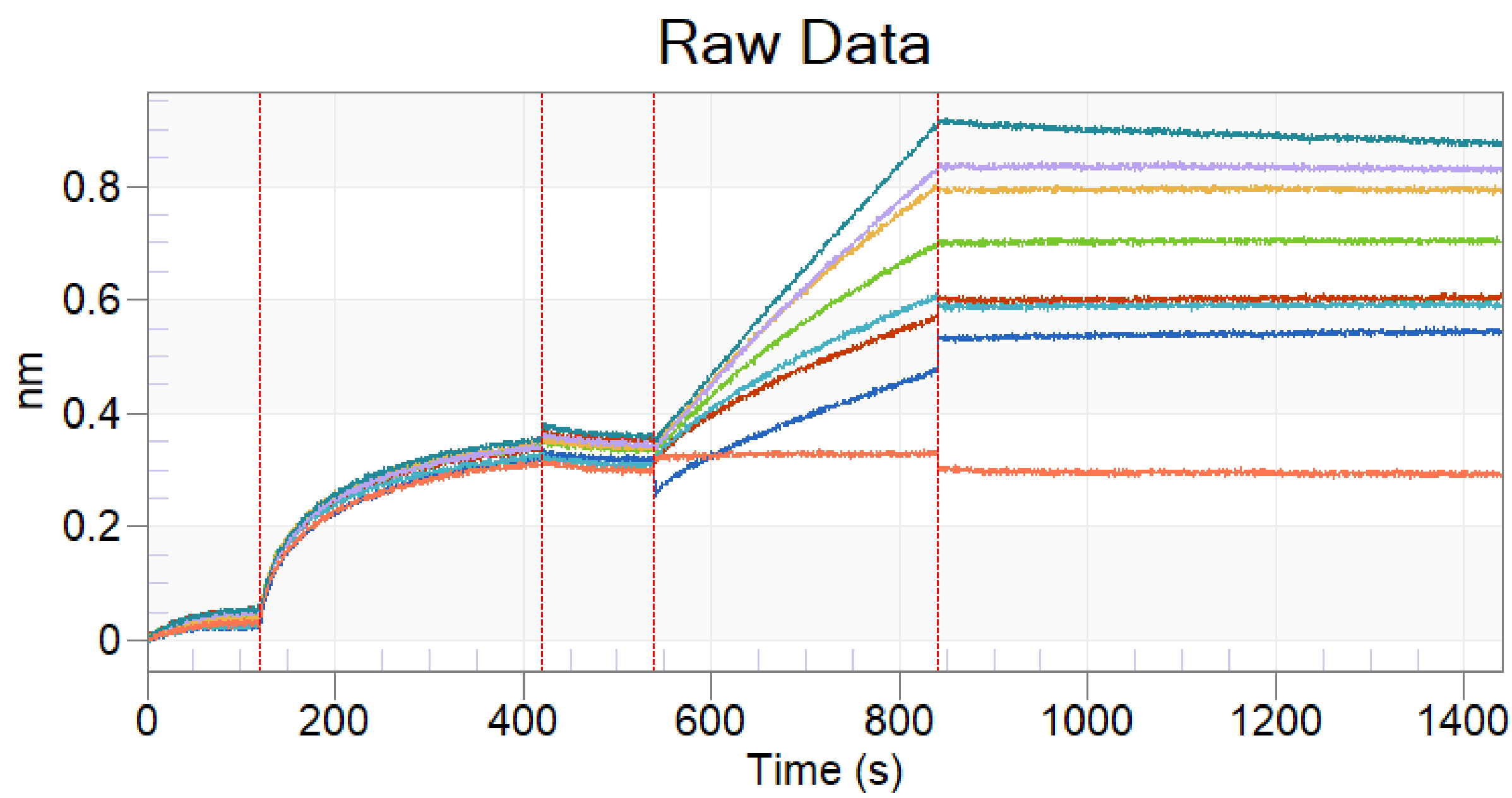

Figure S11. BLI sensorgram (raw data) of the competitive interaction between biotinylated heparin (5 μg/mL) and FGF2 (35 nM) with a two-fold serial dilution of compound **49** (15.63-500 μM).

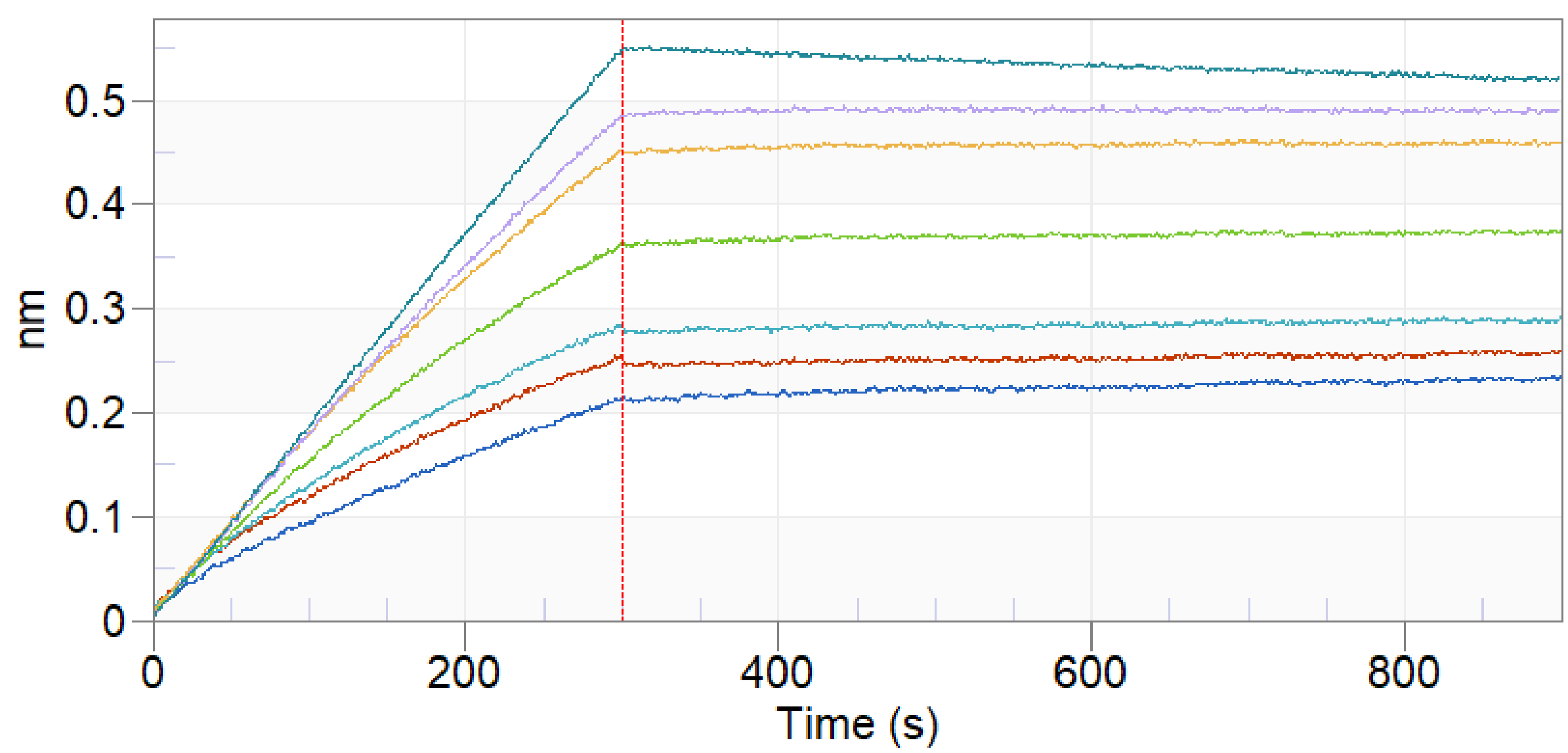

Figure S12. BLI sensorgram (processed data) of the competitive interaction between biotinylated heparin (5 μg/mL) and FGF2 (35 nM) with a two-fold serial dilution of compound **49** (15.63-500 μM).

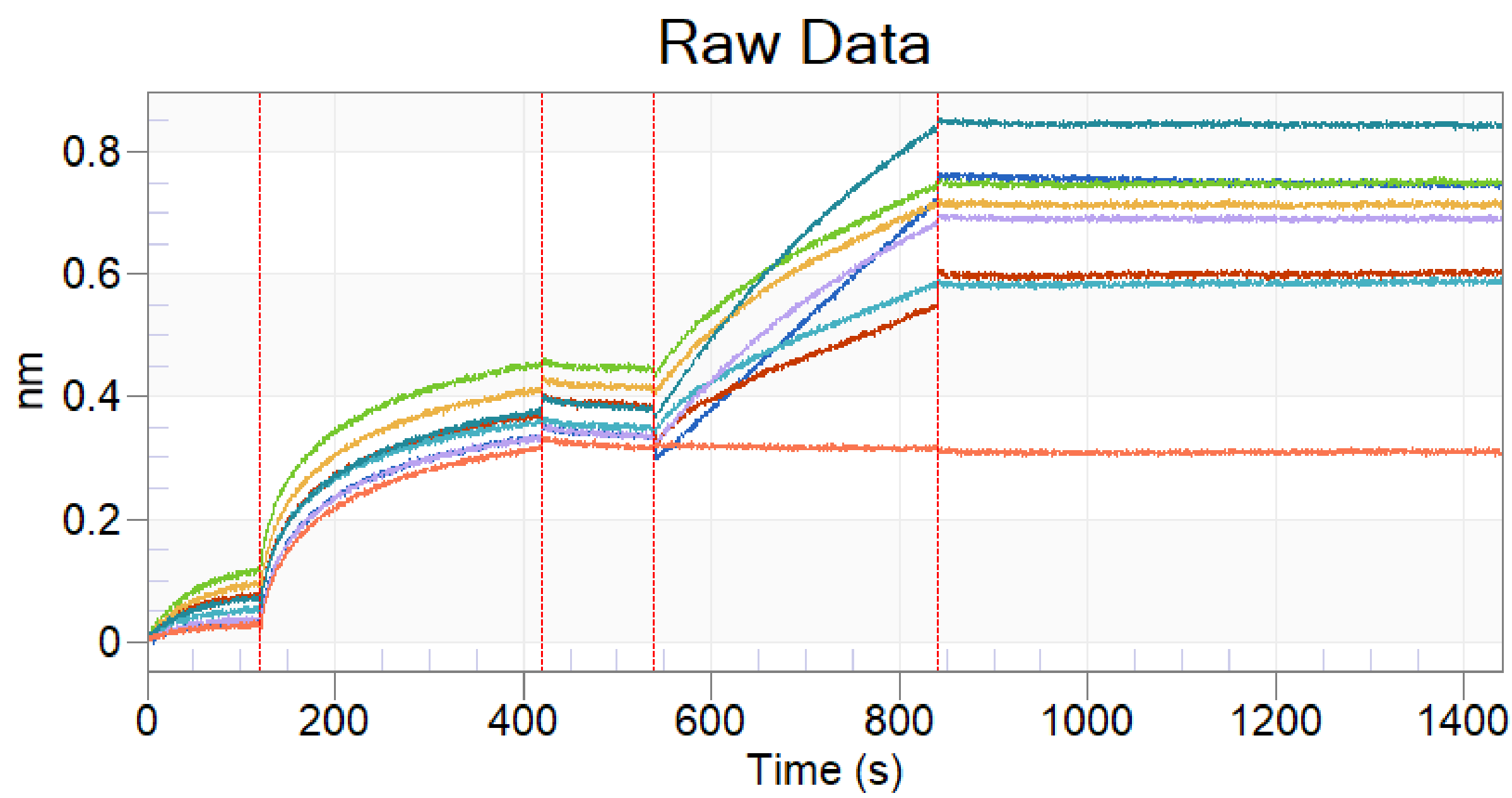

Figure S13. BLI sensorgram (raw data) of the competitive interaction between biotinylated heparin (5 µg/mL) and FGF2 (35 nM) with a two-fold serial dilution of compound **50** (12.5-400 µM).

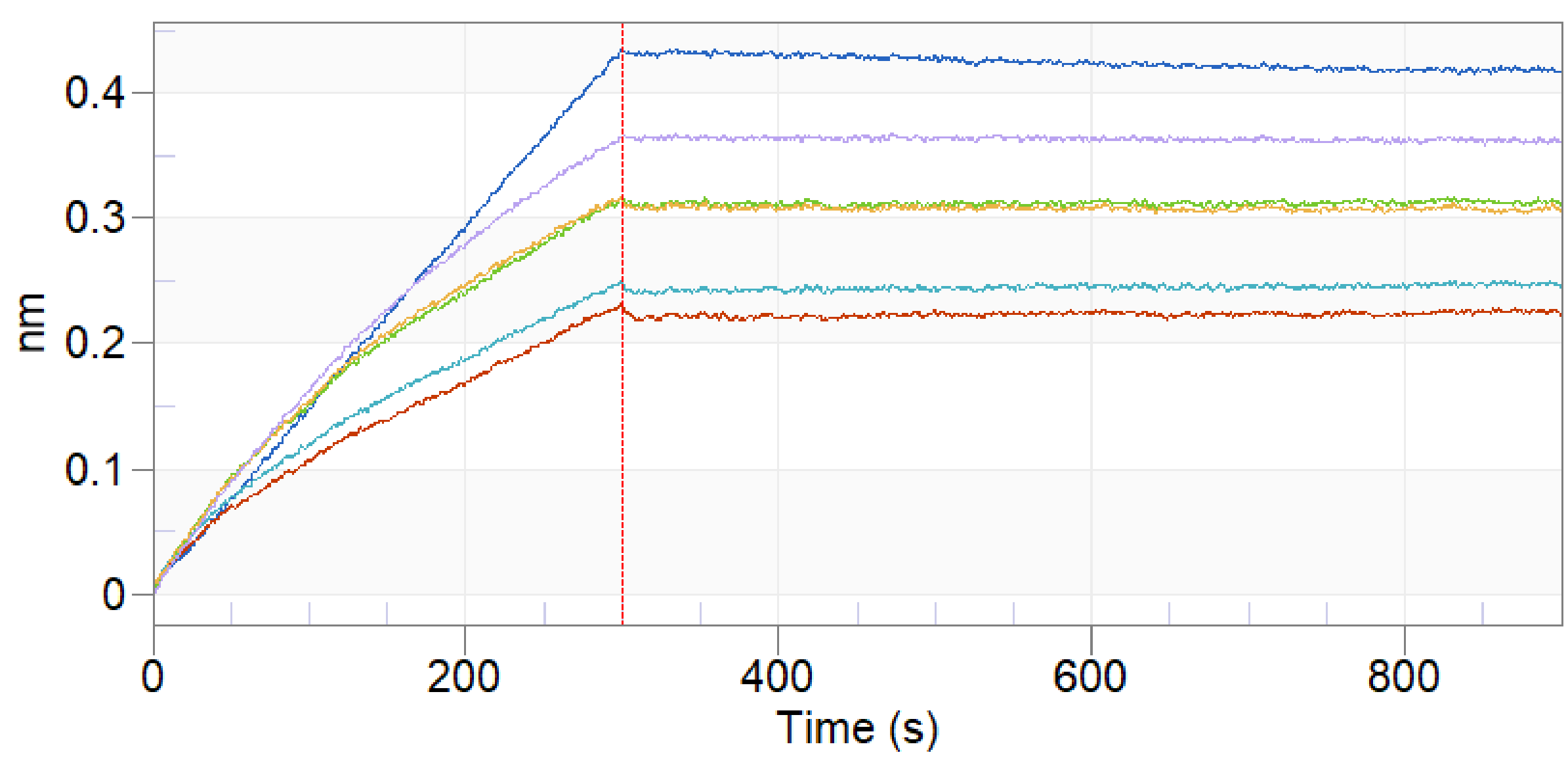

Figure S14. BLI sensorgram (processed data) of the competitive interaction between biotinylated heparin (5 µg/mL) and FGF2 (35 nM) with a two-fold serial dilution of compound **50** (12.5-400 µM).

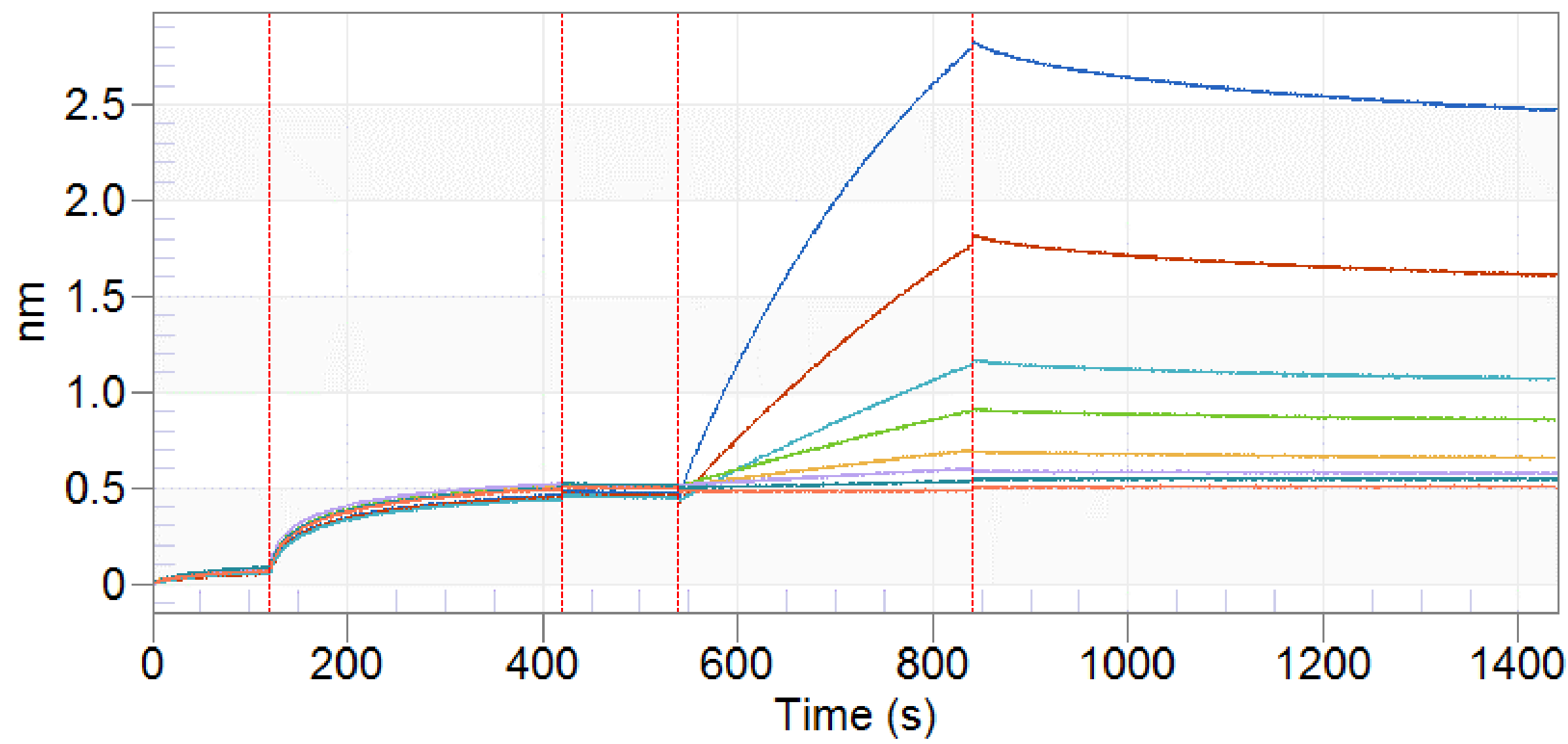

Figure S15. BLI sensorgram (raw data) of the interaction between biotinylated heparin (5 μg/mL) and a two-fold serial dilution of FGFR1 (15-970 nM).

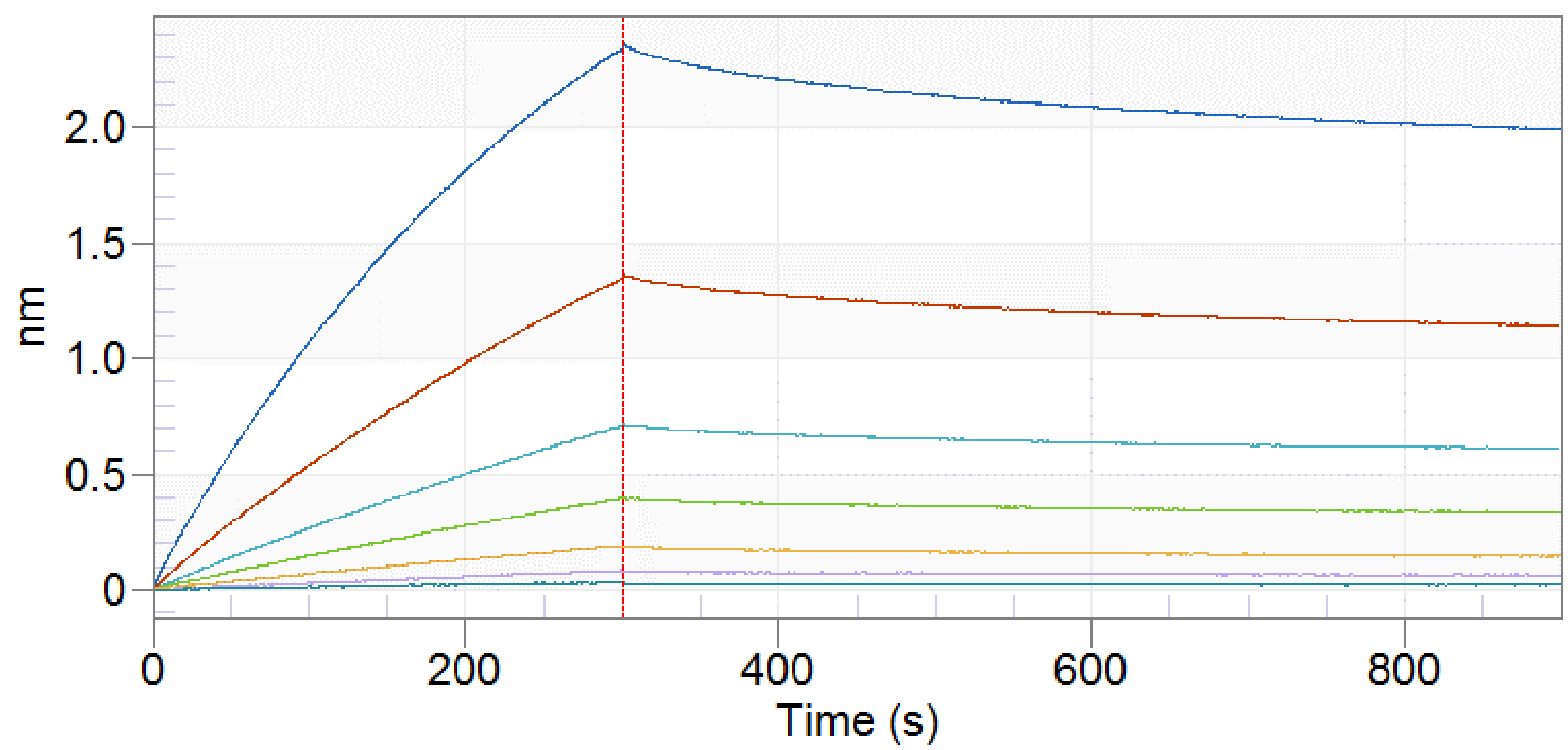

Figure S16. BLI sensorgram (processed data) of the interaction between biotinylated heparin (5 μg/mL) and a two-fold serial dilution of FGFR1 (15-970 nM).

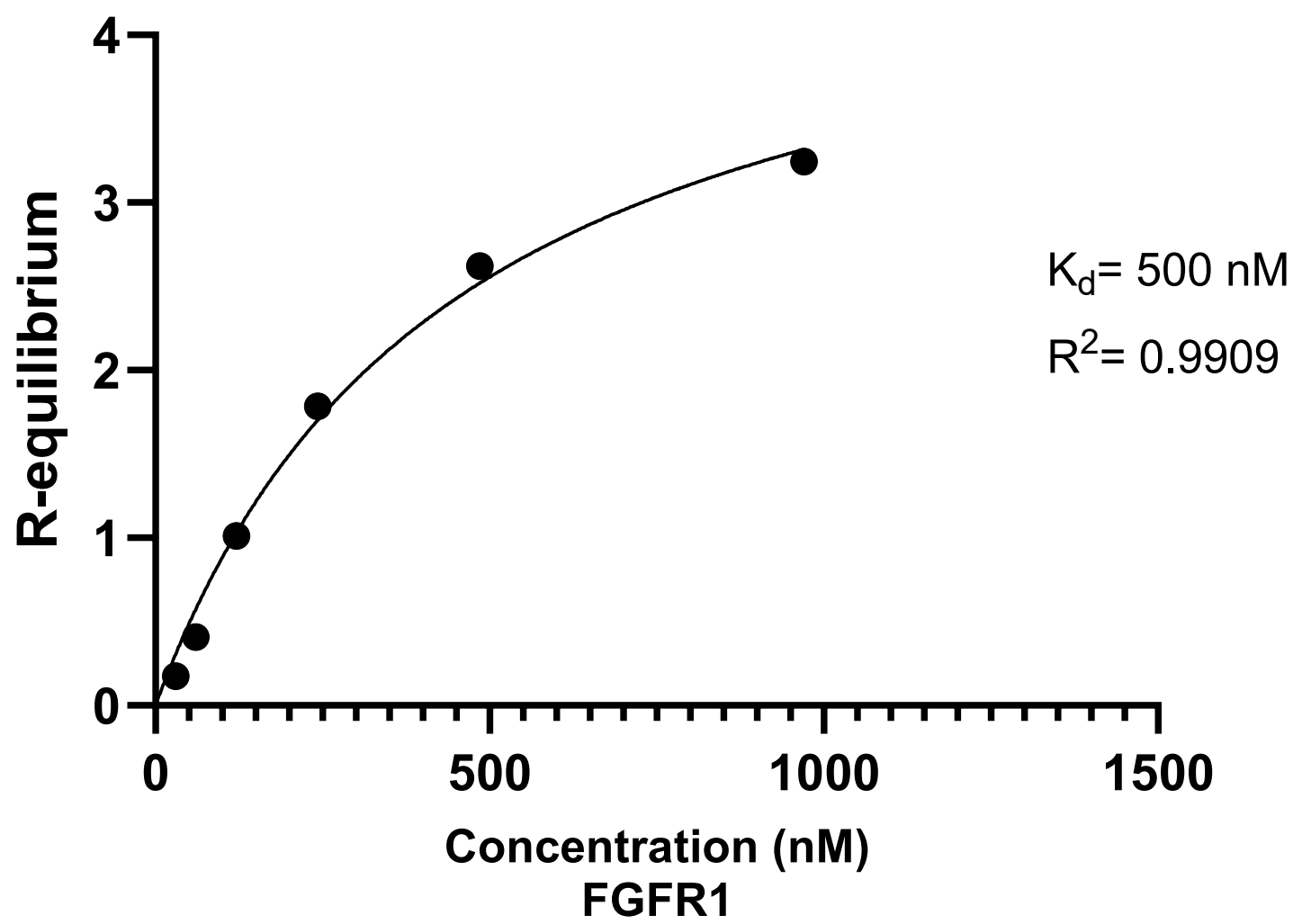

Figure S17. Binding affinity (K_d) of heparin with FGFR1

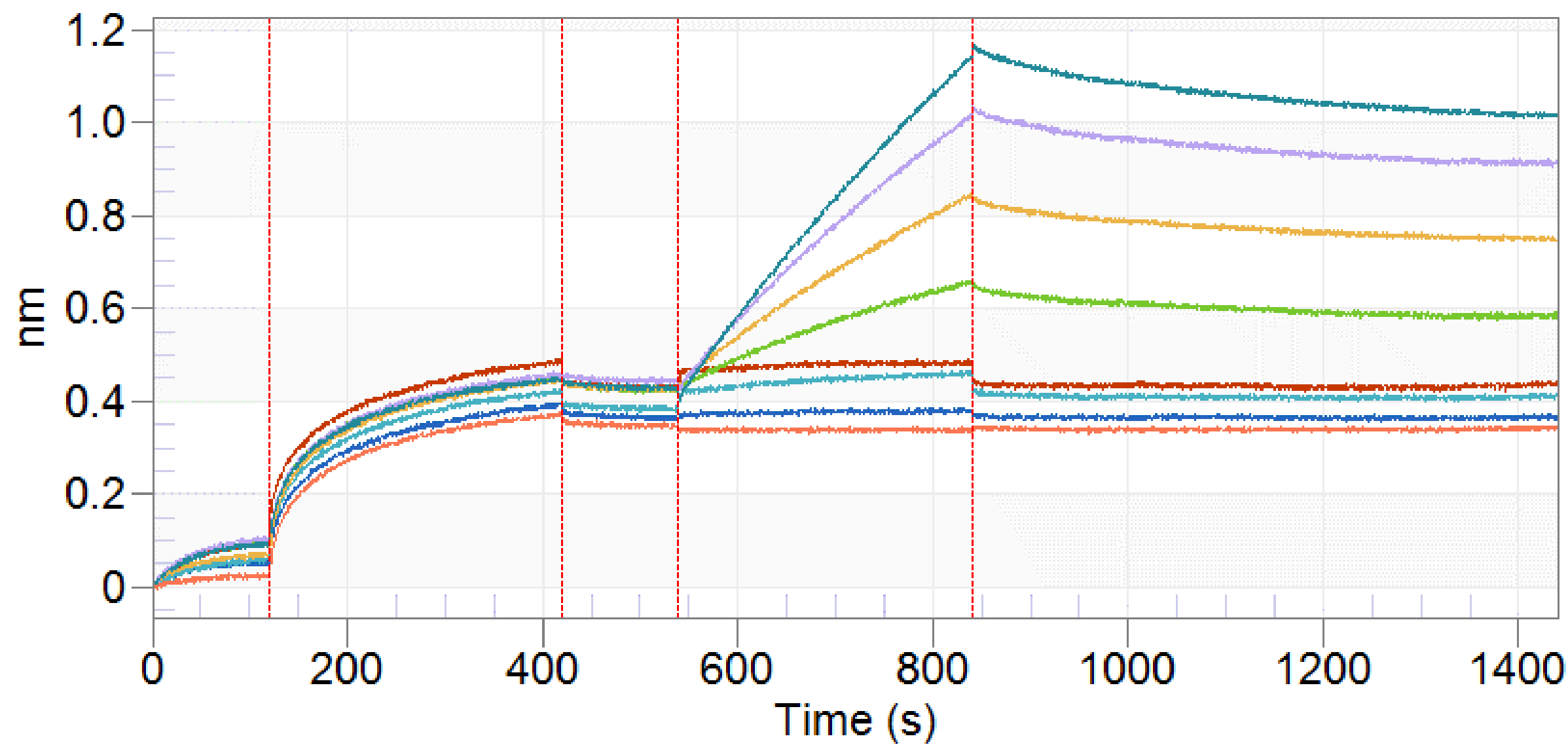

Figure S18. BLI sensorgram (raw data) of the competitive interaction between biotinylated heparin (5 μg/mL) and FGFR1 (10 μg/mL) with a two-fold serial dilution of compound **8** (78.1-2500 nM).

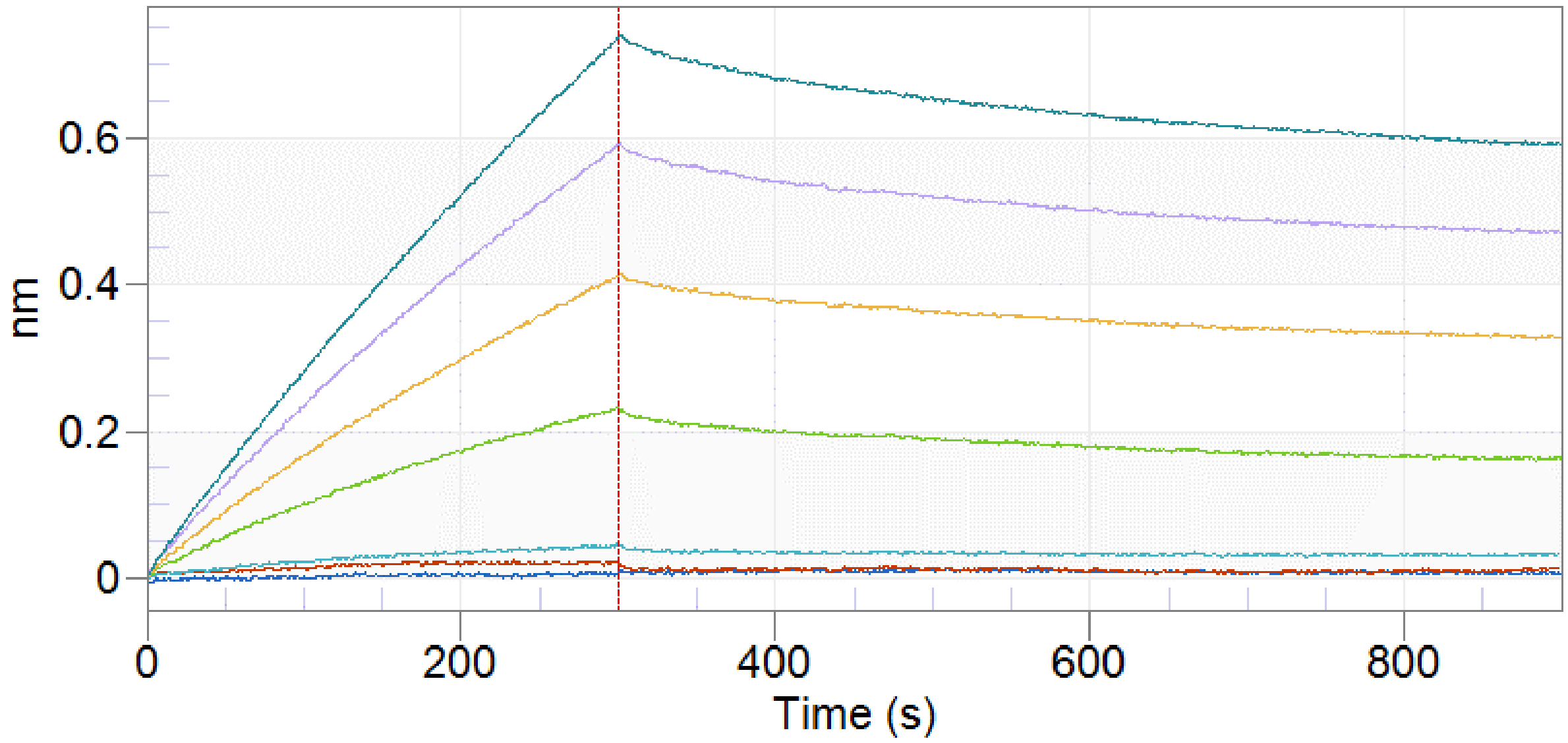

Figure S19. BLI sensorgram (processed data) of the interaction between biotinylated heparin (5 µg/mL) and FGFR1 (10 µg/mL) with a two-fold serial dilution of compound **8** (78.1-2500 nM).

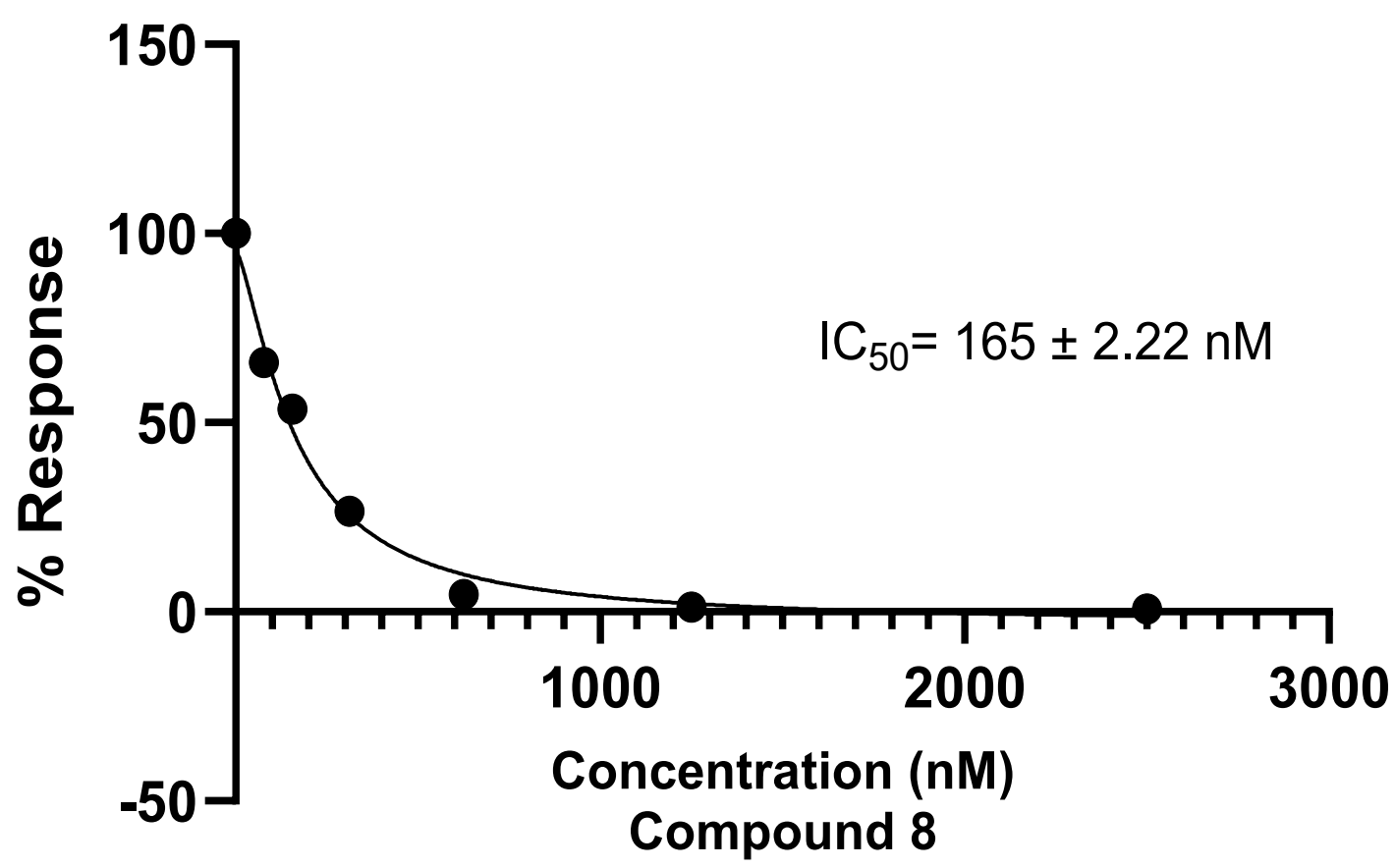

Figure S20. Inhibition activity (IC_{50}) of compound **8** against FGFR1 for binding with biotinylated heparin

Biolayer interferometry assay (BLI) data of ligands in complex with $VEGF_{165}$

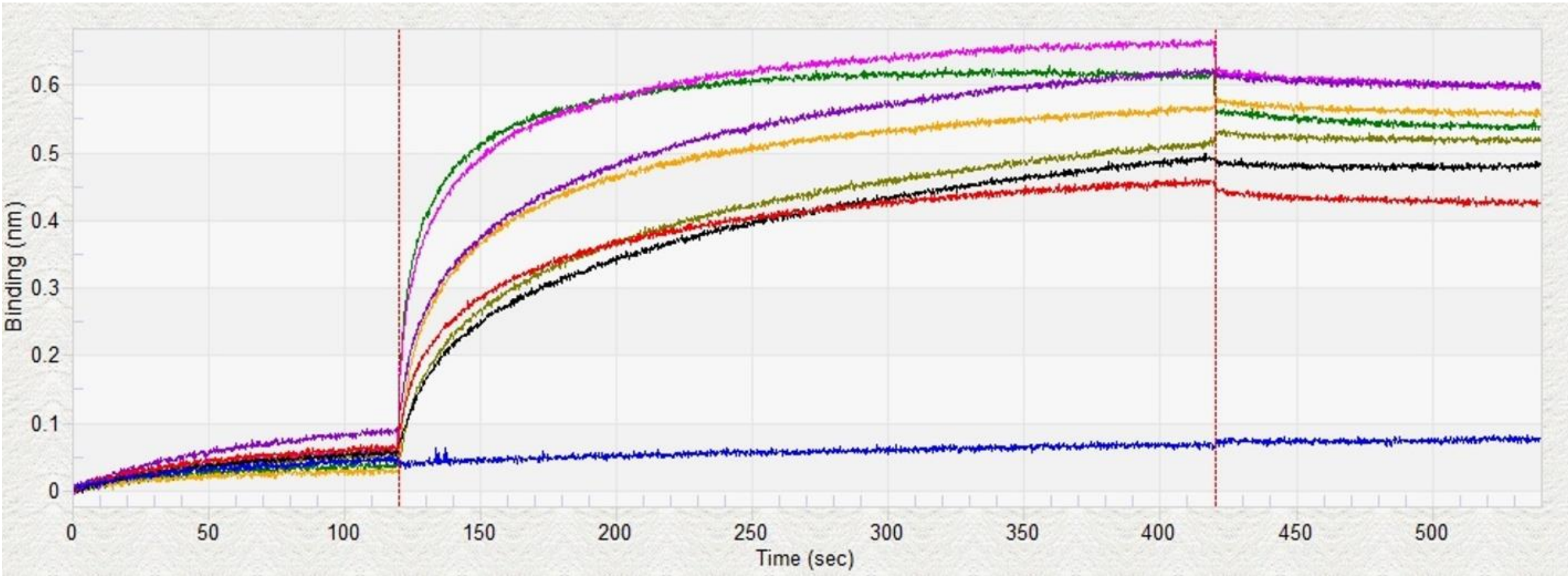

Figure S21. BLI sensorgram (raw data) shows the determination of an optimal binding curve for biotinylated heparin (27 kDa). A 2-fold serial dilution of six concentrations (1-30 μg/mL) were tested with a good binding curve established at 1 μg/mL. Red is reference (i.e. biotinylated heparin, 18 kDa) and green is the highest concentration.

A)

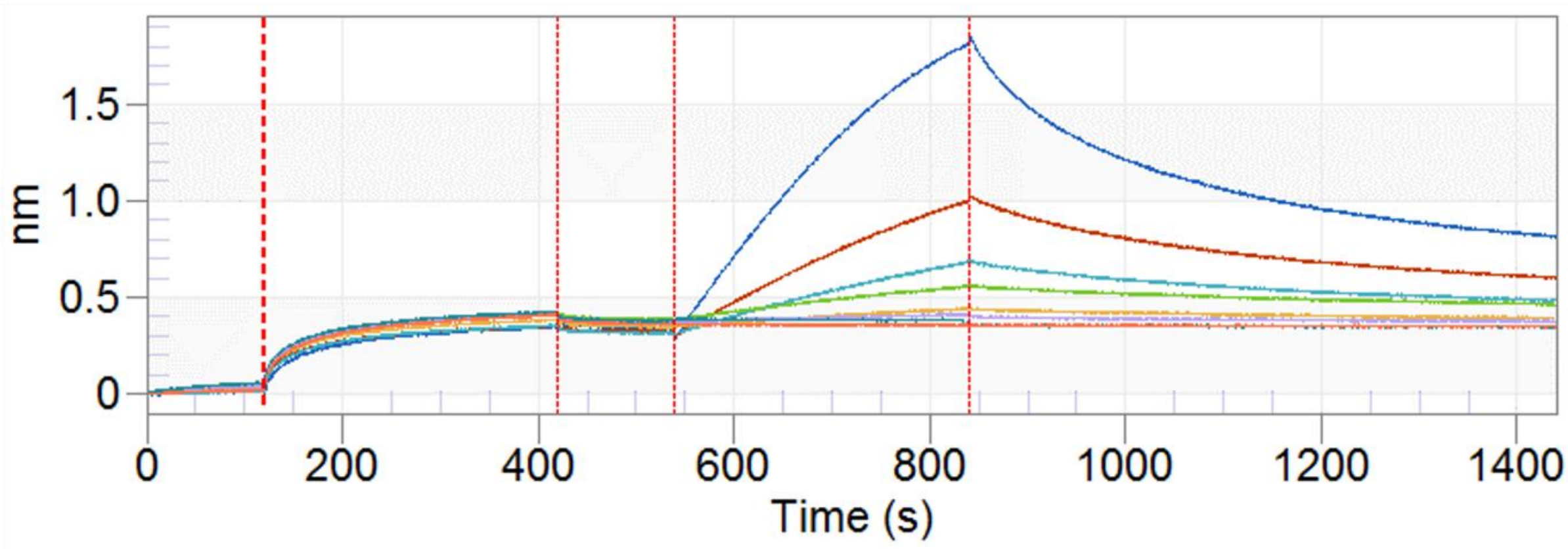

B)

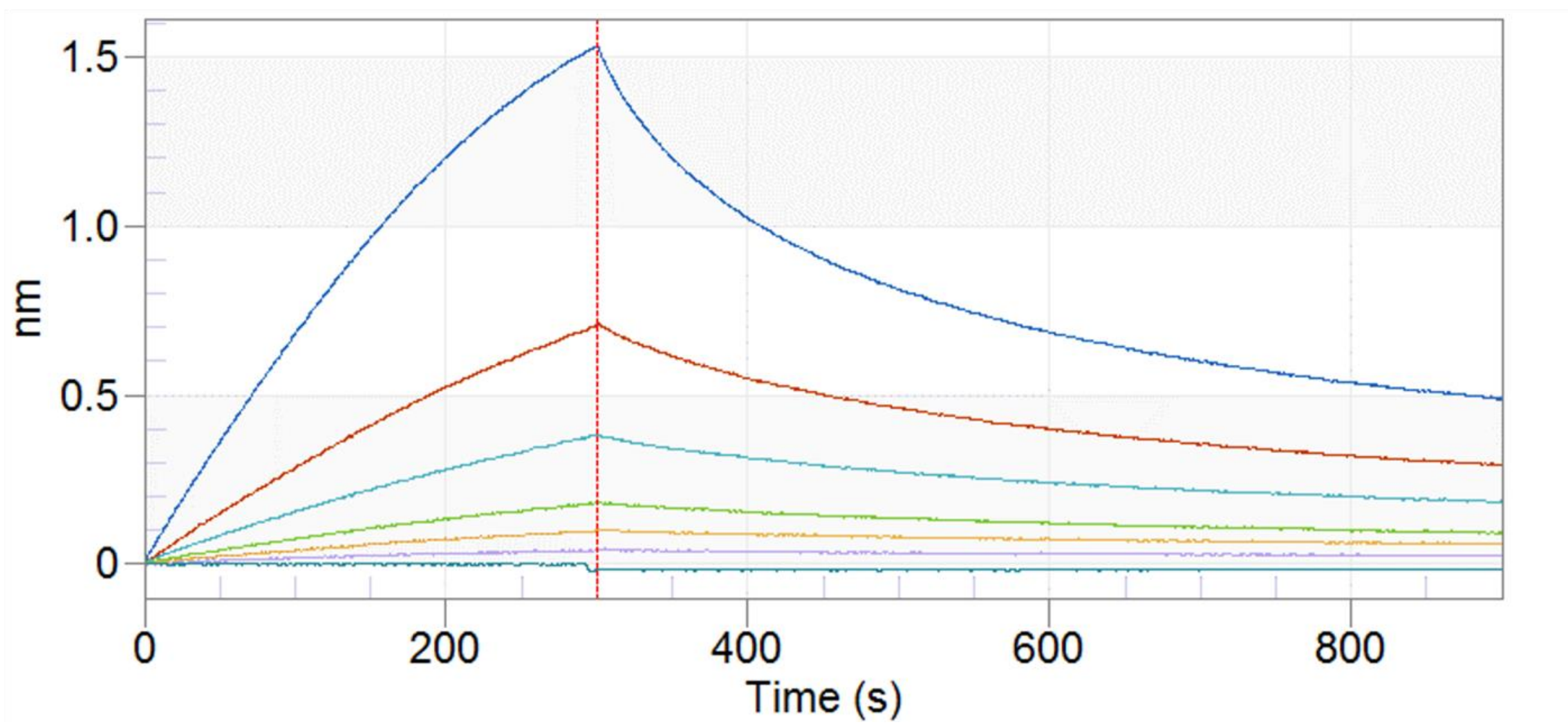

Figure S22. A.) BLI sensorgram (raw data) shows the determination of a standard curve for recombinant human $VEGF_{165}$. A 2-fold serial dilution of six concentrations (1.6 nM – 52 nM) were tested with a good binding curve established at 25 nM. B.) Processed data of the standard curve of $VEGF_{165}$ using BLI.

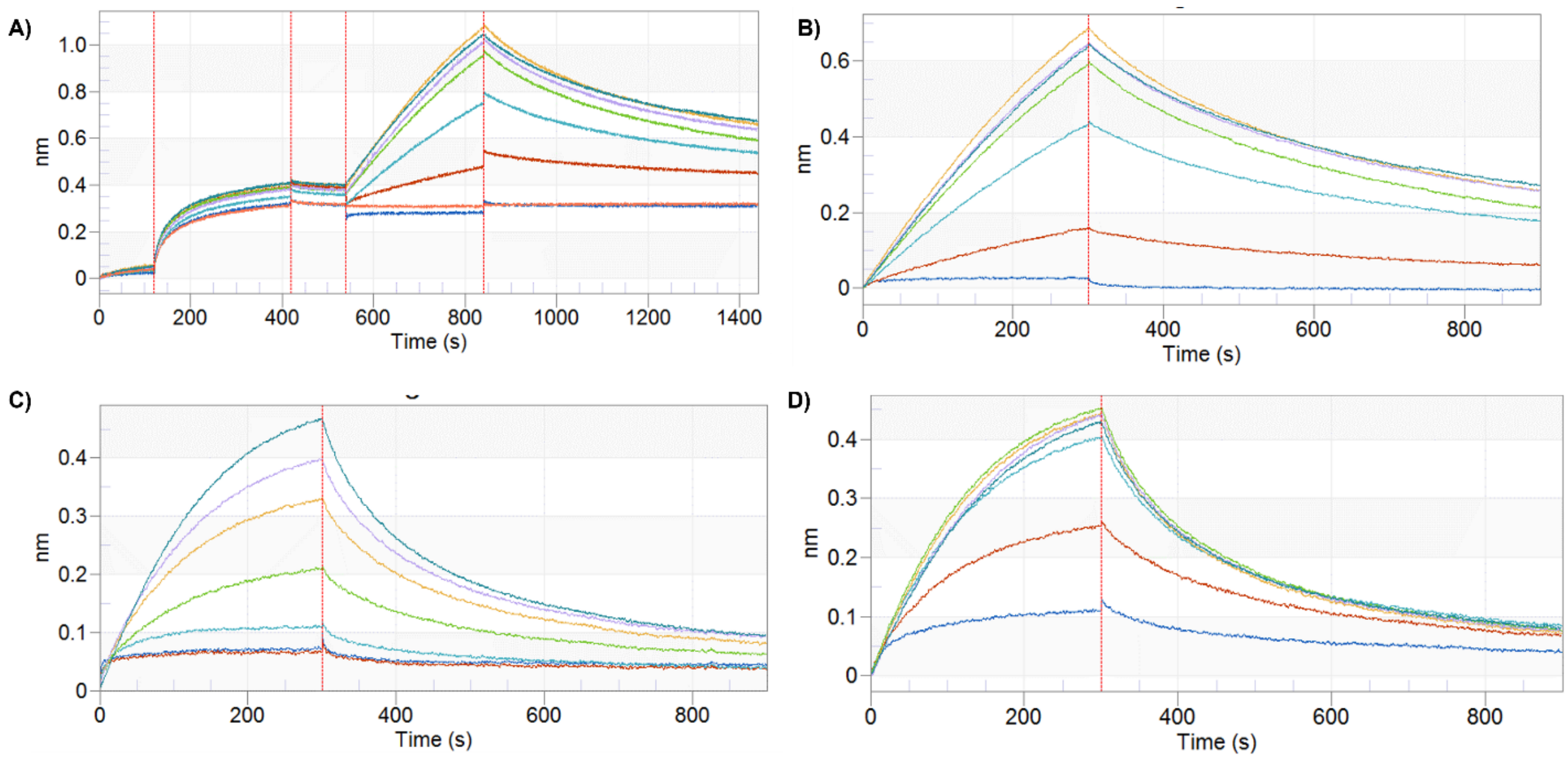

Figure S23. A) BLI sensorgram (raw data) of the interaction between biotinylated heparin (1 μg/mL), $VEGF_{165}$ (25 nM) and compound **8** (0.06-2 μM). B) Processed sensorgram of the competitive interaction between biotinylated heparin (1 μg/mL), $VEGF_{165}$ and compound **8**. C) BLI sensorgram (processed data) of the interaction between biotinylated heparin, $VEGF_{165}$ and compound **35** (3.23-100 μM). D) BLI sensorgram (processed data) of the interaction between biotinylated heparin, $VEGF_{165}$ and compound **37** (3.23-100 μM).

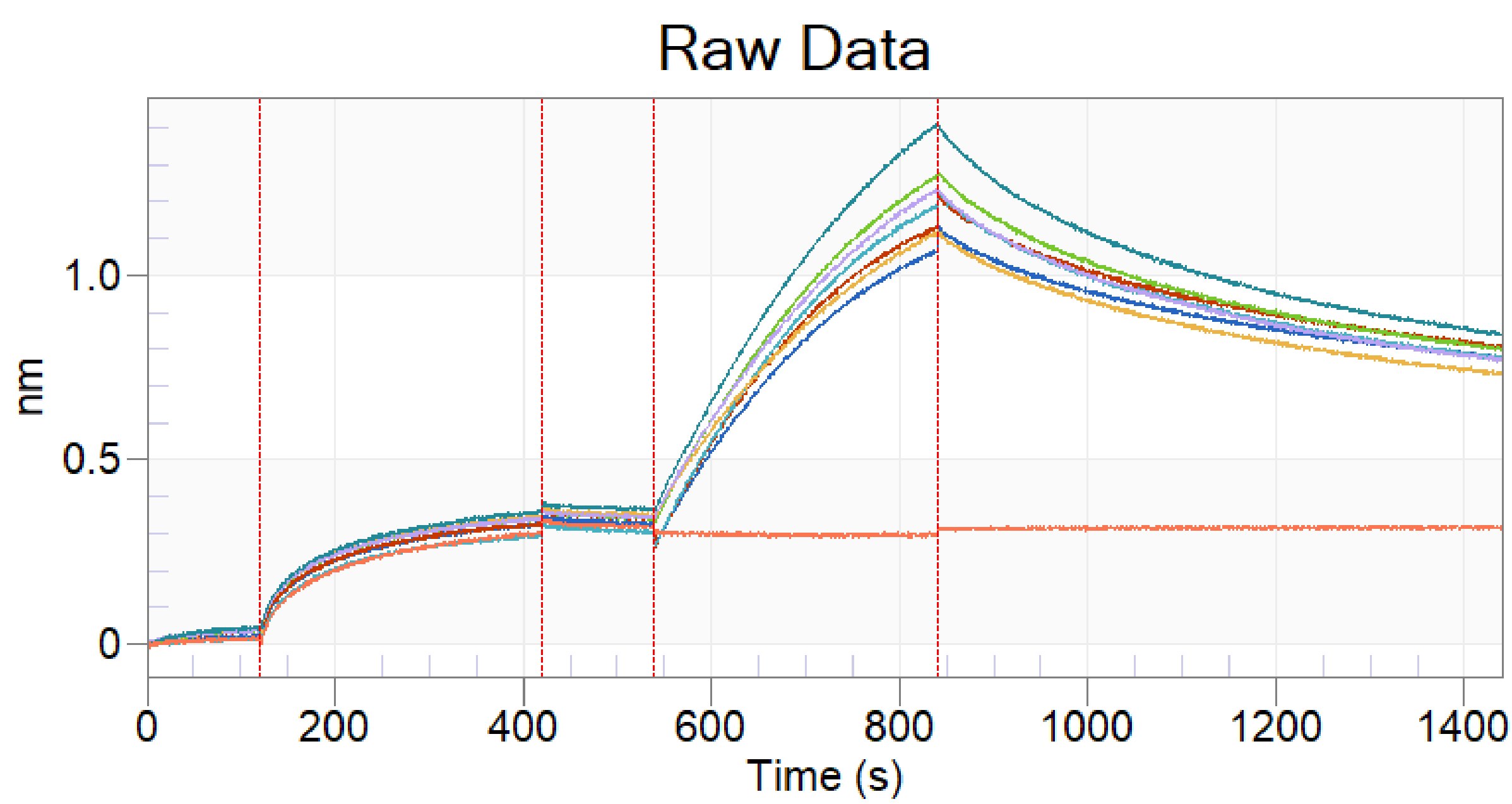

Figure S24. BLI sensorgram (raw data) of the competitive interaction between biotinylated heparin (5 µg/mL) and VEGF$_{165}$ (35 nM) with a two-fold serial dilution of compound **49** (15.63-500 µM).

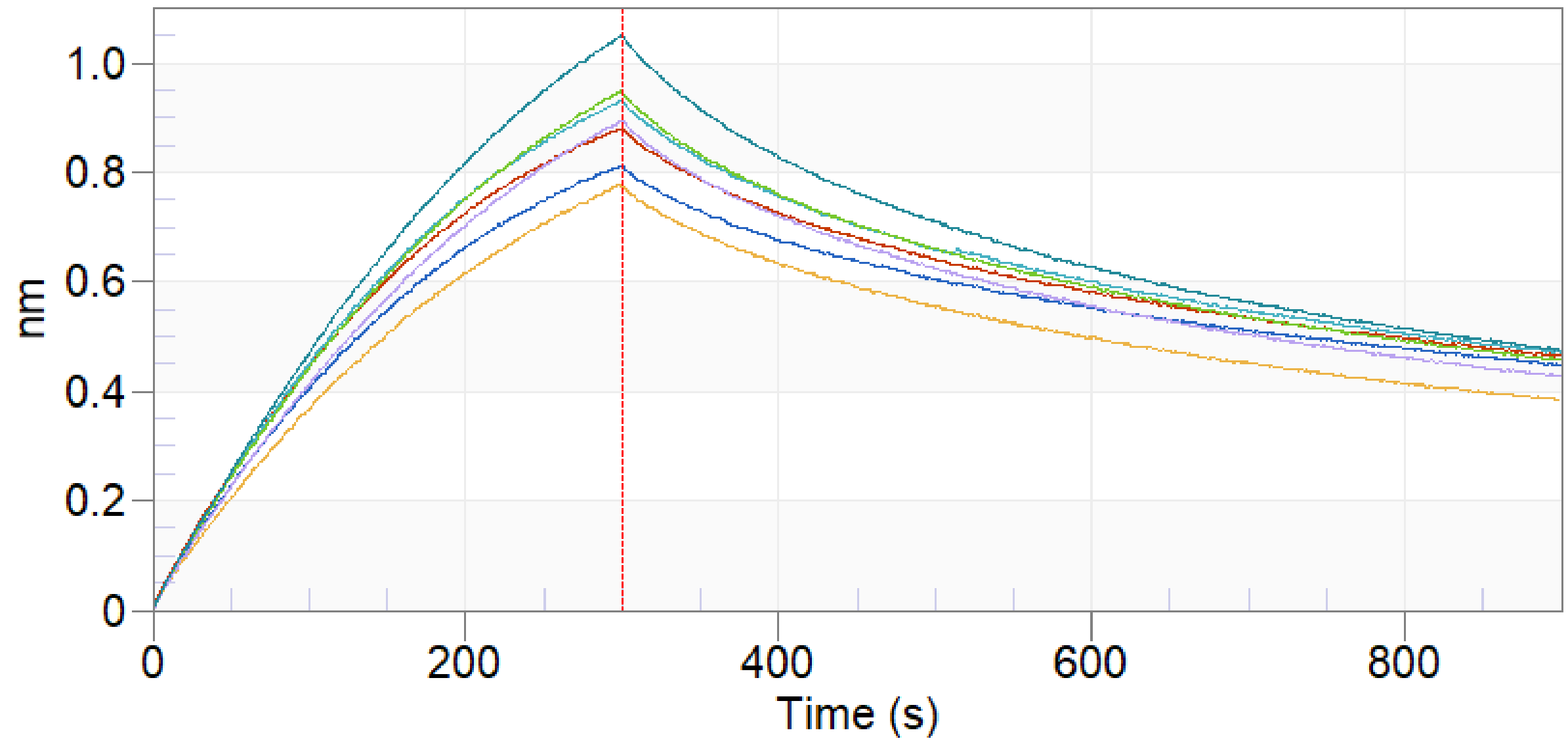

Figure S25. BLI sensorgram (raw data) of the competitive interaction between biotinylated heparin (5 µg/mL) and VEGF$_{165}$ (35 nM) with a two-fold serial dilution of compound **49** (15.63-500 µM).

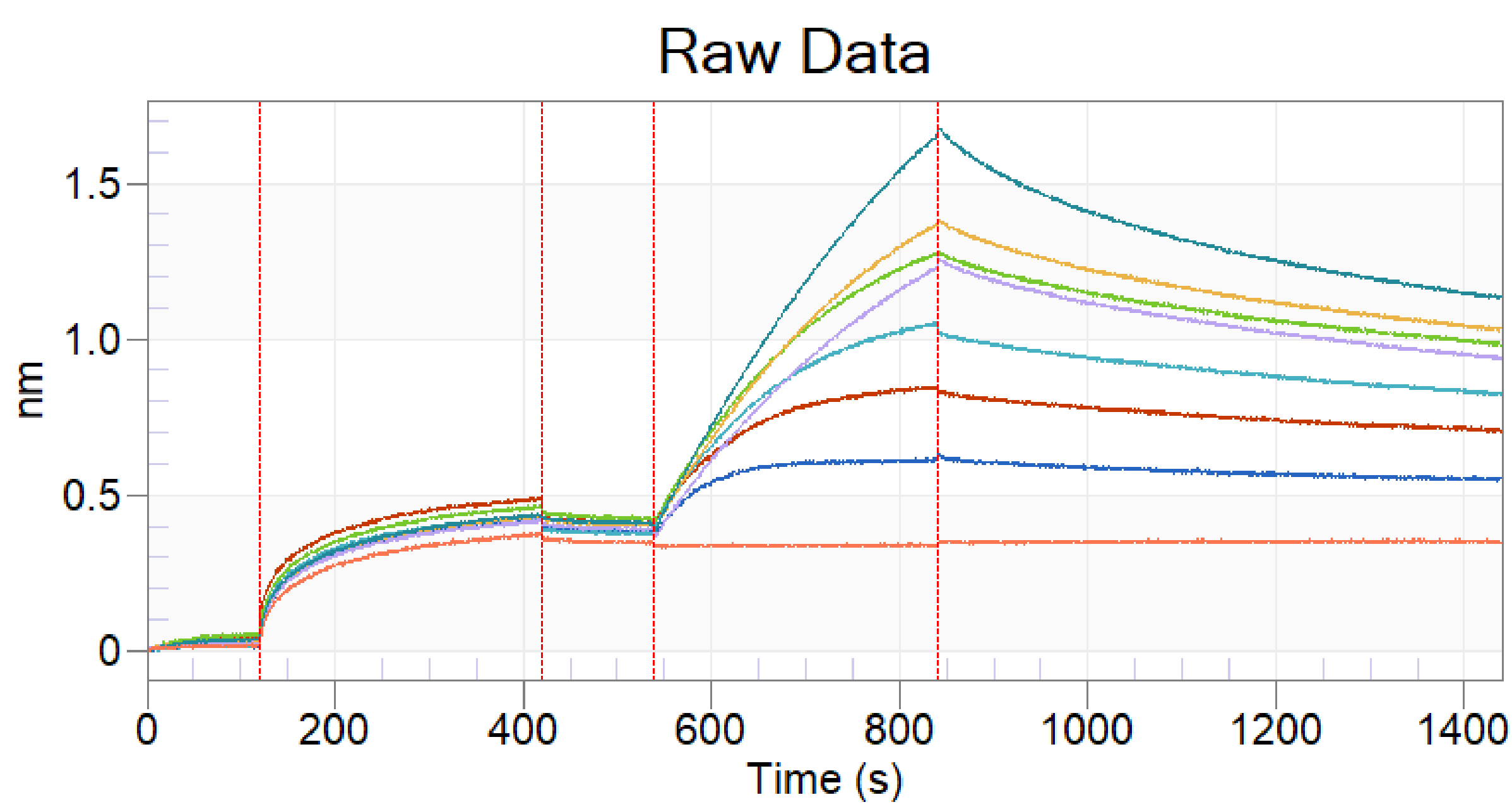

Figure S26. BLI sensorgram (raw data) of the competitive interaction between biotinylated heparin (5 µg/mL) and VEGF$_{165}$ (35 nM) with a two-fold serial dilution of compound **50** (15.63-250 µM).

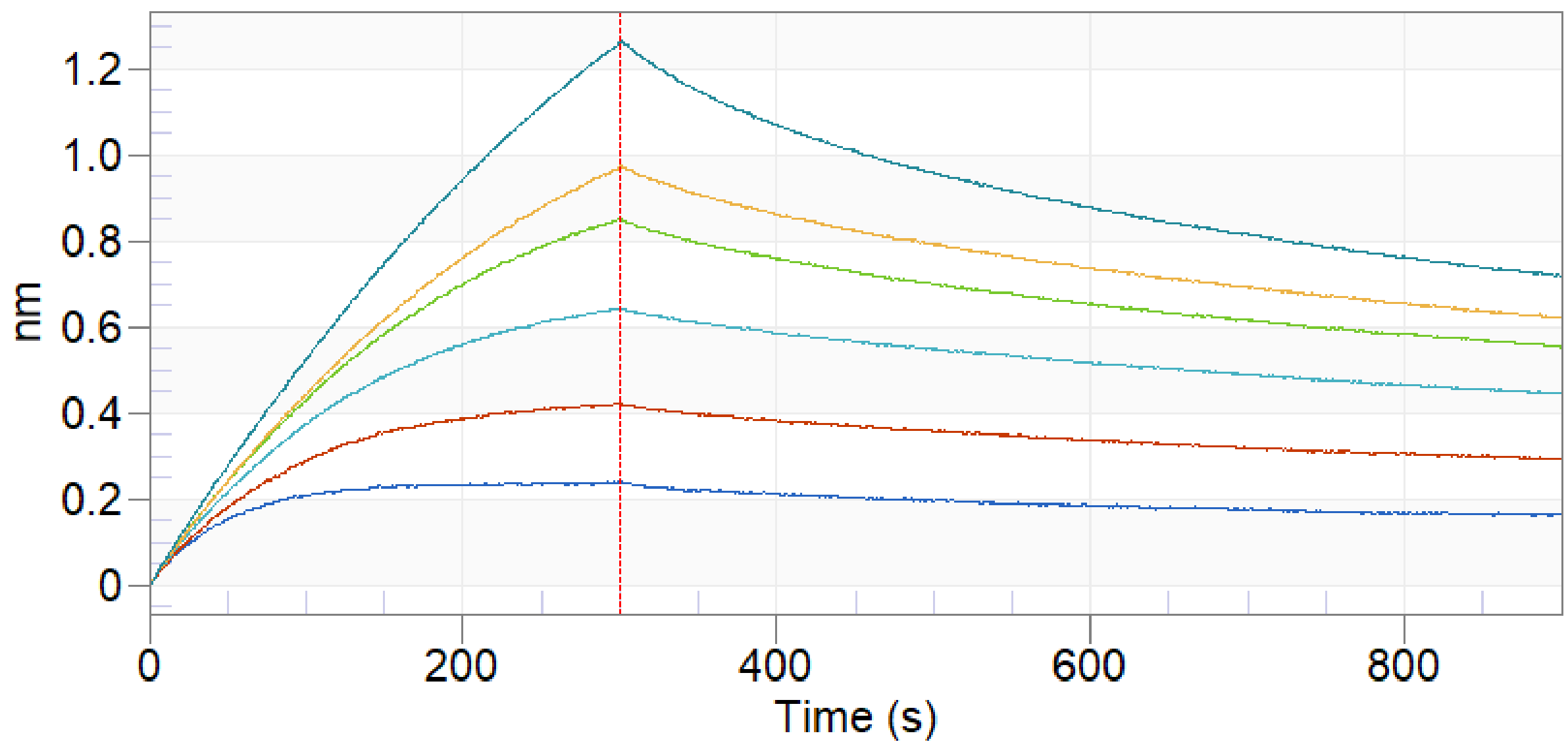

Figure S27. BLI sensorgram (processed data) of the competitive interaction between biotinylated heparin (5 µg/mL) and $VEGF_{165}$ (35 nM) with a two-fold serial dilution of compound **50** (15.63-250 µM).

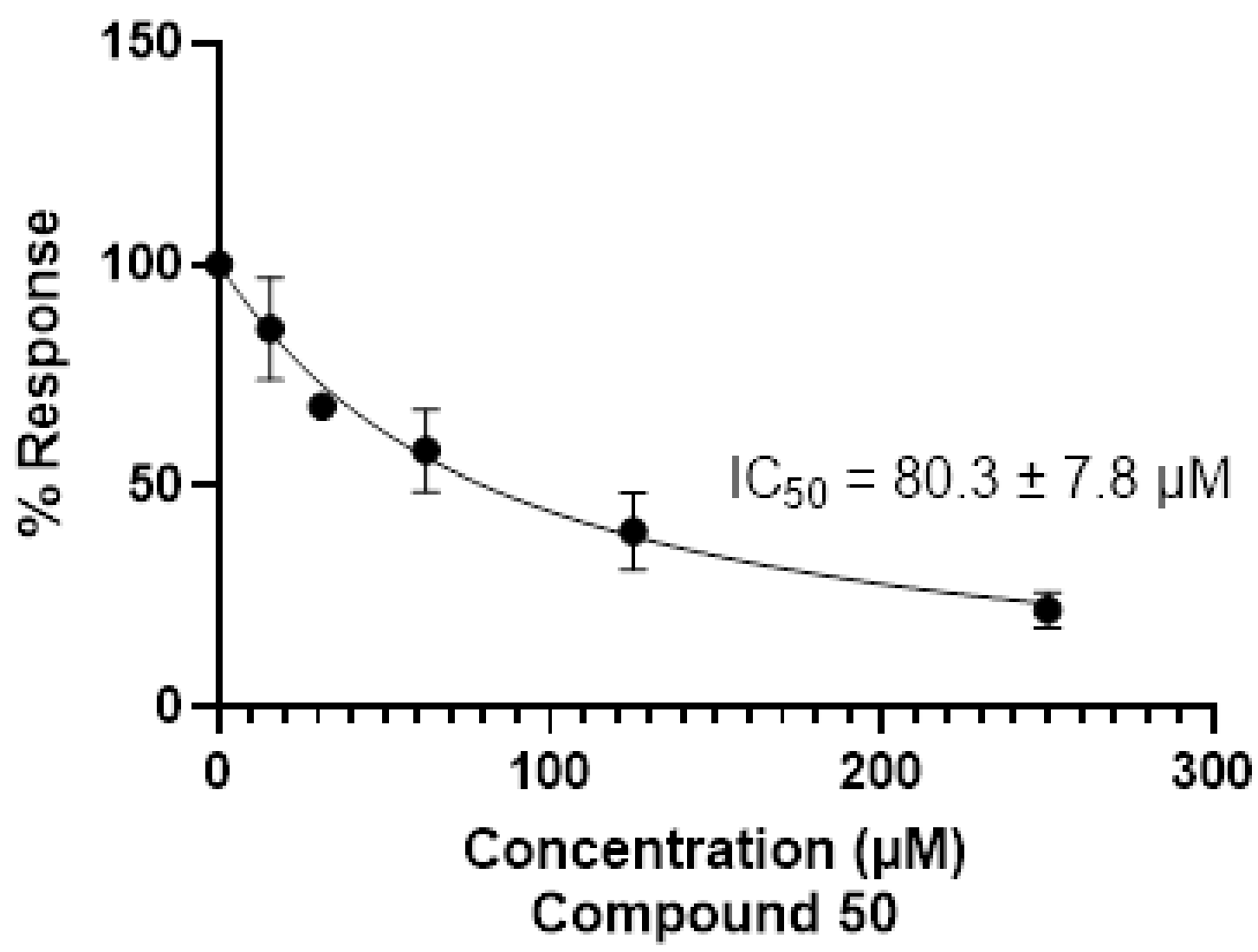

Figure S28. Binding curve of compound **50** against $VEGF_{165}$ (35 nM) for binding with biotinylated heparin.

Biolayer interferometry assay (BLI) data of ligands in complex with FGF4

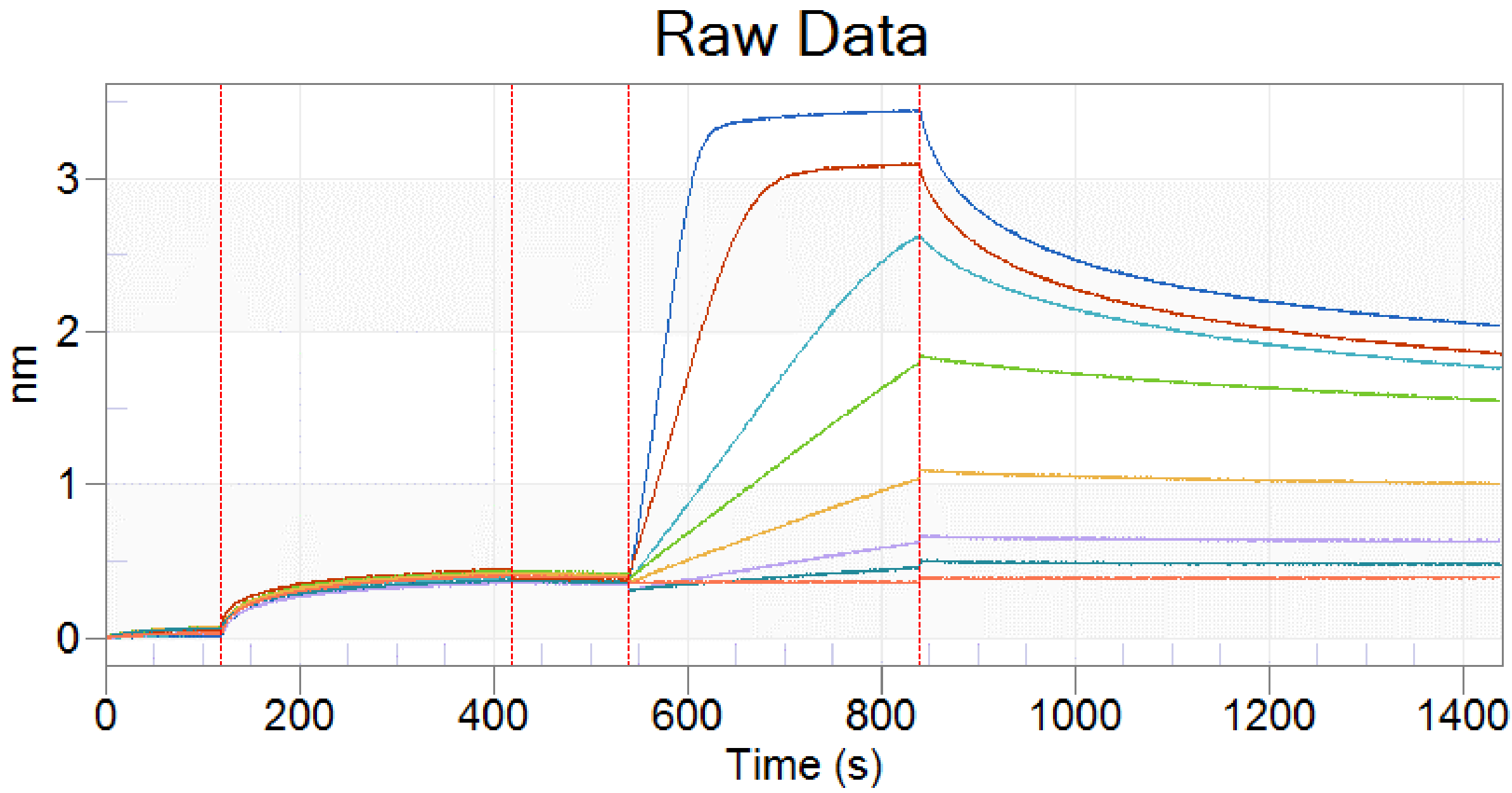

Figure S29. BLI sensorgram (raw data) of the interaction between 18 kDa biotinylated heparin (5 µg/mL) and a two-fold serial dilution of FGF4 (4.37-280 nM).

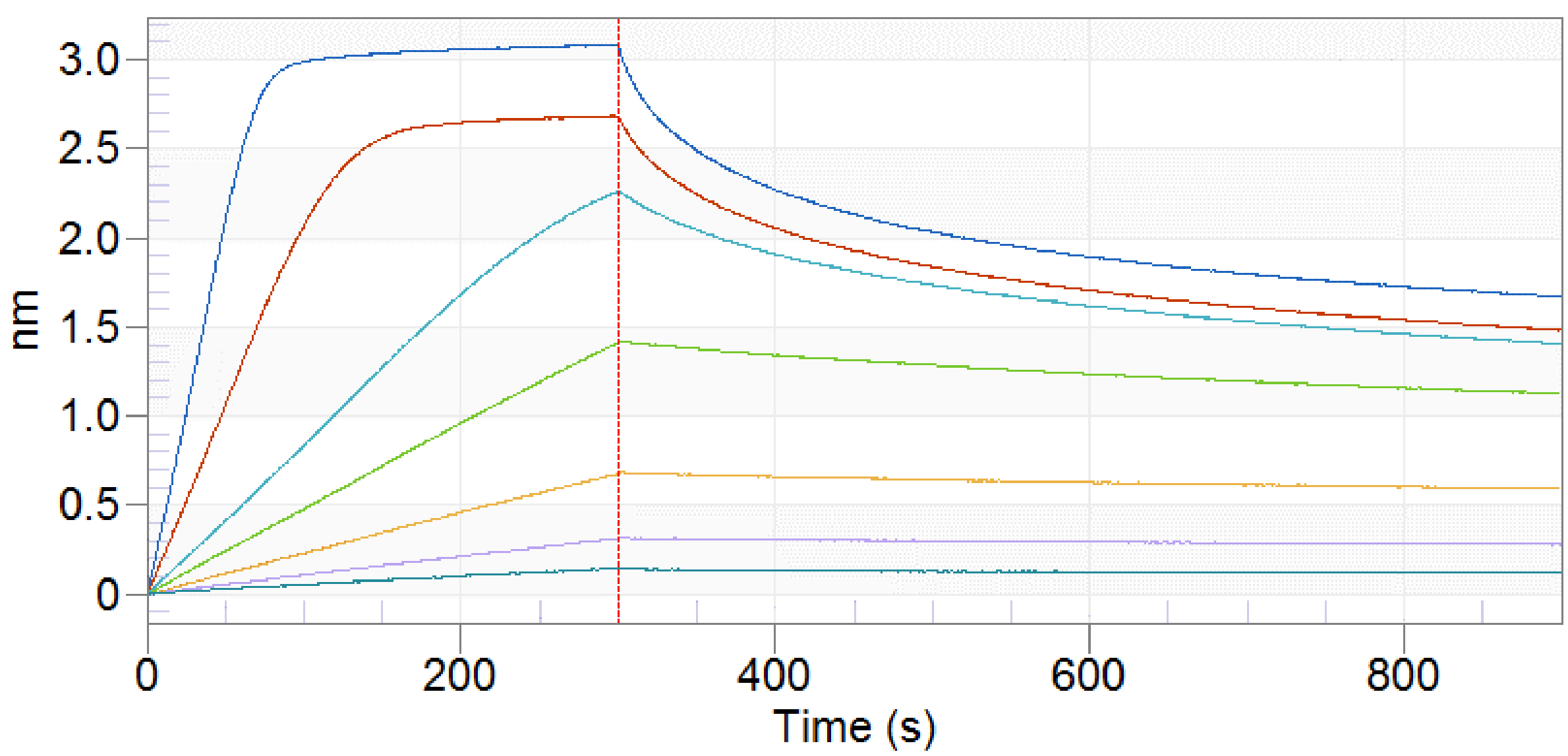

Figure S30. BLI sensorgram (processed data) of the interaction between biotinylated heparin (5 µg/mL) and a two-fold serial dilution of FGF4 (4.37-280 nM).

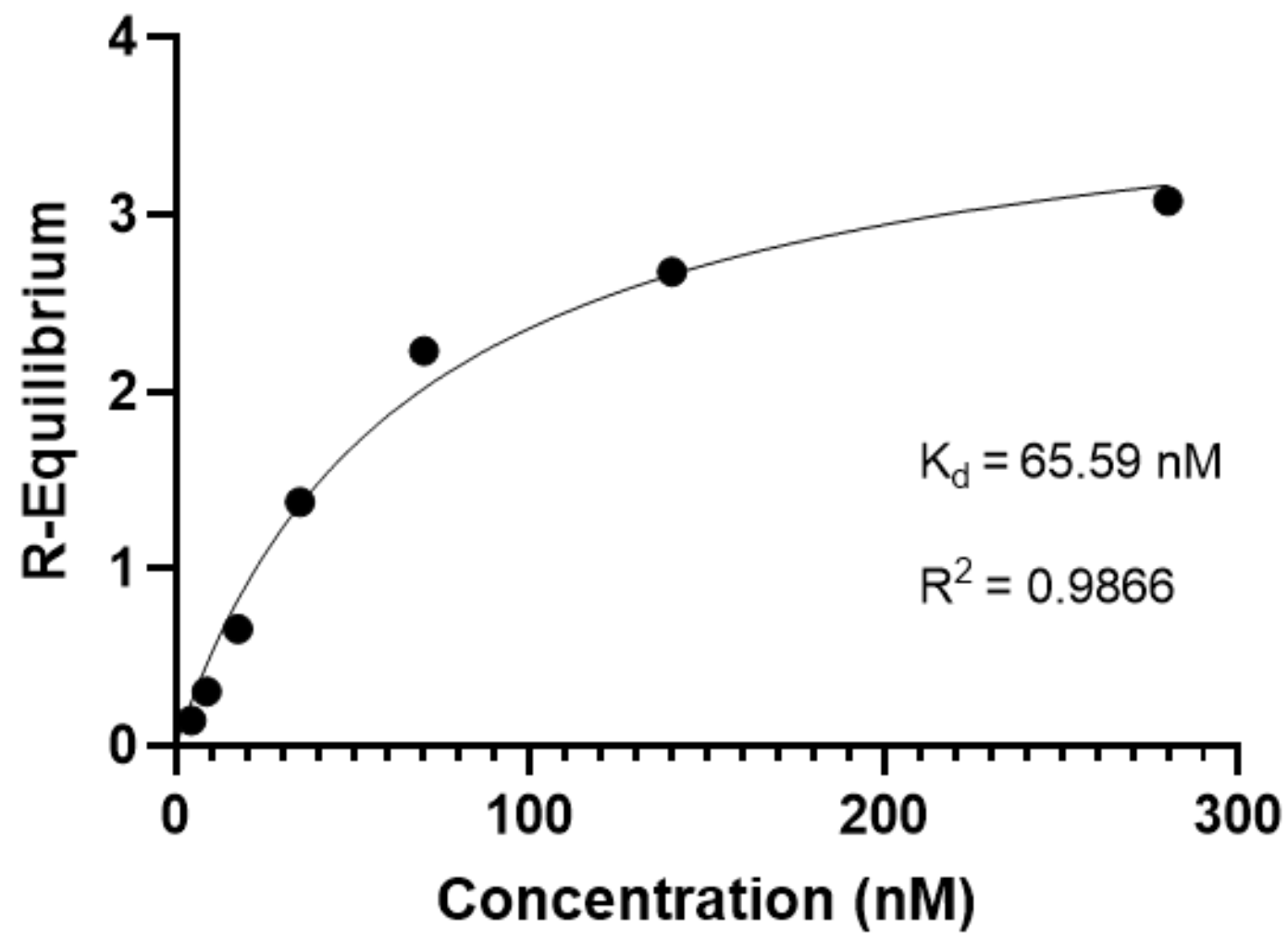

Figure S31. Binding affinity (K_d) of biotinylated heparin with FGF4

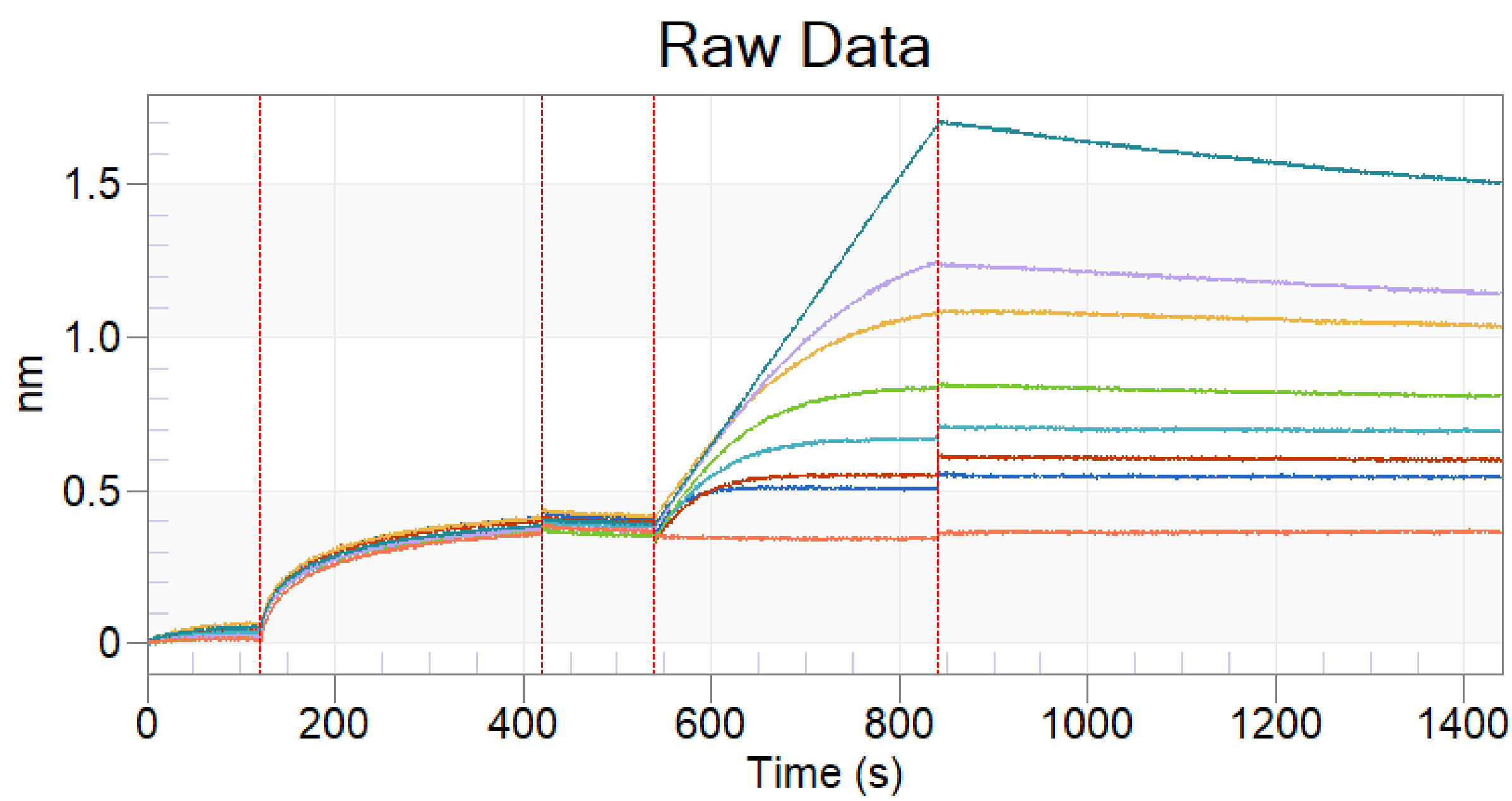

Figure S32. BLI sensorgram (raw data) of the competitive interaction between biotinylated heparin (5 µg/mL) and FGF4 (35 nM) with a two-fold serial dilution of compound **8** (7.81-250 µM).

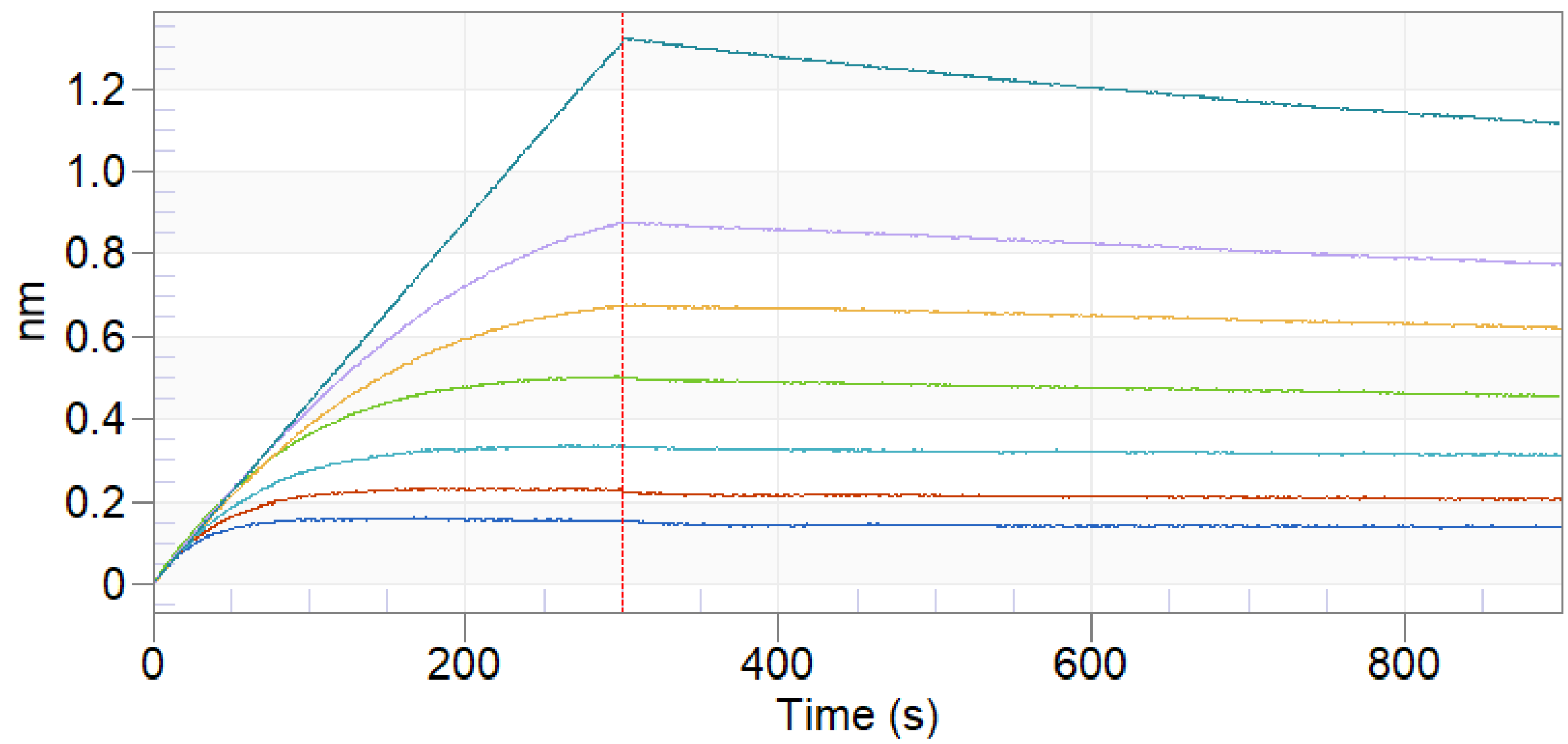

Figure S33. BLI sensorgram (processed data) of the competitive interaction between biotinylated heparin (5 µg/mL) and FGF4 (35 nM) with a two-fold serial dilution of compound **8** (7.81-250 µM).

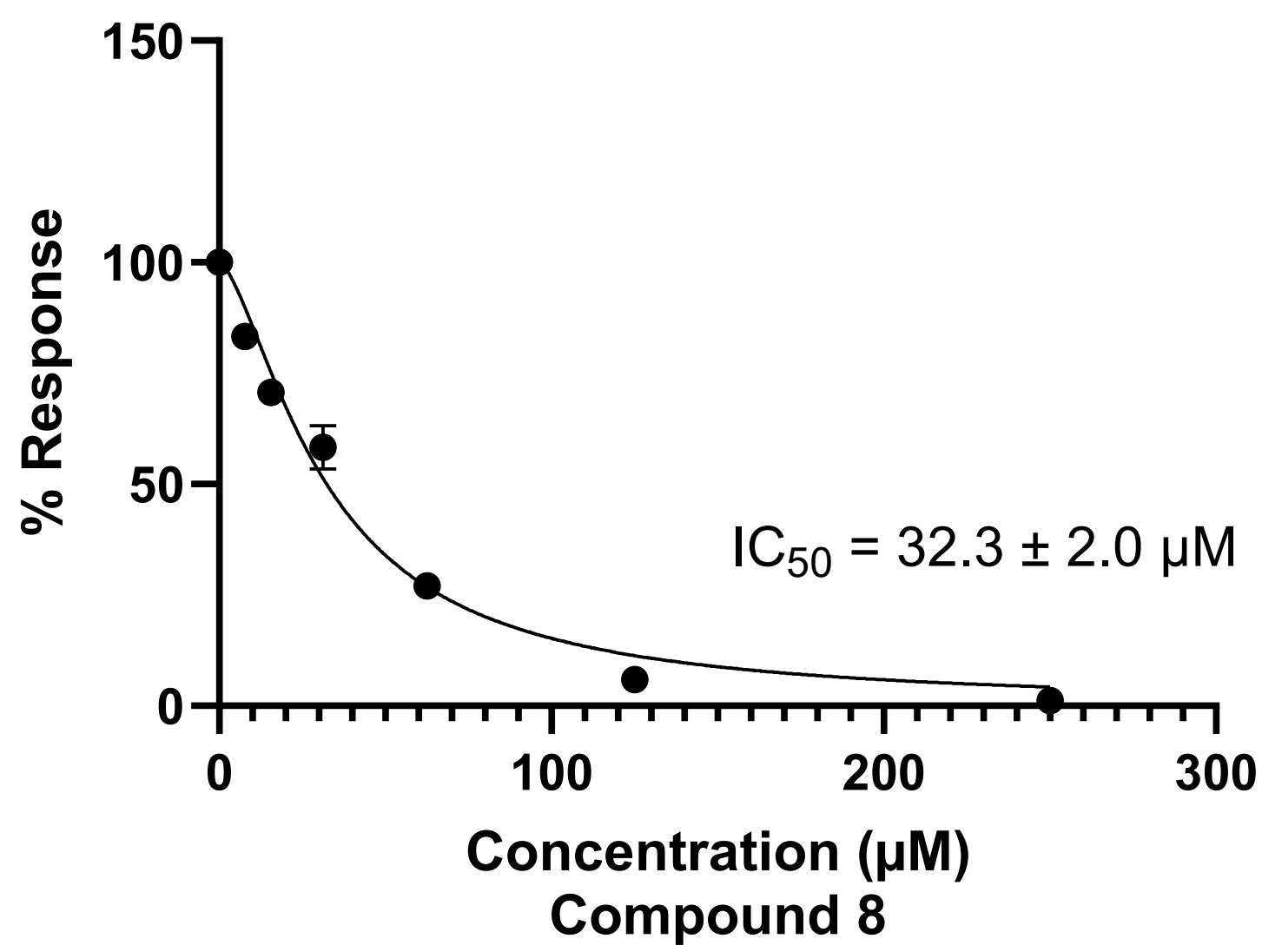

Figure S34. Inhibition activity (IC_{50}) of compound **8** against FGF4 for binding with biotinylated heparin.

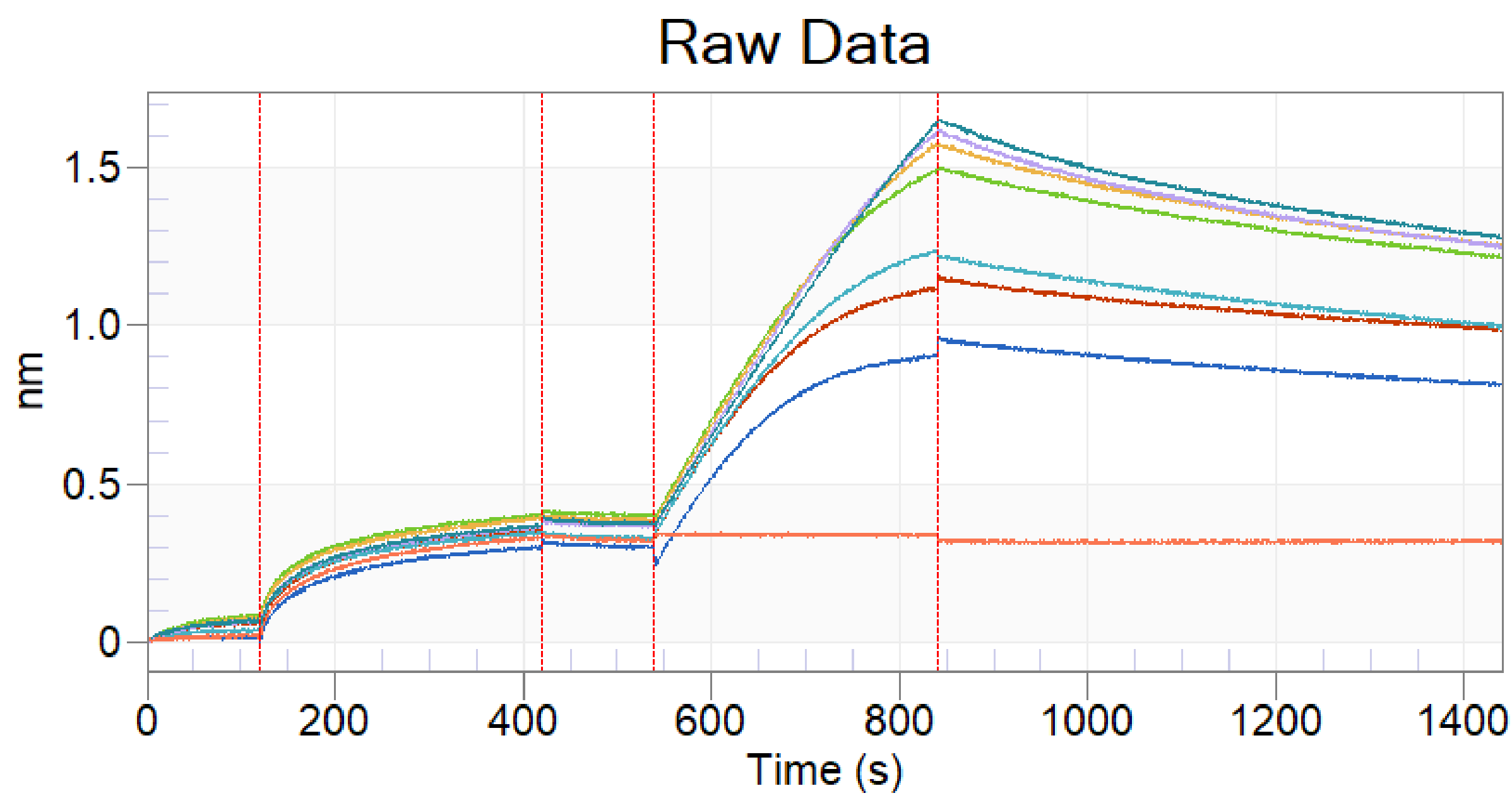

Figure S35. BLI sensorgram (raw data) of the competitive interaction between biotinylated heparin (5 µg/mL) and FGF4 (35 nM) with a two-fold serial dilution of compound **49** (15.63-500 µM).

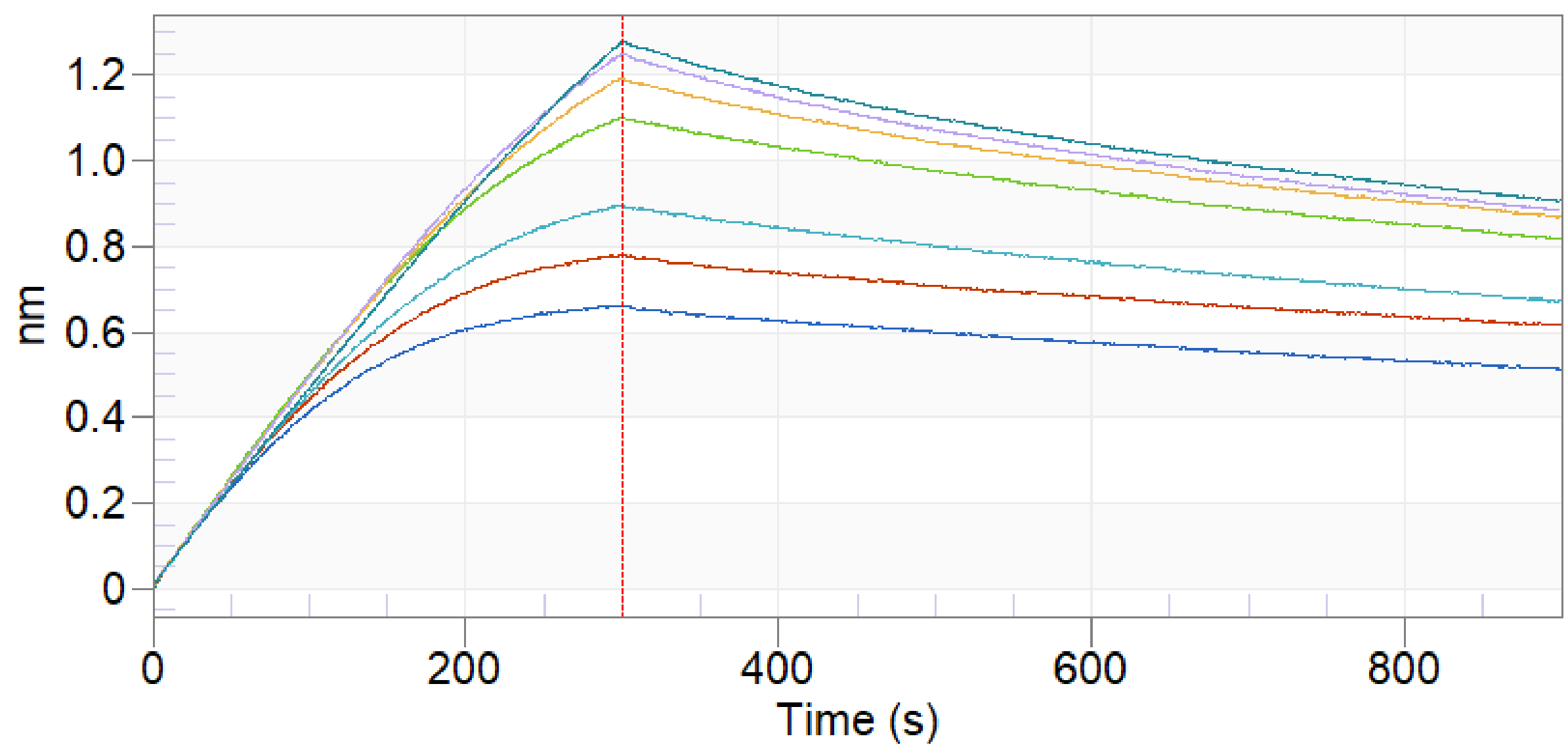

Figure S36. BLI sensorgram (processed data) of the competitive interaction between biotinylated heparin (5 µg/mL) and FGF4 (35 nM) with a two-fold serial dilution of compound **49** (15.63-500 µM).

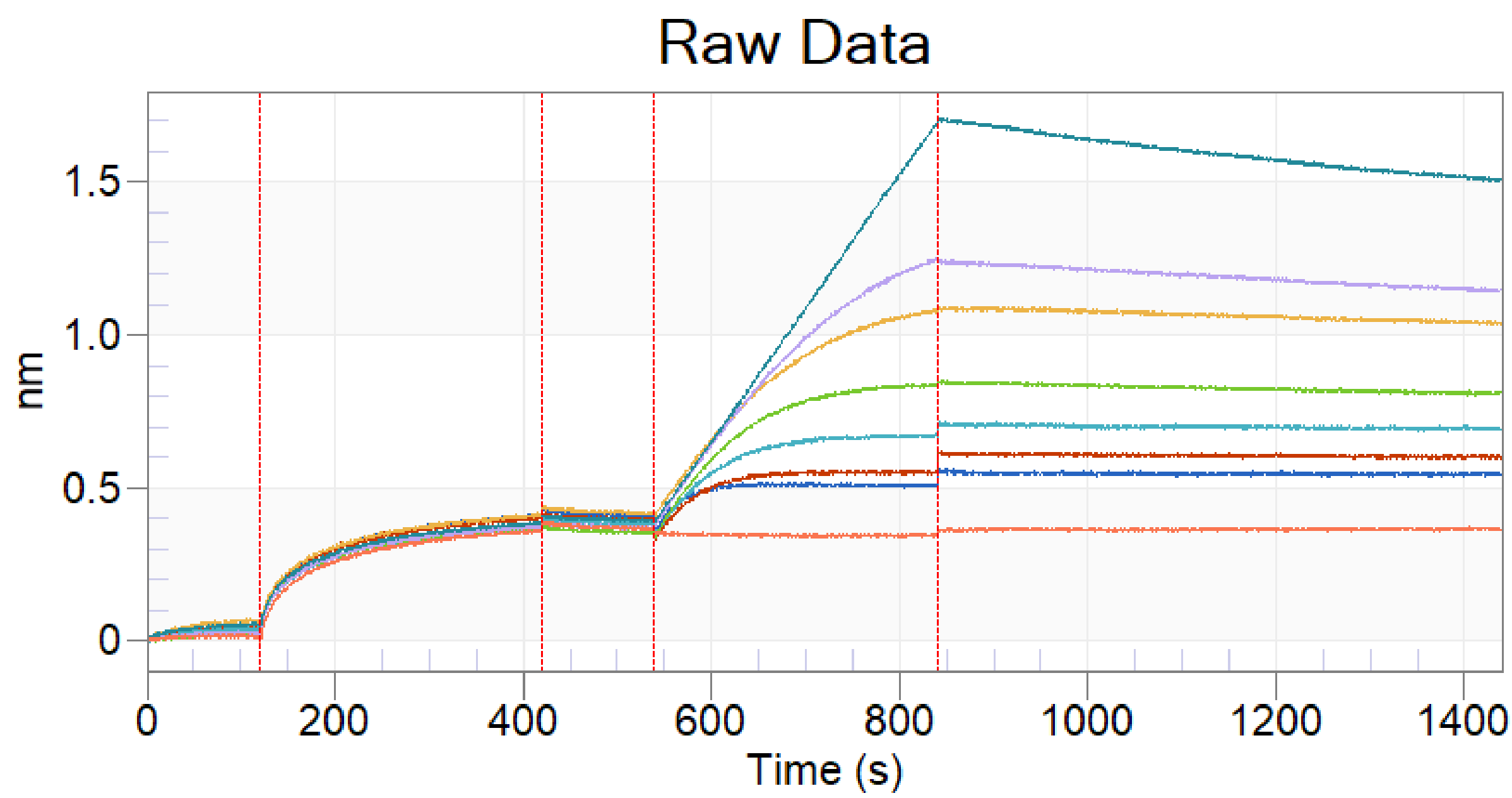

Figure S37. BLI sensorgram (raw data) of the competitive interaction between biotinylated heparin (5 µg/mL) and FGF4 (35 nM) with a two-fold serial dilution of compound **50** (3.13-100 µM).

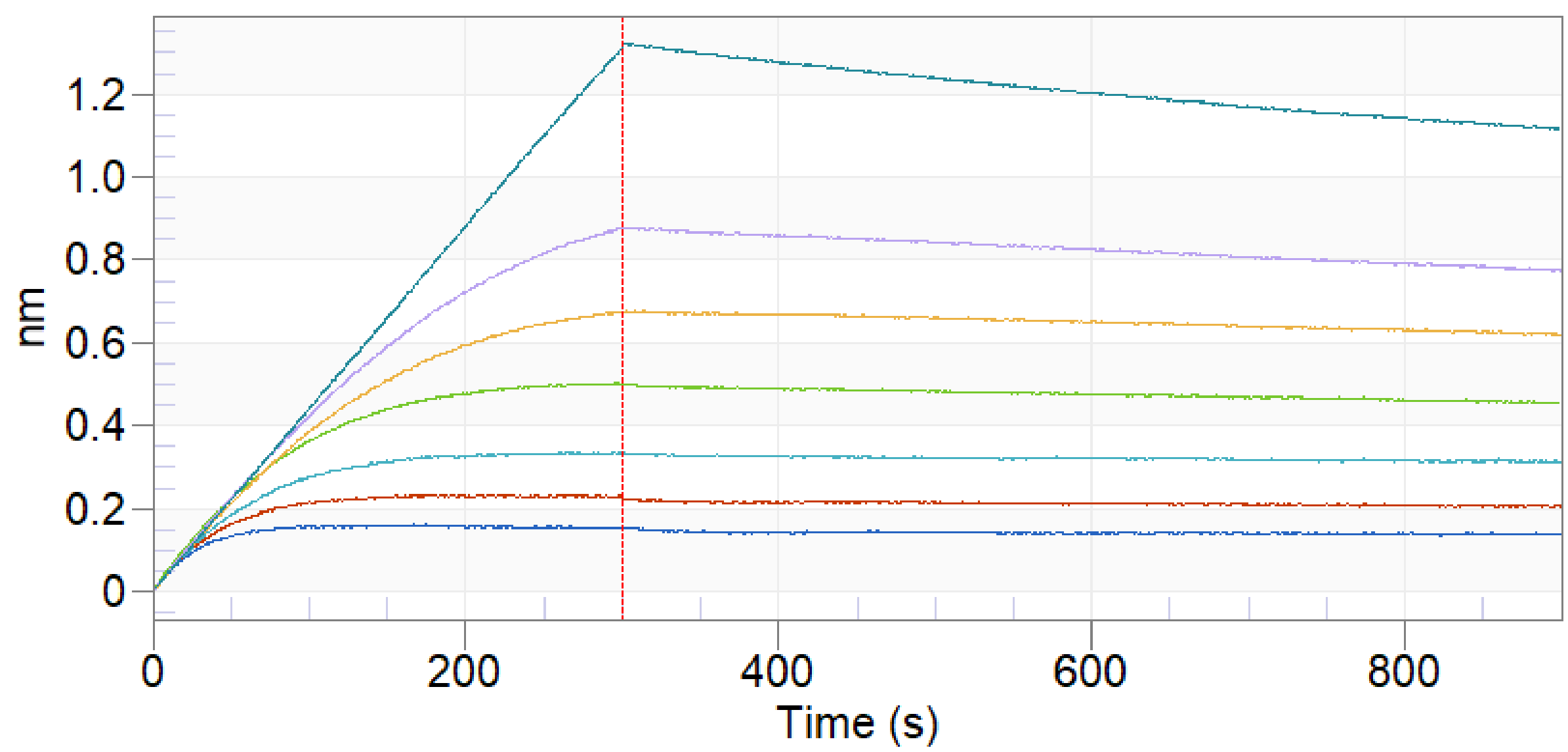

Figure S38. BLI sensorgram (processed data) of the competitive interaction between biotinylated heparin (5 µg/mL) and FGF4 (35 nM) with a two-fold serial dilution of compound **50** (3.13-100 µM).

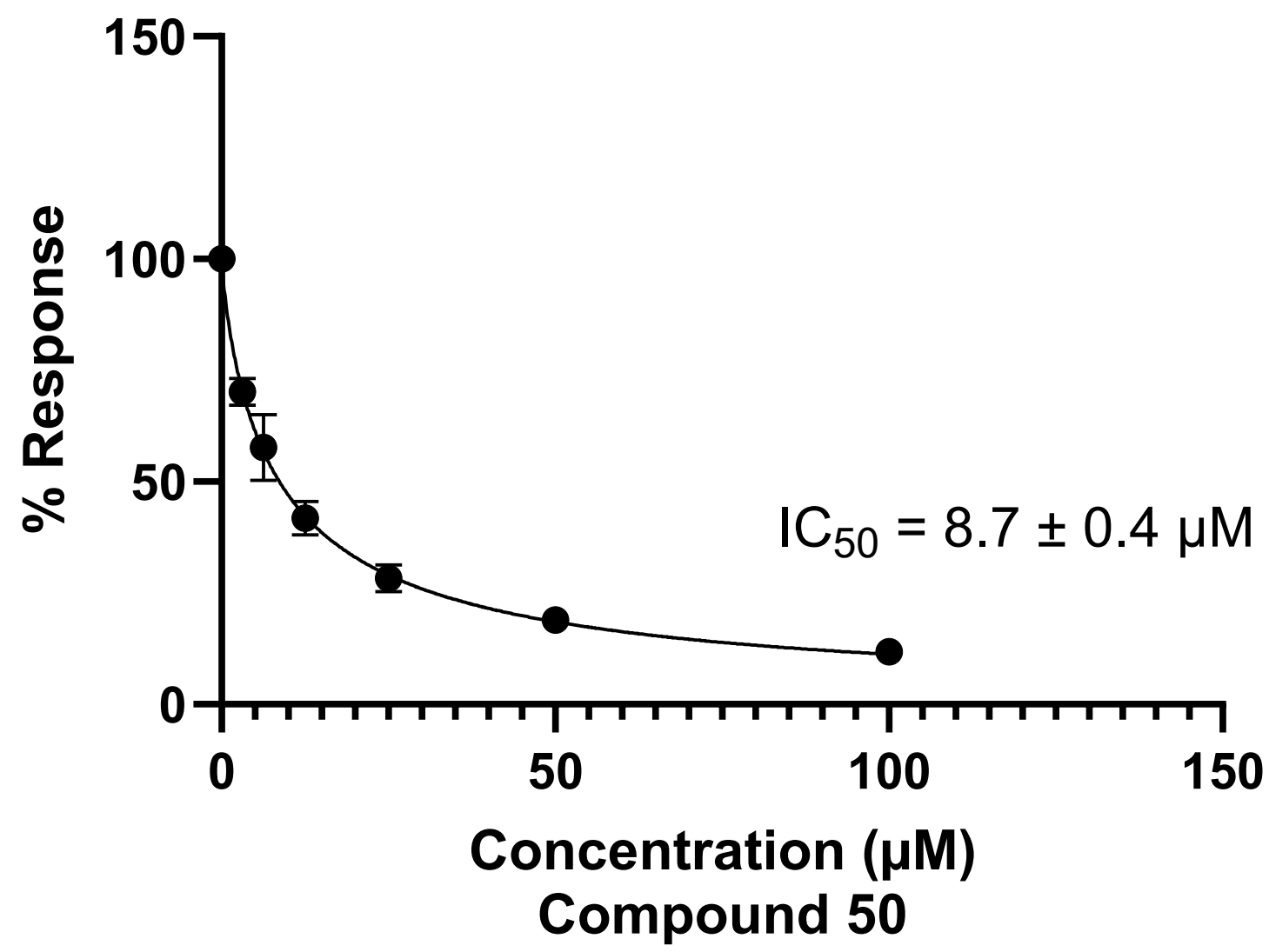

Figure S39. Inhibition activity (IC_{50}) of compound **50** against FGF4 for binding with biotinylated heparin.

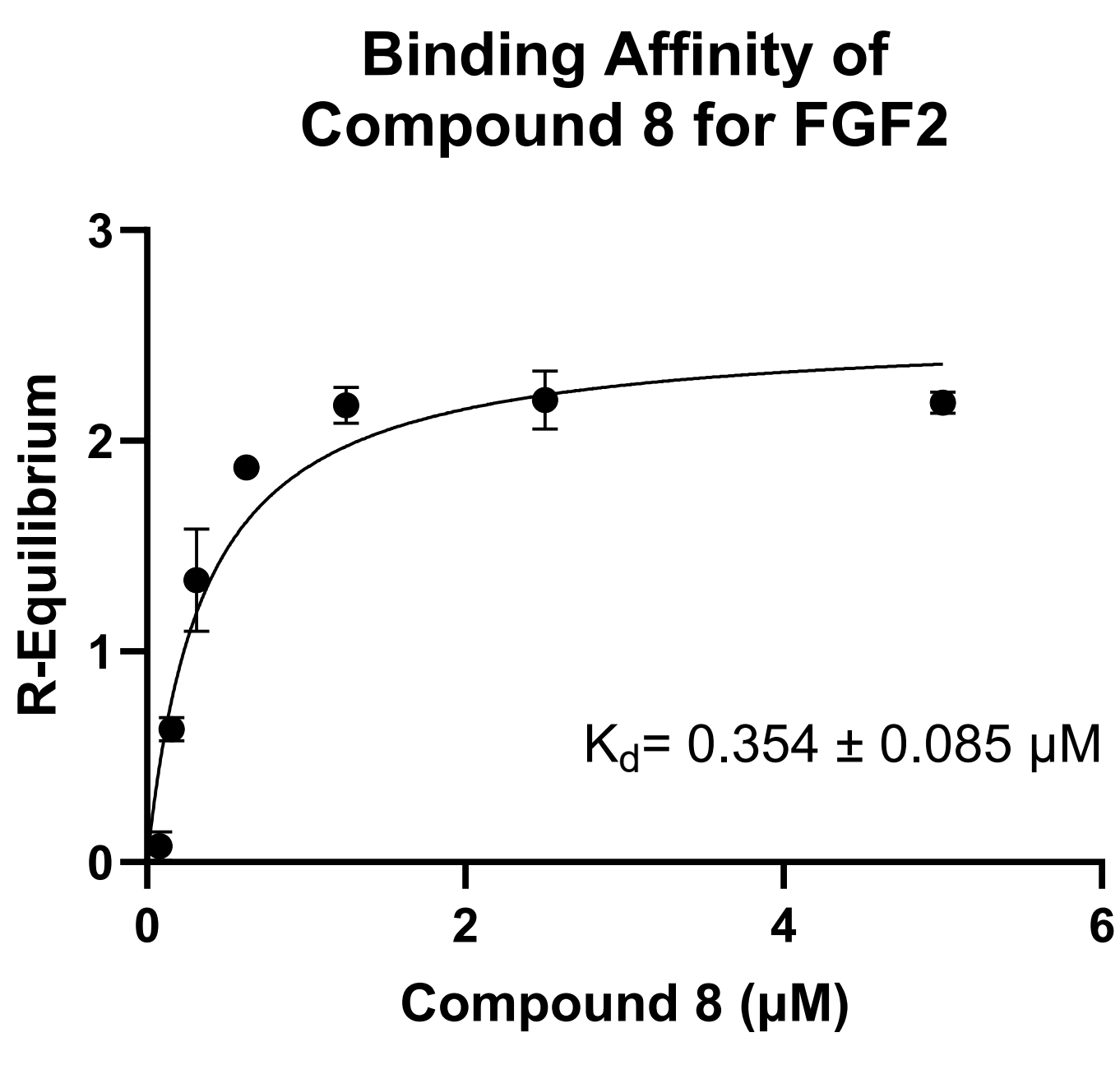

Figure S40. Kinetic binding analysis (K_d) of compound **8** to biotinylated FGF2.

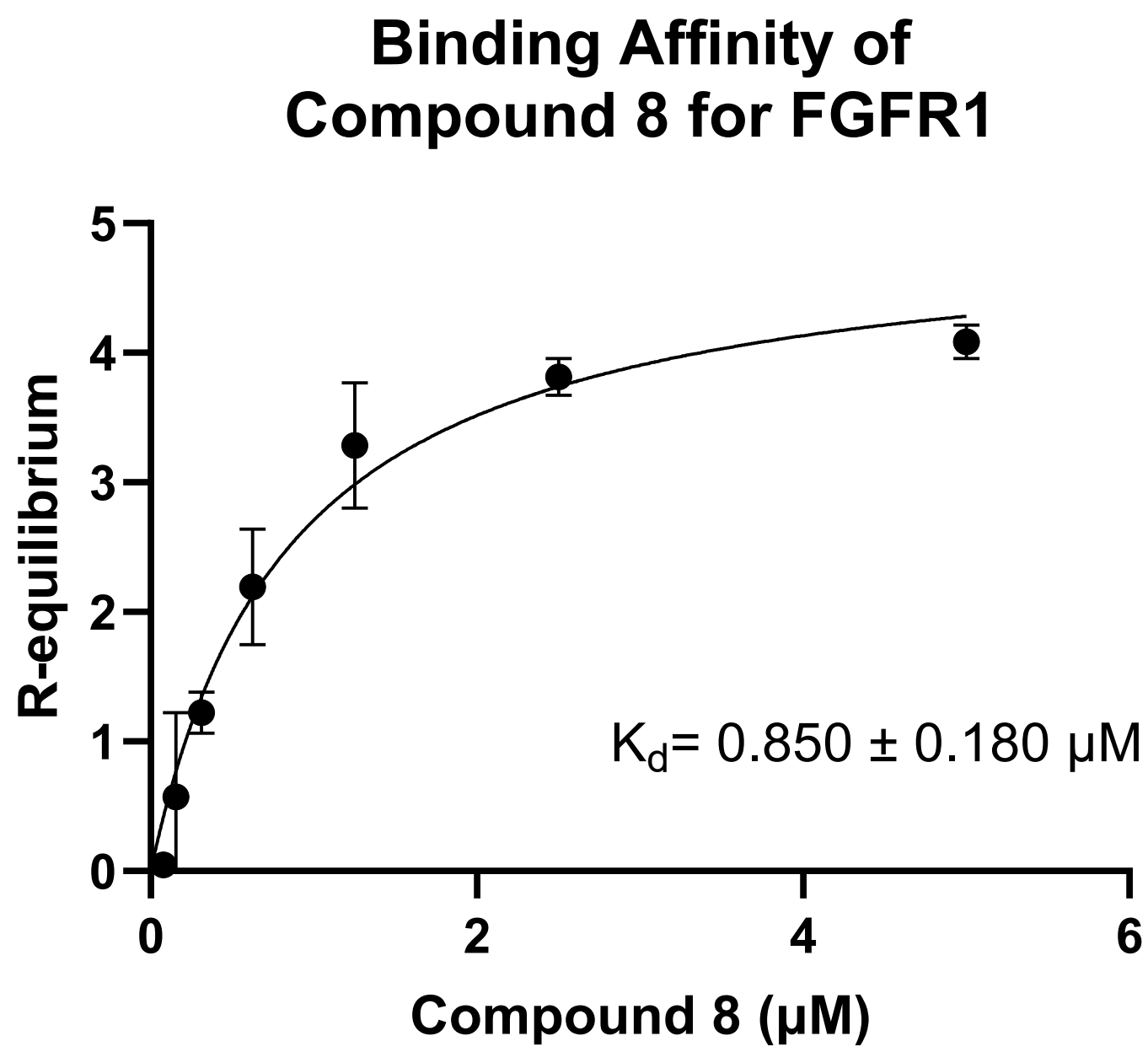

Figure S41. Kinetic binding analysis (K_d) of compound **8** to biotinylated FGFR1.

NIH3T3 Cell Culture and Functional Proliferation Assays

NIH3T3 Cell Culture: NIH3T3 fibroblast cells were cultured in Dulbecco's Modified Eagle Medium (DMEM; Gibco) supplemented with 10% fetal bovine serum (FBS) and 1% penicillin-streptomycin (100 U/mL) at 37°C in a humidified incubator with 5% CO_2. Cells were passaged at 70–80% confluence using 0.25% trypsin-EDTA and resuspended in fresh complete DMEM. For experimental assays, cells were seeded at appropriate densities and allowed to adhere overnight. Medium was replaced every 2–3 days, and cells were regularly monitored to maintain healthy morphology and prevent over confluence. For long-term storage, cells were cryopreserved in DMEM containing 10% FBS and 10% DMSO and stored in liquid nitrogen.

NIH3T3 Cell Viability Assay: NIH3T3 fibroblast cells (logarithmic growth phase) were trypsinized and counted using a hemocytometer. Cells were seeded in the inner wells of 96-well plates at a density of 5,000 cells per well in complete DMEM supplemented with 10% FBS and 1% penicillin-streptomycin. The outer wells were filled with 100 μL of sterile PBS to minimize edge effects. When cells reached approximately 50% confluence, the medium was replaced with serum-free DMEM to induce starvation for 12 hours. Subsequently, the medium was replaced with DMEM containing 2.5% FBS, and cells were treated with the indicated experimental conditions. After 24 hours of treatment, 10 μL of CCK-8 reagent was added to each

well and incubated for 2 hours at 37°C. Absorbance at 450 nm was measured using a microplate reader. Cell viability was calculated as a percentage relative to the control group using the formula:

$$\text{Cell viability (\%)} = \frac{\mathrm{OD}_{\mathrm{treatment}} - \mathrm{OD}_{\mathrm{blank}}}{\mathrm{OD}_{\mathrm{control}} - \mathrm{OD}_{\mathrm{blank}}} \times 100$$

where ODtreatment is the absorbance of the treated cells, ODControl is the absorbance of untreated control cells, and ODblank is the absorbance of wells containing medium only. All experiments were performed in triplicate.

NIH3T3 Cytotoxicity Assay: NIH3T3 fibroblast cells in the logarithmic growth phase were trypsinized and counted using a hemocytometer. Cells were seeded in the inner wells of 96-well plates at a density of 5,000 cells per well in complete DMEM supplemented with 10% fetal bovine serum (FBS) and 1% penicillin–streptomycin. To minimize edge effects, the outer wells were filled with 100 µL of sterile PBS. Cells were cultured until they reached approximately 50% confluence.

The medium was then replaced with serum-free DMEM for 12 h to induce serum starvation. Subsequently, the cells were cultured in DMEM containing 2.5% FBS and treated with compound **8** at the indicated concentrations (1, 10, 20, and 40 µM). Control wells were treated with 0.1% bovine serum albumin (BSA) in PBS. After 48 h of treatment, 10 µL of CCK-8 reagent (GlpBio, Catalog No.GK10001) was added to each well and incubated for 2 h at 37 °C.

Absorbance was measured at 450 nm using a microplate reader. Cell viability was calculated as a percentage relative to the control group using the following formula:

$$\text{Cell viability (\%)} = \frac{\mathrm{OD}_{\mathrm{C8}} - \mathrm{OD}_{\mathrm{blank}}}{\mathrm{OD}_{\mathrm{control}} - \mathrm{OD}_{\mathrm{blank}}} \times 100$$

NIH3T3 Response Assay to Exogenous FGF2: Following the cytotoxicity assay protocol, NIH3T3 cells in the logarithmic growth phase were seeded in the inner wells of 96-well plates at a density of 5,000 cells per well in complete DMEM containing 10% FBS and 1% penicillin–streptomycin. Outer wells were filled with sterile PBS to minimize edge effects. When cells reached approximately 50% confluence, The medium was then replaced with serum-free DMEM for 12 h to induce serum starvation. Subsequently, the cells were cultured in DMEM containing 2.5% FBS and treated with exogenous fibroblast growth factor 2 (FGF2) at final concentrations of 10, 20, 50, and 100 ng/mL. Control wells received 0.1% BSA in PBS. After 24 h of treatment, 10 µL of CCK-8 reagent was added to each well and incubated for 2 h at 37 °C. Absorbance was

measured at 450 nm, and cell viability was calculated relative to the control group using the formula described above. All experiments were performed in triplicate.

NIH3T3 Response Assay to Exogenous FGF2 in the Presence of Compound 8: NIH3T3 cells were seeded and cultured following the same protocol as described for NIH3T3 Cell Viability Assay. After reaching approximately 50% confluence, the medium was then replaced with serum-free DMEM for 12 h to induce serum starvation. Subsequently, the cells were cultured in DMEM containing 2.5% FBS and treated with. Cells were then treated under the following conditions: (1) 20 ng/mL FGF2 alone, and (2) 20 ng/mL FGF2 in combination with **8** at final concentrations of 0.5, 10, and 20 μM. Control wells were treated with 0.1% bovine serum albumin (BSA) in PBS. After 24 h of treatment, 10 μL of CCK-8 reagent was added to each well, and the wells were incubated for 2 h at 37 °C.

Statistical Analysis: All experiments were performed in at least three independent biological replicates. Data are presented as mean ± standard error of the mean (SEM). Statistical comparisons between groups were performed using one-way analysis of variance (ANOVA) followed by Tukey's multiple comparisons test. Differences were considered statistically significant when $p < 0.05$. GraphPad Prism 10 (GraphPad Software, USA) was used for all analyses and figure generation.

Group A (*N*-sulfated ligands)

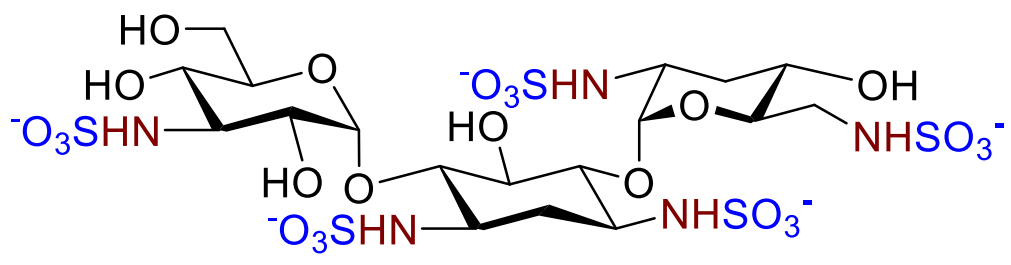

1

FGF2 (6.30 ± 0.80 μM)
FGFR1 (5.27 ± 0.70 μM)

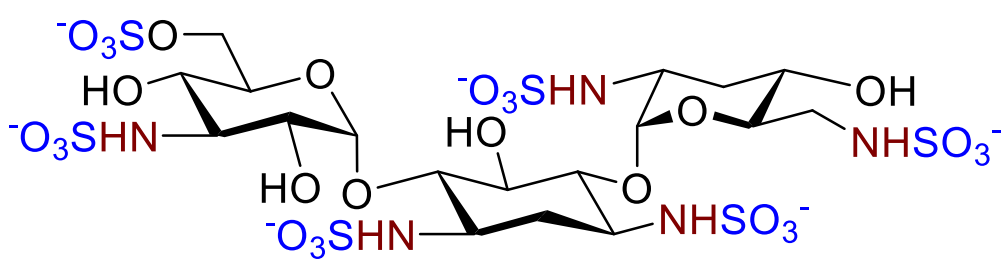

2

FGF2 (26.6 ± 1.43 μM)

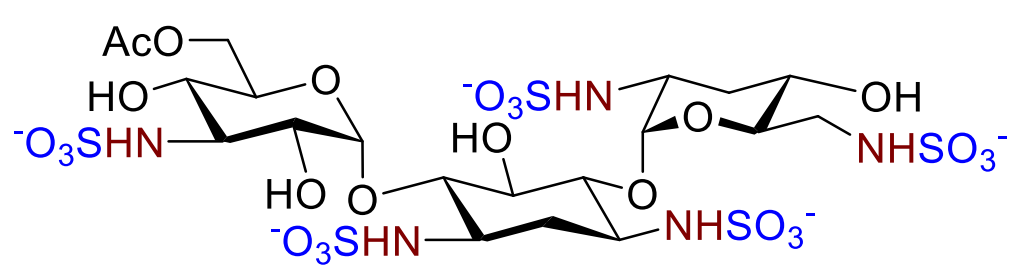

3

FGF2 (7.24 ± 0.86 μM)

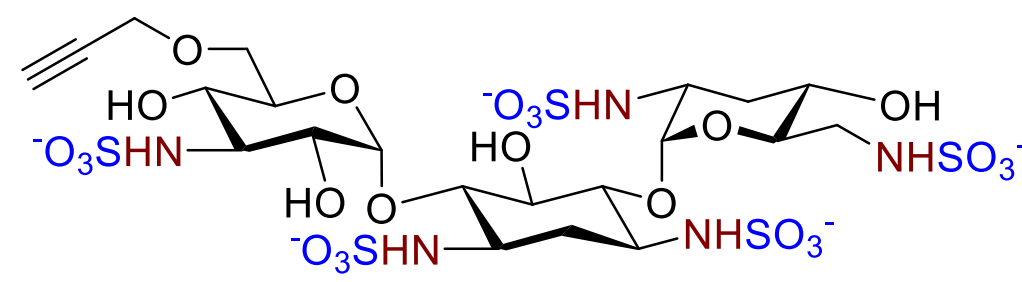

4

FGF2 (9.64 ± 0.88 μM)

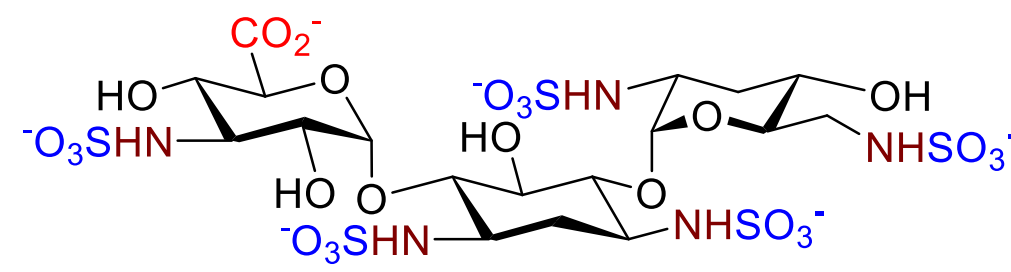

5

FGF2 (11.18 ± 1.05 μM)

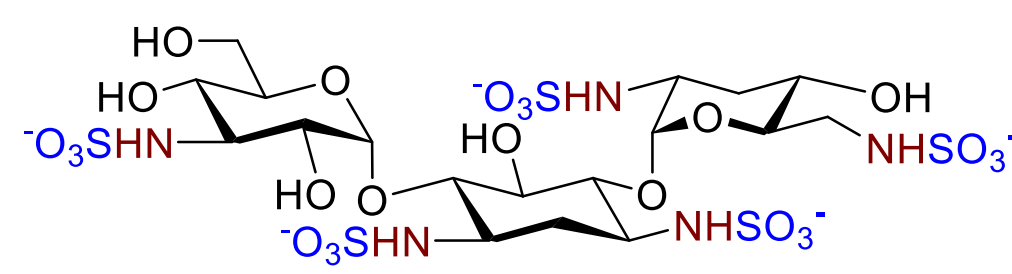

6

FGF2 (11.4 ± 0.057 μM)

Figure S42. IC_{50} values of Group A Ligands with Na^+ as a counterion. Data are shown as mean ± SEM (n=3)

Group B (*O*-sulfated ligands)

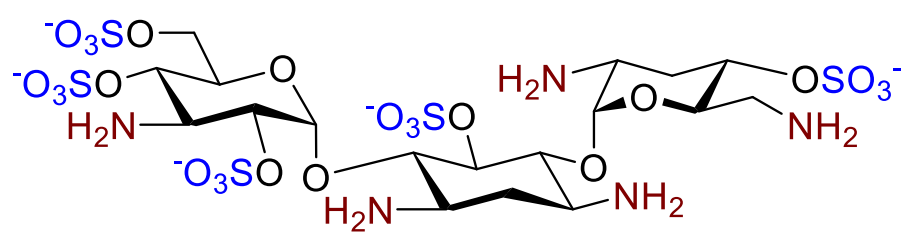

7

FGF2 (33 ± 1.5 μM)

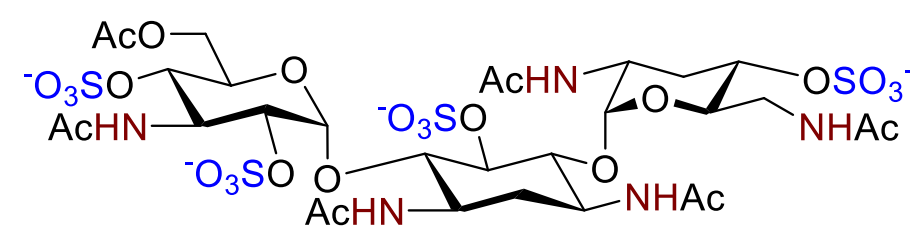

9

FGF2 (9.86 ± 0.99 μM)

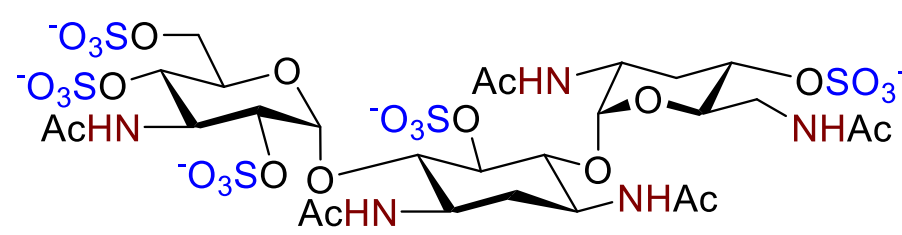

8

FGF2 (0.23 ± 0.06 μM)
FGFR1 (0.17 ± 0.06 μM)
VEGF165 (0.66 ± 0.01 μM)
BioFGF2-FGFR1 (0.13 ± 0.01 μM)

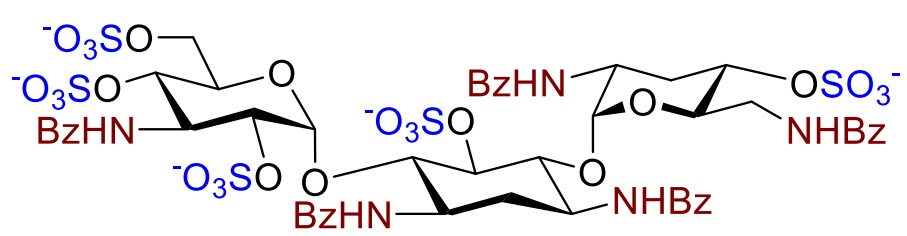

10

FGF2 (70 ± 2.0 μM)

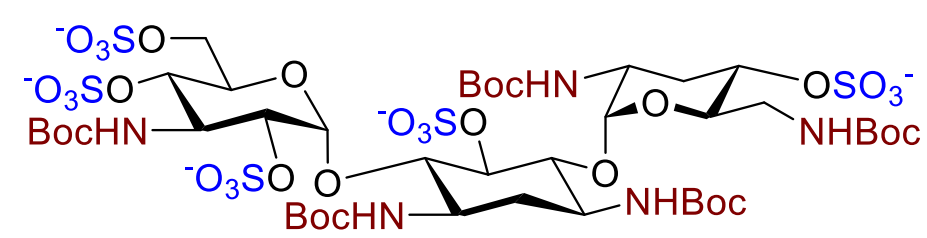

12

FGF2 (34.4 ± 1.5 μM)

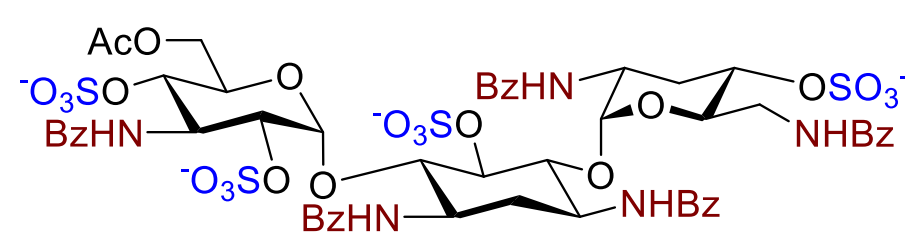

11

FGF2 (1.12 ± 0.05 μM)

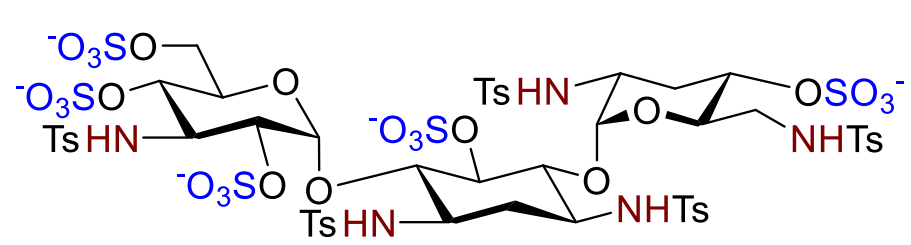

13

FGF2 (46.43 ± 1.67 μM)

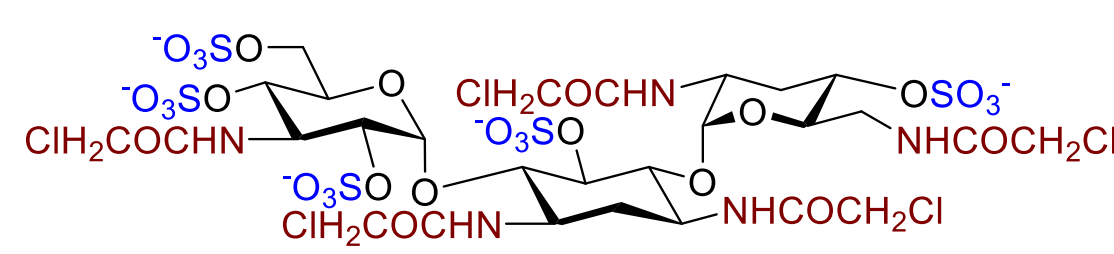

15

FGF2 (28 ± 1.45 μM)

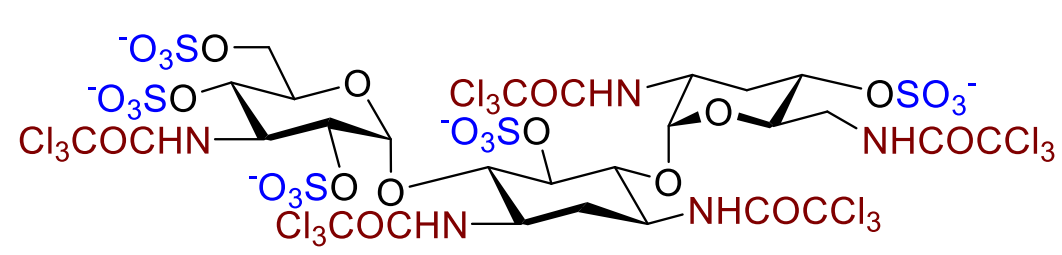

14

FGF2 (49.15±1.69 μM)

Figure S43. IC_{50} values of Group B Ligands with Na^+ as a counterion. Data are shown as mean ± SEM (n=3)

Group C (Hydrophobic aryl and aliphatic ligands)

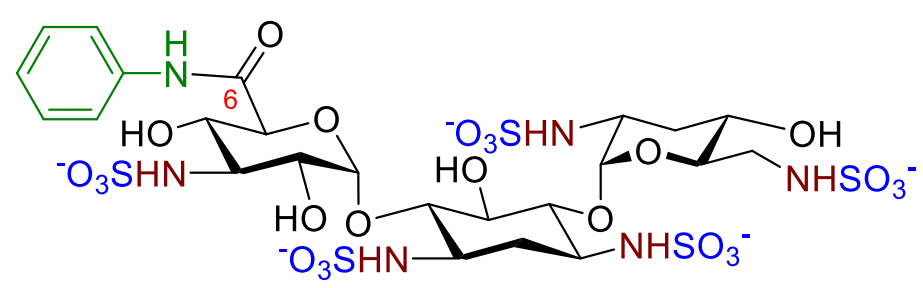

16

FGF2 (9.7 ± 0.99 μM)

FGF2 (2.21 ± 0.35 μM)

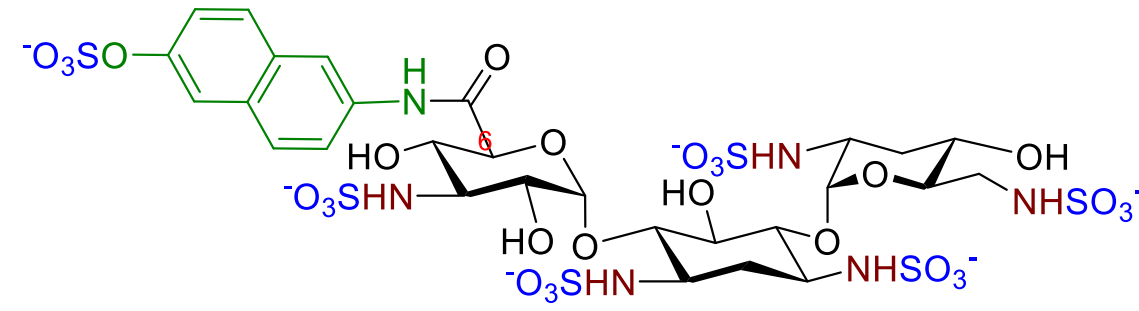

21

FGF2 (77 ± 1.9 μM)

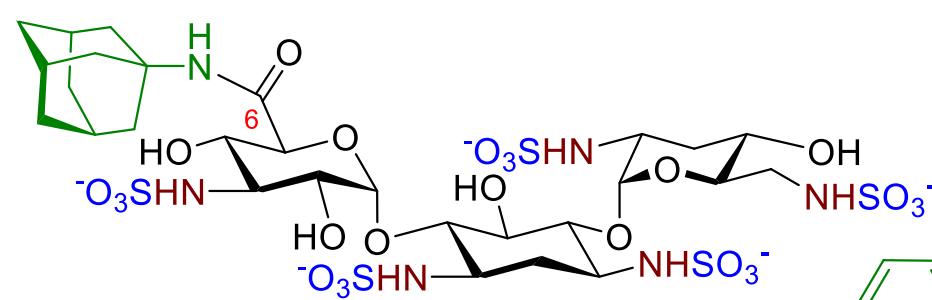

22

FGF2 (3.02 ± 0.48 μM)

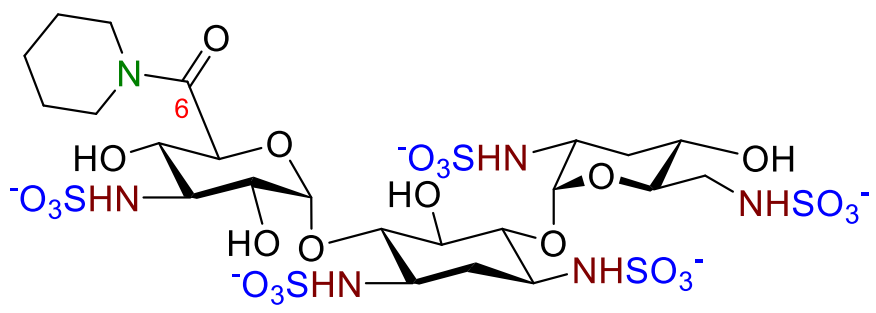

23

FGF2 (9.3 ± 0.97 μM)

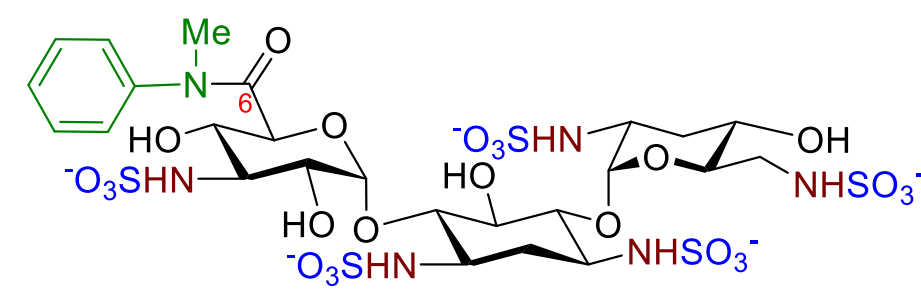

20

FGF2 (5.85 ± 0.77 μM)

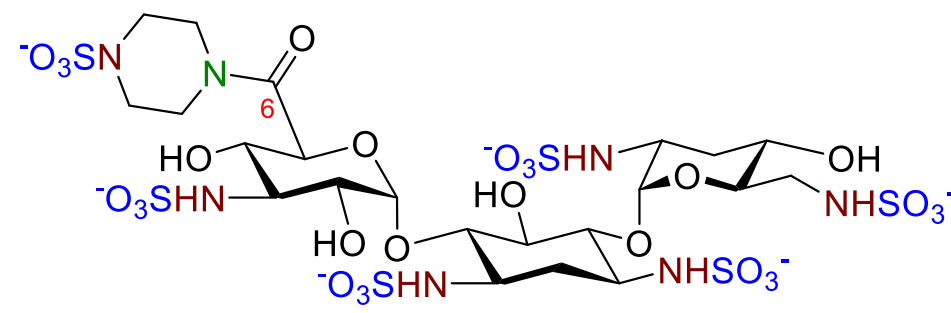

25

FGF2 (725 ±3 μM)

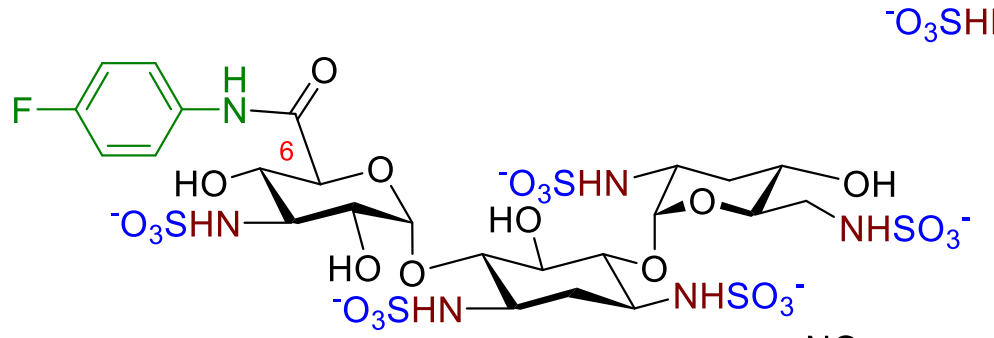

17

FGF2 (56 ± 1.8 μM)

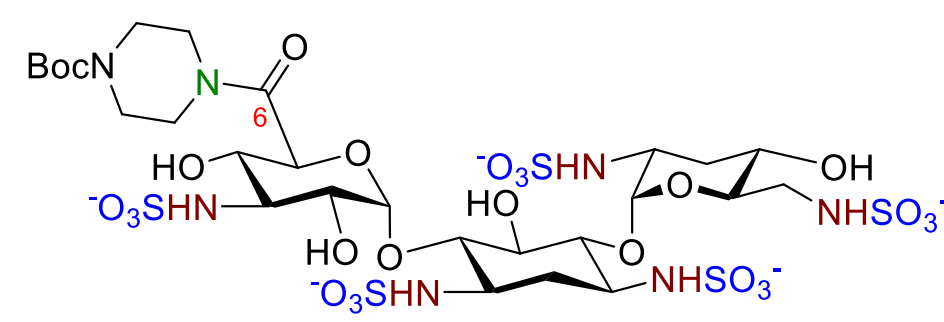

27

FGF2 (68 ± 1.83 μM)

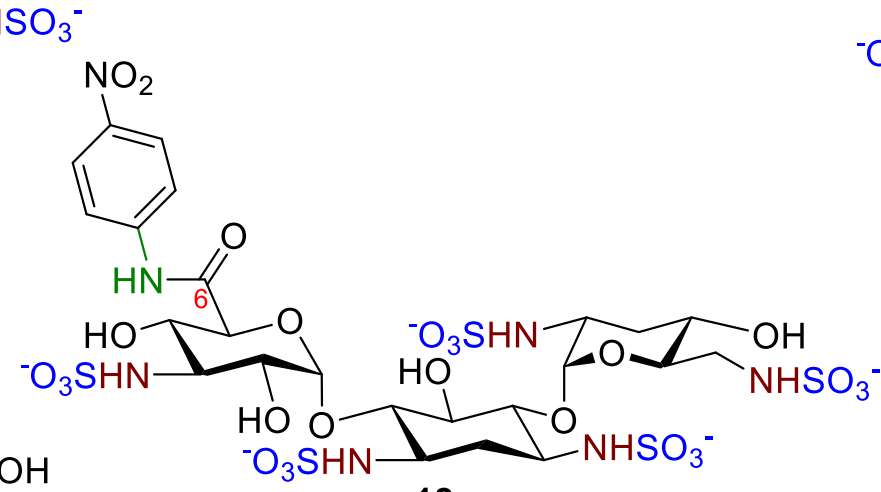

18

FGF2 (23 ± 1.4 μM)

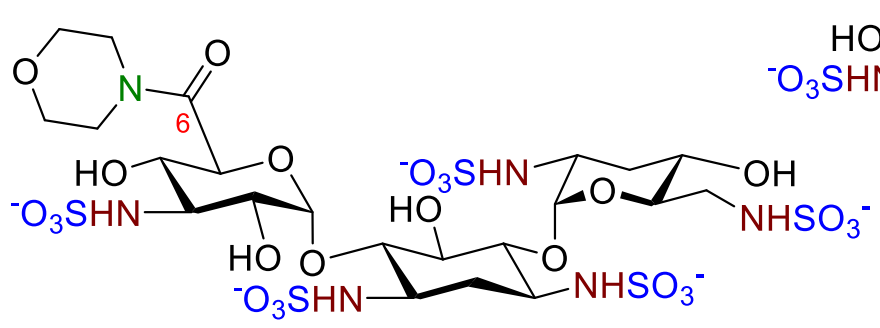

24

FGF2 (130 ± 2.12 μM)

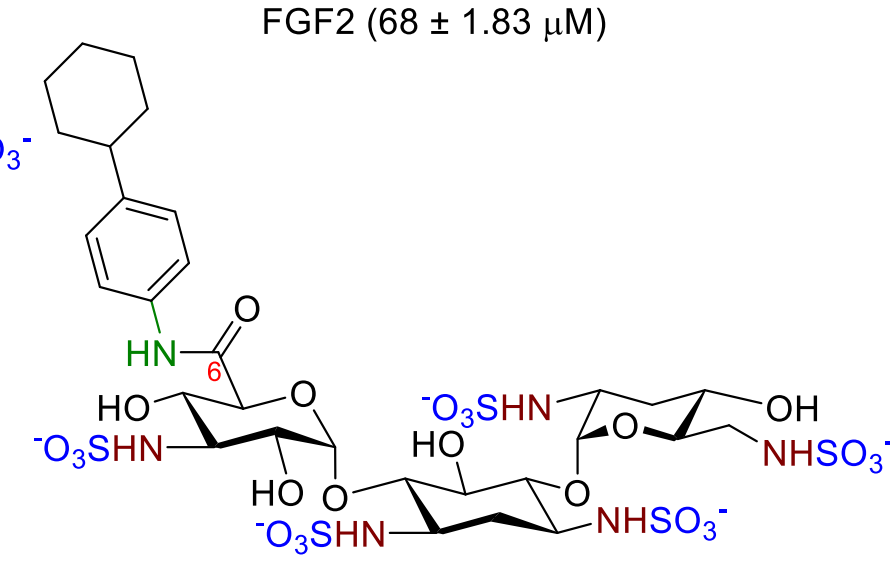

19

FGF2 (1.25 ± 0.11 μM)

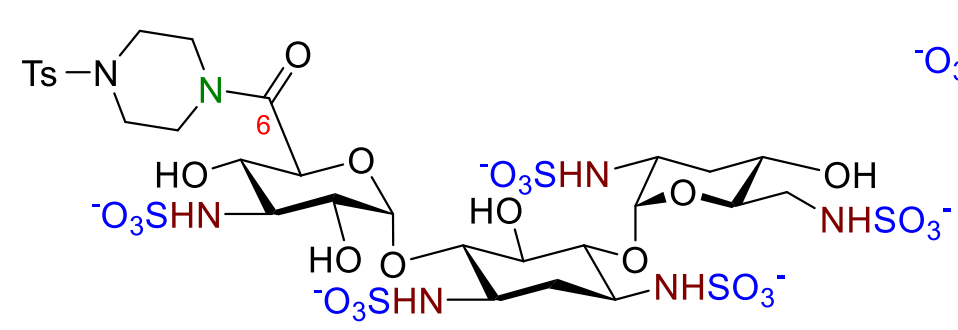

26

FGF2 (70 ± 1.8 μM)

Figure S44. IC_{50} values of Group C Ligands with Na^+ as a counterion. Data are shown as mean ± SEM (n=3)

Group D (Naphthalene ligands)

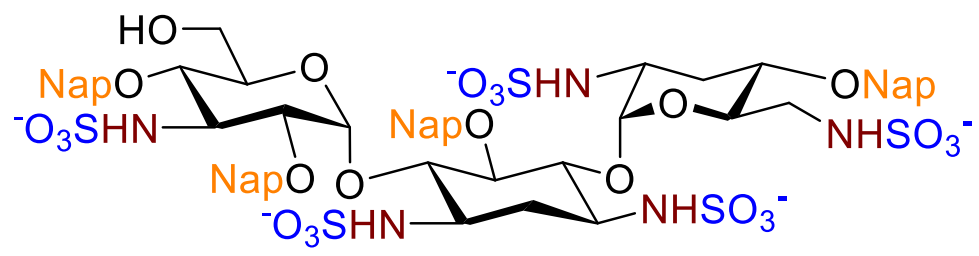

28
FGF2 (2.6 ± 0.41 μM)

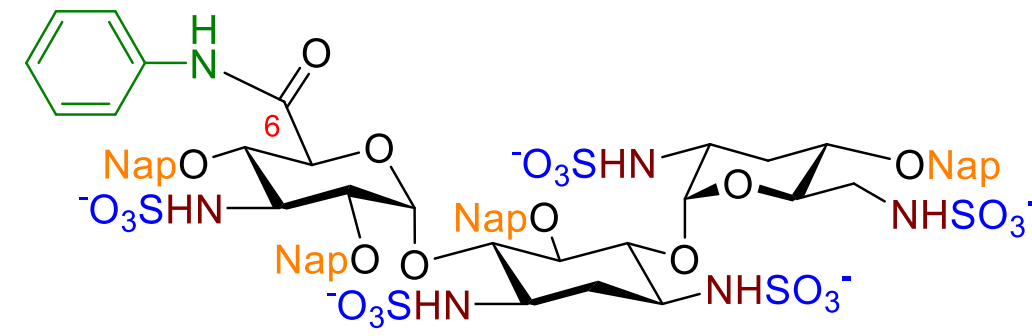

33
FGF2 (1.9 ± 0.29 μM)

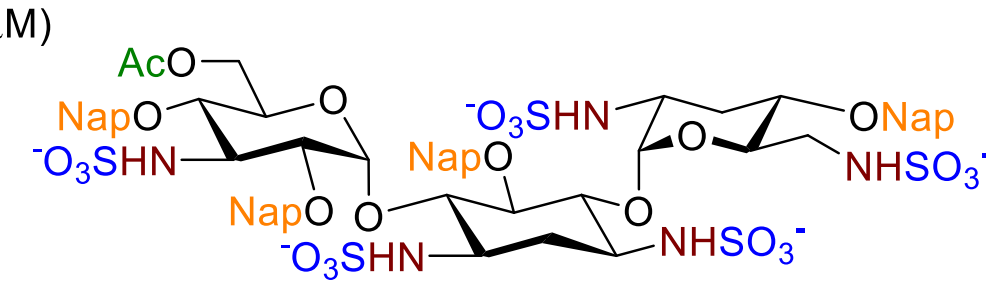

29
FGF2 (5.86 ± 0.77 μM)

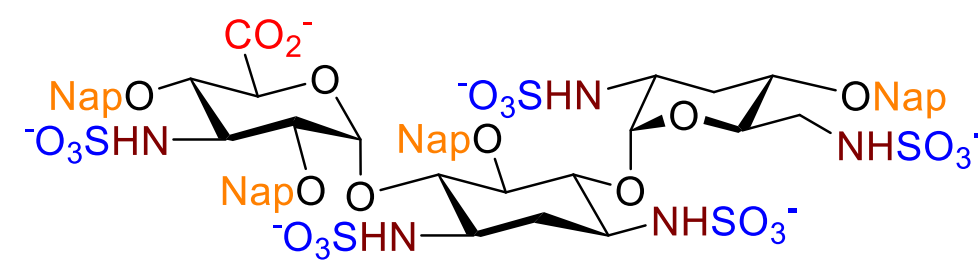

31
FGF2 (4.9 ± 0.69 μM)

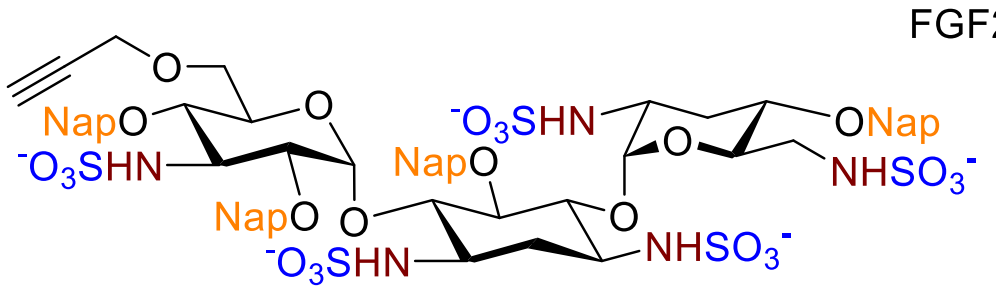

30
FGF2 (0.80 ± 0.09 μM)

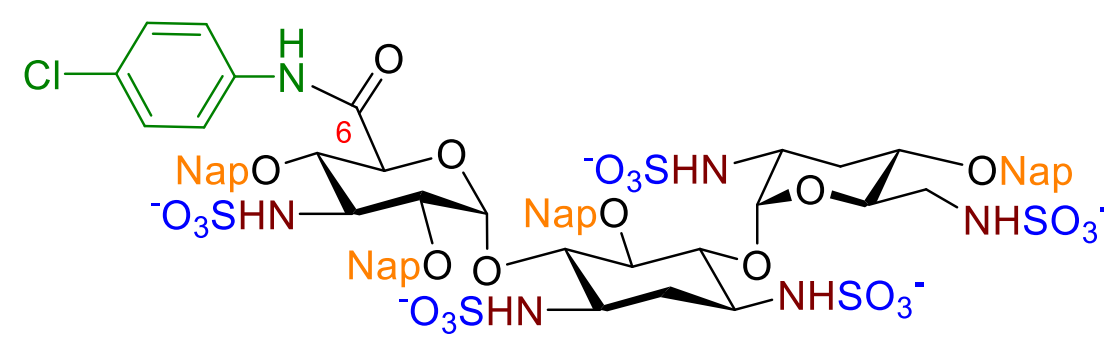

34
FGF2 (1.6 ± 0.19 μM)

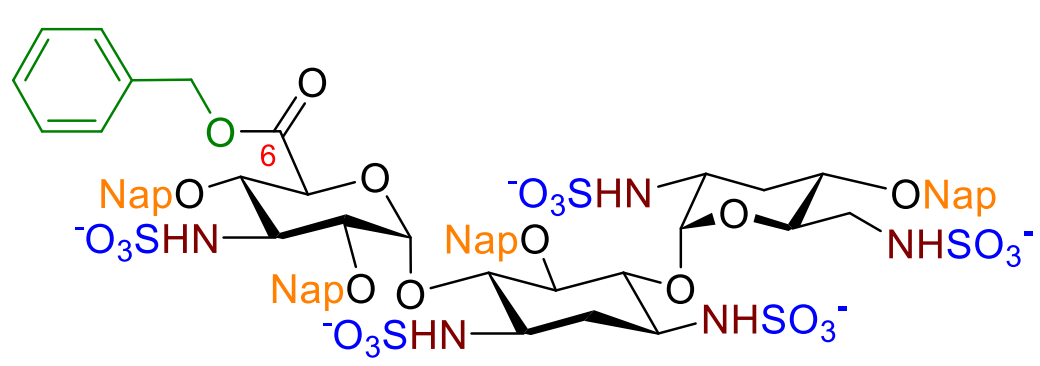

35
FGF2 (0.69 ± 0.03 μM)

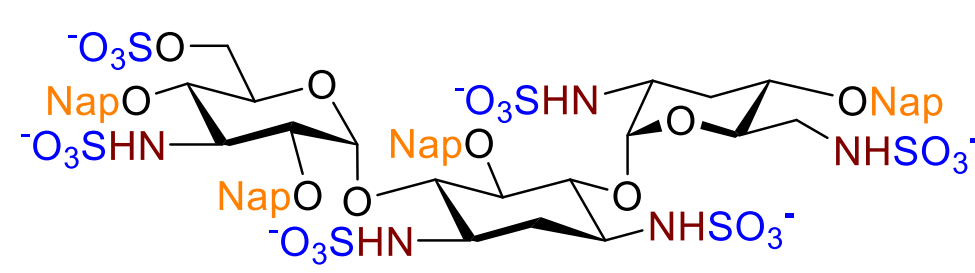

32
FGF2 (10.4 ± 1.02 μM)

Figure S45. IC_{50} values of Group D Ligands with Na^+ as a counterion. Data are shown as mean ± SEM (n=3)

Group E (Oleanolic acid ligands)

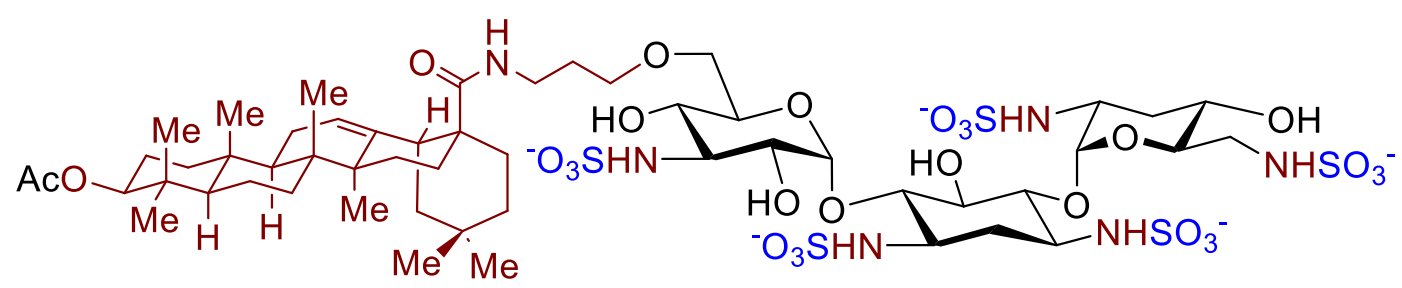

38

FGF2 (37.6 ± 1.6 μM)

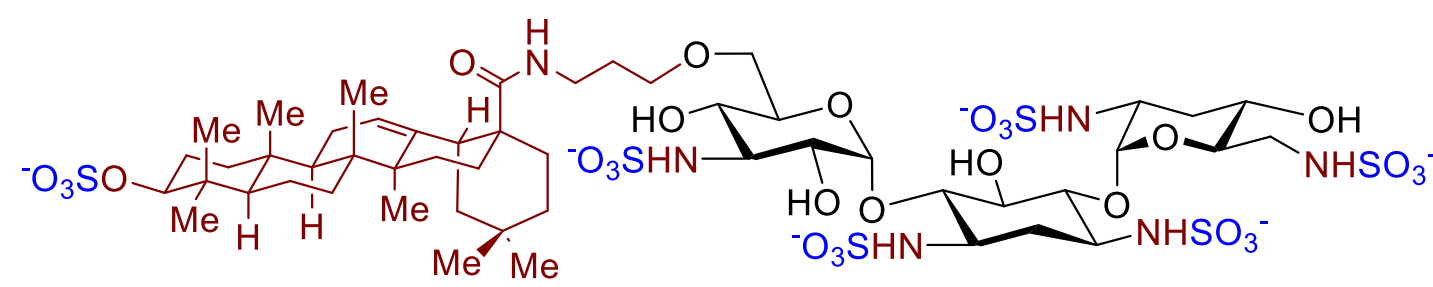

39

FGF2 (10.8 ± 1.03 μM)

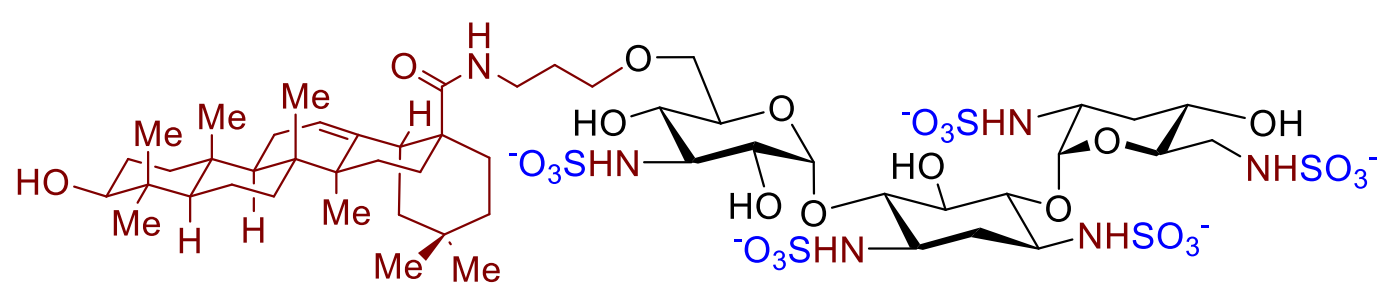

36

FGF2 (172 ± 2.2 μM)

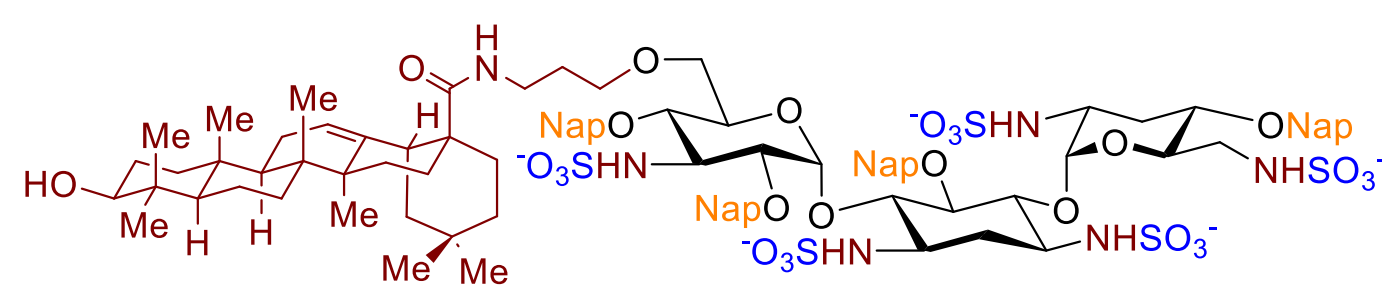

37

FGF2 (0.65 ± 0.02 μM)
FGFR1 (14.1 ± 1.01 μM)

Figure S46. IC_{50} values of Group E Ligands with Na^{+} as a counterion. Data are shown as mean ± SEM (n=3)

Group F (Tetrasaccharide ligands)

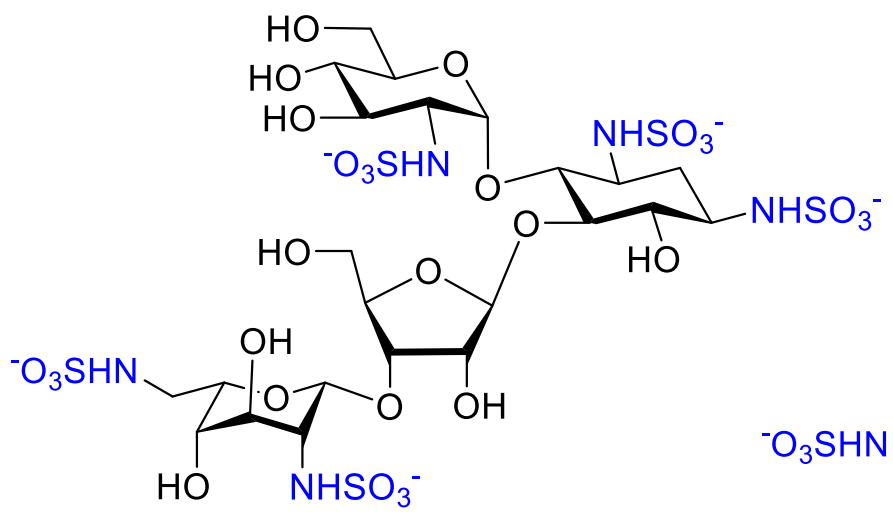

41
FGF2 (108 ± 1.65 μM)

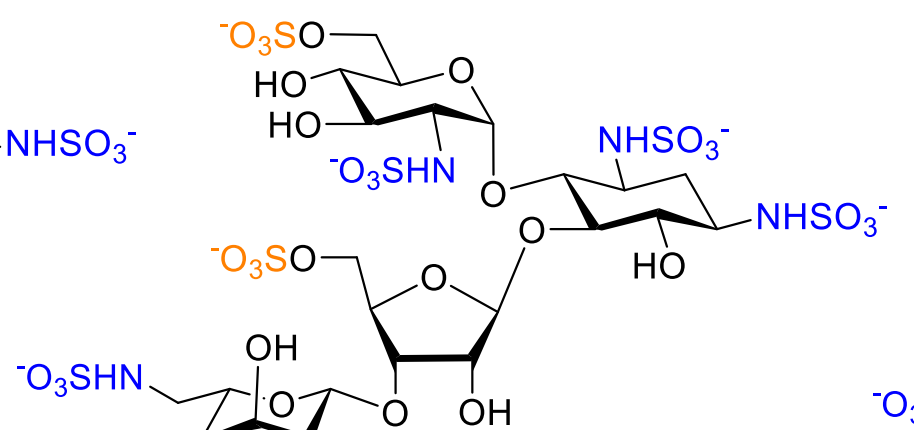

42
FGF2 (159 ± 1.89 μM)

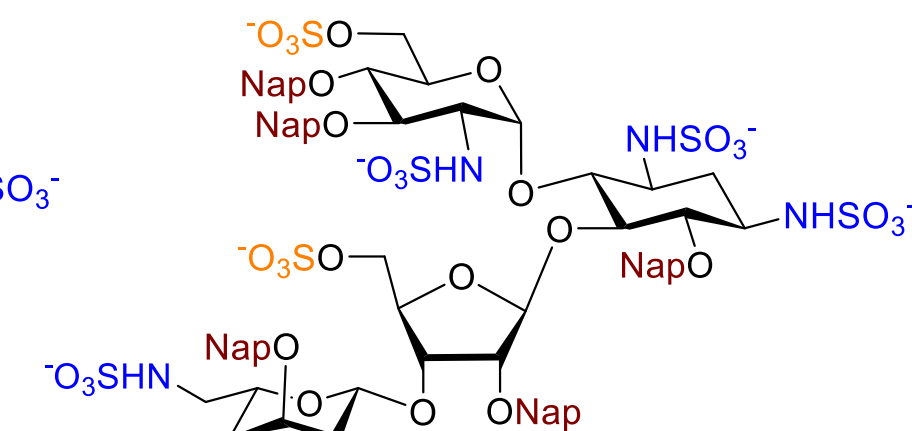

43
FGF2 (3.81 ± 0.23 μM)

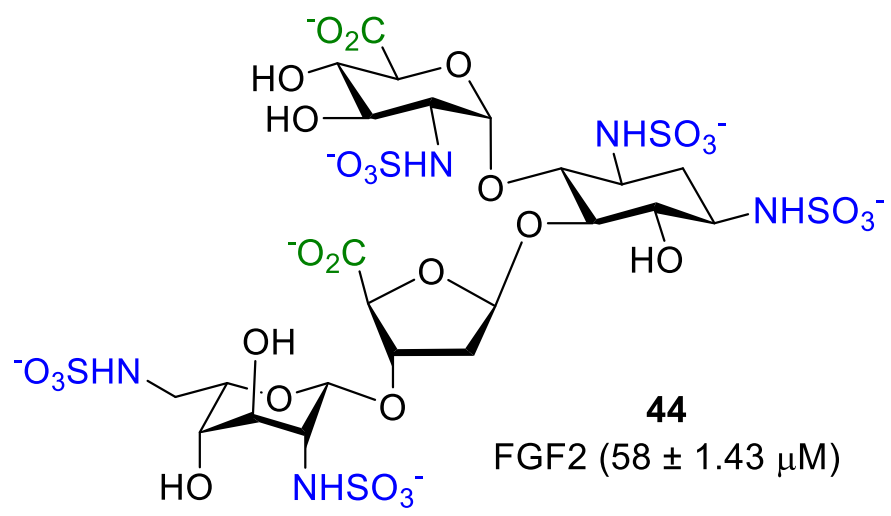

44
FGF2 (58 ± 1.43 μM)

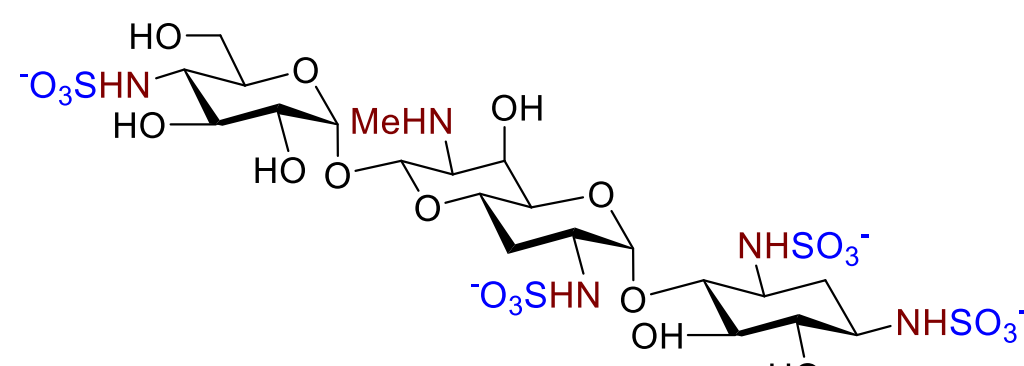

40
FGF2 (4.19 ± 0.62 μM)

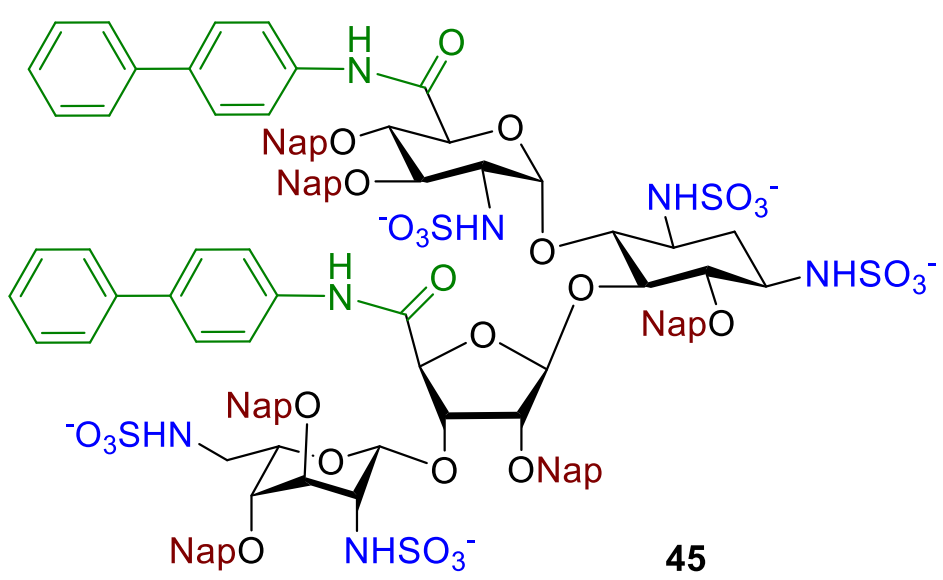

45
FGF2 (2.30 ± 0.01 μM)

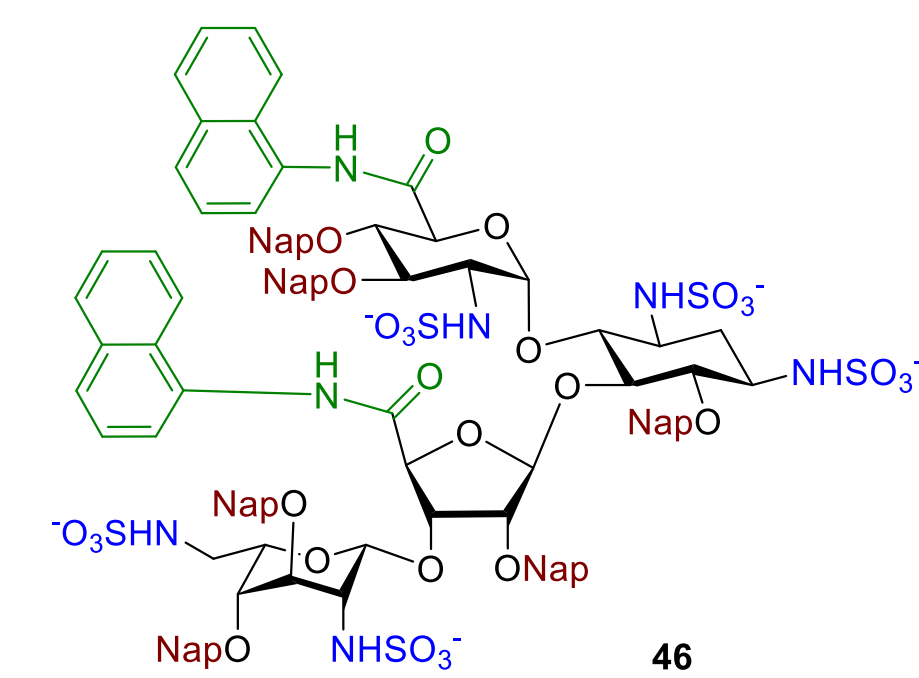

46
FGF2 (3.54 ± 0.05 μM)

Figure S47. IC_{50} values of Group F Ligands with Na^+ as a counterion. Data are shown as mean ± SEM (n=3)

Computational Studies

Electrostatic potential surface of FGF2, FGFR1, and VEGF165 in complex with HS mimetics in their HS/heparin binding sites or allosteric binding sites.

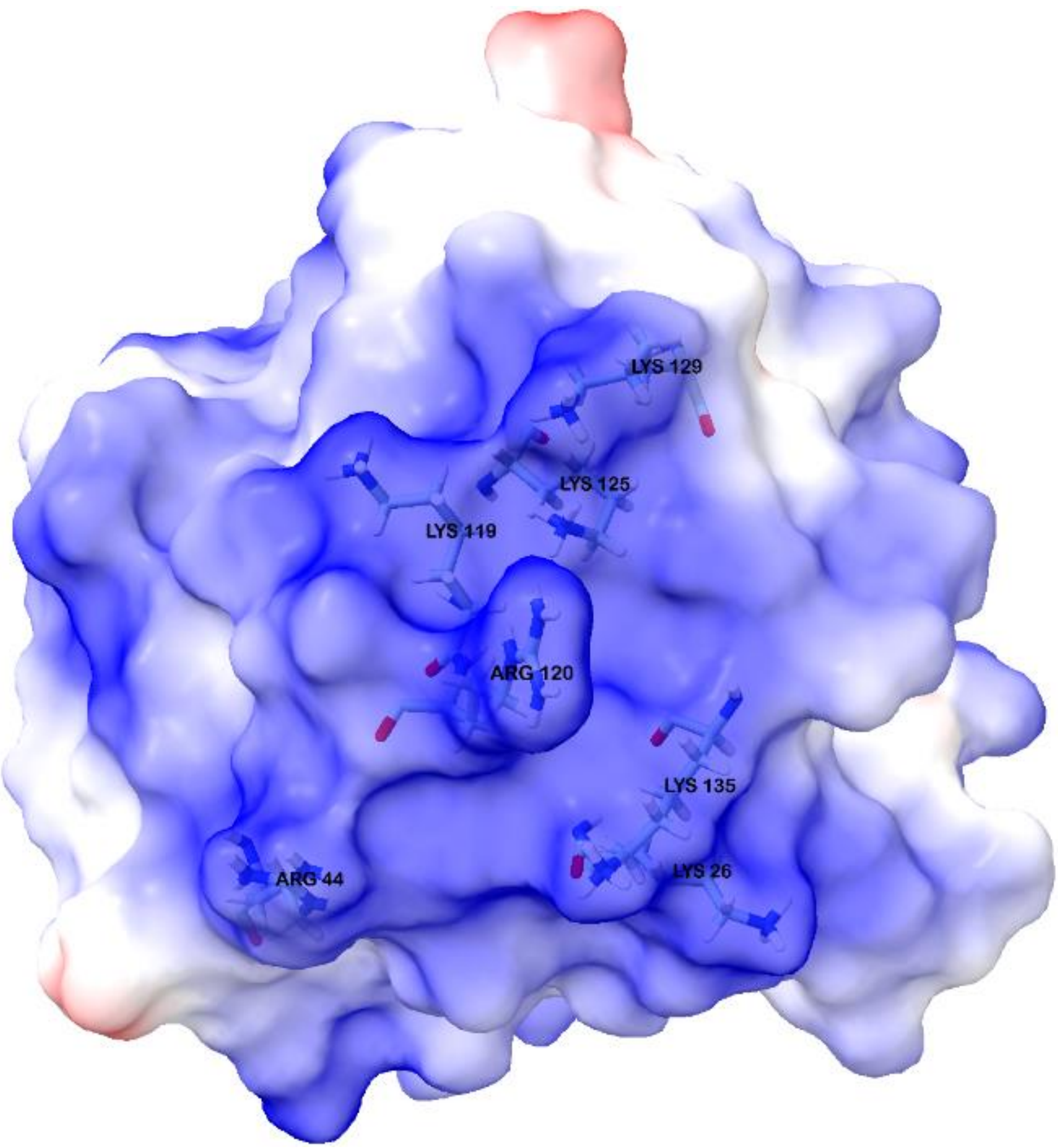

Figure S48. HS binding sites of FGF2

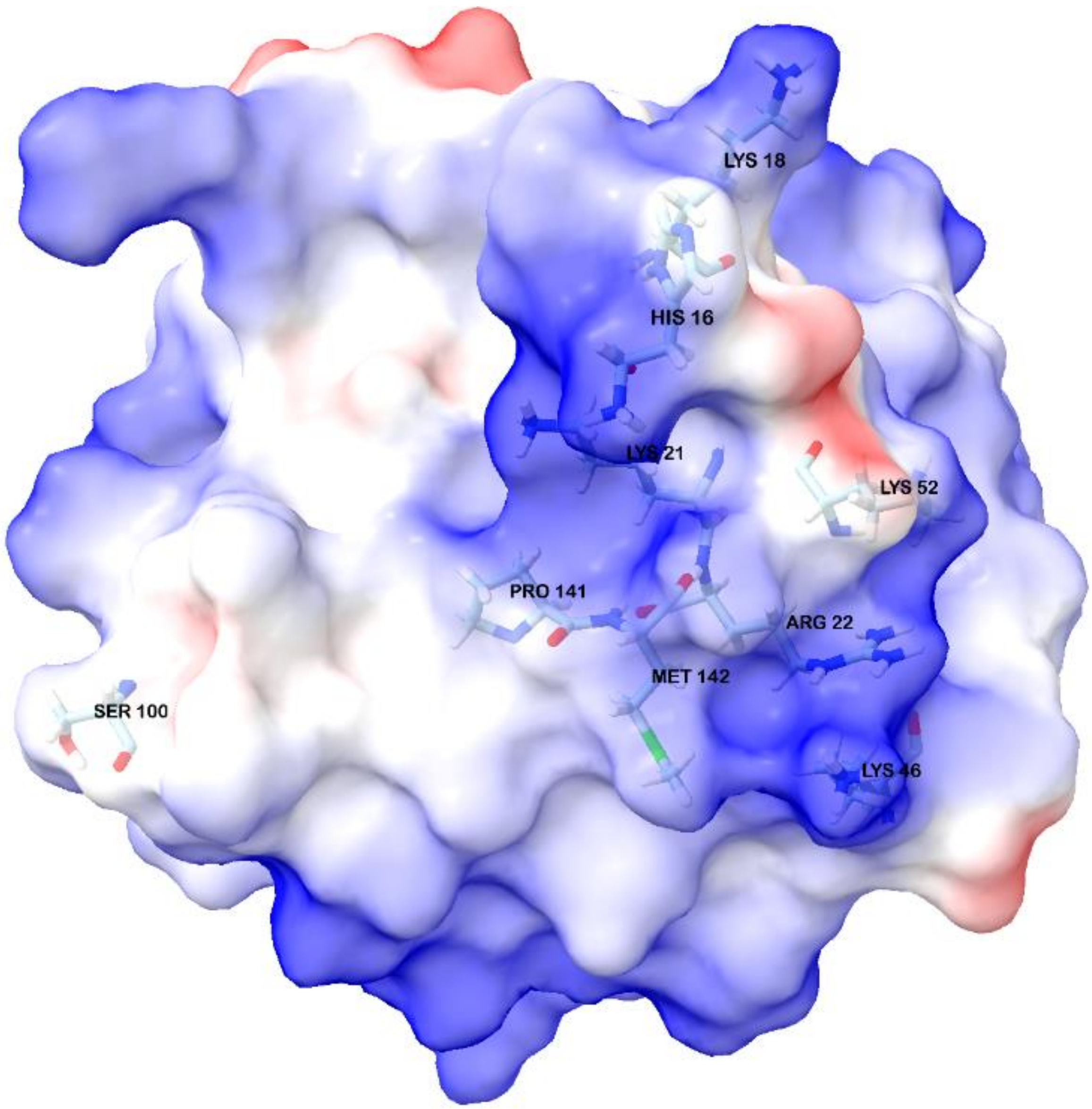

Figure S49. FGFR1 D2 binding site of FGF2

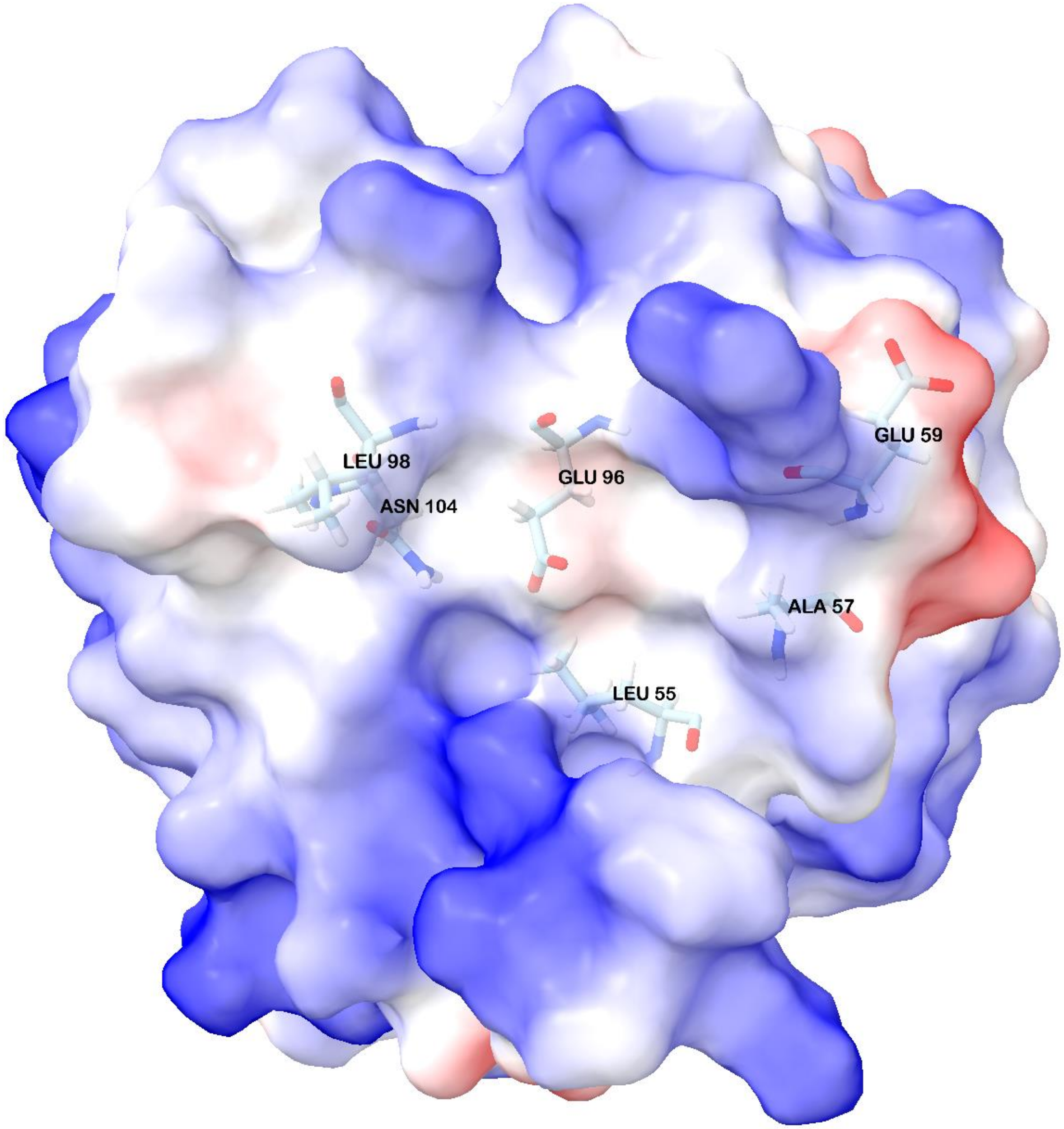

Figure S50. FGFR1 D3 binding site of FGF2

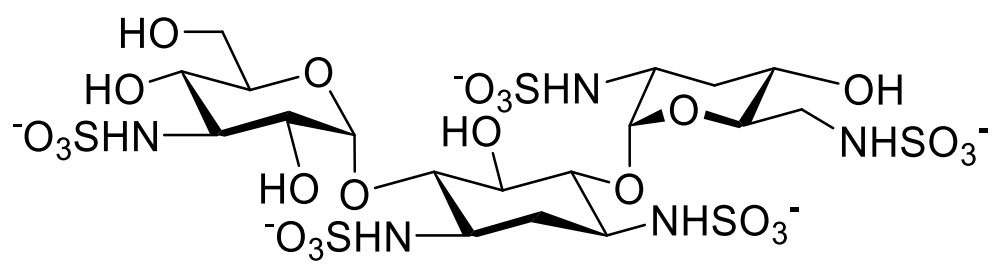

1

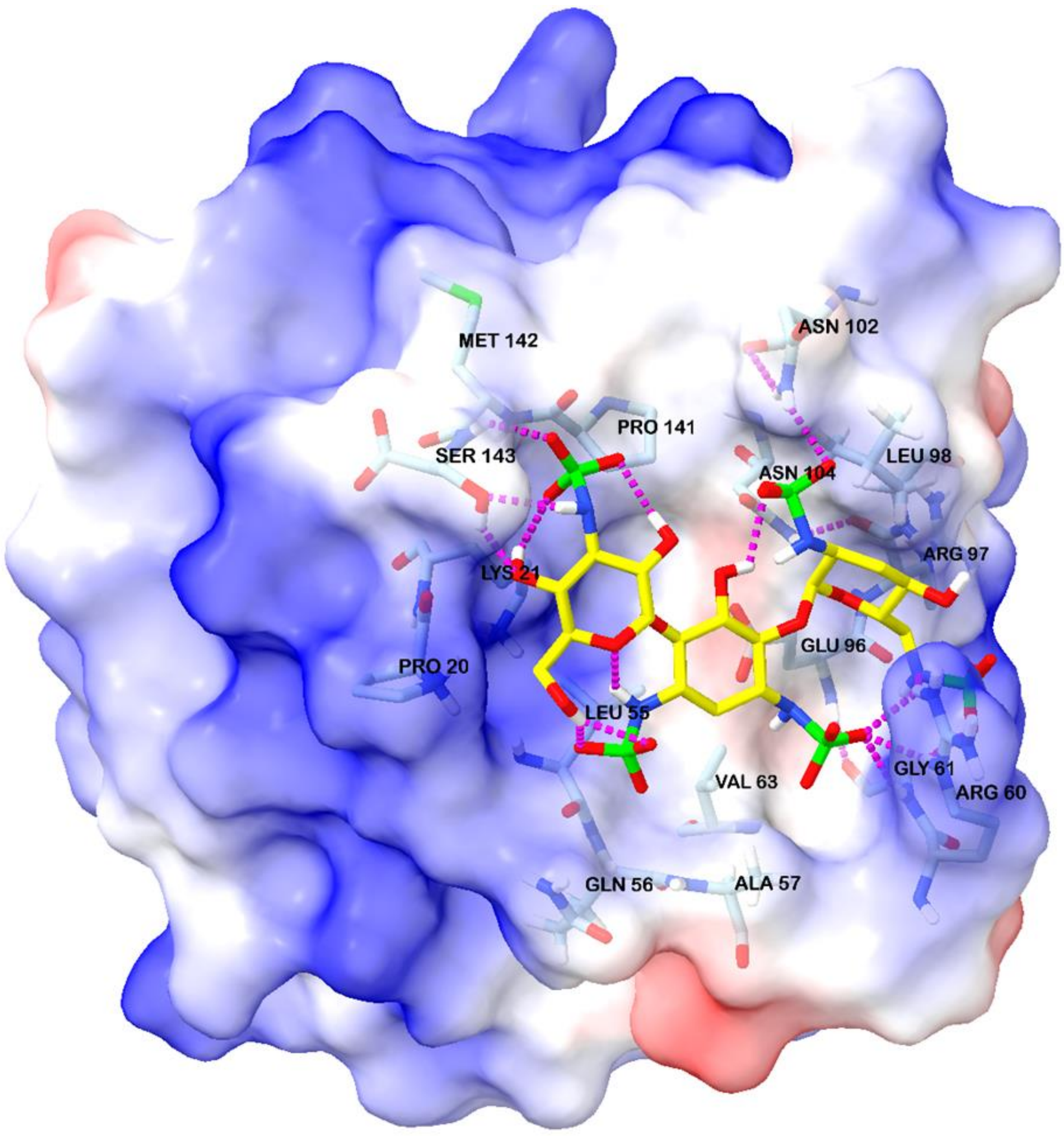

Figure S51. Ligand **1** in complex with FGF2

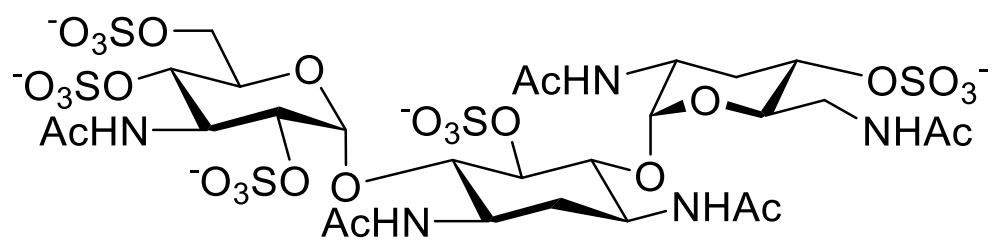

8

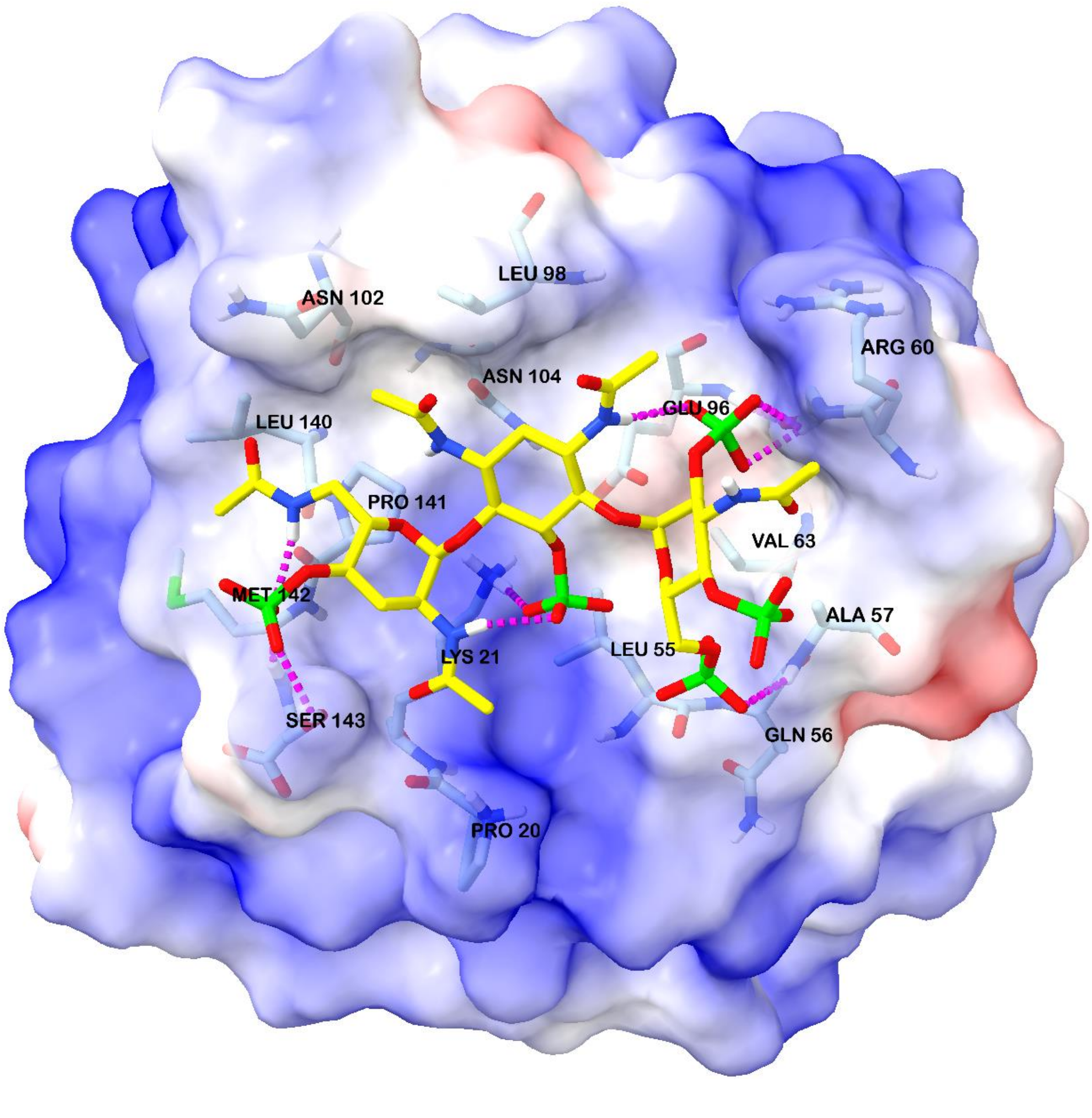

Figure S52. Ligand **8** in complex with FGF2

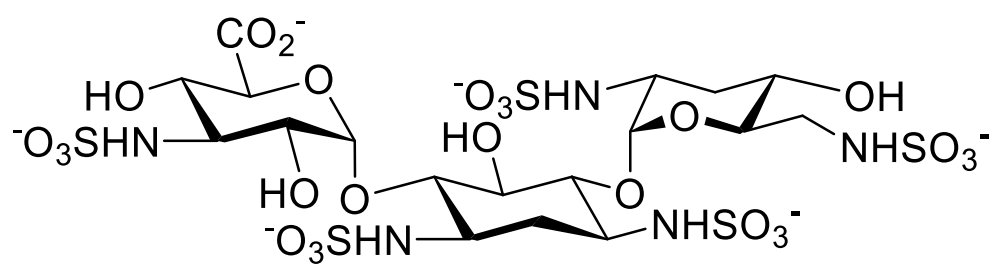

5

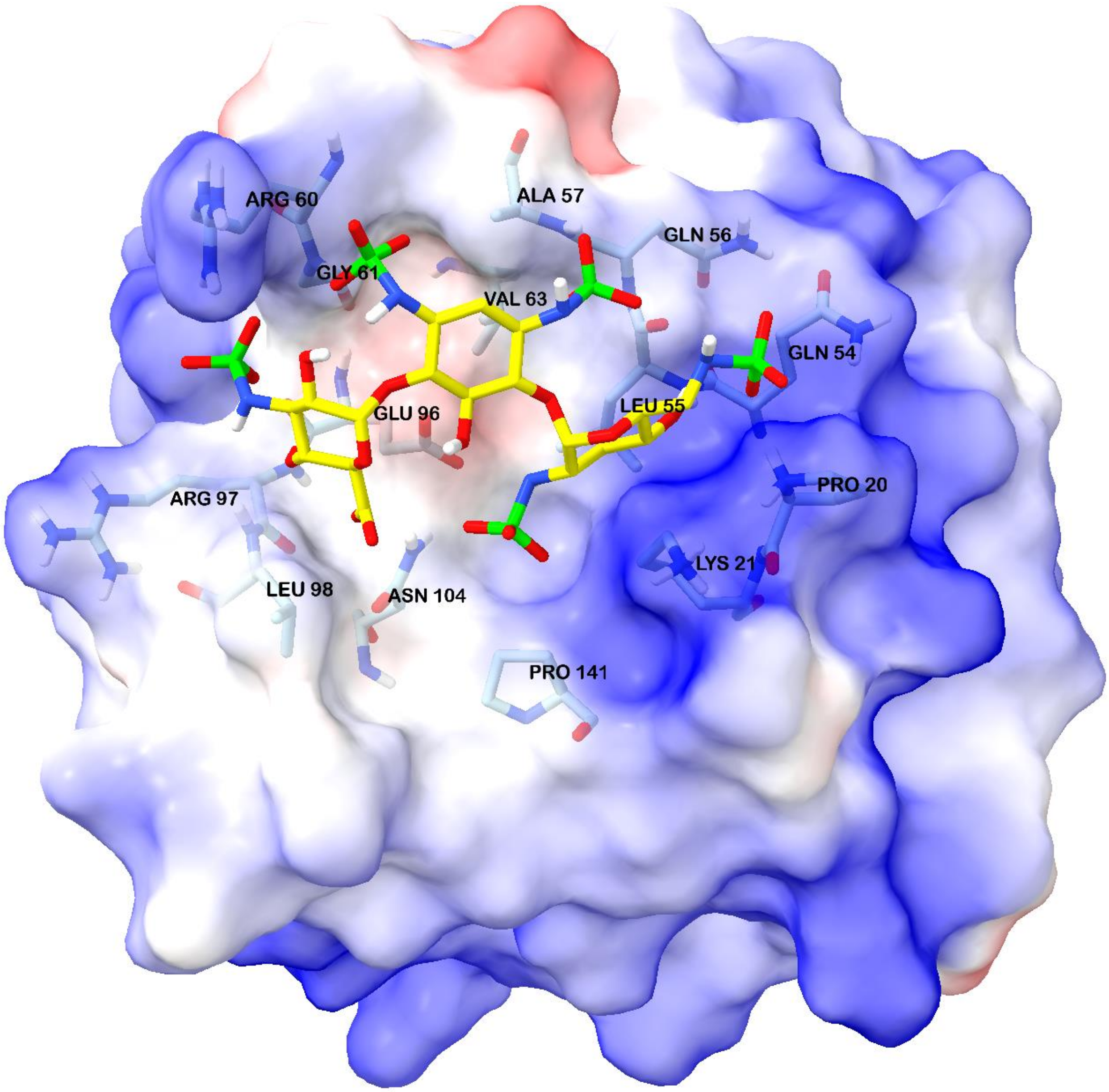

Figure S53. Ligand **5** in complex with FGF2

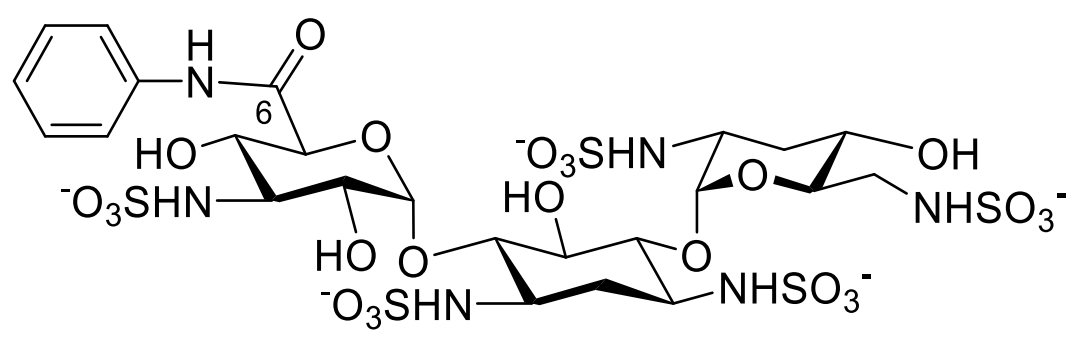

16

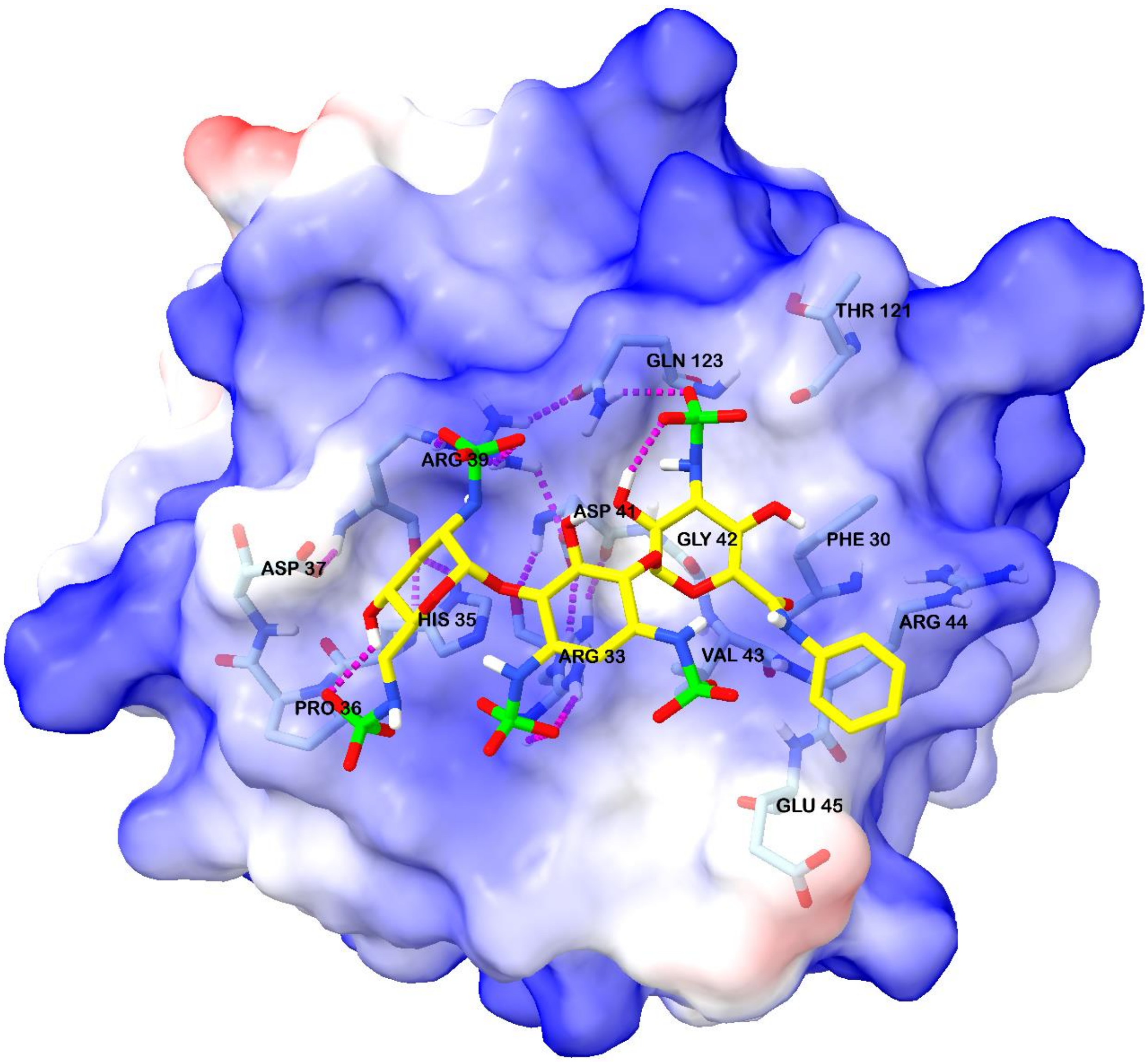

Figure S54. Ligand **16** in complex with FGF2

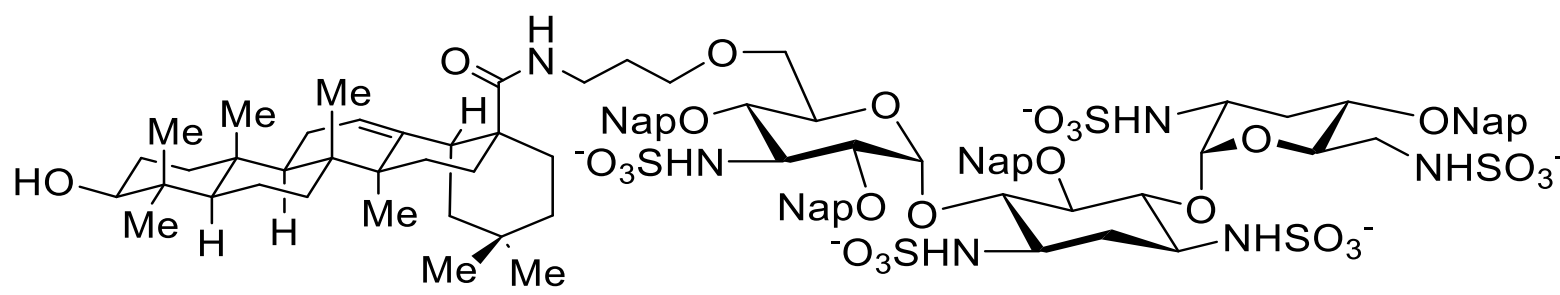

37

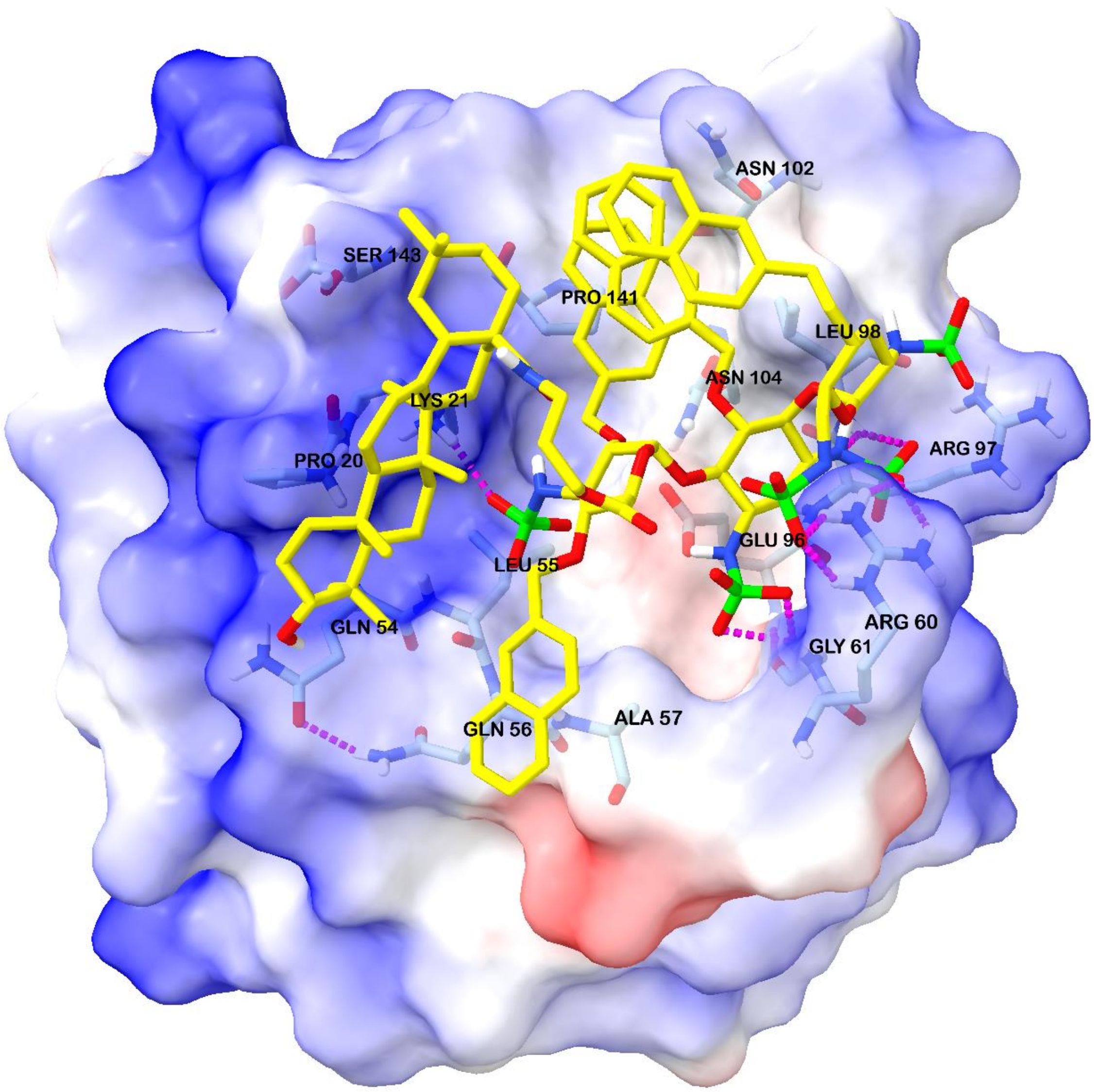

Figure S55. Ligand **37** in complex with FGF2

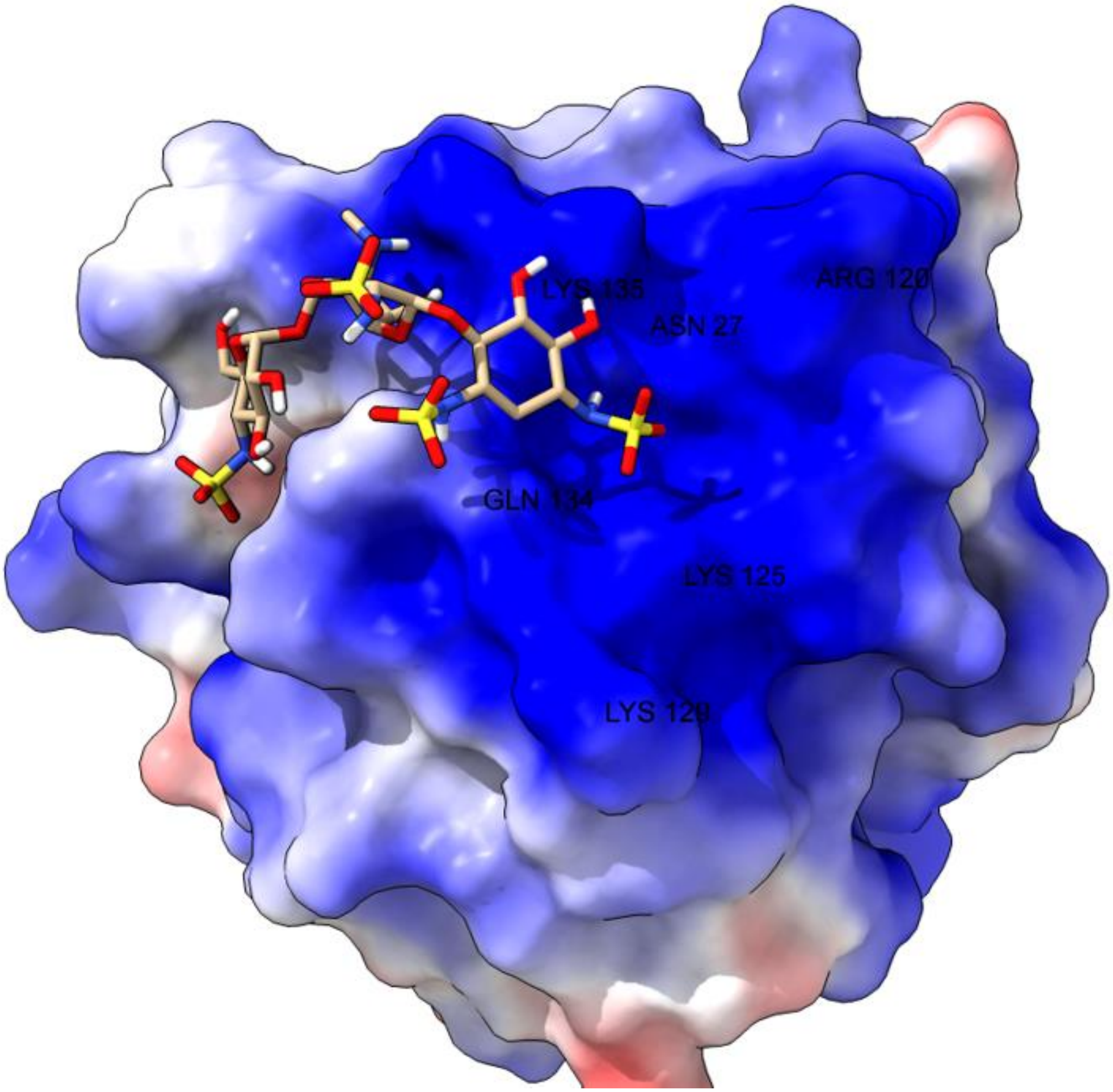

Figure S56. Ligand **40** in complex with FGF2

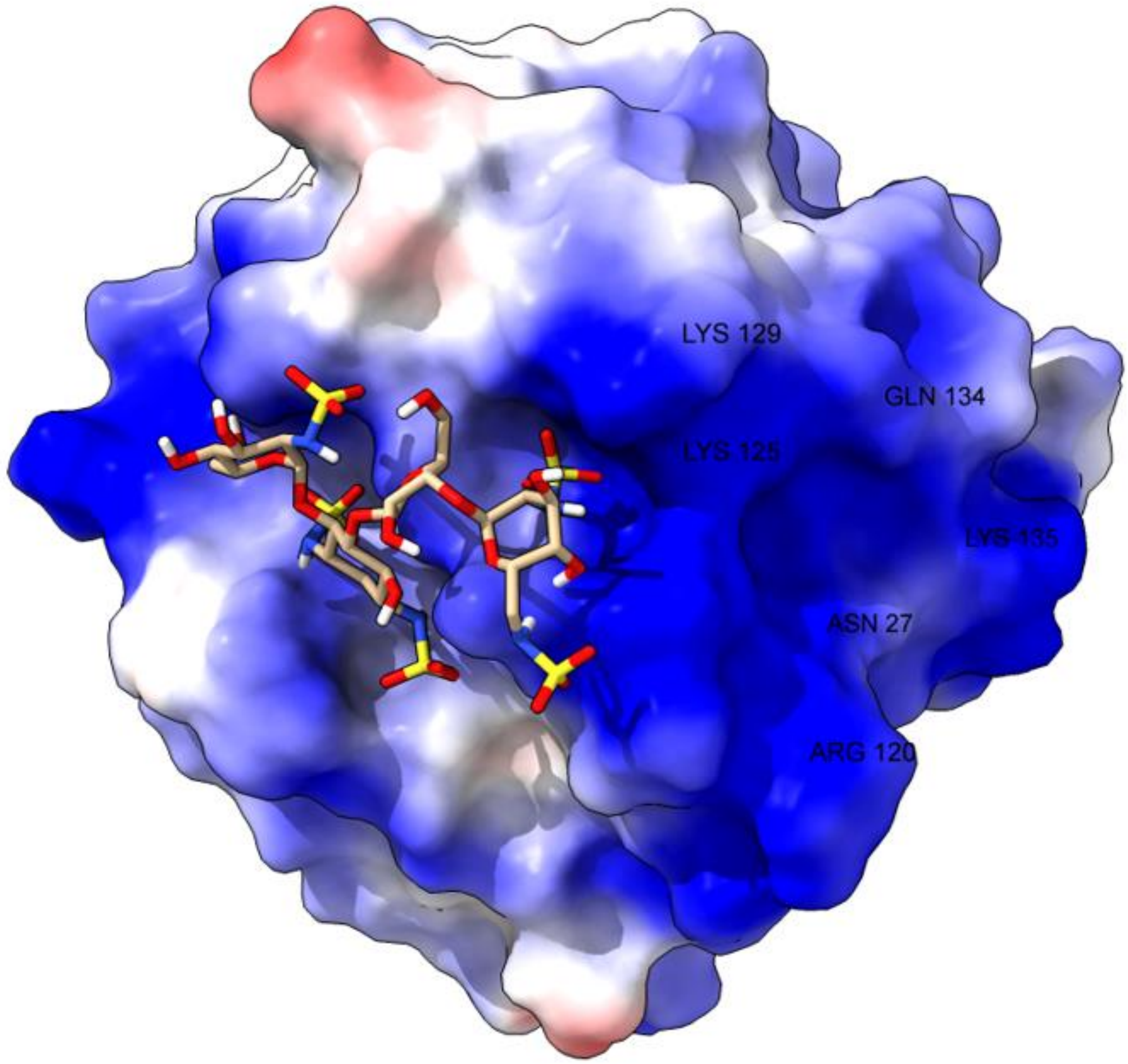

Figure S57. Ligand **41** in complex with FGF2

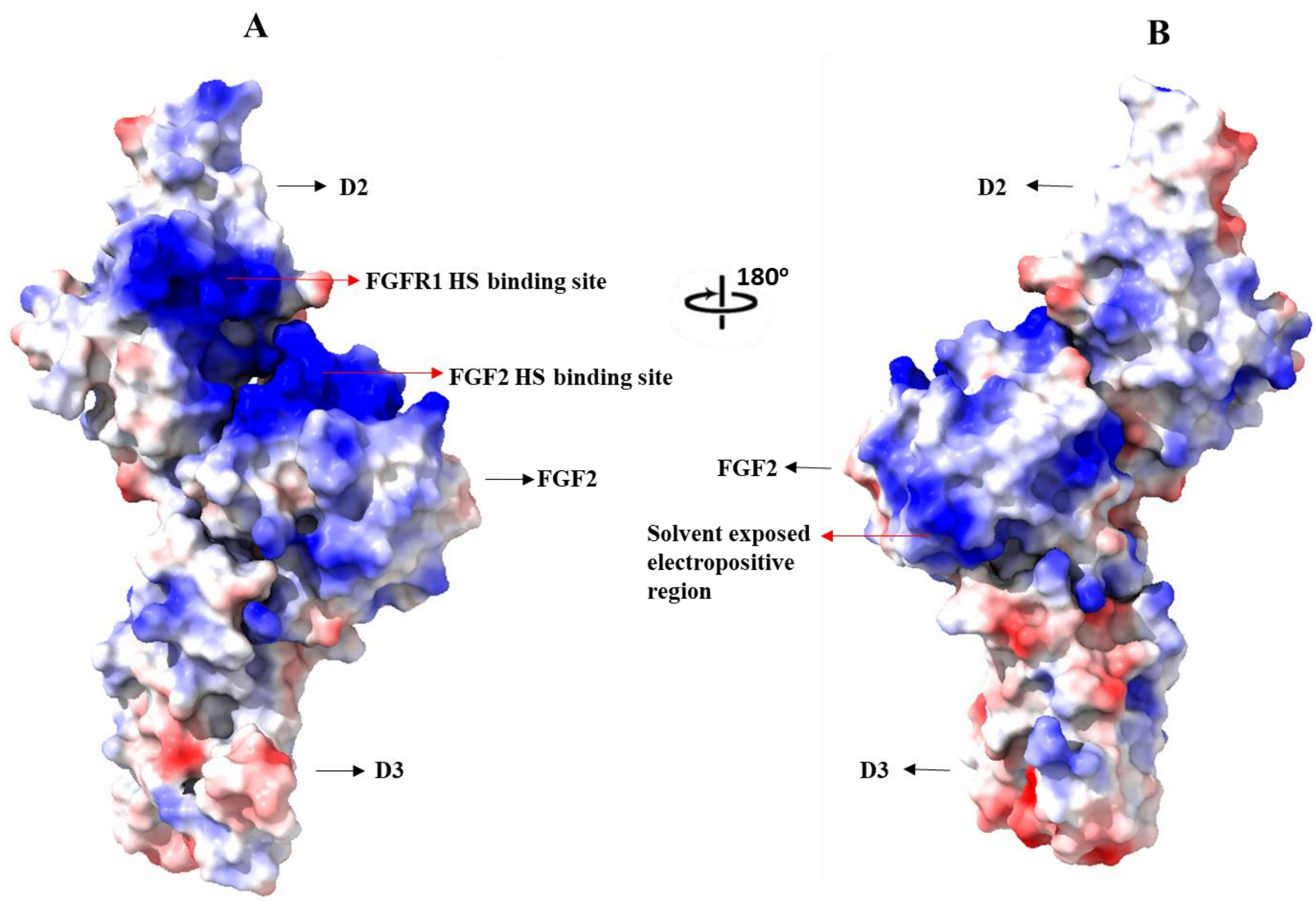

Figure S58. Electrostatic potential surface of FGFR1 complexed with FGF2. (A) The front view highlights the HS binding site of FGFR1 and FGF2, showing key basic residues, such as lysine and arginine, constituting the heparin/HS binding site. (B) A back view illustrates electrostatic potential distribution across the complex, provides insights into the overall charge distribution, and identifies potential secondary binding sites.

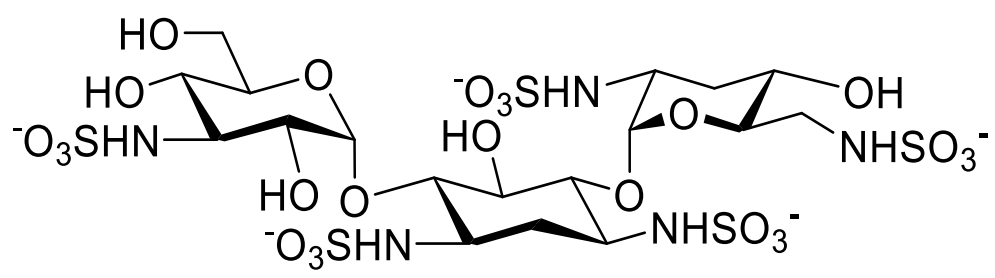

1

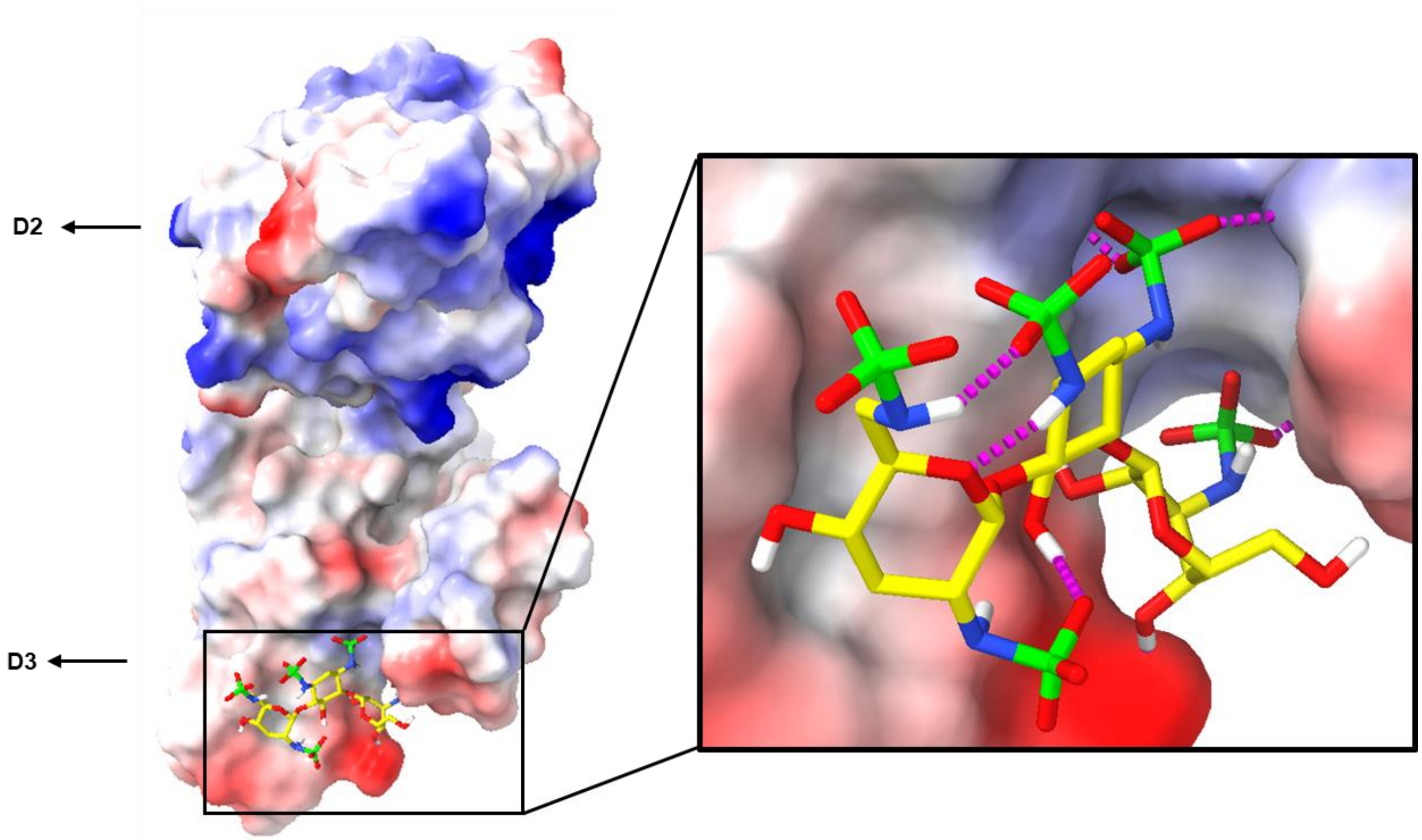

Figure S59. Electrostatic potential surface of FGFR1 complexed with ligand **1**

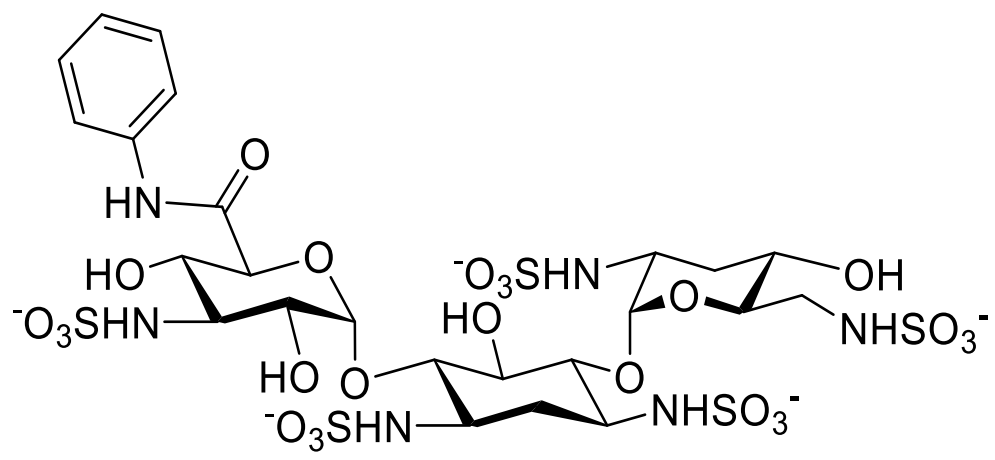

16

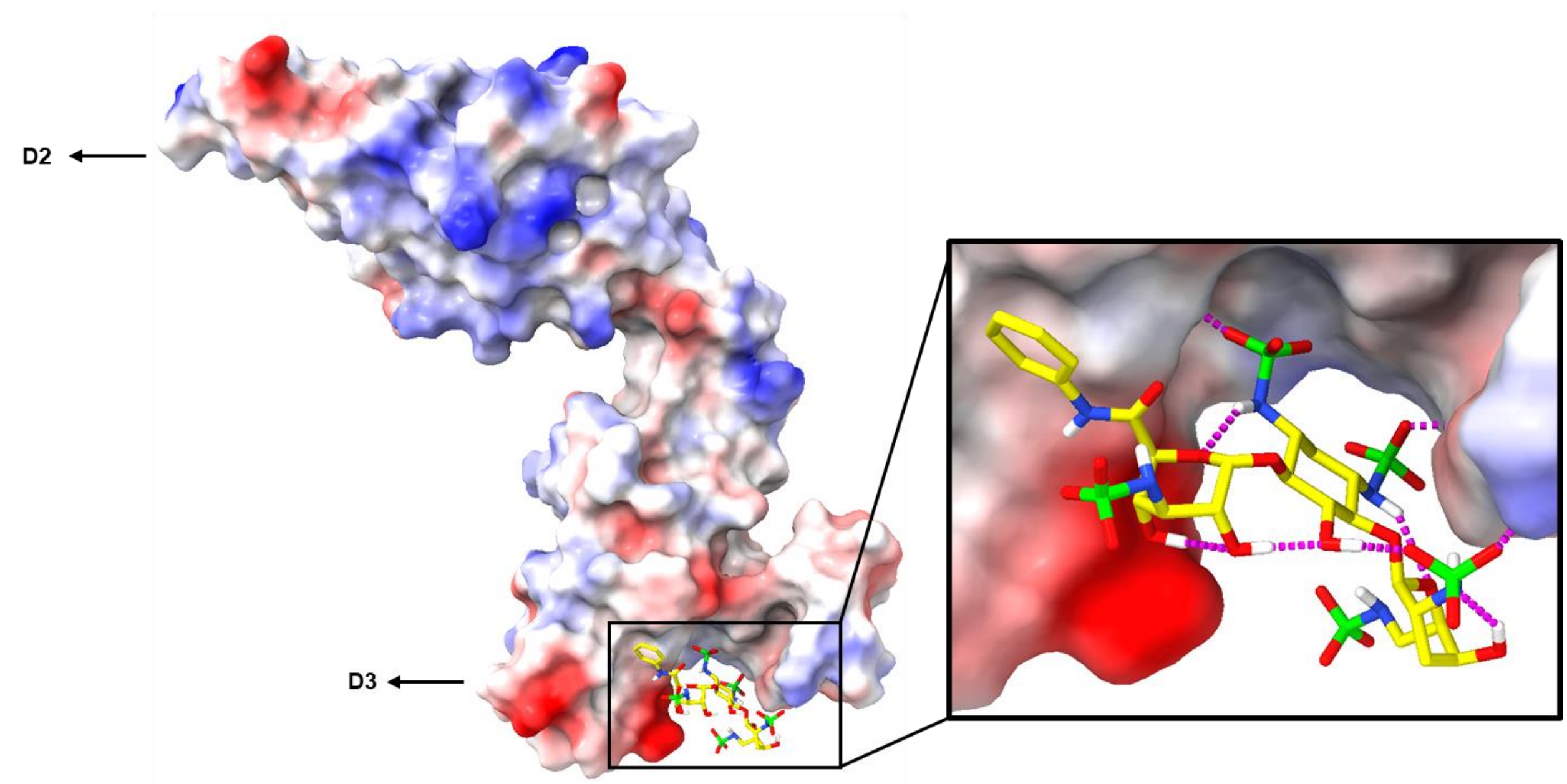

Figure S60. The electrostatic potential surface of FGFR1 complexed with ligand **16**

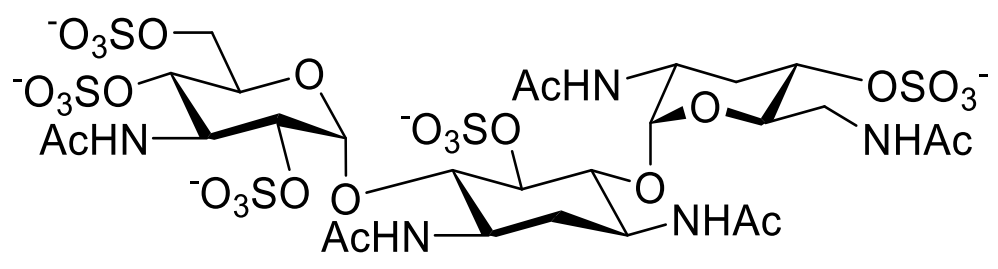

8

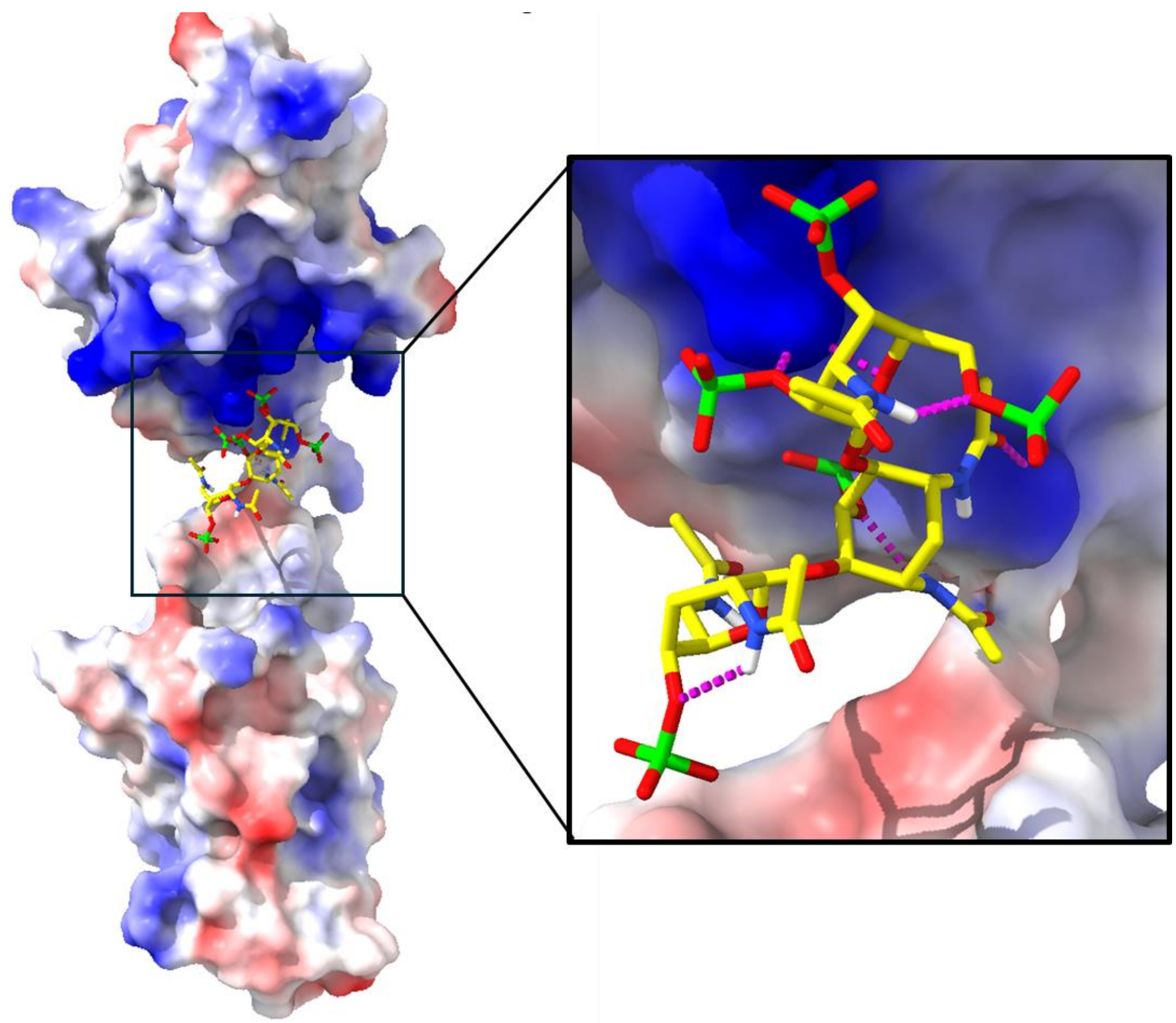

Figure S61. The electrostatic potential surface of FGFR1 complexed with ligand **8**

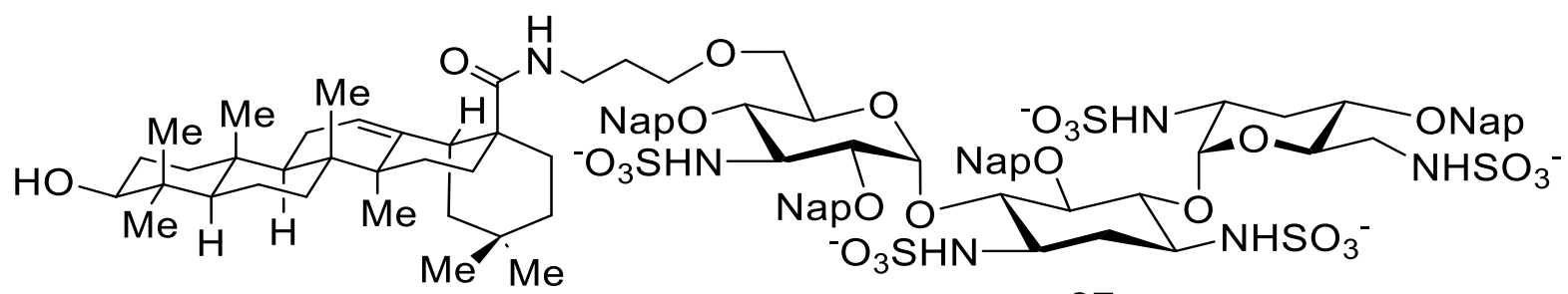

37

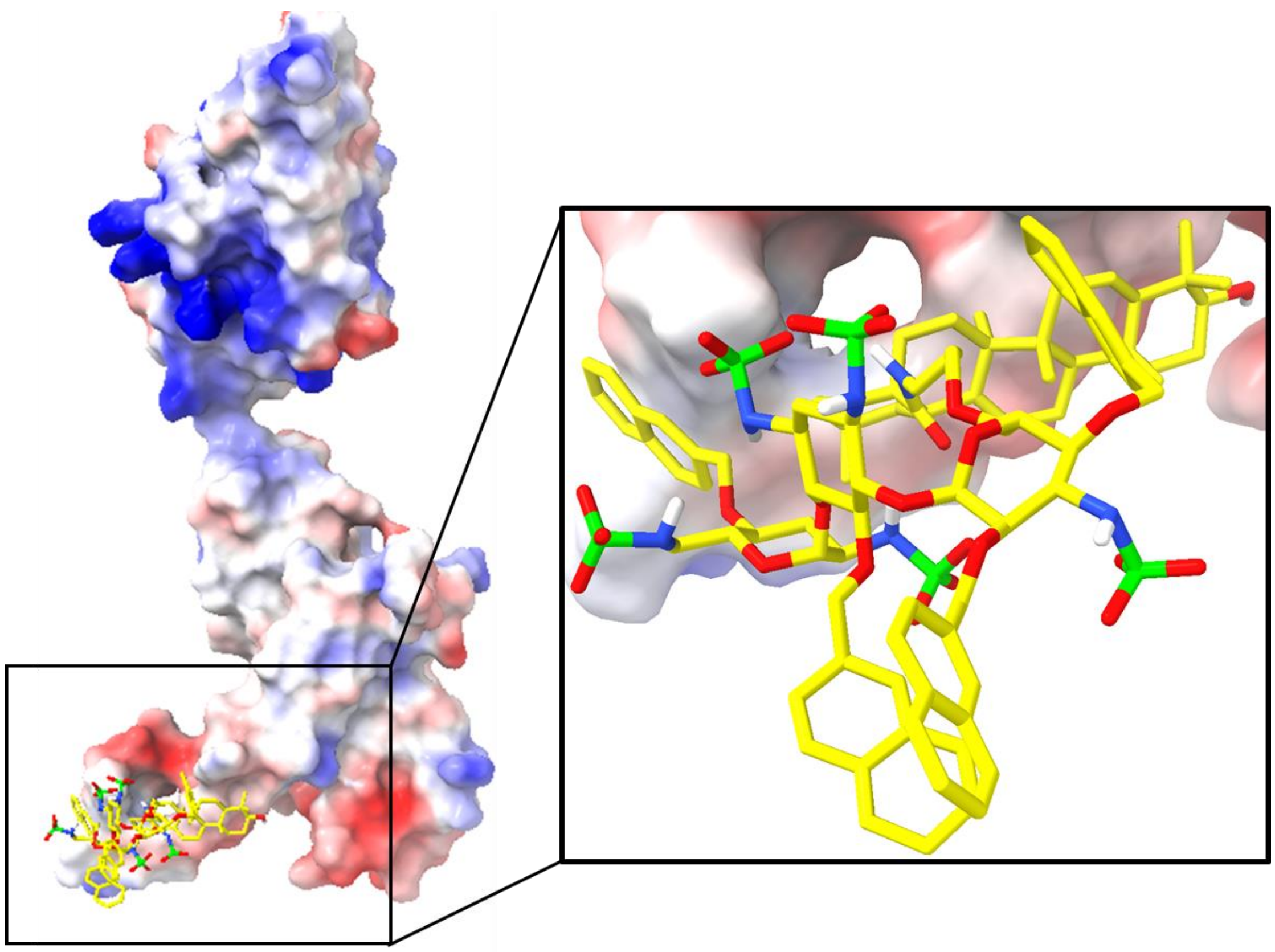

Figure S62. The electrostatic potential surface of FGFR1 complexed with ligand **37**

Electrostatic potential surface of ligands in complex with $VEGF_{165}$.

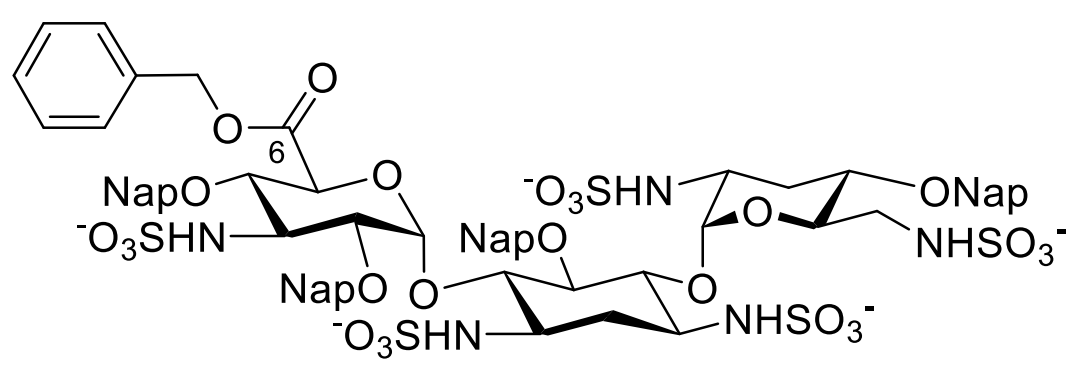

35

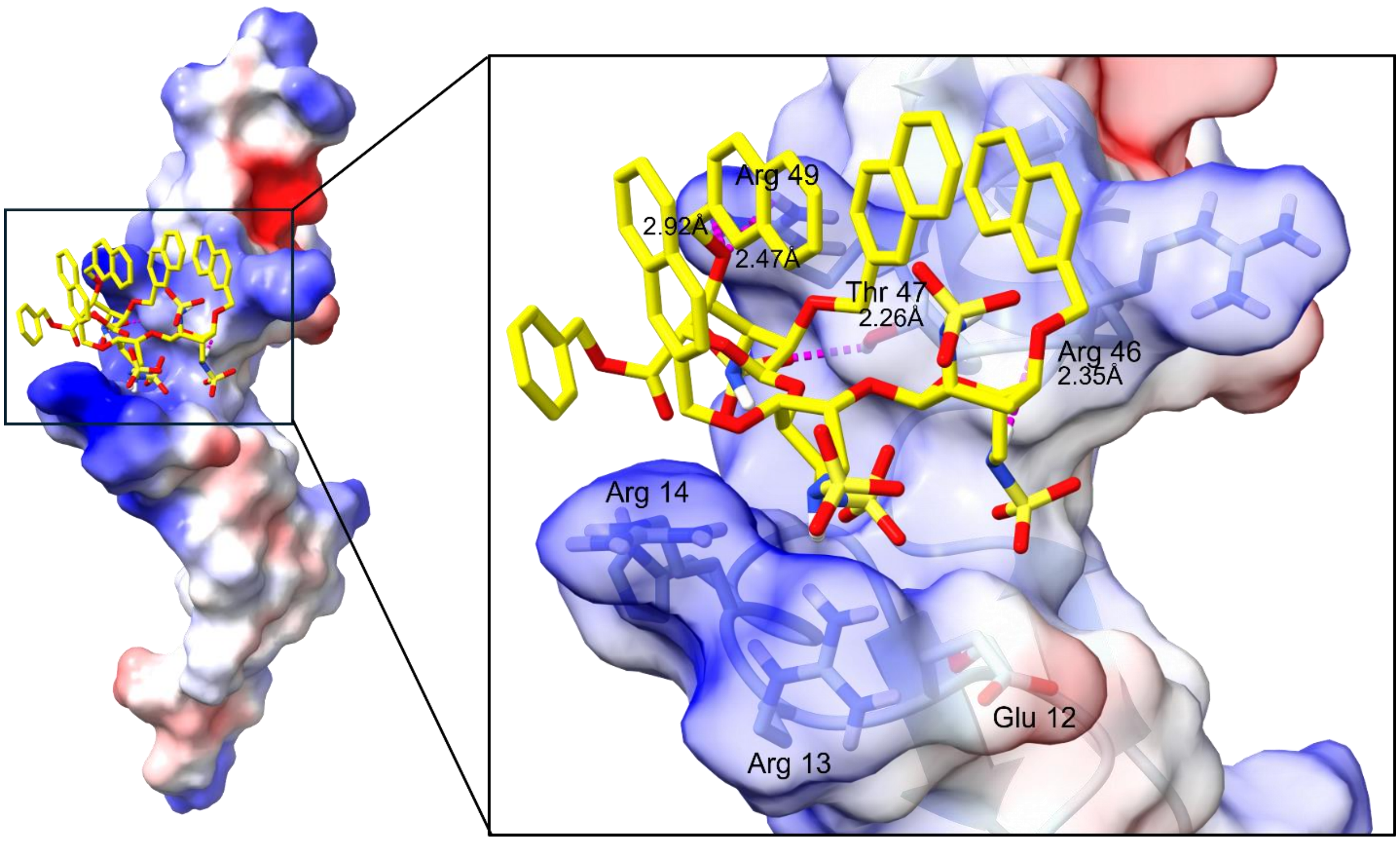

Figure S63. Ligand **35** in complex with the HS binding domain of $VEGF_{165}$ monomer.

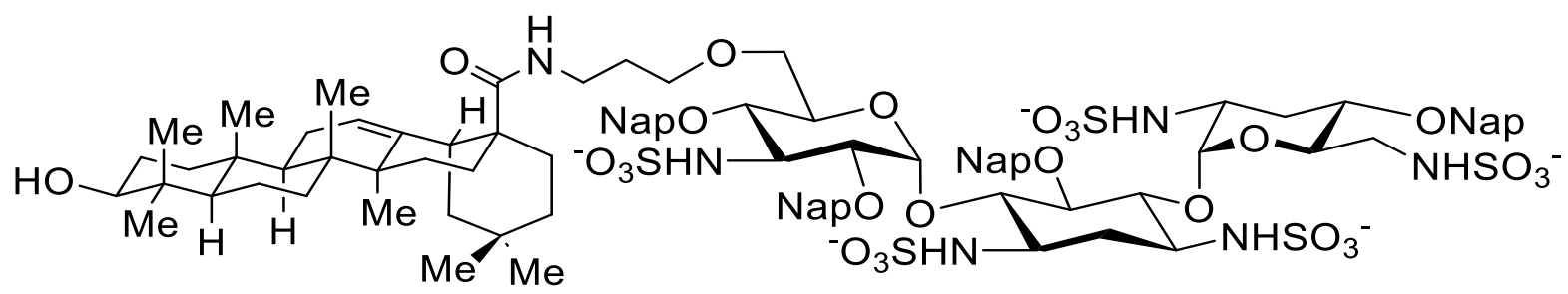

37

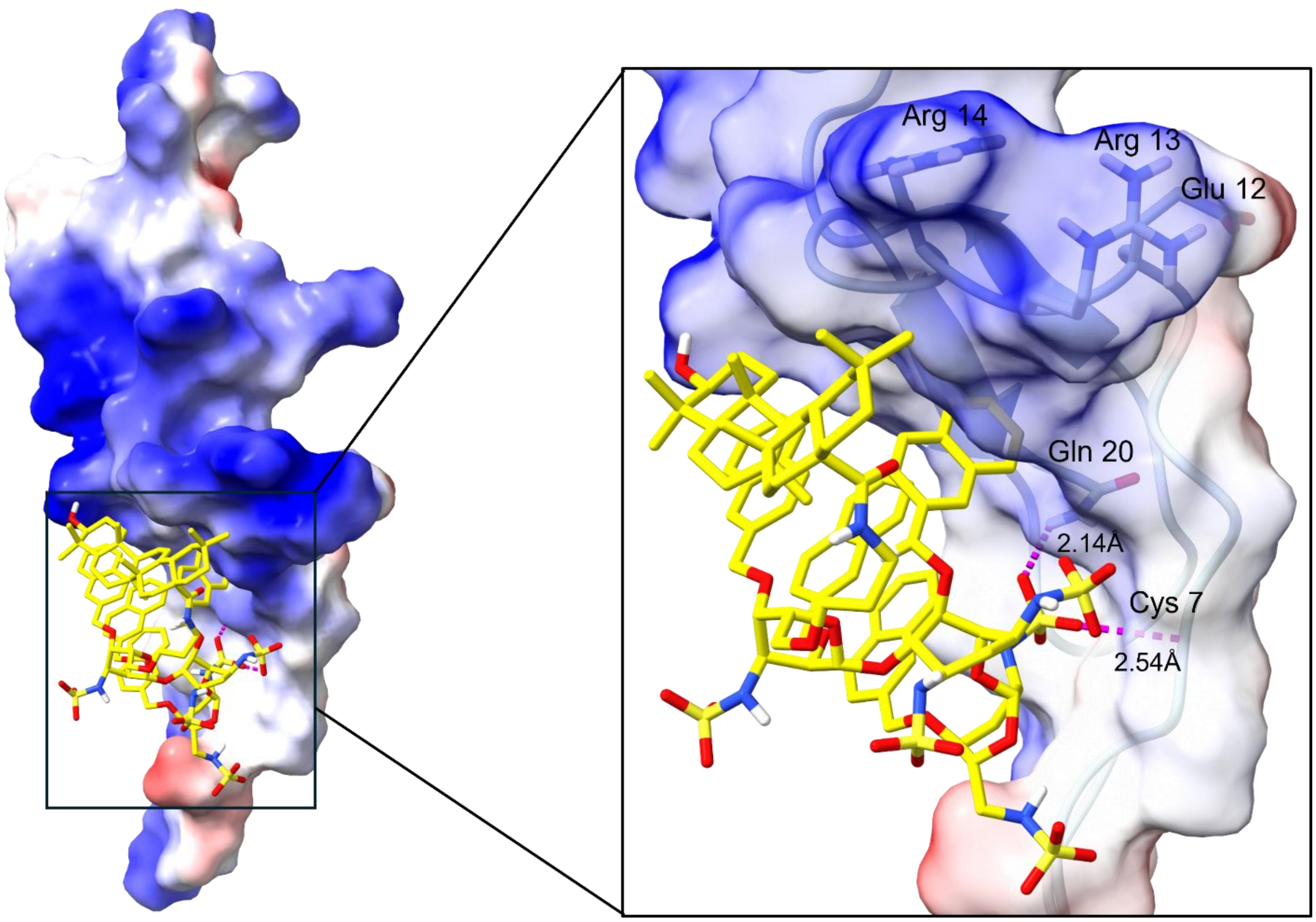

Figure S64. Ligand **37** bound to $VEGF_{165}$ monomer but does not occupy the HS binding site of the protein.

The root mean square deviation (RMSD) of ligands in complex with FGF2

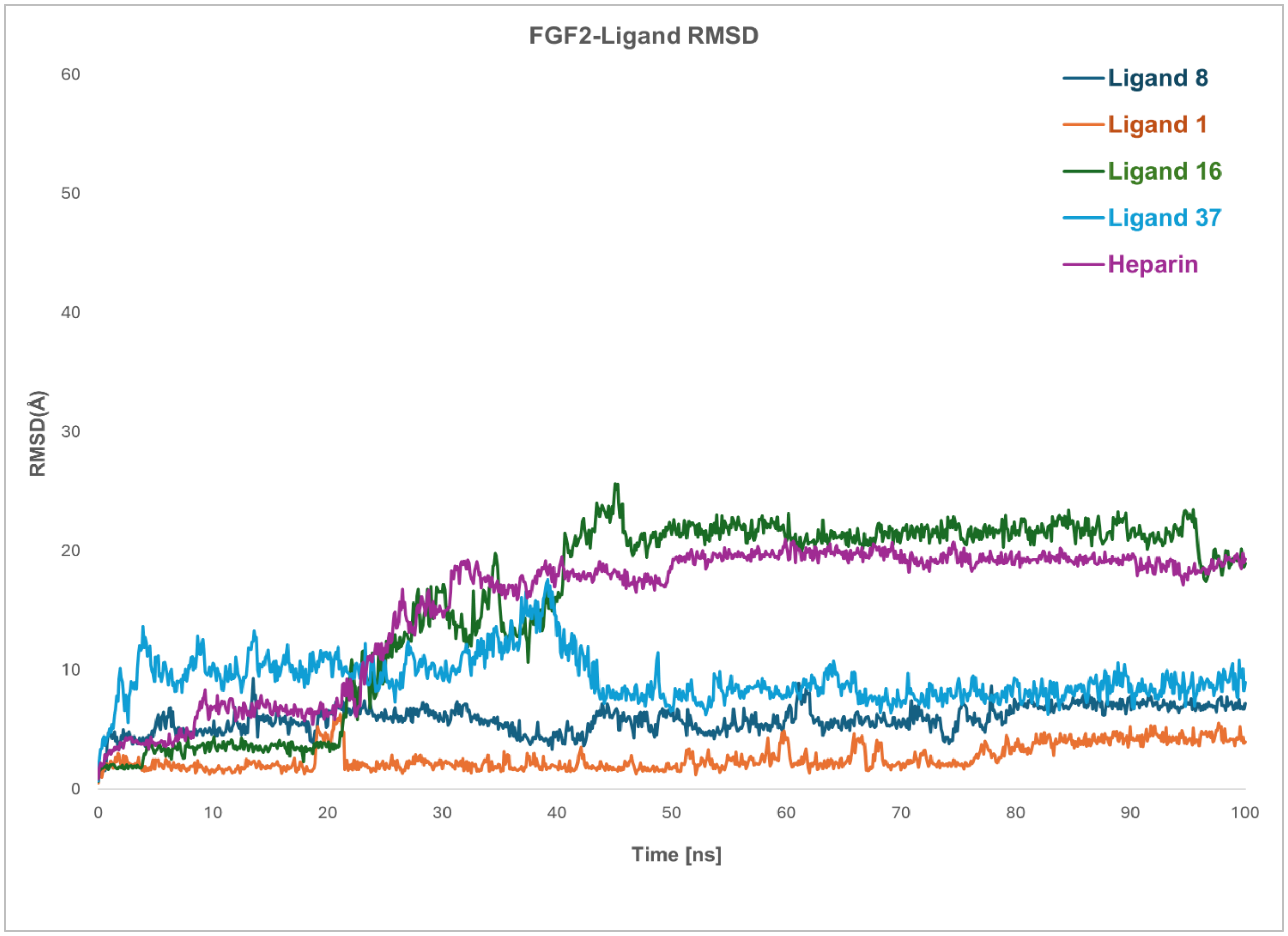

Figure S65. Molecular dynamics (MD) simulation shows the root mean square deviation (RMSD) of ligands **1**, **8**, **16**, **37** and **heparin** in complex with FGF2 over a 100 ns simulation period in complex with the HS binding domain of $VEGF_{165}$ monomer. Ligand **8** (dark blue) remains relatively stable throughout the simulation period and does not show any major drifting in the binding pocket, indicating its relatively high affinity for FGF2. On the other hand, ligands **1**, **16**, **37** all tend to have some drifts during the first half of the simulation time before stabilizing for the remainder of the time, with **16** plateauing downwards (destabilized) towards the end of the simulation. Heparin only had a small spike around the 20 s time but remained more stable throughout the 100 ns simulation period.

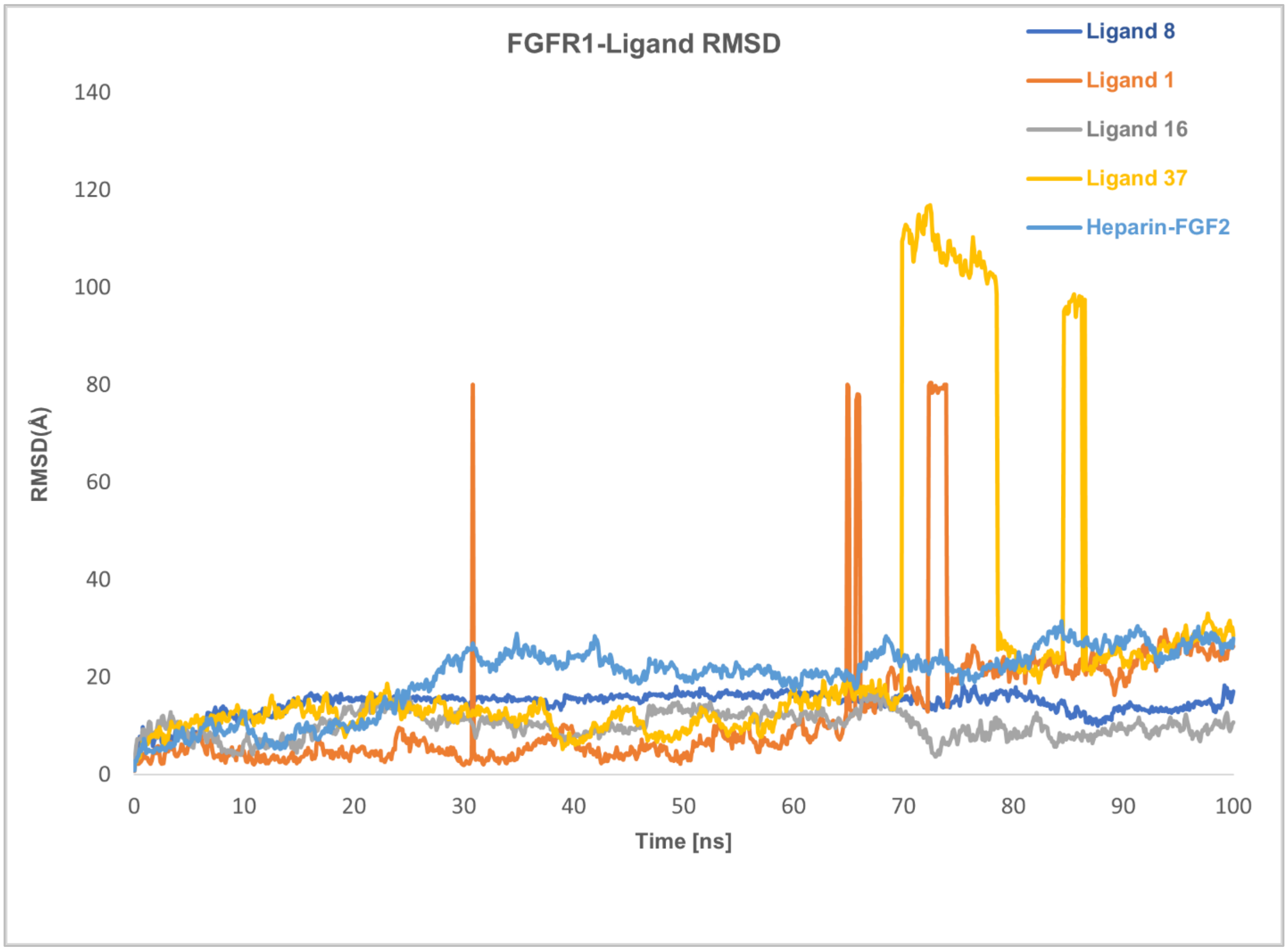

Figure S66. MD simulation shows the RMSD of ligands **1**, **8**, **16**, **37** and **heparin-FGF2** in complex with FGFR1 over a 100 ns simulation period. MD simulations were performed using periodic cell boundaries where some ligands tend to cross the cell boundary and appear on the opposite side of the simulation cell, leading to a spike in the RMSD plot. This behavior is a result of the large size of the receptor complex. Ligands **1** and **37** exhibited poor binding to FGFR1 and remained relatively less stable at the end of the simulation period. Additionally, ligands **1** and **37** bound almost entirely through Van der Walls interactions to a hydrophobic area of the D3 region of FGFR1 which makes them prone to partial and full dissociation events. These dissociations frequently resulted in the ligands crossing the simulation cell boundary, leading to the observed spikes in the RMSD. Ligand **8** shows consistent high stability throughout the simulation period, while **16** and **FGF2-heparin** complex are also relatively stable but less stable than **8**.

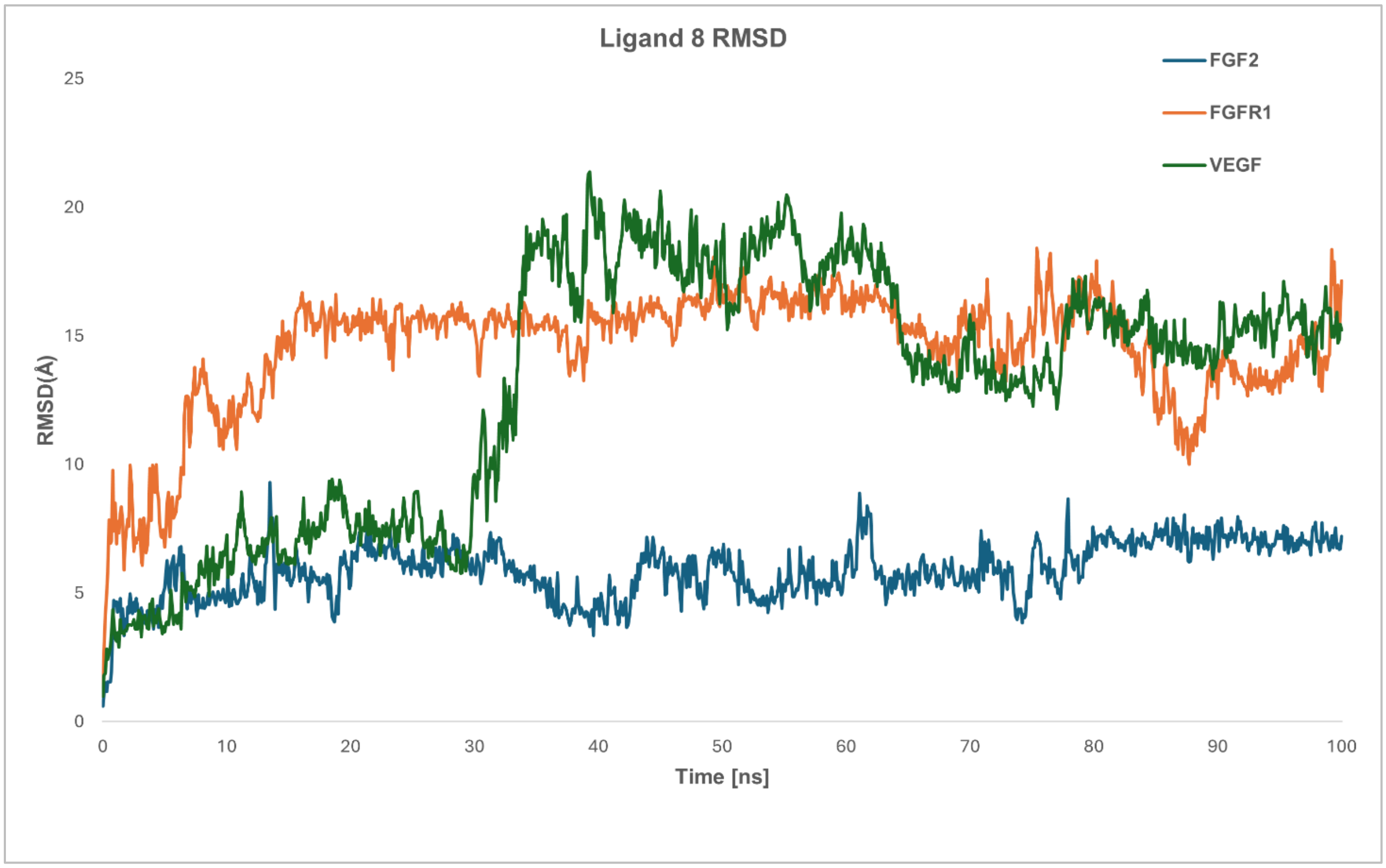

Figure S67. Comparative analysis of the RMSD of ligand **8** in complex with FGF2, FGFR1 and $VEGF_{165}$ over a 100 ns simulation period.

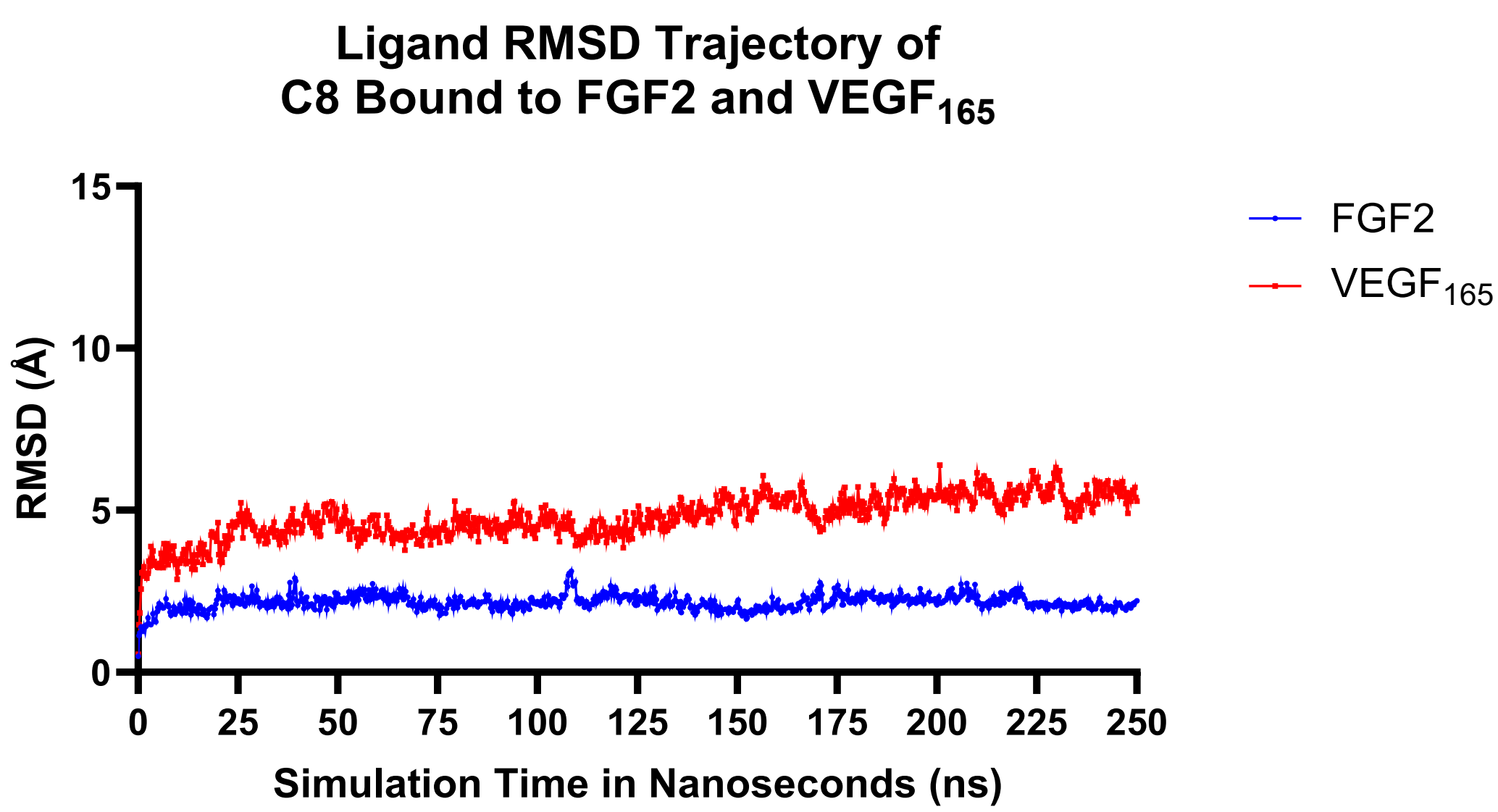

Figure S68. Comparative analysis of the RMSD of ligand **8** in complex with FGF2 and $VEGF_{165}$ for 250 ns simulation period.

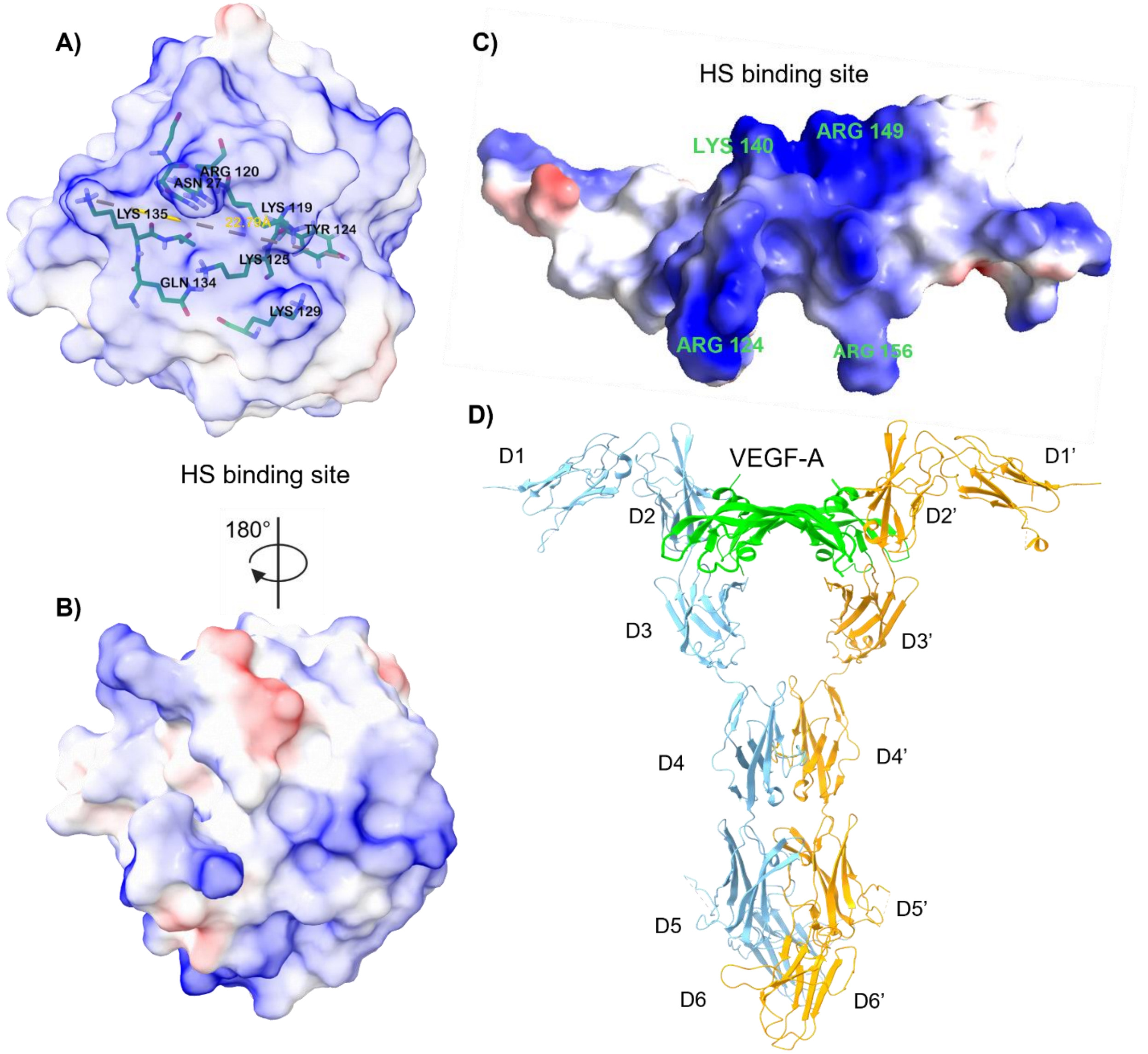

Figure S69. A) The electrostatic potential surface of the FGF2 model highlights the electropositive regions of the proteins (PDB code: 4OEF for FGF2; 2VGH for VEGF165). The surfaces are color-coded to indicate electrostatic potential, with positive potential in blue and negative potential in red. The figures are rotated by 180° to show the electropositive residues on the opposite side. The electrostatic potential surface of FGF2, displaying the primary heparan sulfate (HS) binding site. B) Rear view of FGF2, illustrating the second electropositive surface that interacts with the D2 and D3 domains of FGFR1. C) The heparin binding domain of $VEGF_{165}$ monomer (PDB code: 2VGH, the B monomer) showing key basic residues in the active site. D) Structure of the extracellular domain of VEGFR1 in complex with VEGF-A. Six of the seven organized immunoglobulin domains of VEGFR1 are shown with VEGF-A bound to the D2 domain

(between D2 and D3 domains). Each monomer ribbon of VEGFR1 is depicted in a different color code with D1-D6 and D1'-D6' domains shown in cyan and gold respectively while the VEGF-A homodimer is illustrated in green.

Key non-covalent interactions of the protein-ligand complex. Values are reported as percentages.

Ligand 8 - VEGF Key Interactions							
**Residue**	Arg 13	Arg 14	His 16	Arg 35	Arg 39	Thr 47	Arg 49
**None**	69	31	45	76	57	79	41
**HB**	7	17	25	2	3	5	13
**Hyd**	3	3	13	-	2	13	1
**Hyd+HB**	2	6	17	-	1	3	1
**Ion**	6	8	-	3	5	-	4
**Ion+HB**	11	29	-	18	27	-	38
**Ion+Hyd**	-	1	-	-	-	-	-
**Ion+Hyd+HB**	2	4	-	-	4	-	2

Figure S70. Key interactions of ligand **8** to VEGF as the fraction of the 100 ns simulation, the given interaction was present, including no interaction (none), hydrogen bonding (HB), hydrophobic (Hyd), ionic (Ion), and combinations of multiple interactions, interactions that were present in less than 1% of the simulation are shown using a dash.

Ligand 37 - VEGF Key Interactions						
**Residue**	Ala 1	Pro 6	Cys 7	Pro 9	Arg 13	Lys 15
**None**	65	17	56	58	46	21
**HB**	-	-	1	-	3	-
**Hyd**	35	83	43	42	3	7
**Hyd+HB**	-	-	-	-	2	10
**Ion**	-	-	-	-	4	5
**Ion+HB**	-	-	-	-	41	8
**Ion+Hyd**	-	-	-	-	-	3
**Ion+Hyd+HB**	-	-	-	-	2	46

Figure S71. Key interactions of ligand **37** to VEGF as the fraction of the 100 ns simulation, the given interaction was present, including no interaction (none), hydrogen bonding (HB), hydrophobic (Hyd), ionic (Ion), and combinations of multiple interactions that were present in less than 1% of the simulation are shown using a dash.

Ligand 35 - VEGF Key Interactions												
**Residue**	Cys 10	Ser 11	Glu 12	Arg 13	Arg 14	Lys 15	Phe 18	Cys 27	Arg 39	Asn 44	Thr 47	Asp 51
**None**	73	3	10	7	15	68	7	53	60	58	23	64
**HB**	-	10	90	34	26	32	-	-	14	-	-	-
**Hyd**	27	6	-	-	-	-	93	47	-	42	77	36
**Hyd+HB**	-	81	-	4	-	-	-	-	-	-	-	-
**Ion**	-	-	-	1	1	-	-	-	3	-	-	-
**Ion+HB**	-	-	-	48	54	-	-	-	22	-	-	-
**Ion+Hyd**	-	-	-	-	-	-	-	-	-	-	-	-
**Ion+Hyd+HB**	-	-	-	6	2	-	-	-	-	-	-	-

Figure S72. Key interactions of ligand **35** to VEGF as the fraction of the 100 ns simulation the given interaction was present, including no interaction (none), hydrogen bonding (HB), hydrophobic (Hyd), ionic (Ion), and combinations of multiple interactions, interactions that were present in less than 1% of the simulation are shown using a dash.

Ligand 8 - FGF2 Key Interactions									
**Residue**	Tyr 24	Lys 26	Phe 31	Arg 44	Tyr 103	Lys 135	Leu 140	Met 142	
**None**	5	32	72	76	30	75	29	61	
**HB**	20	1	-	3	20	16	-	5	
**Hyd**	12	8	28	-	15	6	71	33	
**Hyd+HB**	62	-	-	-	35	2	-	1	
**Ion**	-	12	-	3	-	-	-	-	
**Ion+HB**	-	40	-	18	-	1	-	-	
**Ion+Hyd**	-	5	-	-	-	-	-	-	
**Ion+Hyd+HB**	-	1	-	-	-	-	-	-	

Figure S73. Key interactions of ligand **8** to FGF2 as the fraction of the 100 ns simulation the given interaction was present, including no interaction (none), hydrogen bonding (HB), hydrophobic (Hyd), ionic (Ion), and combinations of multiple interactions, interactions that were present in less than 1% of the simulation are shown using a dash.

Ligand 1 - FGF2 Key Interactions				
**Residue**	Arg 33	Arg 39	Asp 41	Gln 123
**None**	40	20	12	50
**HB**	59	40	87	44
**Hyd**	-	3	-	3
**Hyd+HB**	-	32	1	3
**Ion**	-	-	-	-
**Ion+HB**	-	1	-	-
**Ion+Hyd**	-	-	-	-
**Ion+Hyd+HB**	-	3	-	-

Figure S74. Key interactions of Ligand **1** to FGF2 as the fraction of the 100 ns simulation the given interaction was present, including no interaction (none), hydrogen bonding (HB), hydrophobic (Hyd), ionic (Ion), and combinations of multiple interactions, interactions that were present in less than 1% of the simulation are shown using a dash.

Ligand 16 - FGF2 Key Interactions					
**Residue**	Arg 39	Arg 44	Arg 120	Arg 123	Arg 135
**None**	79	78	35	80	44
**HB**	8	8	9	3	1
**Hyd**	-	-	-	13	7
**Hyd+HB**	-	-	-	4	19
**Ion**	-	-	4	-	-
**Ion+HB**	12	13	50	-	4
**Ion+Hyd**	-	-	-	-	-
**Ion+Hyd+HB**	-	-	2	-	24

Figure S75. Key interactions of Ligand **16** to FGF2 as the fraction of the 100 ns simulation the given interaction was present, including no interaction (none), hydrogen bonding (HB), hydrophobic (Hyd), ionic (Ion), and combinations of multiple interactions, interactions that were present in less than 1% of the simulation are shown using a dash.

Ligand 37 - FGF2 Key Interactions					
**Residue**	His 16	Phe 17	Lys 21	Ala 57	Arg 60
**None**	49	2	52	79	54
**HB**	1	-	-	-	3
**Hyd**	1	98	-	21	2
**Hyd+HB**	-	-	-	-	6
**Ion**	3	-	23	-	1
**Ion+HB**	45	-	24	-	11
**Ion+Hyd**	-	-	-	-	2
**Ion+Hyd+HB**	1	-	-	-	20

Figure S76. Key interactions of Ligand **37** to FGF2 as the fraction of the 100 ns simulation the given interaction was present, including no interaction (none), hydrogen bonding (HB), hydrophobic (Hyd), ionic (Ion), and combinations of multiple interactions, interactions that were present in less than 1% of the simulation are shown using a dash.

Ligand 1 - FGFR1 Interactions									
**Residue**	Lys 291	Glu 294	Lys 299	Phe 333	Glu 334	Ala 336	Glu 338	Thr 357	Val 358
**None**	12	4	44	77	6	29	23	68	46
**HB**	-	34	-	-	94	68	77	4	48
**Hyd**	-	6	-	23	-	-	-	24	-
**Hyd+HB**	-	56	-	-	-	3	-	4	6
**Ion**	47	-	25	-	-	-	-	-	-
**Ion+HB**	41	-	30	-	-	-	-	-	-
**Ion+Hyd**	-	-	-	-	-	-	-	-	-
**Ion+Hyd+HB**	-	-	-	-	-	-	-	-	-

Figure S77. Key interactions of Ligand **1** to FGFR1 as the fraction of the 100 ns simulation the given interaction was present, including no interaction (none), hydrogen bonding (HB), hydrophobic (Hyd), ionic (Ion), and combinations of multiple interactions, interactions that were present in less than 1% of the simulation are shown using a dash.

Ligand 8 - FGFR1 Key Interactions								
**Residue**	Lys 163	His 166	Val 168	Lys 172	Lys 175	Phe 176	Lys 177	His 286
**None**	43	59	66	22	7	76	22	70
**HB**	7	-	-	6	18	-	7	30
**Hyd**	3	41	34	3	-	24	-	-
**Hyd+HB**	44	-	-	-	-	-	1	-
**Ion**	1	-	-	11	4	-	9	-
**Ion+HB**	1	-	-	52	64	-	55	-
**Ion+Hyd**	-	-	-	1	-	-	1	-
**Ion+Hyd+HB**	1	-	-	4	6	-	4	-

Figure S78. Key interactions of Ligand **8** to FGFR1 as the fraction of the 100 ns simulation the given interaction was present, including no interaction (none), hydrogen bonding (HB), hydrophobic (Hyd), ionic (Ion), and combinations of multiple interactions, interactions that were present in less than 1% of the simulation are shown using a dash.

Ligand 16 - FGFR1 Key Interactions						
**Residue**	Lys 291	Glu 294	Lys 299	Lys 337	Glu 338	Trp 355
**None**	76	24	63	31	24	3
**HB**	-	68	-	-	76	-
**Hyd**	-	2	-	69	-	94
**Hyd+HB**	-	6	-	-	-	2
**Ion**	7	-	4	-	-	-
**Ion+HB**	17	-	32	-	-	-
**Ion+Hyd**	-	-	-	-	-	-
**Ion+Hyd+HB**	-	-	-	-	-	-

Figure S79. Key interactions of Ligand **16** to FGFR1 as the fraction of the 100 ns simulation the given interaction was present, including no interaction (none), hydrogen bonding (HB), hydrophobic (Hyd), ionic (Ion), and combinations of multiple interactions, interactions that were present in less than 1% of the simulation are shown using a dash.

Ligand 37 - FGFR1 Key Interactions												
**Residue**	Ile 293	Glu 294	Val 295	Gly 297	Tyr 307	Val 308	Gln 309	Phe 333	Glu 334	Ala 336	Trp 355	Glu 358
**None**	1	17	1	62	38	68	50	21	27	39	59	63
**HB**	-	53	-	-	-	32	-	-	-	-	-	-
**Hyd**	99	4	96	38	62	-	50	79	73	59	41	37
**Hyd+HB**	-	26	3	-	-	-	-	-	-	3	-	-
**Ion**	-	-	-	-	-	-	-	-	-	-	-	-
**Ion+HB**	-	-	-	-	-	-	-	-	-	-	-	-
**Ion+Hyd**	-	-	-	-	-	-	-	-	-	-	-	-
**Ion+Hyd+HB**	-	-	-	-	-	-	-	-	-	-	-	-

Figure S80. Key interactions of Ligand **37** to FGFR1 as the fraction of the 100 ns simulation the given interaction was present, including no interaction (none), hydrogen bonding (HB), hydrophobic (Hyd), ionic (Ion), and combinations of multiple interactions, interactions that were present in less than 1% of the simulation are shown using a dash.

NMR Spectral Data

^{1}H-NMR of **7** (600 MHz D_2O)

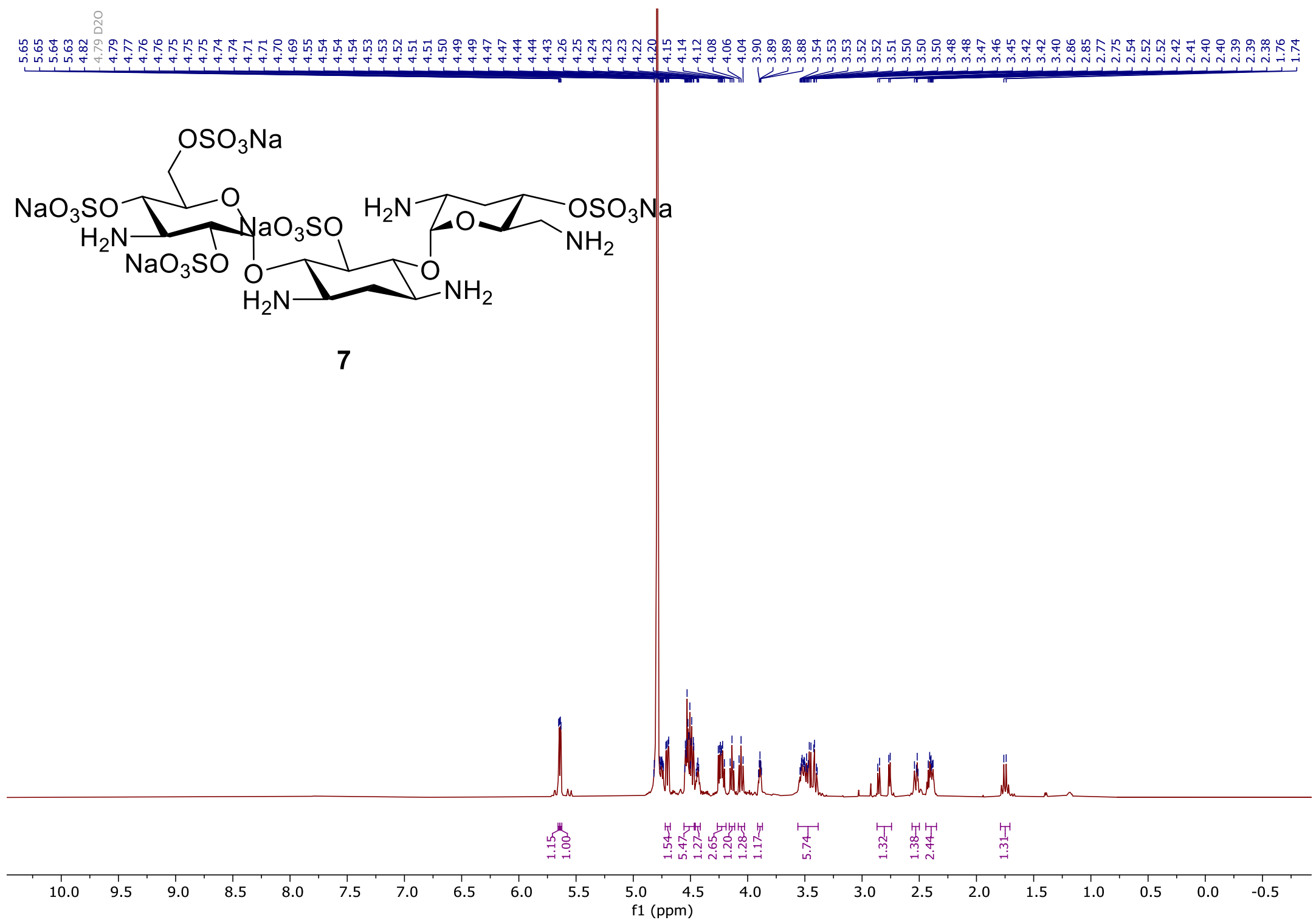

^{13}C-NMR of **7** (151 MHz D_2O)

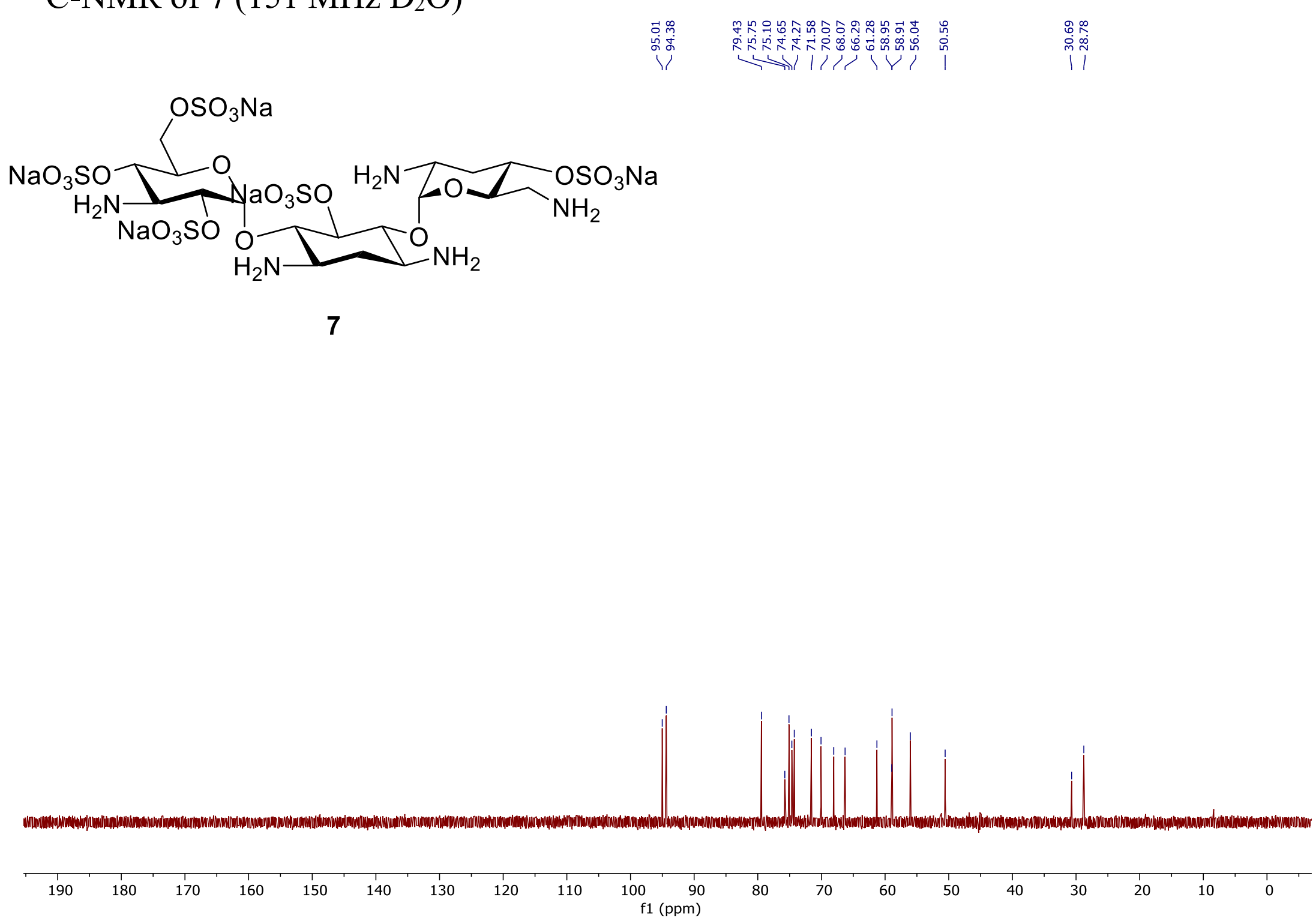

^{1}H-NMR of **8** (500 MHz CD_3OD)

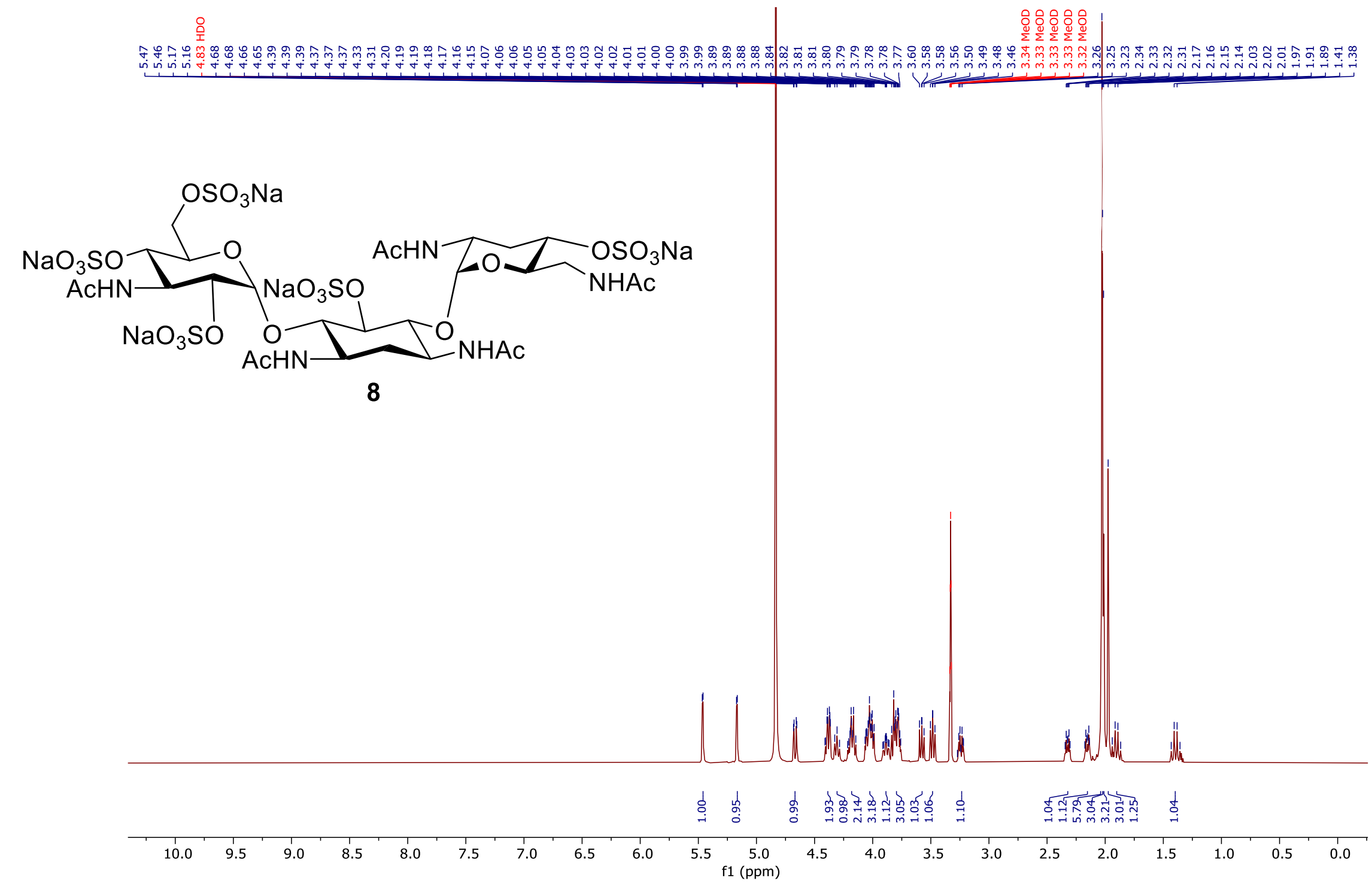

^{13}C-NMR of **8** (101 MHz CD_3OD)

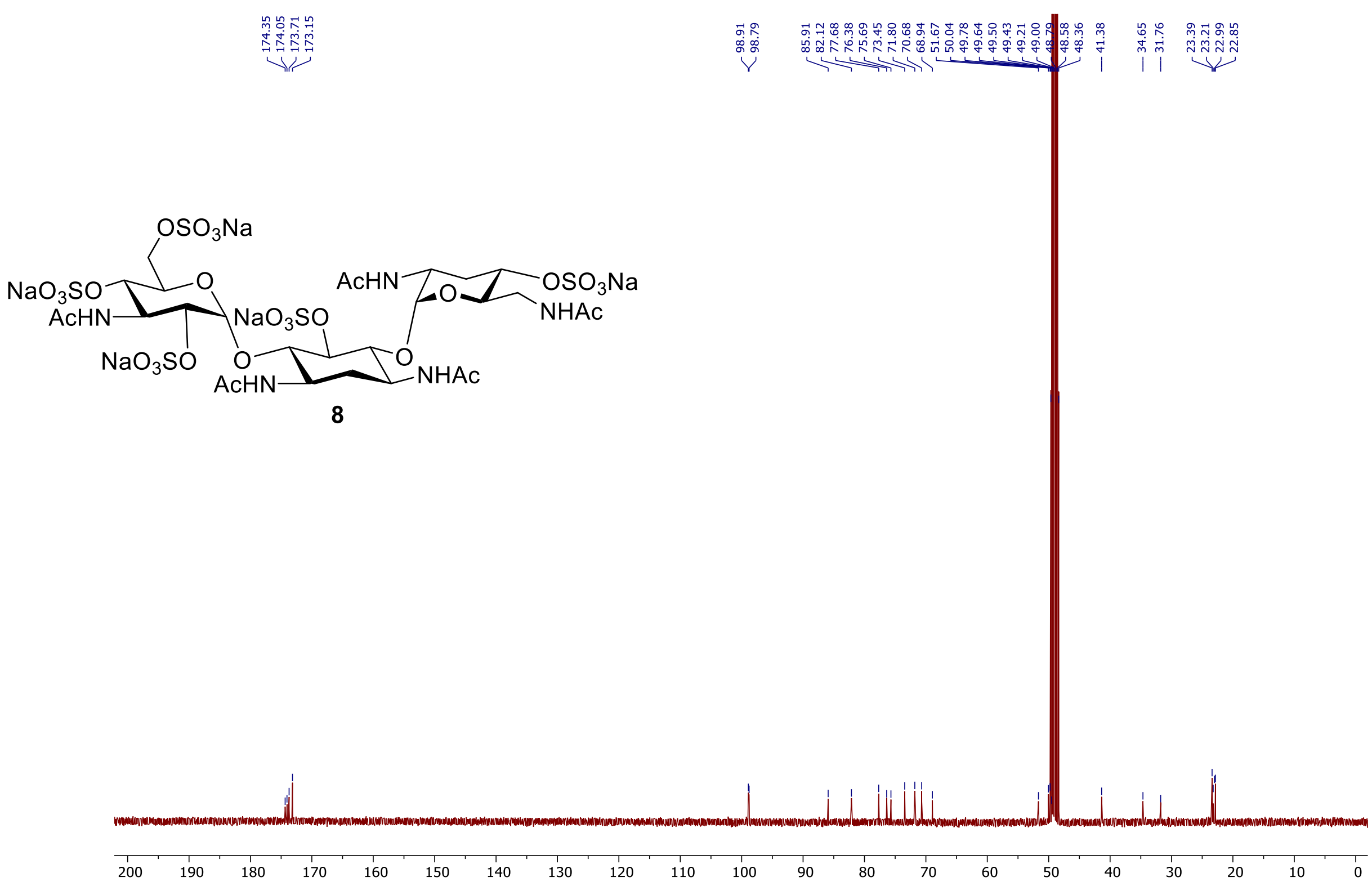

^{1}H-^{1}H COSY NMR of **8** (400 MHz CD_3OD)

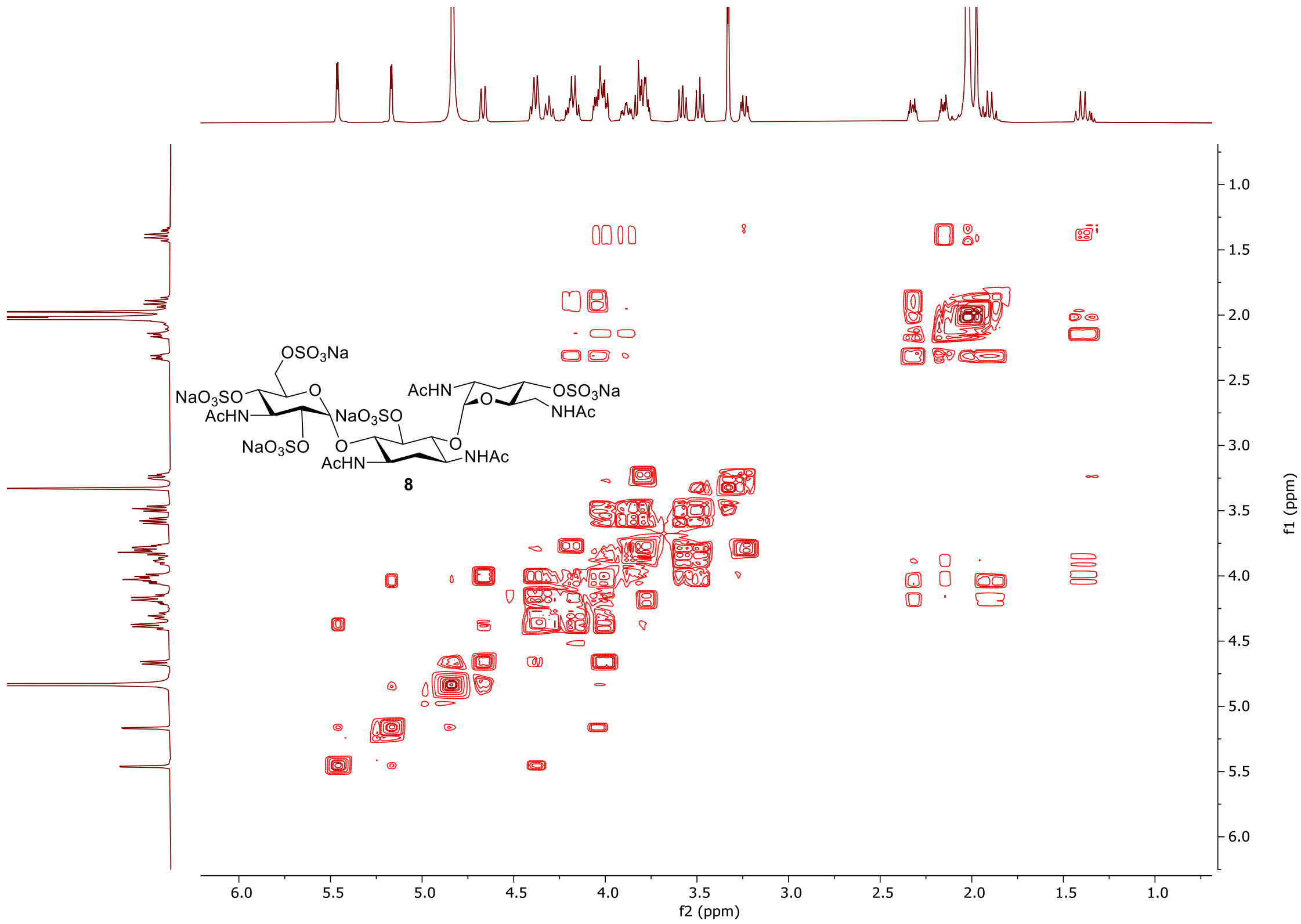

^{1}H-^{13}C HSQC NMR of **8** (500 MHz CD_3OD)

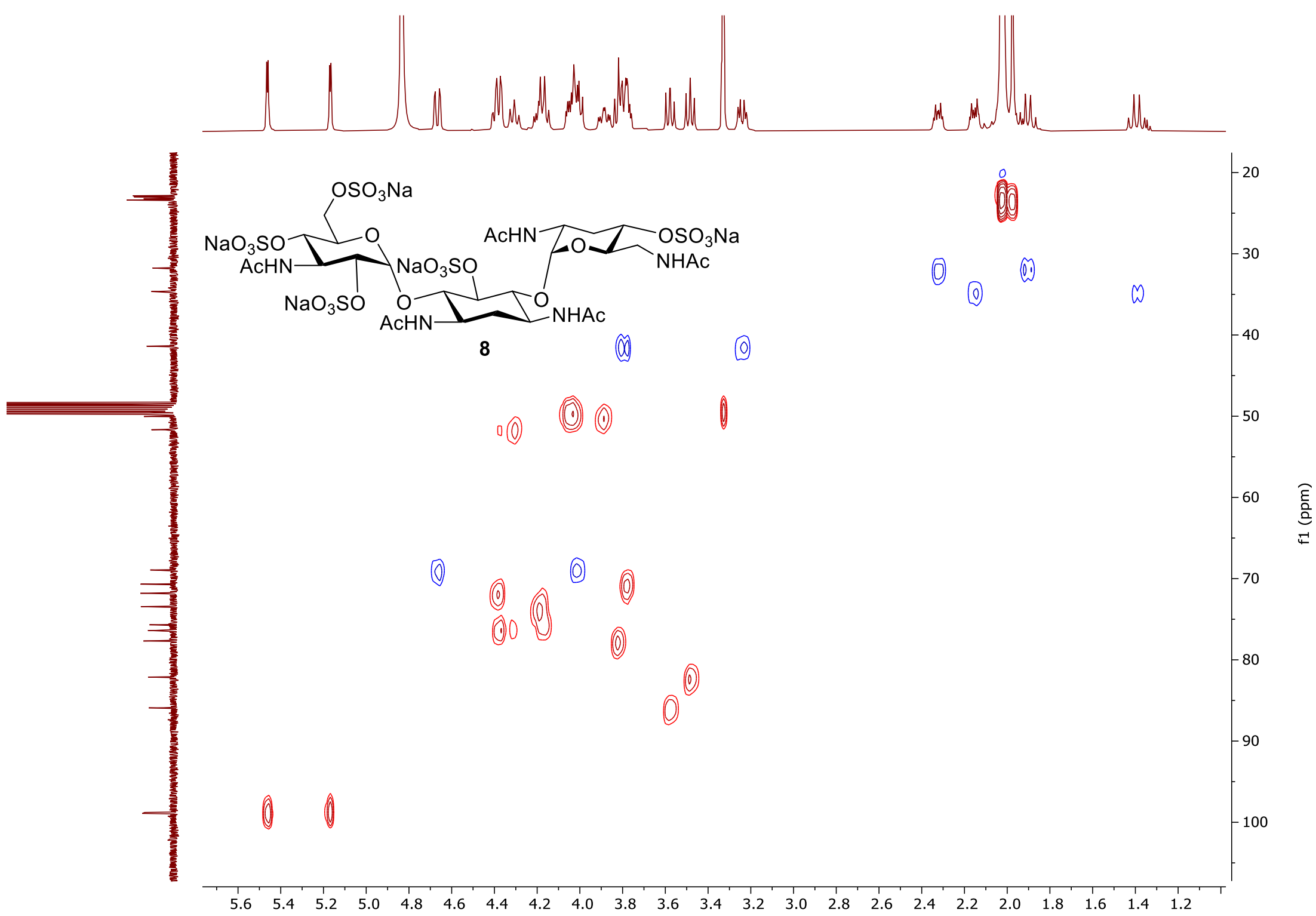

^{1}H-NMR of **9** (600 MHz D_2O)

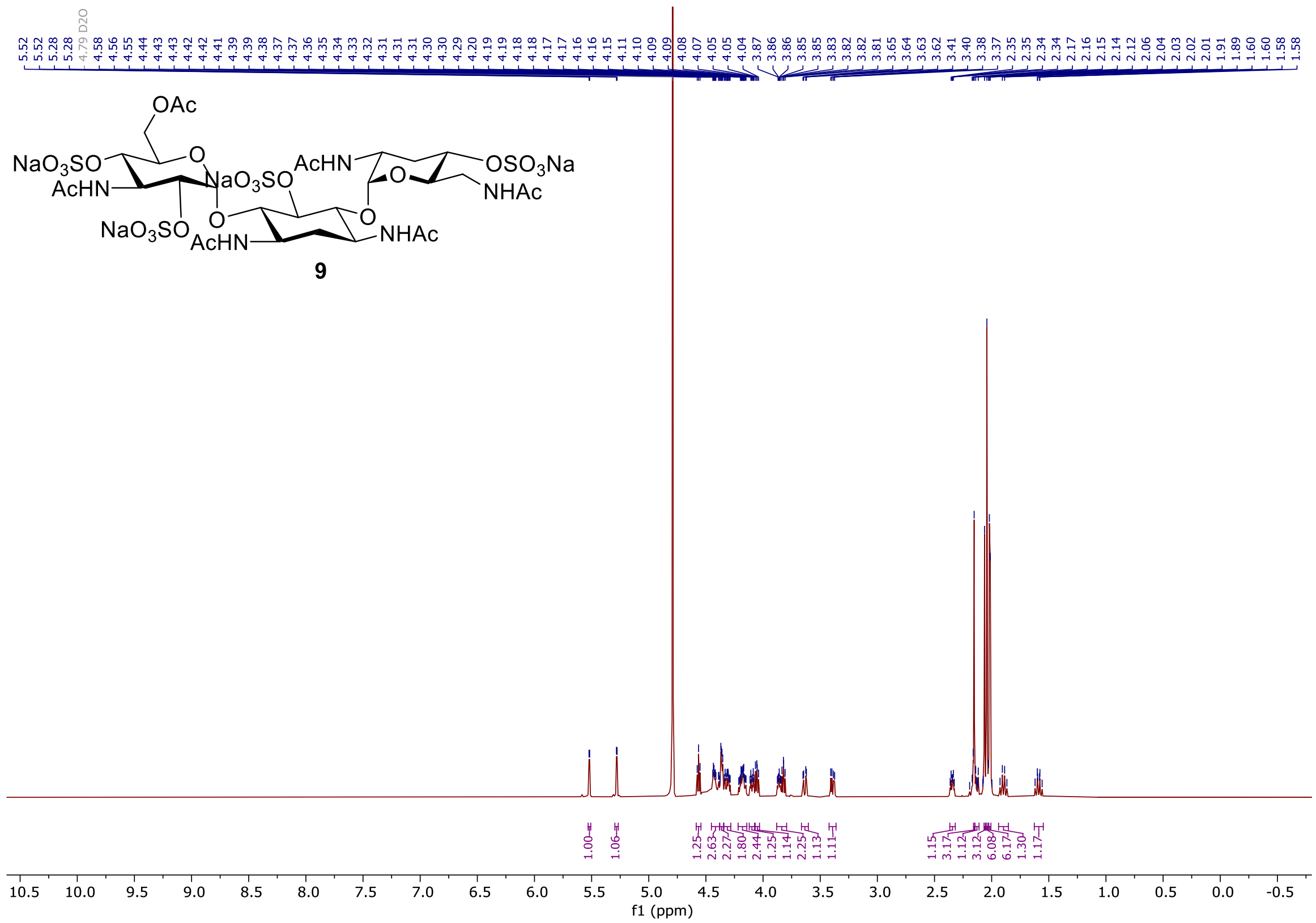

^{13}C-NMR of **9** (151 MHz D_2O)

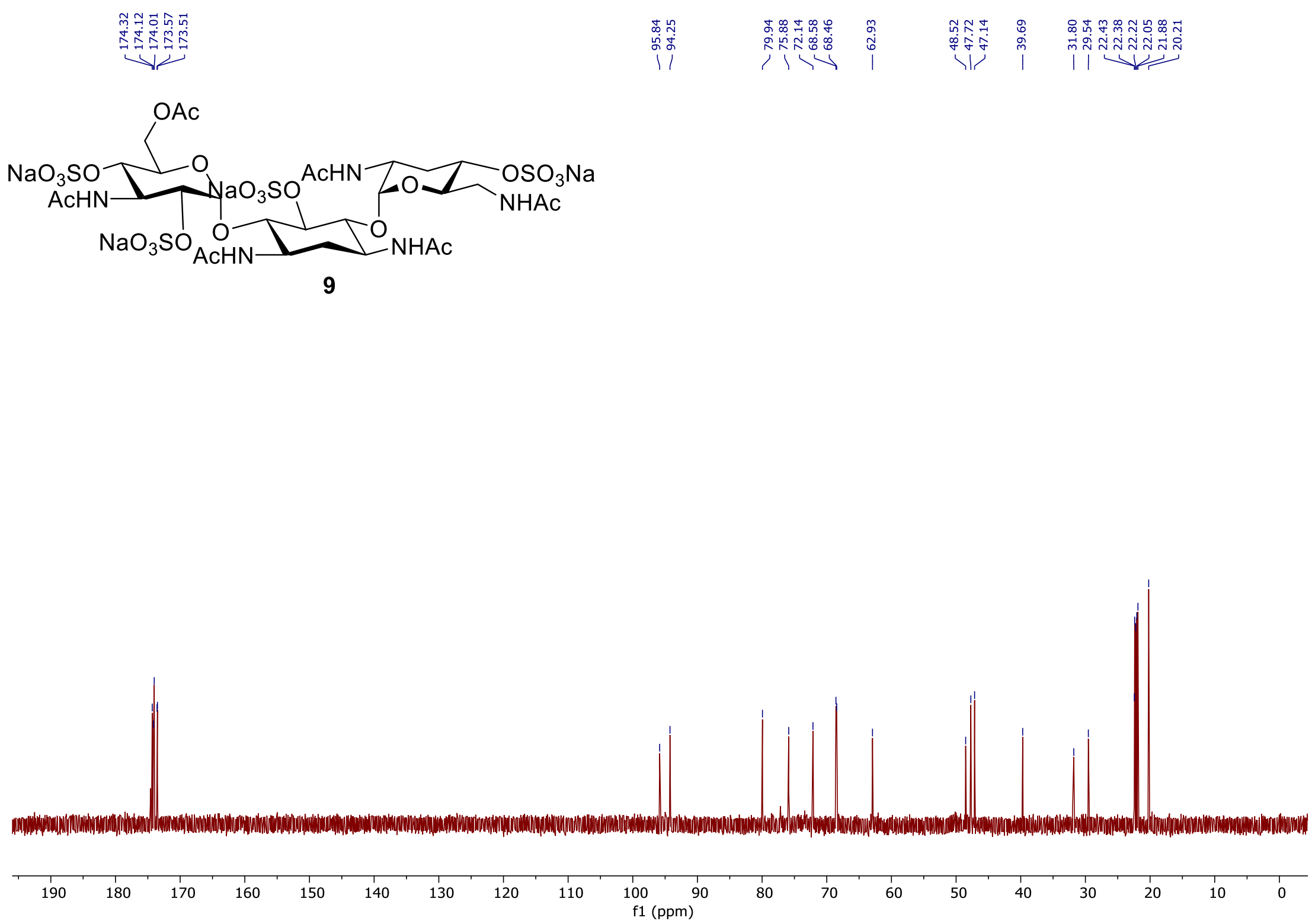

^{1}H-NMR of **10** (600 MHz D_2O)

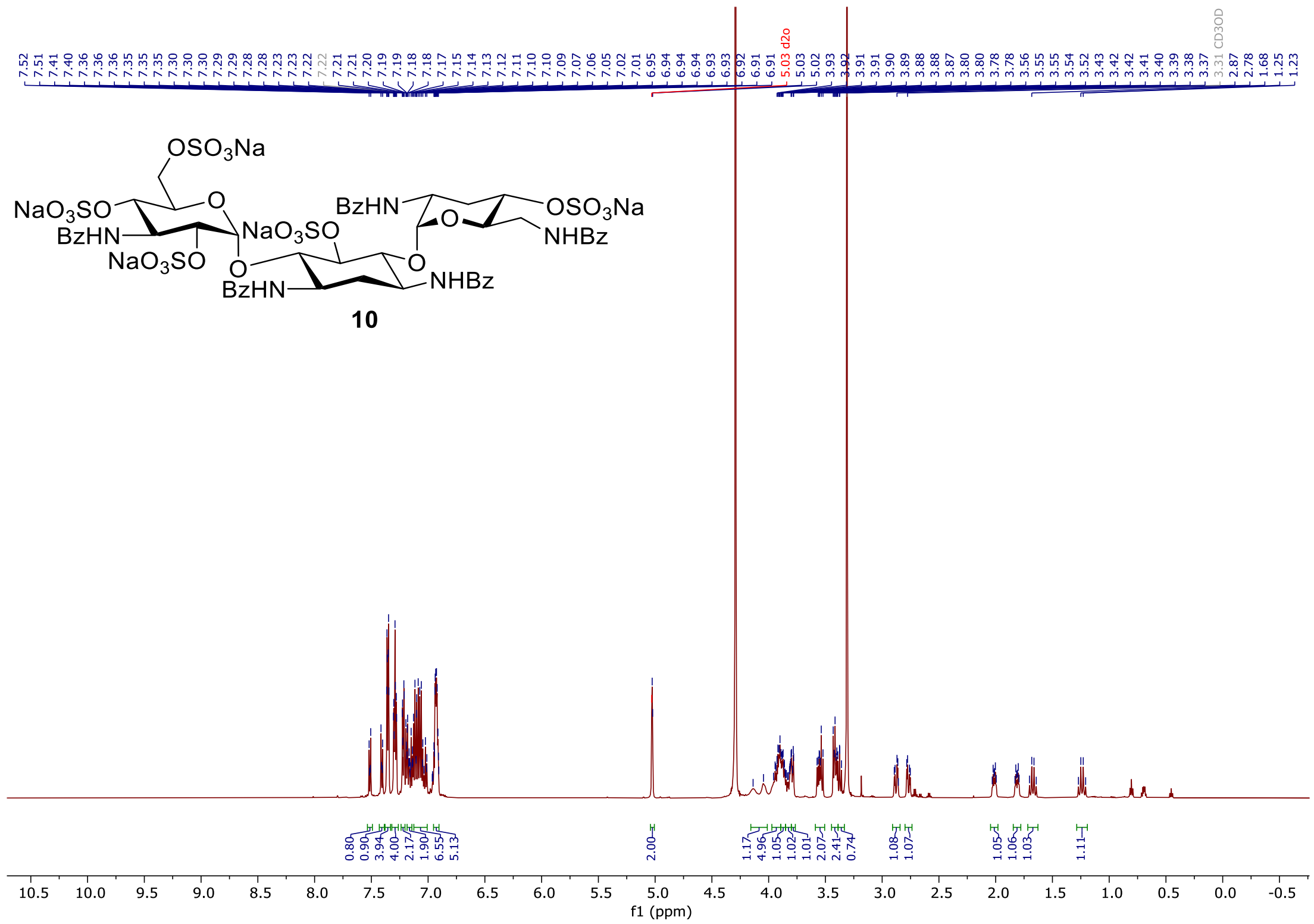

^{13}C-NMR of **10** (126 MHz D_2O)

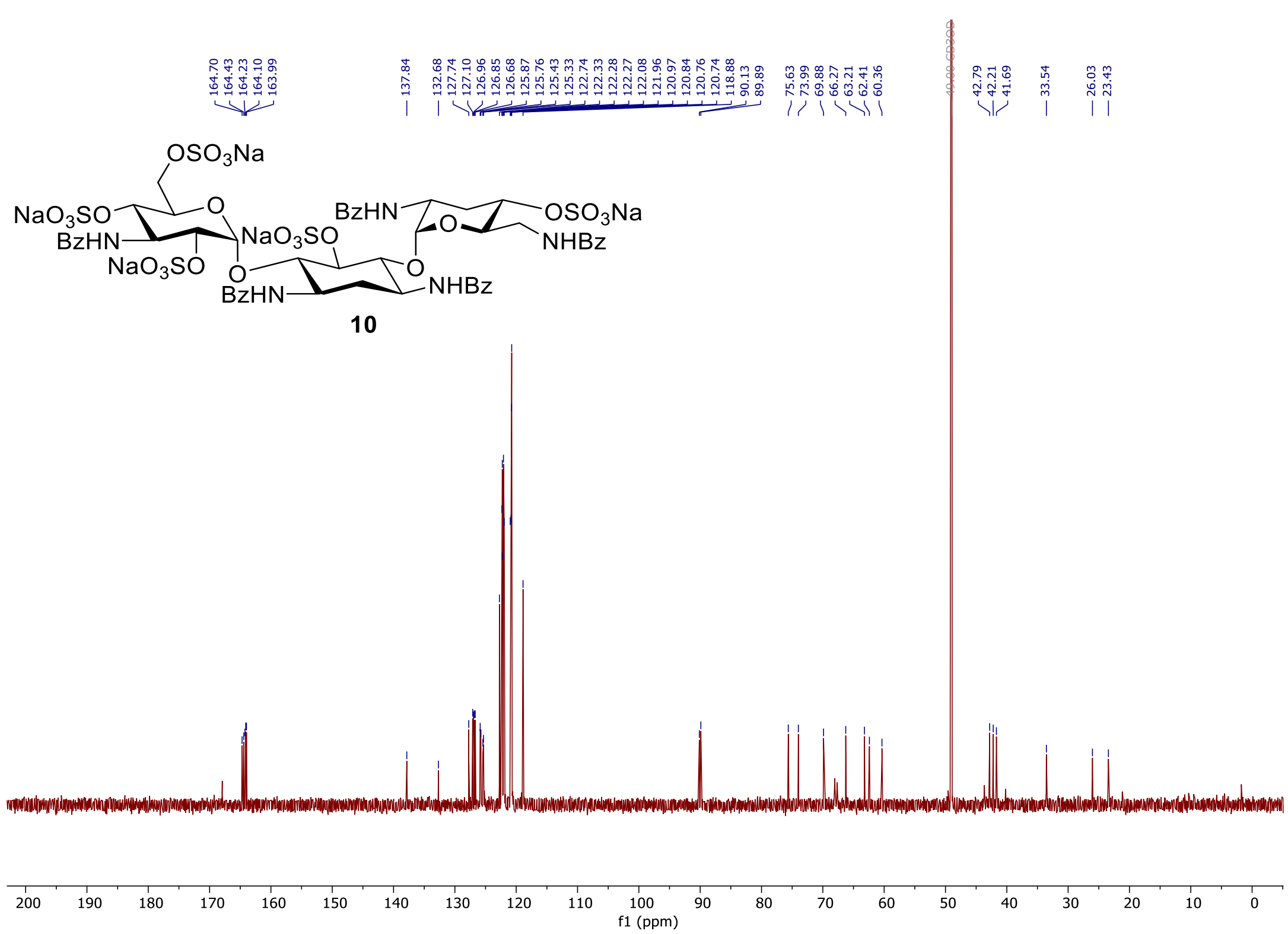

^{1}H-NMR of **11** (600 MHz D_2O)

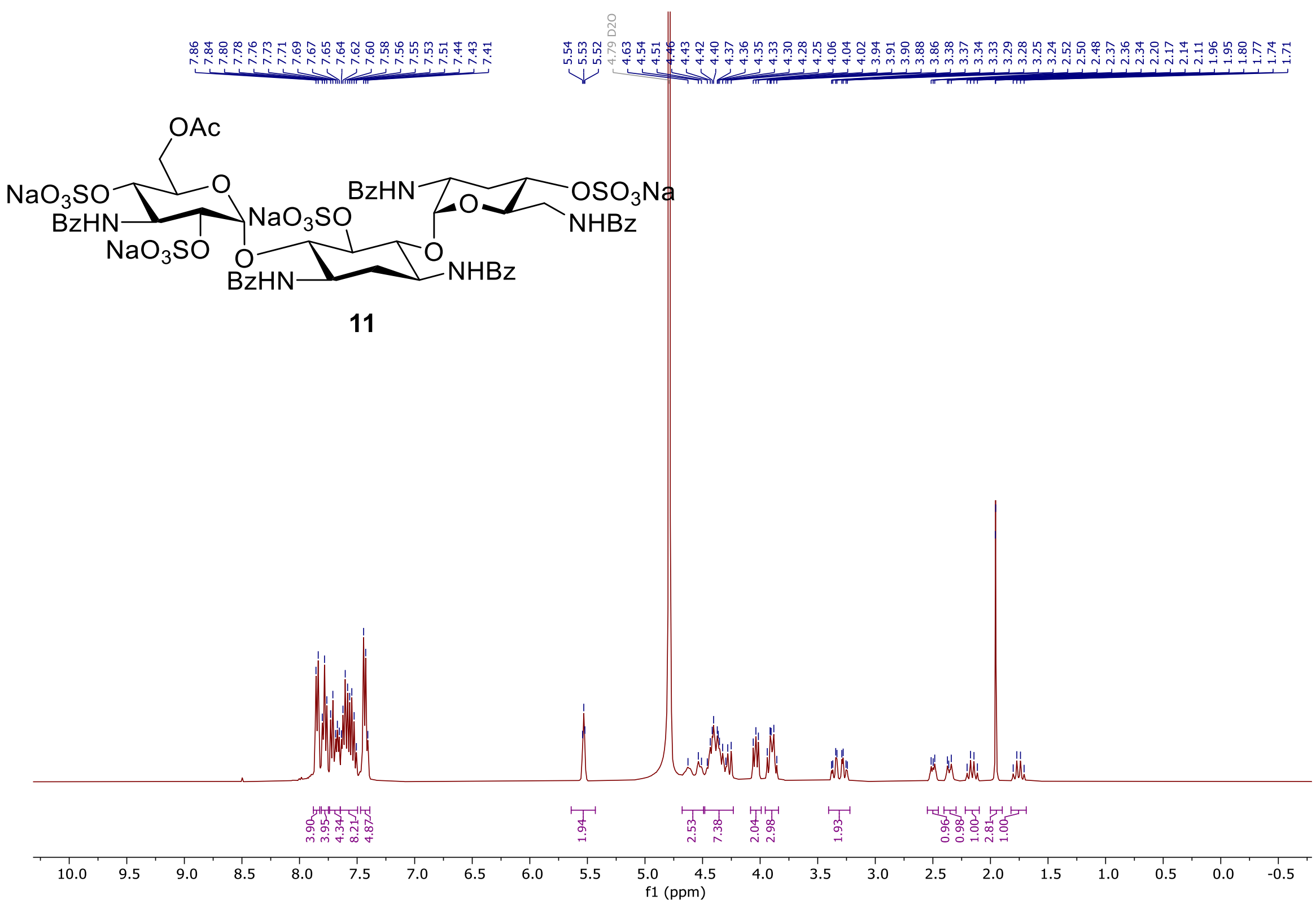

^{13}C-NMR of **11** (151 MHz, D_2O)

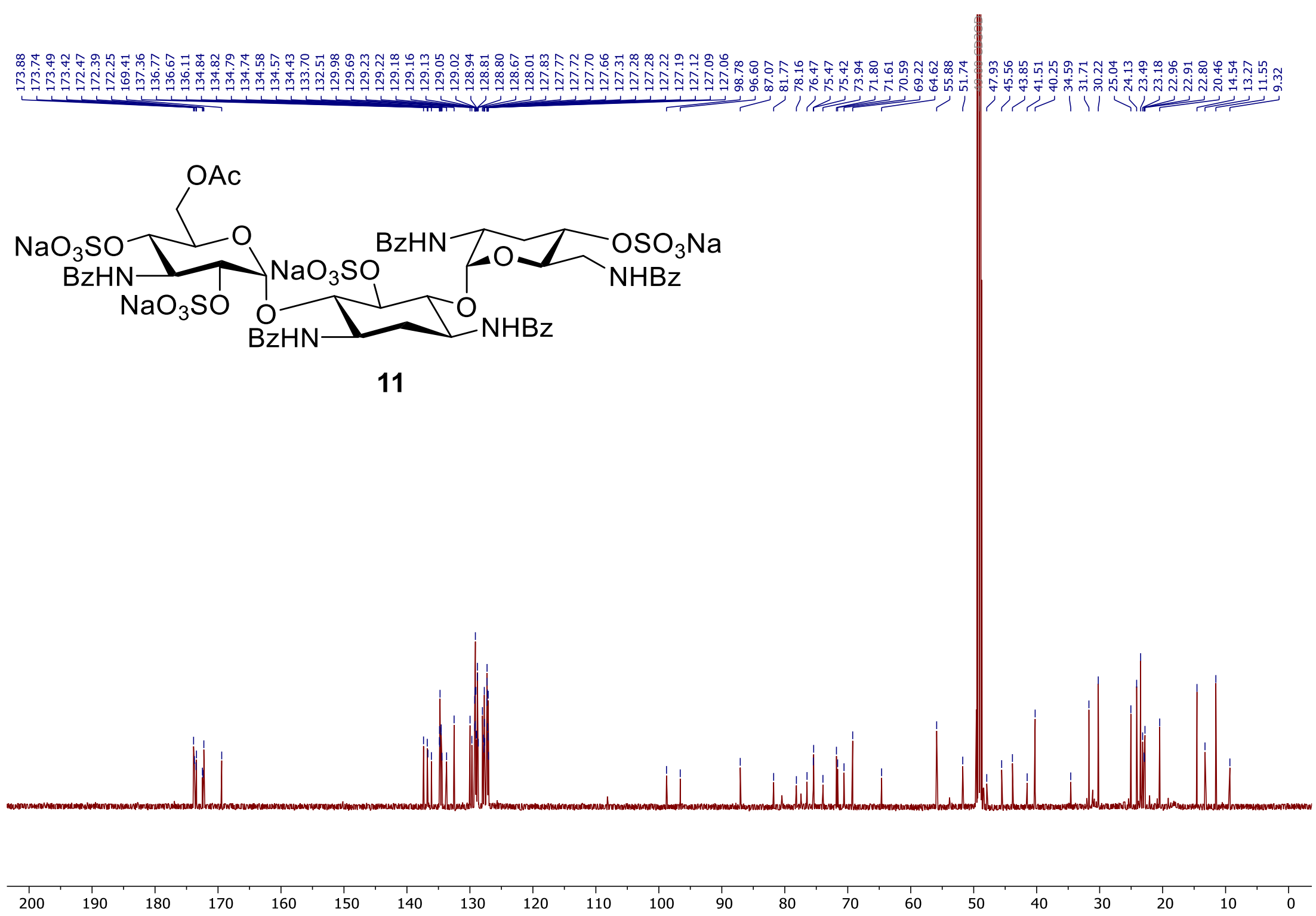

^{1}H-NMR of **12** (600 MHz D_2O)

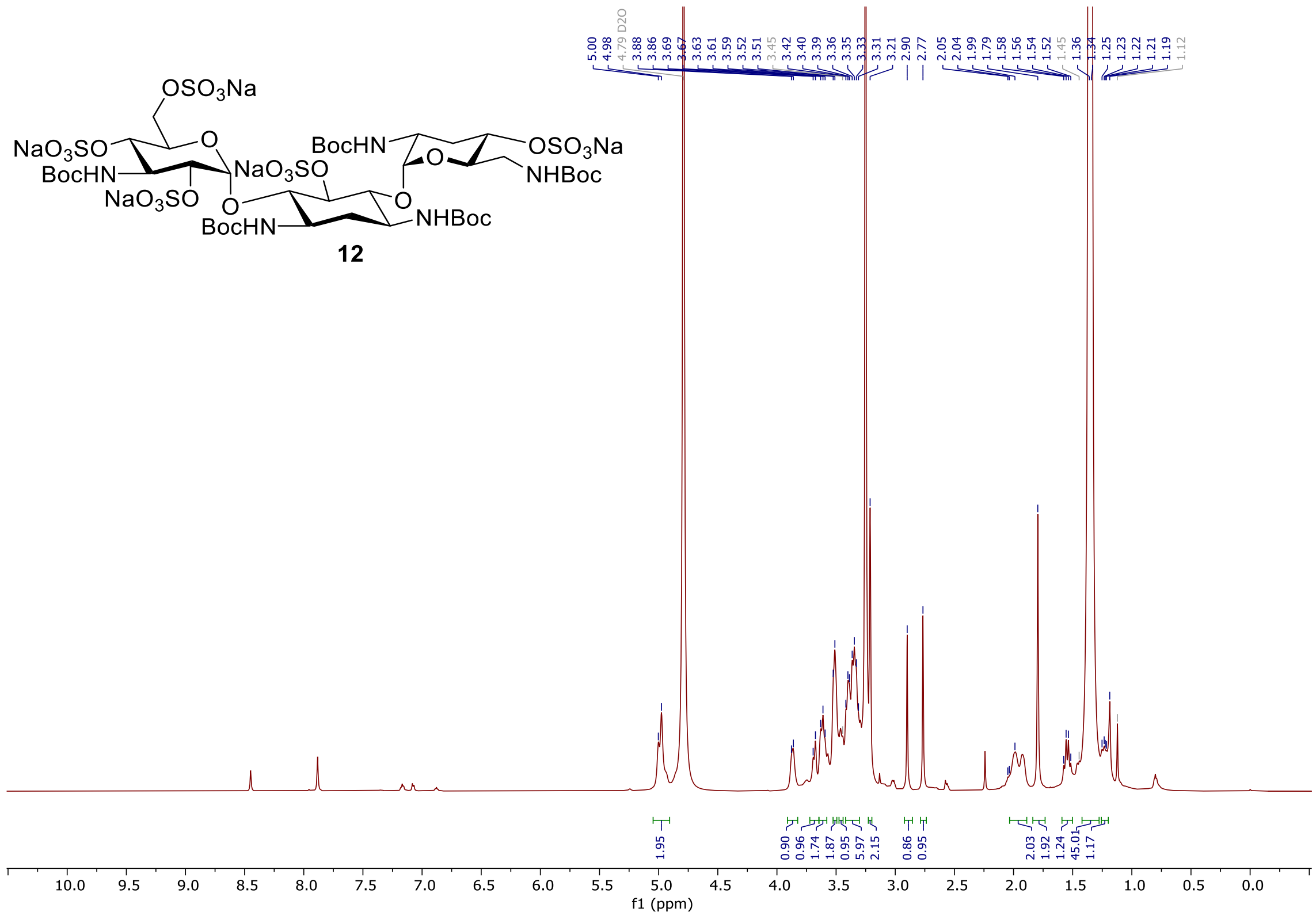

^{13}C-NMR of **12** (126 MHz D_2O)

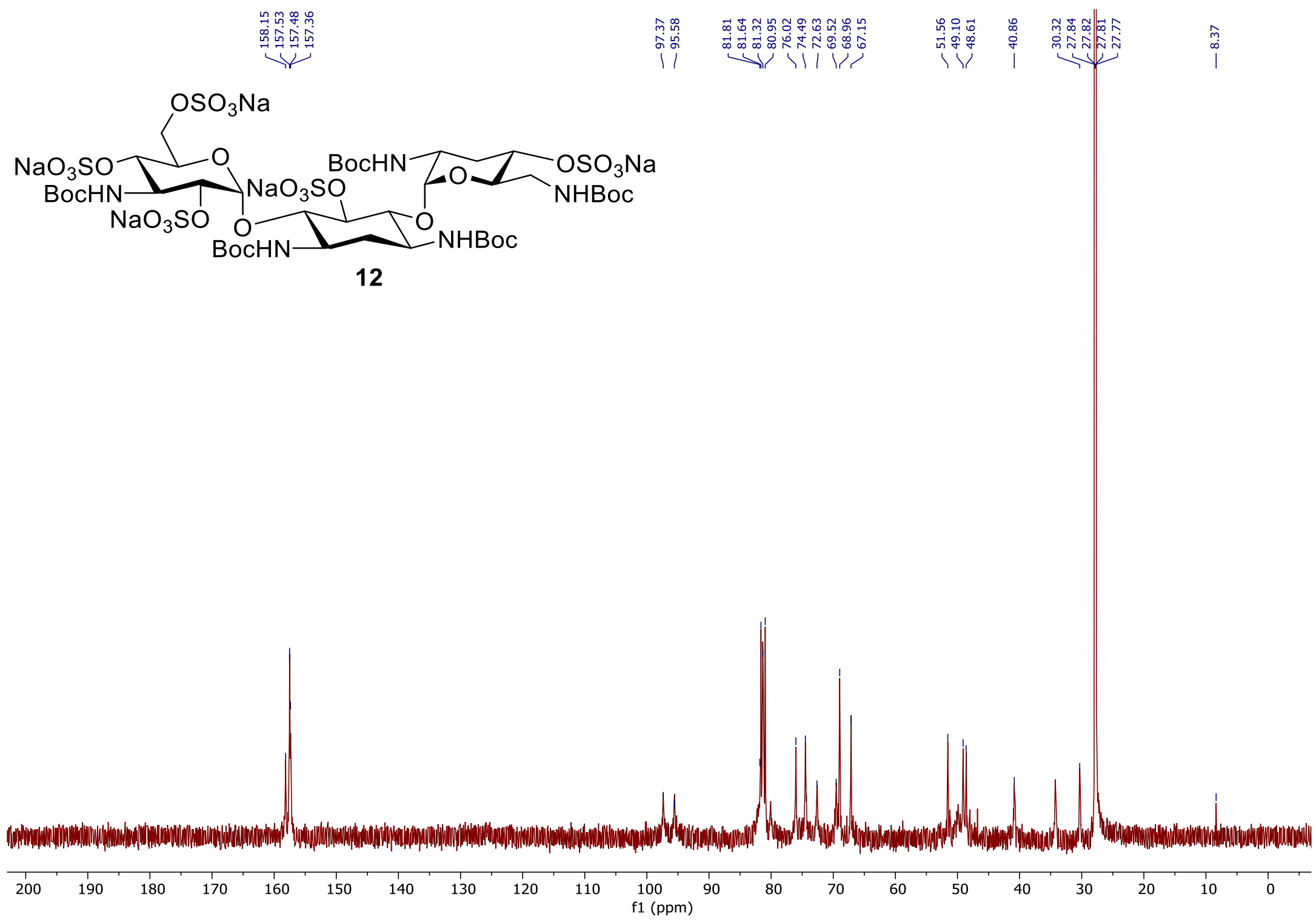

^{1}H-NMR of **13** (600 MHz D_2O)

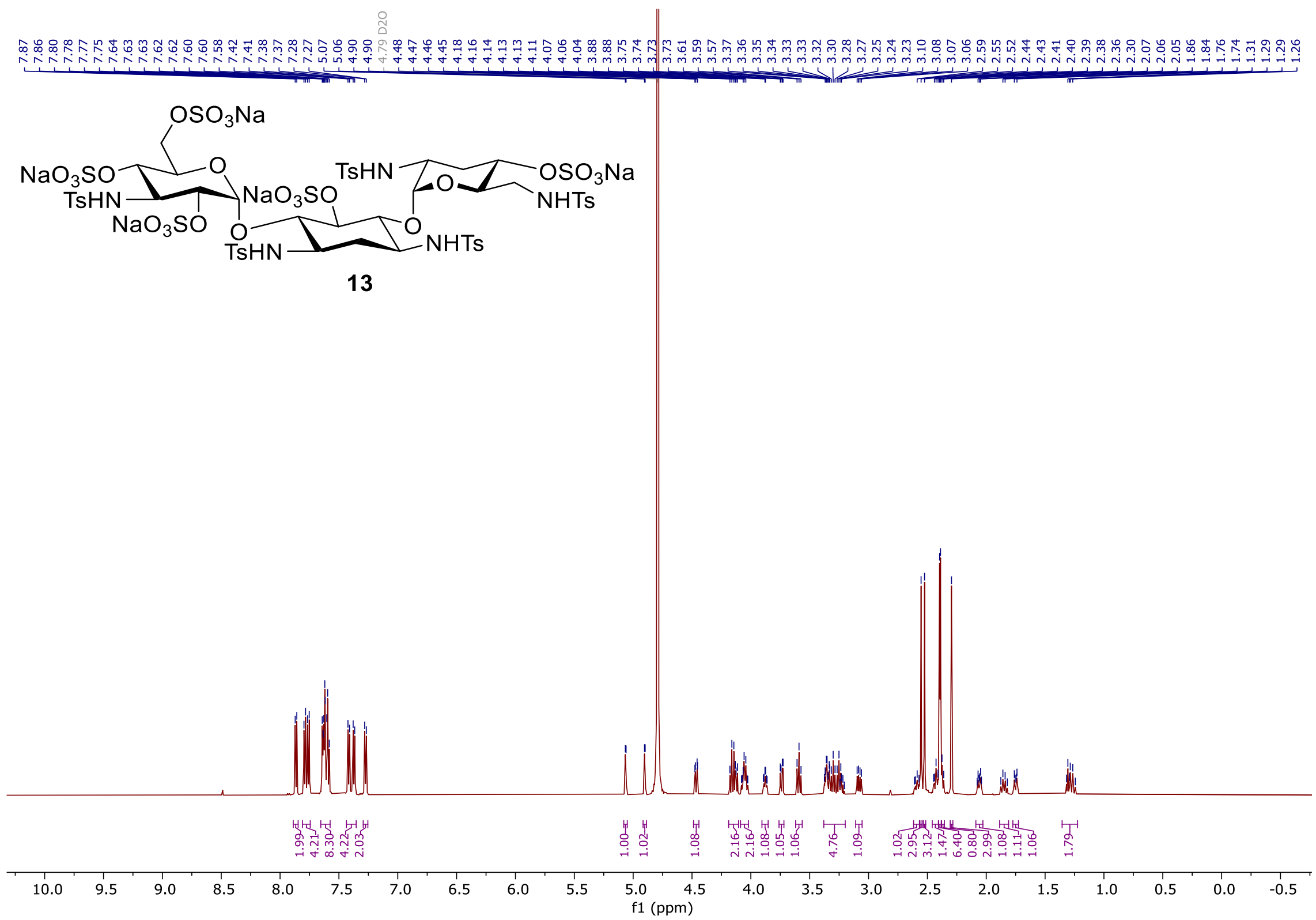

^{13}C-NMR of **13** (126 MHz D_2O)

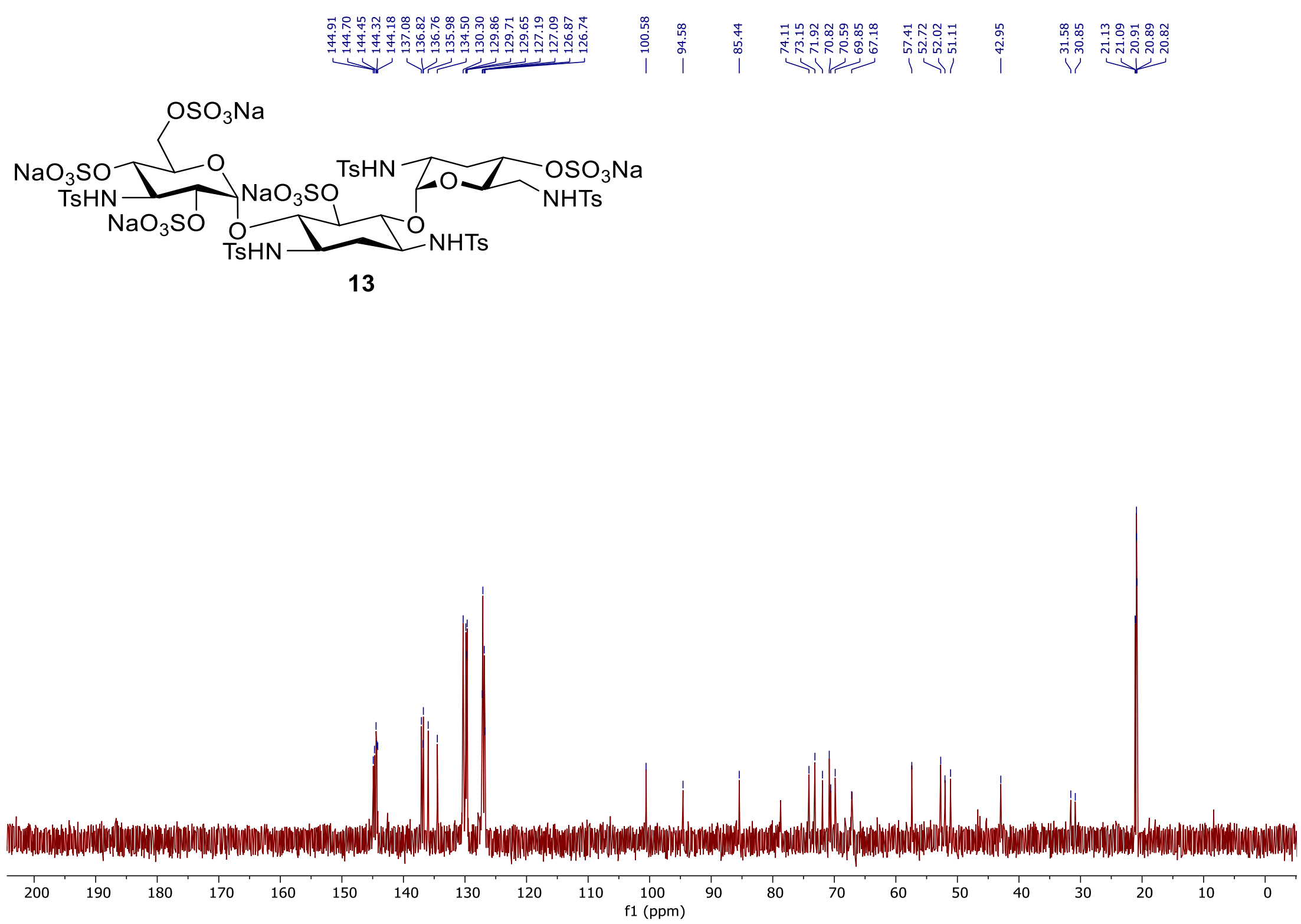

^{1}H-NMR of **14** (600 MHz D_2O)

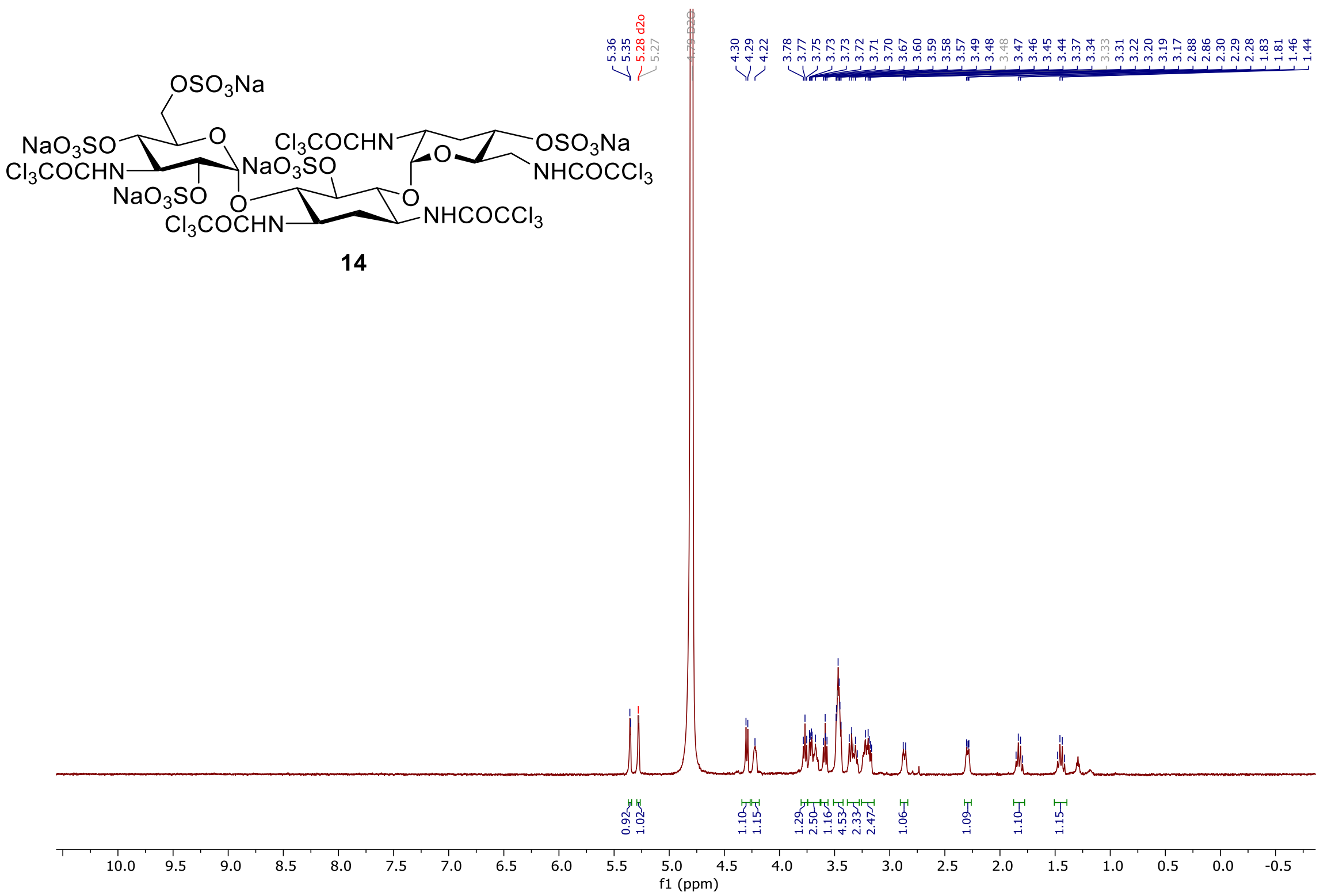

^{13}C-NMR of **14** (126 MHz D_2O)

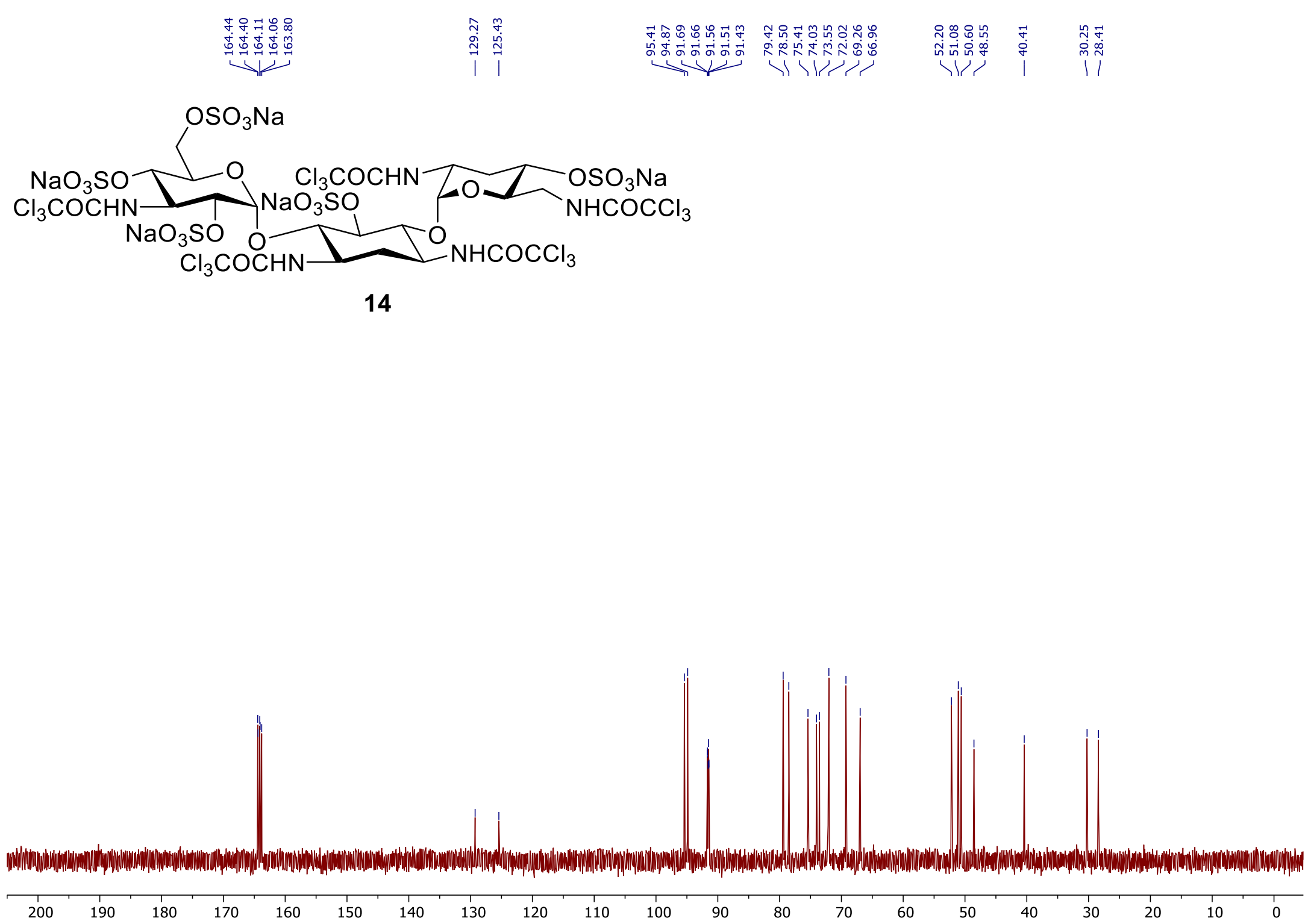

^{1}H-NMR of **15** (600 MHz D_2O)

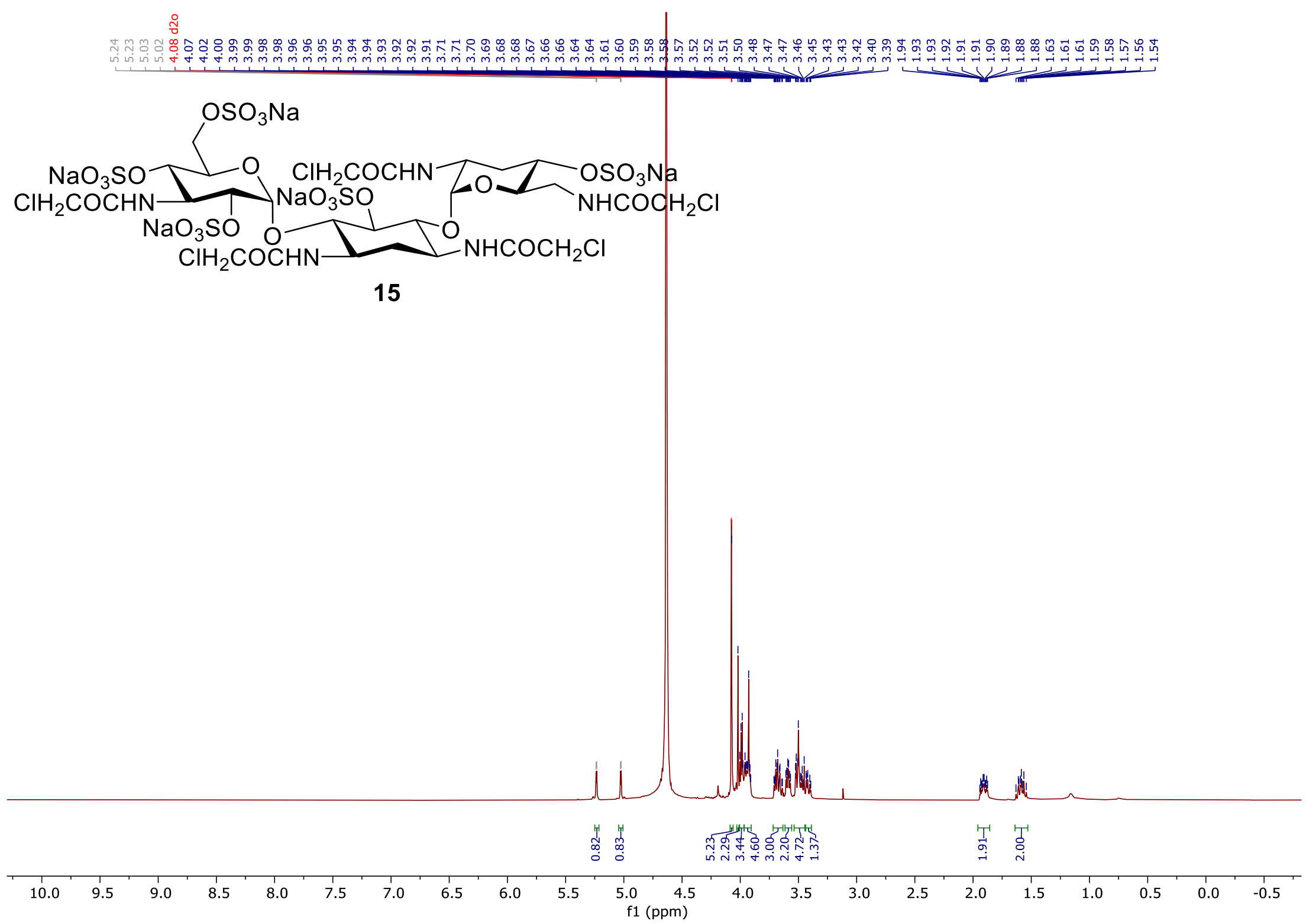

^{13}C-NMR of **15** (126 MHz D_2O)

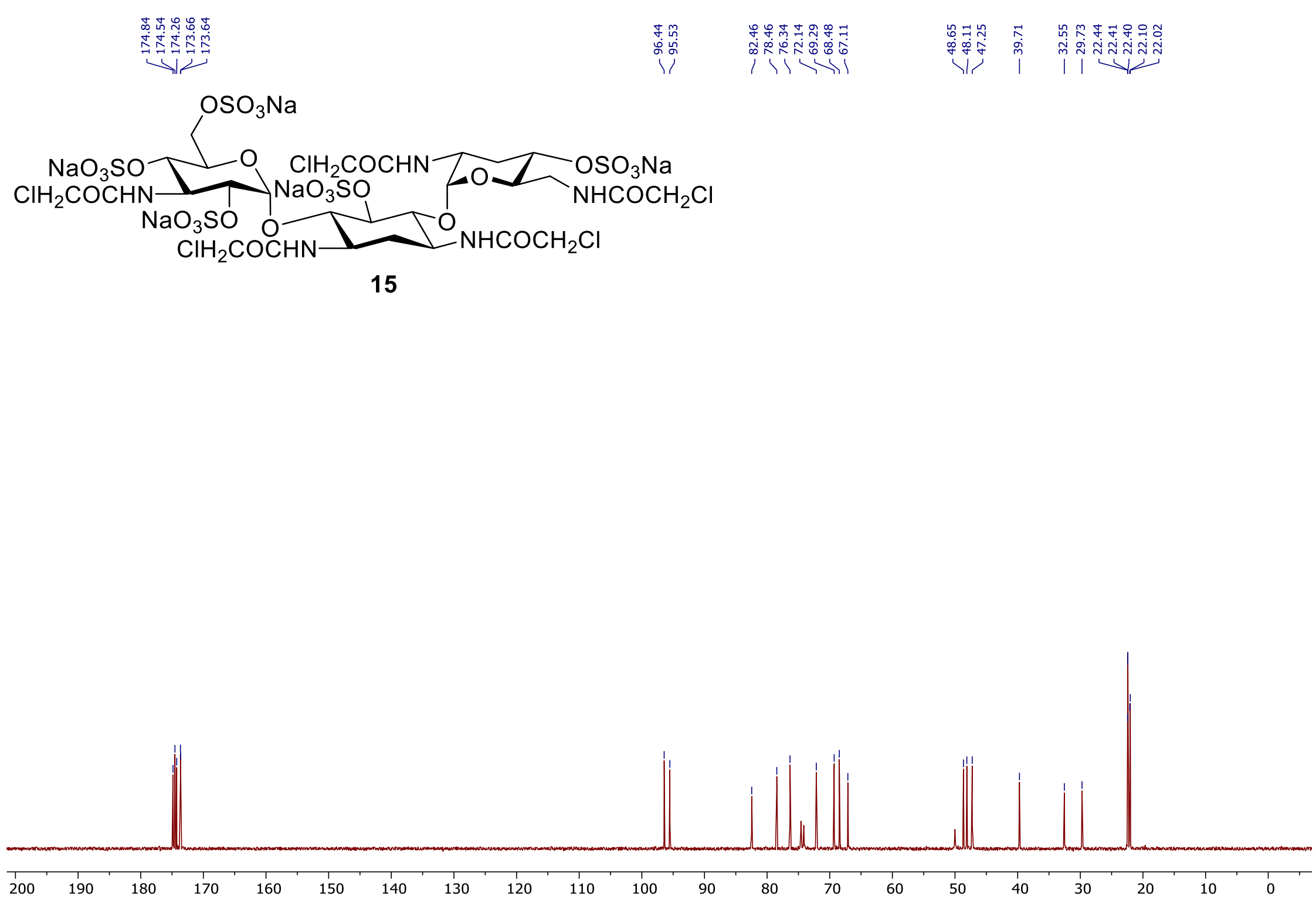

^{1}H-NMR of **48** (600 MHz $CDCl_3$)

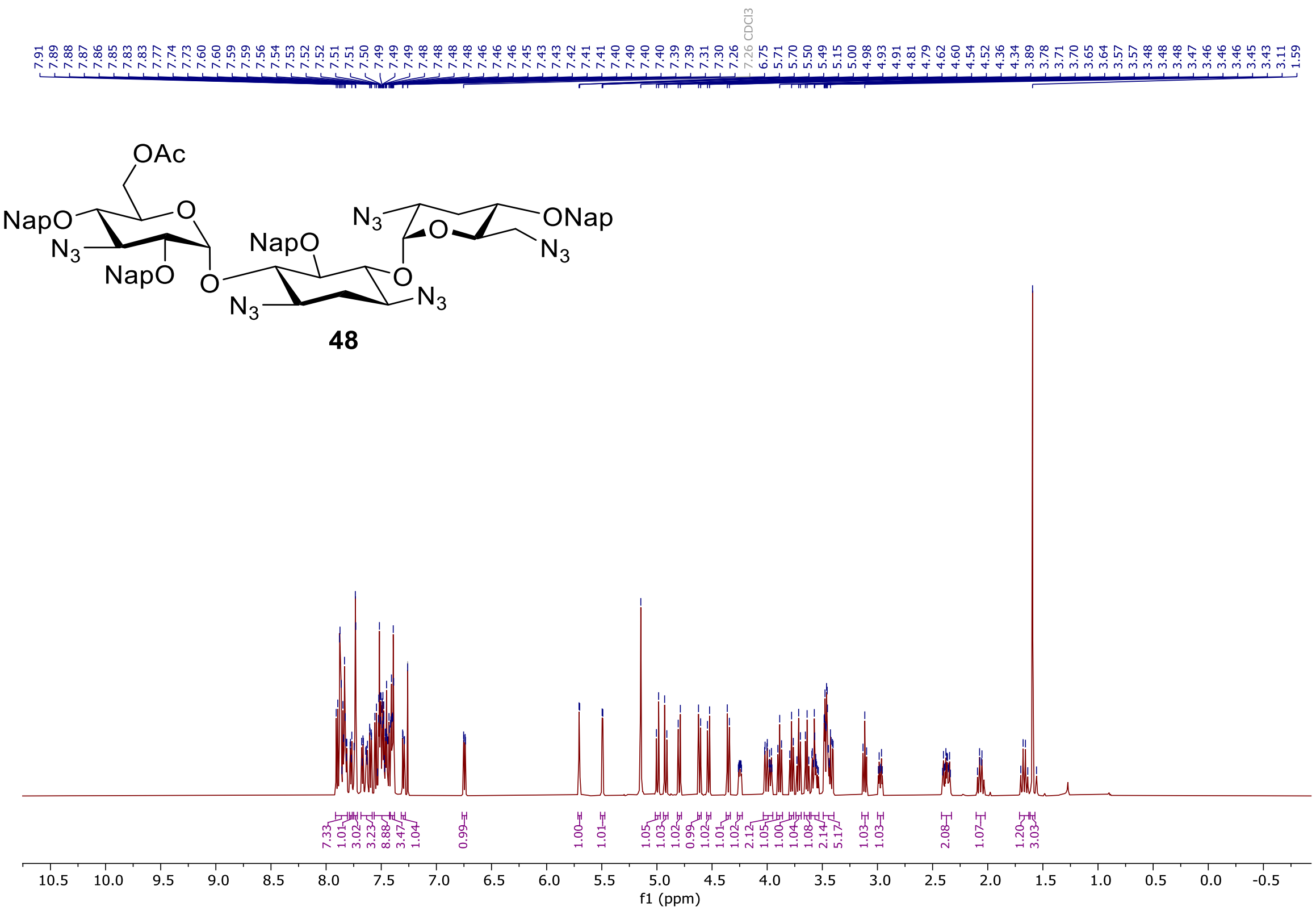

^{13}C-NMR of **iv** (151 MHz $CDCl_3$)

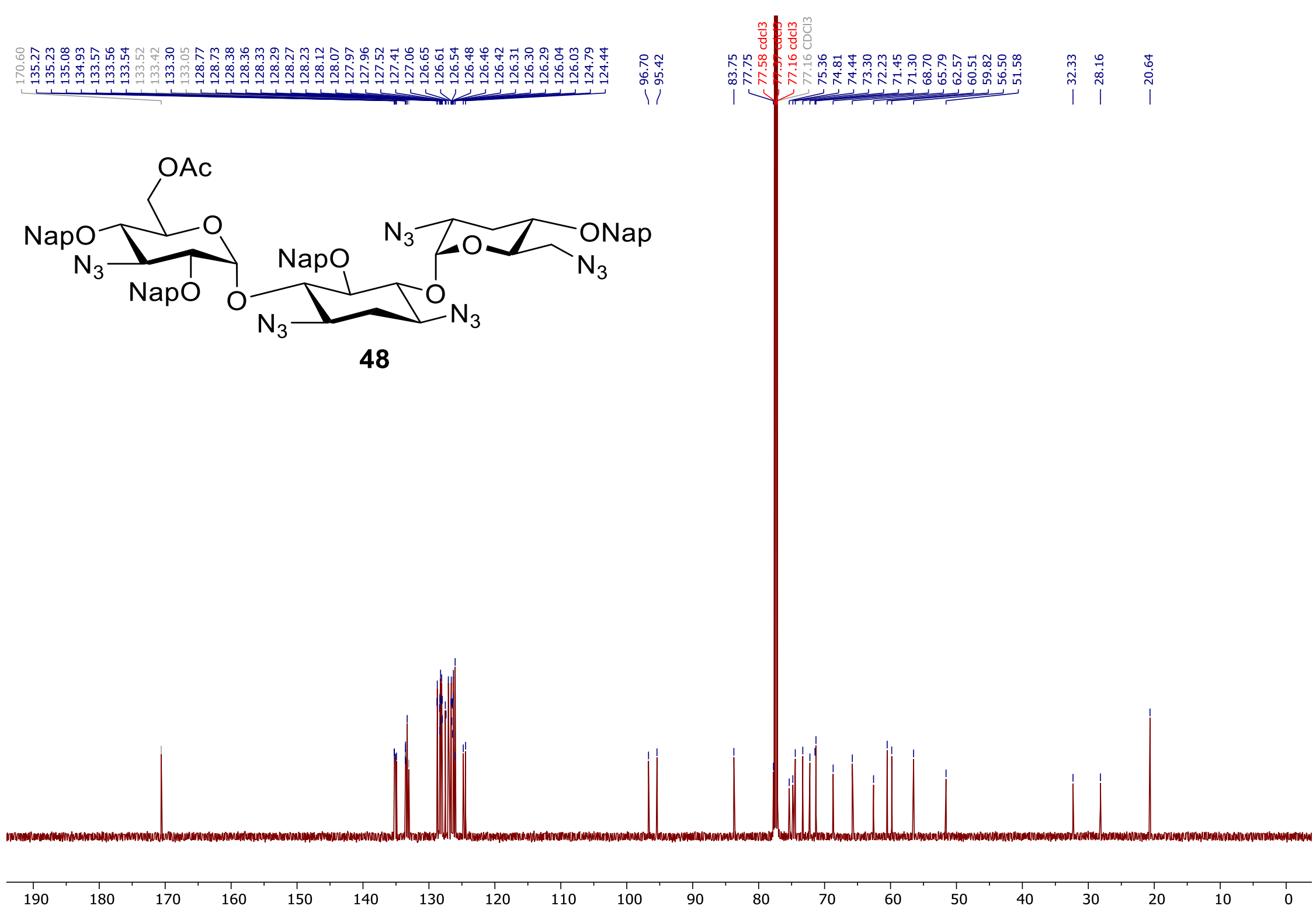

^{1}H-NMR of **49** (600 MHz D_2O)

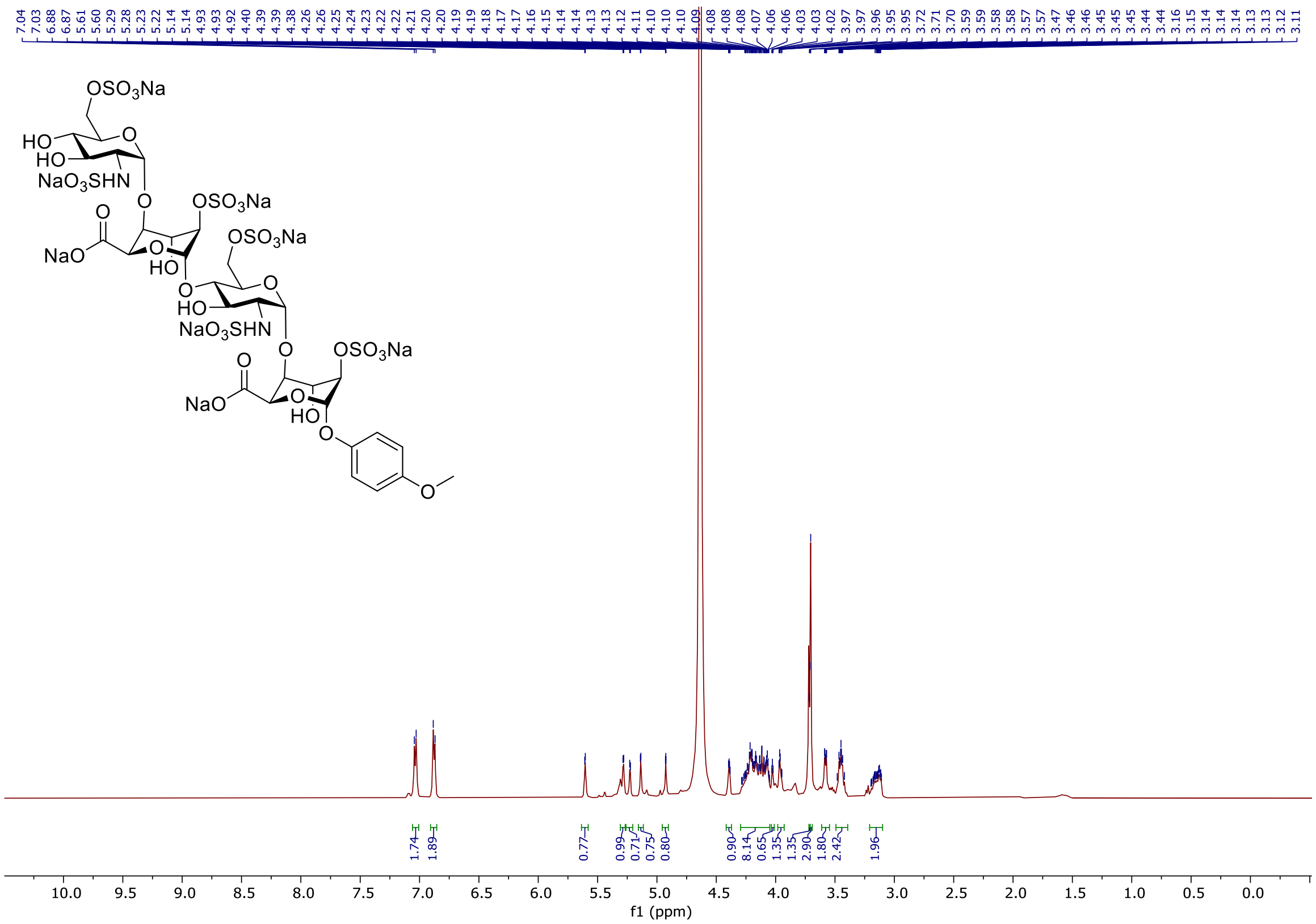

^{13}C NMR of **49** (151 MHz, D_2O)

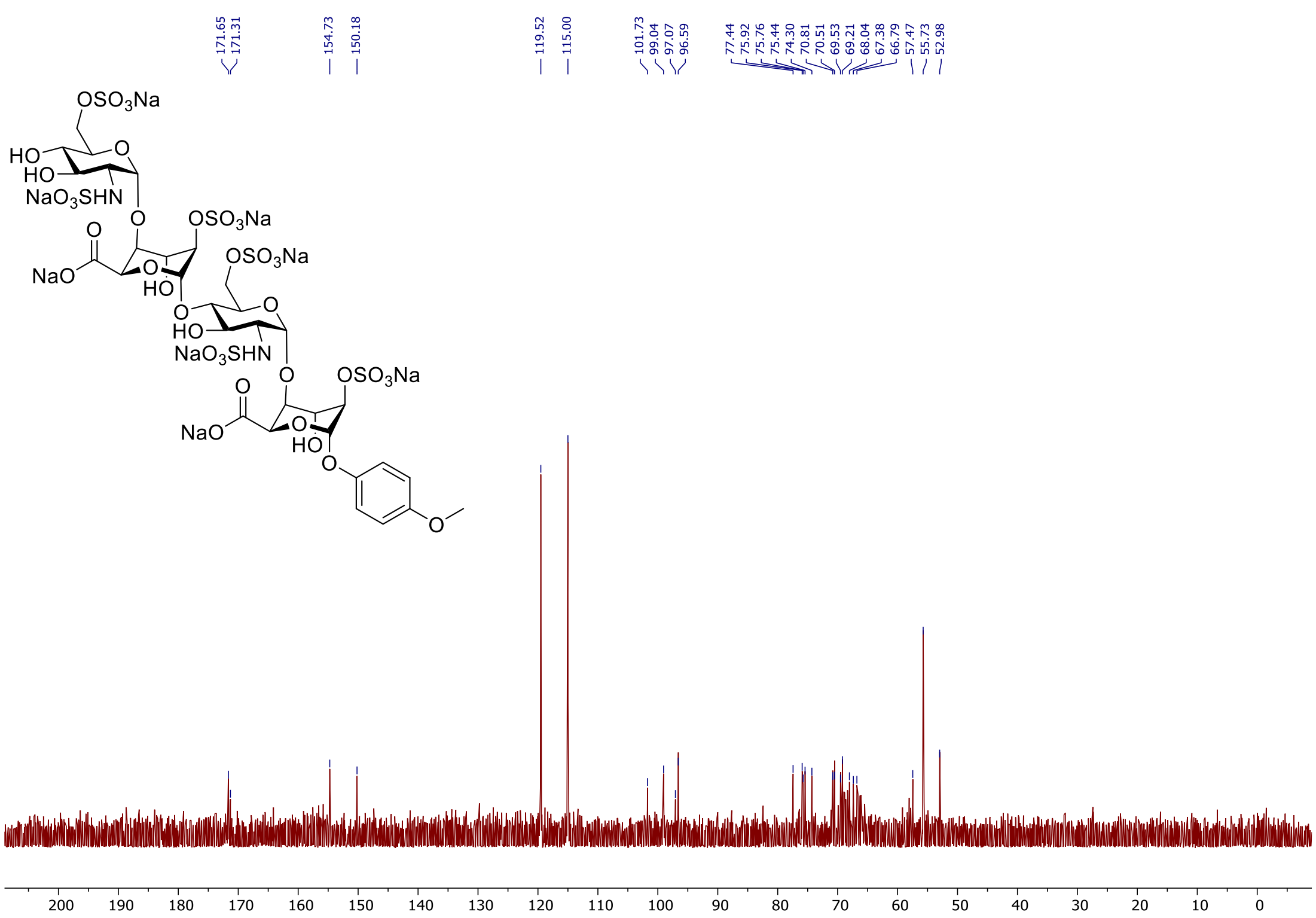

HSQC NMR of **49**

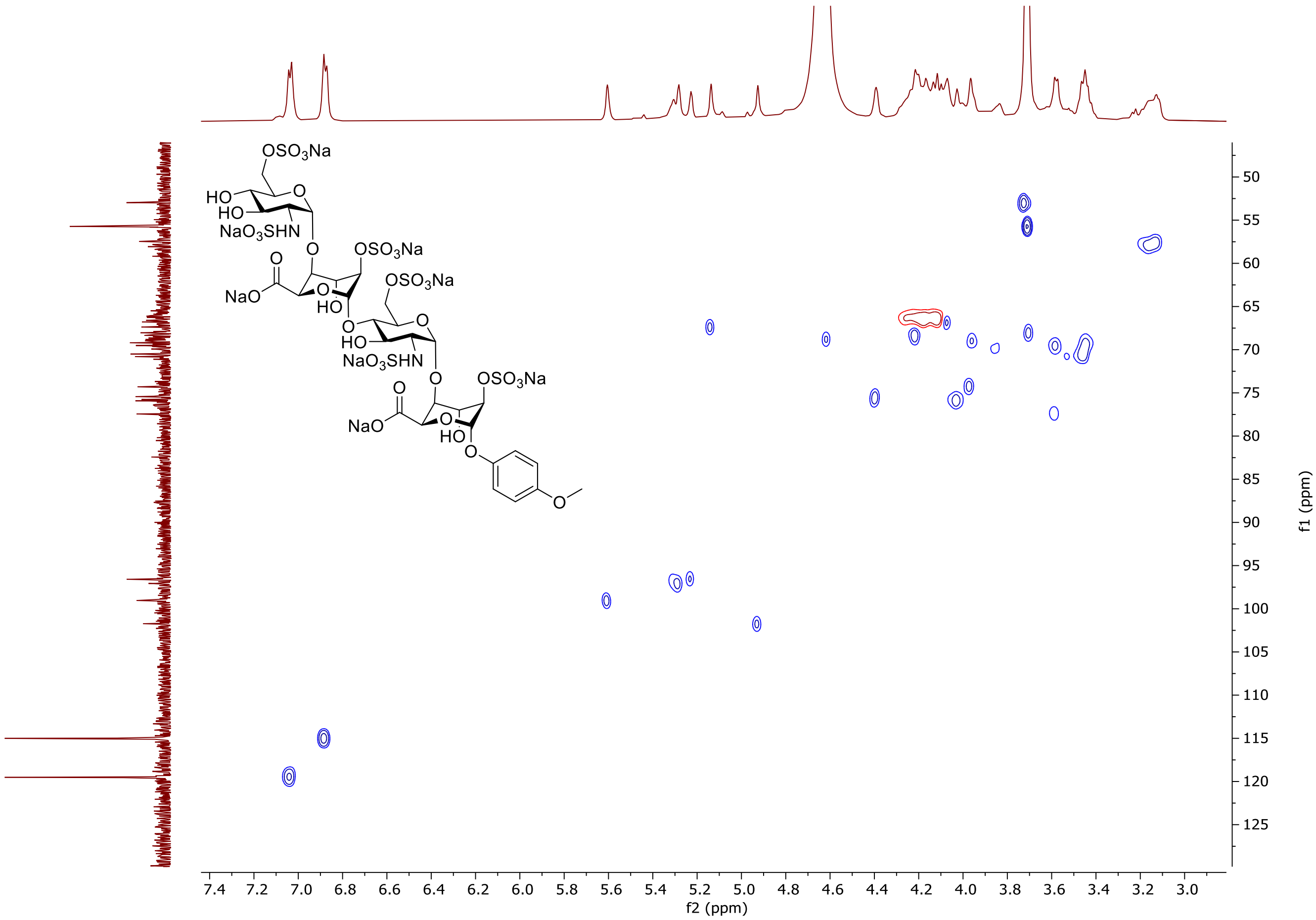

References

(1) Wakpal, J.; Pathiranage, V.; Walker, A. R.; Nguyen, H. M. Rational Design and Expedient Synthesis of Heparan Sulfate Mimetics from Natural Aminoglycosides for Structure and Activity Relationship Studies. *Angew. Chem. Int. Ed.* **2023**, *62* (32), e202304325. DOI: 10.1002/anie.202304325

(2) Orthwein, T.; Huergo, L. F.; Forchhammer, K.; Selim, K. A. Kinetic Analysis of a Protein-protein Complex to Determine its Dissociation Constant (K(D)) and the Effective Concentration (EC(50)) of an Interplaying Effector Molecule Using Bio-layer Interferometry. *Bio. Protoc.* **2021**, *11* (17), e4152. DOI: 10.21769/BioProtoc.4152
